# Predicting hypertension and identifying most important factors among married women in Bangladesh using machine learning approach

**DOI:** 10.1371/journal.pone.0335442

**Published:** 2025-10-30

**Authors:** Novel Chandra Das, Probir Kumar Ghosh, Md. Alamgir Hossain, Uddip Acharjee Shuvo, Nipa Rani Talukder, Fatema Khatun, Mohammad Ziaul Islam Chowdhury

**Affiliations:** 1 International Centre for Diarrhoeal Disease Research, Dhaka, Bangladesh; 2 Institute of information technology, University of Dhaka, Dhaka, Bangladesh; 3 Department of Computer Science and Engineering, North East University, Dhaka, Bangladesh; 4 Department of Psychiatry, University of Calgary, Hospital Drive NW, Calgary, Canada; 5 Provincial Research Data Services, Alberta Health Services, Alberta, Canada; 6 Department of General Educational Development, Daffodil International University, Dhaka, Bangladesh; Khulna University, BANGLADESH

## Abstract

**Introduction:**

Hypertension is a leading contributor to maternal and cardiometabolic morbidity in Bangladesh. We developed and interpreted machine-learning (ML) models to predict hypertension and rank associated factors among married women with the goal of informing targeted screening and policy in low-resource settings.

**Methods:**

We analyzed 4,253 married women from the nationally representative BDHS 2017–18 survey (hypertension prevalence: 23.1%). Twelve ML algorithms were trained under six class-balancing strategies with hyperparameters tuned via random search. Validation used a hold-out test set (80/20) and repeated stratified k-fold cross-validation; bootstrap confidence intervals were estimated for the selected model. Model performance was compared with parametric and non-parametric tests. To interpret results, SHAP was used to rank the top 20 predictors and visualize feature effects. Models quantify associations rather than causation.

**Results:**

The Extra Trees classifier with SMOTE+ENN achieved the best discrimination (F1 = 0.94; AUC-PR = 0.95; ROC-AUC = 0.95). Compared with the original imbalanced training, minority-class detection improved substantially (Extra Trees F1 increased from 0.08 to 0.94; recall from 0.04 to 0.95) while accuracy and ROC-AUC remained relatively stable across samplers. Statistical testing favored SMOTE+ENN for recall, F1, G-mean and AUC-PR. SHAP identified age, parity, recent births, contraceptive use, spousal education and BMI as key predictors. Younger age (<35 years) and normal/underweight status were protective, while parity ≥2–3, husbands’ age ≥ 40 years and overweight/obesity increased risk.

**Conclusions:**

An interpretable ensemble model built primarily on sociodemographic and behavioral variables supplemented by limited biometric markers (BMI, glucose) can accurately flag hypertensive risk among married women in Bangladesh. Findings support programmatic integration of risk scores into eRegistries, routine blood pressure checks in family planning and postpartum visits, husband-focused education/SMS interventions and prioritization of high-parity households in high-risk regions. External validation on BDHS-2022 is planned to assess generalizability.

## Introducvtion

Hypertension is a major contributor to cardiovascular disease and chronic kidney disease, two of the leading causes of death and disability worldwide [1–4]. Globally, an estimated 1.13 billion people are affected with 66.7% residing in low- and middle-income countries (LMICs) where prevalence is rising at an alarming pace, particularly in Asia and Southeast Asia [1,3]. In Bangladesh, prevalence among adults aged ≥35 years has nearly doubled, increasing from 25.7% to 48% in recent decades [5]. Gender differences are striking: women, especially those ≥35 years, show a prevalence of 45% compared to 34% in men, representing a marked rise since 2011 [6–12]. Several nationwide studies confirm that women are disproportionately affected with one reporting 28.9% of women hypertensive versus 23.5% of men [13]. Chronic hypertension in women carries serious implications including higher risks of maternal and neonatal complications [14]. Importantly, multiple studies highlight that married women face even higher hypertension rates than unmarried or never-married women, reflecting the interplay of reproductive demands, economic responsibilities, psychosocial stressors and healthcare disparities [15–18].

Machine learning (ML), a key component of artificial intelligence (AI) has emerged as a transformative tool in healthcare. Unlike traditional regression models, which rely on predefined assumptions of linearity and limited variable interactions, ML methods can process high-dimensional data, capture nonlinear relationships and rank the relative importance of predictors. These advantages have allowed ML to consistently outperform conventional approaches in disease prediction, particularly when datasets are complex or involve interrelated risk factors. At the same time, ML faces limitations: many models require large sample sizes for stability are sensitive to data imbalance and if not properly explained, may be viewed as “black boxes.” Addressing interpretability and ensuring fairness remain essential for public health adoption [19–23].

In cardiovascular research, ML has been applied successfully to echocardiogram analysis and risk prediction for acute decompensated heart failure with models such as K-Nearest Neighbor (KNN), Support Vector Machines (SVM) and ensemble methods achieving strong predictive accuracy [24,25].

For hypertension specifically, ML has demonstrated clear advantages. Tree-based algorithms like random forest (RF) and extreme gradient boosting (XGBoost) have outperformed regression-based methods with XGBoost achieving AUROC values ranging from 0.766 to 1.00 across datasets including 0.894 in semi-laboratory settings when ranking predictors such as systolic blood pressure, waist circumference and albumin levels [26–33]. Recursive feature elimination (RFE) further enhances these models by systematically refining the set of predictors. Comparative studies also confirm that ML surpasses Cox and logistic regression in larger and more complex datasets [34,35]. Hybrid approaches, such as combining RFE with XGBoost, have achieved superior accuracy while models applied to electronic health records capture dynamic features often missed by traditional statistics [36–39].

The utility of ML extends beyond hypertension. In oncology, artificial neural networks and Bayesian networks stratify patients into risk categories while in obesity research, classifiers such as SVMs and quadratic discriminant analysis outperform logistic regression by detecting nonlinear behavioral patterns [40,41]. Nutritional epidemiology studies show k-nearest neighbors and random forests classify cardiometabolic risk more effectively than linear regression [42]. Ensemble models are particularly advantageous in smaller datasets, such as South African studies predicting abnormal angiograms where they outperformed traditional statistical approaches [43]. ML has also been employed for imputing missing data, which improved breast cancer recurrence prediction and for enhancing cerebral ischemia outcome prediction in aneurysmal subarachnoid hemorrhage patients [44,45]. For coronary heart disease survival, SVMs achieved high accuracy [46] while neural networks such as multilayer perceptrons (MLP) and radial basis function (RBF) networks outperformed other classifiers in predicting essential hypertension, demonstrating their ability to capture complex and nonlinear relationships [47]. Notably, gradient boosting methods with RFE outperformed Cox regression and recalibrated Framingham Risk Scores in predicting adverse outcomes in young hypertensive patients, achieving a C-statistic of 0.757 [48].

One persistent challenge in ML health research is data imbalance, which can reduce sensitivity and lead to misclassification of minority outcomes. Methods such as Synthetic Minority Oversampling Technique (SMOTE), random under-sampling (RUS) and cost-sensitive learning have been widely applied to mitigate this issue [36,49–56]. For example, a cost-sensitive deep neural network improved mortality prediction in acute myocardial infarction patients with hypertension by 2.58% AUC compared to ensemble models [57]. SVM models with SMOTE increased accuracy from 91% to 98% [58] while a RUS-applied random forest improved stroke risk prediction among hypertensive adults, yielding AUC 0.624 and sensitivity 63.9% [59].

Interpretability is equally critical. SHAP (Shapley Additive Explanations) has emerged as a powerful, model-agnostic framework that provides consistent and transparent feature attribution [60–65]. Unlike LASSO or ANOVA, SHAP captures nonlinearities and interaction effects, offering granular, instance-level explanations. Visualization tools enhance communication with clinicians, and SHAP’s flexibility in handling missing and imbalanced data makes it highly suitable for real-world datasets [62,65–69]. Moreover, its support for multi-omics integration and human–machine collaboration further enhances its utility in personalized healthcare.

Despite these advances, significant gaps remain. Most hypertension-focused ML studies in South Asia including Bangladesh have concentrated on general adult populations with little attention to married women, who face distinct risks shaped by reproductive roles, domestic workloads and socio-economic constraints. Subgroup-specific validation remains limited, and few studies explicitly integrate cultural and gender-related determinants into predictive models. Although advanced methods such as SMOTE enhance calibration and sensitivity [70–76], they have rarely been applied to this subgroup in Bangladesh. Furthermore, BDHS-2022 was released after our analysis, that is why we trained the model on BDHS-2017–18 and pre-specified external validation on BDHS-2022. This approach ensures robust assessment of generalizability without altering the model development process based on a single additional dataset. Against this backdrop, the present study aims to develop ML models to identify predictive factors of hypertension among married women in Bangladesh. To our knowledge, this is the first study in Bangladesh to apply an extensive set of algorithms combined with class-balancing techniques to this population, contributing both methodological innovation and population-specific insights. Therefore, the objective of this study is to develop and validate interpretable machine-learning models for predicting hypertension among married women in Bangladesh, integrating sociodemographic, behavioral and biometric factors (such as BMI and diabetes status) to identify the most influential predictors and provide evidence to guide targeted screening and public health interventions in low-resource settings

## Methodology

### Data sources

We have used Bangladesh Demographic and Health Survey (BDHS) 2017−18 data in this study, which is the nationally representative survey. The National Institute of Population Research and Training, Medical Education and Family Welfare Division and Ministry of Health and Family Welfare jointly conducted the survey from October 2017 to March 2018.

### Sampling method and sample size/study population and survey design

**Fig 1:**
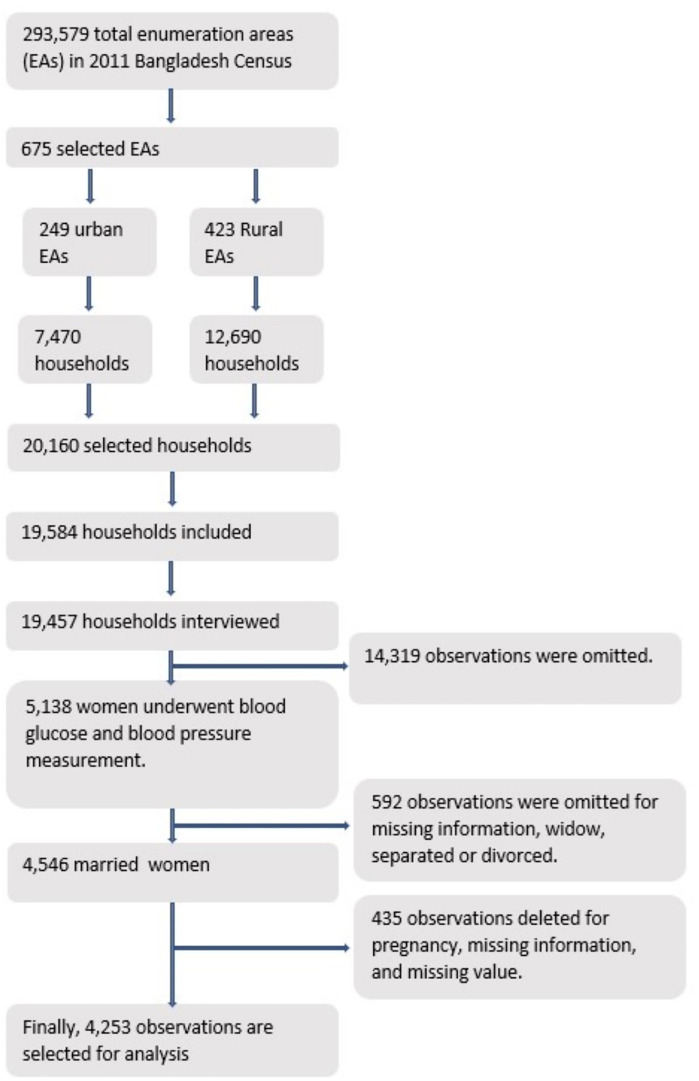
Data selection flow chart.

The Bangladesh Bureau of Statistics of the 2011 Population and Housing Census of the People’s Republic of Bangladesh provided a complete list of enumeration areas (EAs) covering the whole residing population in Bangladesh, which was used in a survey to determine the sampling frame for the 2017−18 BDHS. The survey employed a two-stage stratified cluster sampling as a sampling method where, in the 1st stage of sampling, 675 enumeration areas (EAs) were chosen, whereas 250 EAs were from urban areas and 425 from rural areas with a probability proportional to the EA scale and then a systematic sample of 30 households per EA was chosen in the 2nd stage of sampling to provide statistically accurate estimates of key demographic and health variables for the nation as a whole, rural and urban areas separately and each of the eight divisions. Finally, after selecting 20,250 residential households, approximately 20,100 ever-married women aged 15–49 were expected to complete the interviews [27]. At last, 19,457 households were successfully interviewed and 5,138 women underwent blood pressure and blood glucose measurements. From the 4,546 married women, 4,253 women were considered for final analysis after the termination of pregnant women and deleting missing value or missing information (Fig 1).

In this analytic sample of 4,253 married women, the prevalence of hypertension was about 23.1% (n ≈ 982) ensuring an adequate number of positive events for training supervised ML models. This sample size provides sufficient power for developing and evaluating predictive algorithms in imbalanced health data contexts, consistent with prior methodological recommendations [77–79].

### Dependent feature

Hypertension was considered if the participant’s systolic blood pressure was ≥ 140 mmHg or the diastolic blood pressure was ≥ 90 mmHg or if the person had been taking prescribed medicine to lower blood pressure [80].

### Independent feature

The independent variables in the study are considered from the previous related literature [81–83]. In this study, we considered administrative division of Bangladesh (Barisal, Chittagong, Dhaka, Khulna, Mymensingh, Rajshahi, Rangpur,Sylhet) type of place of residence (urban, rural) respondents highest educational level (no education, primary, secondary and higher)husband/partner educational level (no education, primary, secondary and higher), unmet need for contraception (unmet need for spacing, unmet need for limiting, using for spacing, using for limiting, no unmet need, infecund, menopausal) religion (Islam, Hinduism, Buddhism, Christianity) sex of household head (male, female) wealth index combined (poorest, poorer, middle, richer, richest), current use by method type (no method, folkloric method, traditional method, modern method) currently amenorrhoeic (yes, no) currently abstaining (yes, no) currently residing with husband/partner (living with her, staying elsewhere) household members (< 4 persons and ≥4 persons) respondent’s occupation (working, not working) number of living children (no living children, one, two and more than two children) husband/partner’s occupation (working, not working) respondent’s current age (less than 35 years, 35–40 years and above 40 years old) husband/partner’s age (less than 35 years, 35–40 years and above 40 years old) total children ever born (no children ever born, one, one to three and above three children born) births in last five years (no birth, one and above one) daughters who have died (no died, at least one died) sons who have died (no died, at least one died) age difference between husband/partner and wife (less than ten year, ten and above).

### Derived variables

#### Diabetes status.

Fasting plasma glucose (FPG) was considered to calculate diabetes. The HemoCue Glucose 201 DM system with plasma conversion was used to test a drop of capillary blood obtained from consenting eligible respondents from the middle or ring finger. The system automatically converted the fasting whole blood glucose measurements taken in the survey to FPG equivalent values [84,85]. To classify diabetes World Health Organization (WHO) criteria were used [86]. Diabetes was considered if the FPG level was greater than or equal to 7 mmol/l or self-reported diabetes medication use.

#### Body Mass Index (BMI).

Calculated as weight in kilograms divided by height in meters squared (kg/m²). Categories were defined according to WHO cut-offs: underweight (<18.5), normal (18.5–24.9), overweight (25.0–29.9) and obese (≥ 30) [87].

### Feature selection

All sociodemographic, behavioral, biometric and anthropometric variables with theoretical or empirical relevance to hypertension were extracted from the BDHS 2017–18 dataset. No additional feature engineering or automated feature selection algorithms (e.g., Boruta, LASSO, or recursive feature elimination) were applied. Instead, variable inclusion was guided by existing epidemiological literature and prior BDHS-based hypertension studies.

### Data preparation

#### Missing data.

Cases with missing values were excluded. No imputation was performed to avoid introducing artificial variability.

### Survey weights

The BDHS 2017–18 employs a complex survey design with stratification, clustering and sampling weights to ensure national representativeness. In the present study, we did not apply survey/sample weights because the primary aim was methodological focused on evaluating and comparing the predictive performance of class balancing approaches integrate with machine learning algorithms and ranked risk and protected features rather than estimating population-level prevalence or nationally representative parameters. This approach is consistent with prior ML studies using DHS data in similar contexts [73,88].

### Feature scaling

Data normalization; which is a process of re-scaling the feature value, is very important because most of the machine learning algorithms use Euclidean distance between two data points as a distance metrics, so without Feature scaling, the machine learning algorithms may not execute properly [89]. For rescaling, standardization technique has been applied which the mean is zero and the standard deviation is one.

### Encoding

Categorical variables were transformed using one-hot encoding to allow use in ML algorithms.

### Imbalanced data problem

As imbalanced data lead to the majority class dominates minority class that’s why the, it impacts the reliability of determinations from the dataset, algorithm biased towards majority class and may provide more inaccurate result [90–92]. It is found that for balanced data (where classes proportion are equal) may lead extract best result for identifying the factors. To convey the issue of imbalanced data, Synthetic Minority Oversampling Techniques (SMOTE), Adaptive Synthetic Sampling (ADASYN), Tomek Links (TLs), Edited Nearest Neighbor (ENN), SMOTE-TomekLinks, SMOTE-ENN techniques are applied to resolve the issue (see supplementary File S1 Appendix).

### Machine learning algorithms

We evaluated 12 algorithms: Logistic Regression, Decision Tree, K-Nearest Neighbors (KNN), Random Forest, Extra Trees, AdaBoost, Gradient Boosting Machine (GBM), XGBoost, LightGBM, CatBoost, Support Vector Machine (SVM) and Multilayer Perceptron (MLP).

We included Extra Trees as it is computationally efficient, less prone to overfitting in small subgroups and yields stable feature importance rankings, complementing RF and XGBoost [93–95]. Detailed algorithm descriptions are in Supplementary S2 Appendix.

### Model training with parameter optimization

#### Hold-out cross validation.

Original dataset is divided into training and testing subsets where 80% data belongs to training and the rest 20% belongs to testing subset.

### Repeated stratified k fold cross validation

The dataset is divided into k-folds, where one of the k-folds is selected as a validation set and the remaining sets comprise the training set. Until each one of them forms validation sets, the operation is repeated for each fold, which means for the n number of repetitions the process will be repeated k × n times.

We avoided overfitting and underfitting problems by employing hold-out cross-validation to split training and testing set and to reduce the sampling error, repeated stratified k-fold cross-validation as a validation method was applied. Additionally, we utilized random search to select model parameters using hyper-parameters as there is more chance to select the best parameter [96].

We note that external validation was not possible as BDHS 2022 data was not fully accessible at the time of analysis. This remains a priority for future work to assess model generalizability.

### Evaluation methods

To assess the performance of machine learning methods, we employed confusion matrix, Matthews correlation coefficient, Cohens-kappa, F1-score, G-mean, recall/ sensitivity, specificity, accuracy, precision, AUC-ROC, AUC-PR. For the evaluation of the matric score we used the Anderson-Darling test to check data normality, One-way repeated measure ANOVA was utilized to determine the overall difference among class-balancing techniques, Tukey’s HSD test was used to classify the significance difference among specific group of class balancing techniques, Friedman test was used to find the difference among group instead of one-way ANOVA where normality assumptions are violated (supplementary File S3 Appendix).

### External validation plan

At the time of model development, BDHS-2022 data was not publicly released; consequently, model training and internal validation used BDHS-2017/18. BDHS-2022 is now available and differs modestly in feature scope; to avoid design leakage, we will treat BDHS-2022 strictly as an external test set. Given the scarcity of comparable datasets of the same type, we also reserve BDHS-2022 for validating the deployed system. No retraining or feature re-specification will be performed for the external test; instead, we will apply identical preprocessing and the pre-specified decision threshold. This analysis will provide an out-of-sample assessment of generalizability across a later, post-COVID cohort and any instrument changes.

### Software and hardware

Data preprocessing was performed in STATA 15, ML models in Python (scikit-learn, XGBoost, CatBoost, LightGBM, TensorFlow etc.) and statistical tests in R. Training was carried out on a workstation with Intel Core i5-8365U CPU (1.60 GHz) and 8 GB RAM. While resource limitations constrained deeper architectures and exhaustive grid searches, the chosen algorithms were successfully optimized within these constraints.

### Multicollinearity

To assess multicollinearity, we examined variance inflation factors (VIF) and pairwise correlations among predictors. As expected with one-hot encoded categorical variables, VIF values were inflated to infinity due to perfect linear dependence between dummy categories. Because our primary models were gradient-boosted decision trees, which are robust to correlated features, this did not compromise predictive validity. For clarity, we also inspected pairwise Spearman correlations among key predictors, focusing on socio-demographic and fertility-related variables, which often show natural dependencies.

### Ethical considerations

This study utilized publicly available secondary data from the Bangladesh Demographic and Health Survey (BDHS), which is conducted by the ICF and the Bangladesh Medical Research Council (BMRC). Prior to the data collection, ethical approval was obtained from the Institutional Review Board (IRB) of ICF, USA, and the National Research Ethics Committee of the BMRC. Informed written consent was obtained from all participants involved in the original survey.

As this study involves secondary data analysis, we obtained permission to access the de-identified data from the DHS Program. Since the data was de-identified and publicly available, no additional ethical approval was required for this analysis. The study adhered to the relevant guidelines and regulations for secondary data use.

We additionally highlight that fairness and bias are critical in ML health research. Sensitive variables were treated with caution and SHAP interpretability was used to ensure transparency in feature attribution. These safeguards support responsible use of advanced ML in public health policy.

### Study workflow

**Fig 2:**
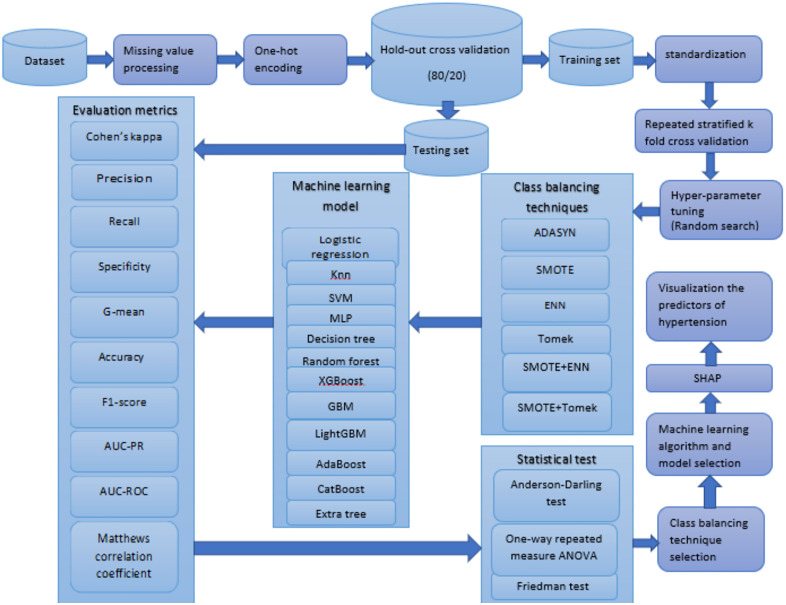
Workflow of machine learning pipeline for hypertension prediction and risk factor ranking of married women in Bangladesh.

An overview of the analytical workflow from data extraction and preprocessing through class balancing, model training, hyperparameter tuning, validation and evaluation, followed by statistical analysis to compare class-balancing techniques and model performance and SHAP-based interpretation is presented in Fig 2**.** This schematic provides a concise visual summary of the methodological pipeline described in the preceding subsections.

## Result

### Outcome characteristics

Among 4,253 married women, 23.1% had hypertension and 76.9% were normotensive.

### Socio-demographic and clinical characteristics of the study participants

Participants were predominantly rural (64.7%). Wealth distribution was approximately even across quintiles (poorest 19.5% to richest 21.9%). Educational attainment was most commonly secondary for women (37.7%) and primary for husbands/partners (32.7%). Median respondent age was 31 years (IQR 25–39); median husband/partner age was 40 years (IQR 32–48). Most respondents were <35 years (61.1%) whereas 41.9% of husbands/partners were >40 years. Fertility profiles showed 51.1% with two to three ever-born children; 59.4% had no births in the past five years. Nutritional status was: underweight 11.7%, normal 53.9%, overweight 27.4%, and obese 7.0. Glycemic categories were: normoglycemia 77.8%, intermediate hyperglycemia 13.1%, hyperglycemia 7.8% and hypoglycemia 1.3% (Table 1).

**Table 1:** Demographic and clinical characteristics of study participants.

Characteristics	Total	Hypertension	95% CI
**Administrative division of Bangladesh**			
Barisal	453 (10.7)	115 (25.4)	21.6–29.6
Chittagong	588 (13.8)	160 (27.2)	23.8–30.9
Dhaka	579 (13.6)	114 (19.7)	16.7–23.1
Khulna	588 (13.8)	136 (23.1)	19.9–26.7
Mymensingh	467 (10.9)	88 (18.8)	15.6–22.6
Rajshahi	580 (13.6)	125 (21.6)	18.5–25.0
Rangpur	551 (12.9)	153 (27.8)	24.2–31.7
Sylhet	447 (10.5)	90 (20.1)	16.6–24.1
**Type of place of residence**			
Urban	1,503 (35.3)	362 (24.1)	21.9–26.5
Rural	2,750 (64.7)	619 (22.5)	20.8–24.3
**Respondent’s highest educational level**			
No education	689 (16.2)	216 (31.3)	27.9–34.9
Primary	1,372 (32.3)	332 (24.2)	21.9–26.7
Secondary	1,605 (37.7)	322 (20.1)	18.2–22.2
Higher	587 (13.8)	111 (18.9)	15.9–22.3
**Husband/partner educational level**			
No education	943 (22.2)	251 (26.6)	23.8–29.5
Primary	1,391 (32.7)	288 (20.7)	18.6–22.9
Secondary	1,240 (29.2)	291 (23.5)	21.2–26.0
Higher	679 (15.9)	151 (22.2)	19.2–25.5
**Unmet need for contraception**			
Unmet need for spacing	172 (4.0)	15 (8.7)	5.3–14.0
Unmet need for limiting	257 (6.0)	59 (23.0)	18.1–28.8
Using for spacing	723 (17.0)	69 (9.9)	7.9–12.4
Using for limiting	2,124 (49.9)	574 (27.1)	25.2–29.1
No unmet need	435 (10.2)	63 (14.5)	11.5–18.0
Infecund/menopausal	542 (12.7)	201 (37.1)	33.2–41.2
**Religion**			
Islam	3,813 (89.7)	855 (22.4)	21.1–23.7
Hinduism	403 (9.5)	114 (28.3)	24.2–32.8
Buddhism	26 (0.6)	8 (30.8)	16.2–51.6
Christianity	11 (0.3)	4 (36.4)	14.5–66.6
**Sex of household head**			
Male	3,724 (87.6)	859 (23.1)	21.8–24.4
Female	529 (12.4)	122 (23.1)	19.8–26.8
**Wealth index combined**			
Poorest	827 (19.5)	154 (18.7)	16.2–21.6
Poorer	833 (19.6)	176 (21.1)	18.4–24.0
Middle	837 (19.7)	194 (23.2)	20.5–26.1
Richer	821 (19.3)	199 (24.2)	21.4–27.3
Richest	935 (21.9)	258 (27.6)	24.7–30.7
**Current use of contraceptive methods**			
No method	1,406 (33.1)	338 (24.1)	21.9–26.4
Folkloric method	14 (0.3)	4 (28.6)	11.7–55.3
Traditional method	455 (10.7)	126 (27.7)	23.7–32.1
Modern method	2,378 (55.9)	513 (21.6)	20.0–23.4
**Currently amenorrhoeic**			
Yes	4,058 (95.4)	948 (23.4)	22.1–24.8
No	195 (4.6)	33 (18.5)	13.5–24.8
**Currently abstaining**			
Yes	4,112 (96.7)	955 (22.2)	20.9–23.6
No	141 (3.3)	26 (18.5)	12.9–26.0
**Currently residing with husband/partner**			
Living with her	3,564 (83.8)	848 (23.8)	22.4–25.2
Staying elsewhere	689 (16.2)	133 (19.3)	16.5–22.5
**Household members**			
< 4 persons	849 (19.9)	221 (26.1)	23.1–29.4
≥ 4 persons	3,404 (80.1)	760 (22.3)	21.0–23.8
**Employment status of respondent**			
Working	2,231 (52.5)	528 (23.7)	21.9–25.6
Not working	2,022 (47.5)	453 (22.4)	20.6–24.3
**Number of living children**			
No living children	275 (6.5)	35 (12.7)	9.3–17.1
One	926 (21.8)	118 (12.7)	10.7–15.1
Two	1,405 (33.0)	316 (22.5)	20.3–24.9
More than two	1,647 (38.7)	512 (31.1)	28.8–33.6
**Employment status of husband/partner**			
Working	4,173 (98.1)	949 (22.7)	21.4–24.0
Not working	80 (1.9)	32 (40.0)	29.9–51.2
**Respondent’s current age**			
< 35 years	2,598 (61.1)	352 (13.5)	12.2–15.0
35–40 years	783 (18.4)	265 (33.9)	30.6–37.3
> 40 years	872 (20.5)	364 (41.7)	38.4–45.1
**Husband/partner’s age**			
< 35 years	1,229 (28.9)	113 (9.2)	7.7–11.0
35–40 years	1,240 (29.2)	243 (19.6)	17.4–22.0
> 40 years	1,784 (41.9)	625 (35.1)	32.9–37.5
**Total children ever born**			
No children ever born	268 (6.3)	31 (11.6)	8.3–16.0
One	844 (19.8)	100 (11.8)	9.7–14.0
Two to three	2,174 (51.1)	548 (25.2)	23.5–27.1
Above three	967 (22.7)	302 (31.2)	28.3–34.3
**Births in last five years**			
No birth	2,526 (59.4)	755 (29.9)	27.9–32.0
One	1,456 (34.2)	193 (13.3)	11.6–15.3
Above one	271 (6.4)	33 (12.2)	8.8–16.7
**Daughters who have died**			
None	3,918 (92.1)	887 (22.6)	21.3–23.9
≥ 1 daughter	335 (7.8)	94 (28.1)	23.6–33.2
**Sons who have died**			
None	3,827 (89.9)	859 (22.4)	21.1–23.7
≥ 1 son	426 (10.1)	122 (28.6)	24.5–33.2
**Age difference between husband/partner and wife**			
< 10 years	3,001 (70.6)	667 (22.2)	20.8–23.6
≥ 10 years	1,252 (29.4)	314 (25.1)	22.7–27.7
**Body mass index (BMI)**			
Underweight	497 (11.7)	55 (11.1)	8.5–14.3
Normal	2,295 (53.9)	406 (17.7)	16.2–19.4
Overweight	1,163 (27.4)	395 (34.0)	31.3–36.9
Obese	298 (7.0)	125 (41.9)	36.5–47.5
**Diabetes**			
Hypoglycemia	56 (1.3)	11 (19.6)	11.5–31.7
Normoglycemia	3,307 (77.8)	706 (21.3)	19.9–22.7
Intermediate hyperglycemia	558 (13.1)	131 (23.5)	20.3–27.0
Hyperglycemia	332 (7.8)	133 (40.1)	34.9–45.6

### Stratification of hypertension among married women in Bangladesh

Table 1 demonstrates that Prevalence varied by division, highest in Rangpur (27.8%) and lowest in Mymensingh (18.8%). Urban residence was associated with higher prevalence than rural (24.1% vs. 22.5%). Prevalence decreased with higher female education (no education 31.3%, higher education 18.9%) and was elevated when husbands had no education (26.6%). Hypertension rose across wealth quintiles (poorest 18.7% to richest 27.6%). Reproductive status mattered: women who were infecund/menopausal had 37.1% prevalence; those using contraception to limit births had 27.1% whereas unmet need for spacing had the lowest (8.7%).

Age gradients were pronounced: < 35 years 13.5%, 35–40 years 33.9% and >40 years 41.7%. Husband/partner age > 40 years was also associated with higher prevalence (35.1%). BMI showed a graded pattern: underweight 11.1%, normal 17.7%, overweight 34.0%, obese 41.9%. By religion, the highest stratum was among Christians (36.4%) and the lowest among Muslims (22.4%), acknowledging small denominators in minority groups. Households with >2 living children had higher prevalence (31.1%). Participants whose husbands/partners were not working had higher prevalence (40.0%) versus those with employed partners (22.7%), noting the small size of the unemployed group (n = 80). Smaller households (<4 persons) showed higher prevalence (26.1%) than larger households (22.3%).

### Class distribution before and after class balancing

To ensure transparency in preprocessing, we report class distributions in the training data before and after class balancing. The original training set contained 3,581 women, with 827 (23.1%) hypertensive and 2,754 (76.9%) normotensive. Both SMOTE and ADASYN achieved perfect balance (50/50). Tomek Links modestly increased the minority proportion to 24.7% by removing borderline majority cases, while ENN shifted the minority share to 35.0%. Hybrid methods produced stronger changes: SMOTE+Tomek restored exact balance (50/50), whereas SMOTE+ENN yielded a minority-dominant distribution (63.3% hypertensive). These comparisons highlight trade-offs between balance and retained sample size across class balancing strategies (Supplementary Table S1 in S4 Appendix).

### Parameter optimization

Model performance shifted with sampling technique. Logistic Regression performed best with L2 (C = 11.29; lbfgs) on the actual data, but under SMOTE/ADASYN/TomekLinks/ENN required lower C, class weighting, or L1 with saga. Extra Trees favored 200 estimators, log2 features, and entropy on actual data; SMOTE reduced trees and used Gini, while ADASYN/TomekLinks retained entropy and increased trees. Decision Trees preferred deeper structures/smaller leaves under SMOTE and SMOTE+ENN; ENN favored Gini. AdaBoost benefited from more estimators and higher learning rates (up to 1.5), often with SAMME.R under ADASYN/ENN. SVMs shifted between RBF and polynomial kernels depending on sampler. XGBoost, LightGBM, KNN, CatBoost, Random Forest, and GBM all exhibited sampler-specific hyperparameter adjustments (Table 2). Unless otherwise noted, cross-model metrics in Table 3 are fold-averaged over stratified 5-fold CV on the training set. For the selected ExtraTrees + SMOTE+ENN model, test-set uncertainty is reported via 95% bootstrap CIs (Table 6).

**Table 2:** Optimal parameters of the machine learning algorithm.

ML model	Sampling	Parameter	ML model	Sampling	Parameter
Logistic Regression	Actual	Penalty = l2, C = 11.288378916846883, Solver = lbfgs, Class_ weight = None, Max _iter = 2500	Extra Trees	Actual	n_ estimators = 200, max_ features = log2, max_ depth = None, min_ samples_ leaf = 6, min_ samples_ split = 76, bootstrap = True, criterion = entropy
	SMOTE	Penalty = l2, C = 1.623776739188721, Solver = liblinear, Class _weight = None, Max _iter = 1000		SMOTE	n_ estimators = 52, max_ features = log2, max_ depth = None, min_ samples_ leaf = 8, min_ samples_ split = 16, bootstrap = False, criterion = gini
	ADASYN	Penalty = l2, C = 0.012742749857031334, Solver = liblinear, Class _weight = balanced, Max _iter = 5000		ADASYN	n_ estimators = 178, max_ features = None, max_ depth = None, min_ samples_ leaf = 2, min_ samples_ split = 10, bootstrap = True, criterion = entropy
	TomekLinks	Penalty = l2, C = 0.0018329807108324356, Solver = lbfgs, Class _weight = balanced, Max _iter = 2500		TomekLinks	n_ estimators = 115, max_ features = None, max_ depth = None, min_ samples_ leaf = 2, min_ samples_ split = 30, bootstrap = True, criterion = entropy
	ENN	Penalty = l1, C = 0.23357214690901212, Solver = saga, Class _weight = None, Max _iter = 2500		ENN	n_ estimators = 157, max_ features = None, max_ depth = None, min_ samples_ leaf = 2, min_ samples_ split = 6, bootstrap = True, criterion = entropy
	SMOTE+ TomekLinks	Penalty = l2, C = 11.288378916846883, Solver = liblinear, Class _weight = None, Max _iter = 5000		SMOTE+ TomekLinks	n_ estimators = 157, max_ features = None, max_ depth = None, min_ samples_ leaf = 6, min_ samples_ split = 18, bootstrap = False, criterion = entropy
	SMOTE+ ENN	Penalty = l1, C = 0.23357214690901212, Solver = liblinear, Class _weight = None, Max _iter = 5000		SMOTE+ ENN	n_ estimators = 94, max_ features = None, max_ depth = None, min_ samples_ leaf = 6, min_ samples_ split = 6, bootstrap = True, criterion = entropy
Decision tree	Actual	Max _depth = 12, Min _samples _split = 44, Min _samples _leaf = 44, Criterion = entropy	AdaBoost	Actual	Algorithm = SAMME.R, n_ estimators = 73, learning_ rate = 0.5
	SMOTE	Max _depth = 28, Min _samples _split = 52, Min _samples _leaf = 4, Criterion = entropy		SMOTE	Algorithm = SAMME, n_ estimators = 136, learning_ rate = 1.5
	ADASYN	Max _depth = 16, Min _samples _split = 48, Min _samples _leaf = 6, Criterion = entropy		ADASYN	Algorithm = SAMME.R, n_ estimators = 178, learning_ rate = 1.5
	TomekLinks	Max _depth = 4, Min _samples _split = 86, Min _samples _leaf = 40, Criterion = entropy		TomekLinks	Algorithm = SAMME, n_ estimators = 115, learning_ rate = 1.5
	ENN	Max _depth = 8, Min _samples _split = 88, Min _samples _leaf = 32, Criterion = gini		ENN	Algorithm = SAMME.R, n_ estimators = 136, learning_ rate = 0.5
	SMOTE+ TomekLinks	Max _depth = 22, Min _samples _split = 52, Min _samples _leaf = 4, Criterion = entropy		SMOTE+ TomekLinks	Algorithm = SAMME.R, n_ estimators = 178, learning_ rate = 1.5
	SMOTE+ ENN	Max _depth = 28, Min _samples _split = 34, Min _samples _leaf = 14, Criterion = gini		SMOTE+ ENN	Algorithm = SAMME.R, n_ estimators = 178, learning_ rate = 1.5
SVM	Actual	C = 0.8, gamma = 0.01, degree = 3,kernel = rbf	XG Boost	Actual	n_ estimators = 136, learning_ rate = 0.05, max_ depth = 10, min_ samples_ split = 38, max_ features = log2
	SMOTE	C = 10, gamma = 0.1, degree = 4, kernel = rbf		SMOTE	n_ estimators = 115, learning_ rate = 0.05, max_ depth = 26, min_ samples_ split = 94, max_ features = sqrt
	ADASYN	C = 0.1, gamma = 0.1, degree = 2, kernel = poly		ADASYN	n_ estimators = 178, learning_ rate = 0.05, max_ depth = 4, min_ samples_ split = 90, max_ features = auto
	TomekLinks	C = 0.8, gamma = 0.1, degree = 3, kernel = poly		TomekLinks	n_ estimators = 52, learning_ rate = 0.05, max_ depth = 2, min_ samples_ split = 18, max_ features = log2
	ENN	C = 0.8, gamma = 0.01 degree = 4,kernel = poly		ENN	n_ estimators = 157, learning_ rate = 0.05, max_ depth = 24, min_ samples_ split = 72, max_ features = auto
	SMOTE+ TomekLinks	C = 0.1,gamma = 1, degree = 4,kernel = poly		SMOTE+ TomekLinks	n_ estimators = 73, learning_ rate = 0.5, max_ depth = 2, min_ samples_ split = 16, max_ features = sqrt
	SMOTE+ ENN	C = 10,gamma = 0.1, degree = 4,kernel = rbf		SMOTE+ ENN	n_ estimators = 178, learning_ rate = 0.05, max_ depth = 26, min_ samples_ split = 68, max_ features = auto
NN	Actual	Activation = identity, Hidden _layer _sizes = (50,100), solver = adam, learning _rate = constant	Light GBM	Actual	n_ estimators = 200, learning_ rate = 0.05, max_ depth = 4
	SMOTE	activation = logistic, hidden _layer _sizes= (50,100), solver = adam, learning _rate = constant		SMOTE	n_ estimators = 94, learning_ rate = 0.05, max_ depth = 28
	ADASYN	activation = relu, hidden _layer _sizes= (100,), solver = adam, learning _rate = constant		ADASYN	n_ estimators = 94, learning_ rate = 0.05, max_ depth = 28
	TomekLinks	activation = identity, hidden _layer _sizes= (50,100), solver = lbfgs, learning _rate = invscaling		TomekLinks	n_ estimators = 178, learning_ rate = 0.005, max_ depth = 14
	ENN	activation = logistic, hidden _layer _sizes= (100,), solver = adam, learning _rate = adaptive		ENN	n_ estimators = 178, learning_ rate = 0.05, max_ depth = 8
	SMOTE+ TomekLinks	activation = relu, hidden_layer_sizes= (100,), solver = adam, learning_rate = constant		SMOTE+ TomekLinks	n_ estimators = 94, learning_ rate = 0.05, max_ depth = 16
	SMOTE+ ENN	activation = tanh, hidden_layer_sizes= (100,), solver = adam, learning_rate = invscaling		SMOTE+ ENN	n_ estimators = 157, learning_ rate = 0.5, max_ depth = 20
KNN	Actual	n_neighbors = 15, weights = distance, algorithm = brute, metric = manhattan	CatBoost	Actual	Depth = 6, learning_ rate = 0.05, iterations = 70, l2_leaf_reg = 9
	SMOTE	n_neighbors = 9, weights = distance, algorithm = kd_tree, metric = manhattan		SMOTE	Depth = 8, learning_ rate = 0.05, iterations = 60, l2_leaf_reg = 3
	ADASYN	n_neighbors = 5, weights = distance, algorithm = auto, metric = manhattan		ADASYN	Depth = 10, learning_ rate = 0.05, iterations = 60, l2_leaf_reg = 5
	TomekLinks	n_neighbors = 15, weights = distance, algorithm = kd_tree, metric = manhattan		TomekLinks	Depth = 6, learning_ rate = 0.05, iterations = 80, l2_leaf_reg = 1
	ENN	n_neighbors = 7, weights = distance, algorithm = ball_tree, metric = manhattan		ENN	Depth = 10, learning_ rate = 0.05, iterations = 80, l2_leaf_reg = 3
	SMOTE+ TomekLinks	n_neighbors = 5, weights = distance, algorithm = auto, metric = manhattan		SMOTE+ TomekLinks	Depth = 10, learning_ rate = 0.5, iterations = 50, l2_leaf_reg = 9
	SMOTE+ ENN	n_neighbors = 7, weights = distance, algorithm = auto, metric = manhattan		SMOTE+ ENN	Depth = 10, learning_ rate = 0.5, iterations = 60, l2_leaf_reg = 1
Random Forest	Actual	n_estimators = 178, max_features = sqrt, max_depth = 20, min_samples_leaf = 6, min_samples_split = 76	GBM	Actual	n_ estimators = 94, learning_ rate = 0.05, max_ depth = 4, max_ features = log2
	SMOTE	n_estimators = 73, max_features = sqrt, max_depth = 28, min_samples_leaf = 4, min_samples_split = 8		SMOTE	n_ estimators = 157, learning_ rate = 0.005, max_ depth = 22, max_ features = log2
	ADASYN	n_estimators = 136, max_features = log2, max_depth = 18, min_samples_leaf = 6, min_samples_split = 42		ADASYN	n_ estimators = 178, learning_ rate = 0.05, max_ depth = 16, max_ features = log2
	TomekLinks	n_estimators = 157, max_features = auto, max_depth = 24, min_samples_leaf = 2, min_samples_split = 32		TomekLinks	n_ estimators = 73, learning_ rate = 0.05, max_ depth = 2, max_ features = sqrt
	ENN	n_estimators = 115, max_features = log2, max_depth = 30, min_samples_leaf = 6, min_samples_split = 4		ENN	n_ estimators = 136, learning_ rate = 0.05, max_ depth = 20, max_ features = log2
	SMOTE+ TomekLinks	n_estimators = 94, max_features = auto, max_depth = 14, min_samples_leaf = 2, min_samples_split = 34		SMOTE+ TomekLinks	n_ estimators = 200, learning_ rate = 0.05, max_ depth = 16, max_ features = sqrt
	SMOTE+ ENN	n_estimators = 157, max_features = sqrt, max_depth = 20, min_samples_leaf = 4, min_samples_split = 34		SMOTE+ ENN	n_ estimators = 178, learning_ rate = 0.05, max_ depth = 24, max_ features = log2

**Table 3:** Performance of the machine learning algorithms.

Model	Class balancing technique	Precision	Recall	F1-score	AUC-ROC	AUC-PR	Cohens-kappa	G-mean	Matthews correlation coefficient	Accu-racy	Specifi-city
Logistic Regression	actual	0.57	0.21	0.31	0.71	0.48	0.21	0.45	0.24	0.78	0.95
Logistic Regression	smote	0.37	0.62	0.46	0.72	0.54	0.24	0.65	0.26	0.67	0.68
Logistic Regression	adasyn	0.35	0.65	0.46	0.71	0.54	0.22	0.65	0.25	0.64	0.64
Logistic Regression	tomeklink	0.36	0.67	0.47	0.72	0.55	0.24	0.66	0.27	0.65	0.64
Logistic Regression	enn	0.38	0.57	0.45	0.71	0.52	0.24	0.64	0.25	0.68	0.72
Logistic Regression	smote+tomek	0.34	0.62	0.44	0.71	0.53	0.21	0.63	0.23	0.64	0.65
Logistic Regression	smote+enn	0.30	0.80	0.44	0.70	0.57	0.16	0.60	0.22	0.53	0.45
Decision Tree	actual	0.55	0.26	0.35	0.70	0.49	0.24	0.49	0.26	0.78	0.94
Decision Tree	smote	0.40	0.34	0.37	0.63	0.45	0.20	0.54	0.20	0.73	0.85
Decision Tree	adasyn	0.34	0.31	0.32	0.61	0.40	0.13	0.50	0.13	0.70	0.82
Decision Tree	tomeklink	0.49	0.35	0.41	0.69	0.50	0.27	0.56	0.27	0.77	0.89
Decision Tree	enn	0.37	0.56	0.45	0.70	0.52	0.23	0.63	0.24	0.68	0.72
Decision Tree	smote+tomek	0.30	0.27	0.28	0.60	0.36	0.08	0.46	0.08	0.69	0.81
Decision Tree	smote+enn	0.34	0.72	0.46	0.69	0.56	0.21	0.64	0.25	0.61	0.57
Neural network	actual	0.53	0.19	0.28	0.71	0.45	0.18	0.42	0.21	0.77	0.95
Neural network	smote	0.30	0.31	0.31	0.59	0.38	0.09	0.49	0.09	0.67	0.78
Neural network	adasyn	0.30	0.29	0.29	0.60	0.38	0.09	0.48	0.09	0.68	0.80
Neural network	tomeklink	0.54	0.26	0.35	0.71	0.48	0.24	0.49	0.26	0.78	0.93
Neural network	enn	0.37	0.59	0.45	0.70	0.53	0.24	0.64	0.25	0.67	0.70
Neural network	smote+tomek	0.32	0.32	0.32	0.61	0.40	0.12	0.51	0.12	0.69	0.80
Neural network	smote+enn	0.33	0.71	0.45	0.69	0.55	0.20	0.63	0.23	0.60	0.56
Knn	actual	0.45	0.18	0.26	0.68	0.41	0.15	0.41	0.17	0.76	0.94
Knn	smote	0.33	0.44	0.38	0.66	0.45	0.15	0.57	0.16	0.66	0.73
Knn	adasyn	0.33	0.42	0.37	0.64	0.44	0.15	0.56	0.15	0.67	0.74
Knn	tomeklink	0.43	0.20	0.28	0.68	0.41	0.15	0.43	0.17	0.75	0.92
Knn	enn	0.36	0.58	0.44	0.67	0.52	0.22	0.63	0.23	0.66	0.69
Knn	smote+tomek	0.33	0.41	0.36	0.64	0.44	0.15	0.55	0.15	0.67	0.75
Knn	smote+enn	0.32	0.76	0.45	0.67	0.56	0.18	0.62	0.23	0.57	0.51
Random Forest	actual	0.72	0.07	0.12	0.71	0.50	0.09	0.26	0.17	0.78	0.99
Random Forest	smote	0.45	0.33	0.38	0.69	0.47	0.23	0.54	0.24	0.75	0.88
Random Forest	adasyn	0.42	0.42	0.42	0.71	0.49	0.25	0.59	0.25	0.73	0.82
Random Forest	tomeklink	0.56	0.18	0.28	0.70	0.47	0.18	0.42	0.22	0.78	0.96
Random Forest	enn	0.37	0.58	0.46	0.71	0.53	0.24	0.64	0.25	0.68	0.71
Random Forest	smote+tomek	0.42	0.38	0.40	0.71	0.47	0.23	0.57	0.23	0.73	0.84
Random Forest	smote+enn	0.34	0.73	0.47	0.71	0.57	0.22	0.65	0.26	0.61	0.58
Extra tree	actual	0.80	0.04	0.08	0.71	0.53	0.06	0.20	0.15	0.78	1.00
Extra tree	smote	0.39	0.52	0.45	0.70	0.51	0.25	0.63	0.25	0.71	0.76
Extra tree	adasyn	0.41	0.40	0.40	0.70	0.47	0.23	0.58	0.23	0.73	0.82
Extra tree	tomeklink	0.57	0.20	0.29	0.70	0.47	0.20	0.44	0.24	0.78	0.95
Extra tree	enn	0.35	0.57	0.43	0.70	0.51	0.21	0.62	0.22	0.66	0.69
Extra tree	smote+tomek	0.42	0.47	0.44	0.70	0.50	0.26	0.61	0.26	0.73	0.80
Extra tree	smote+enn	0.92	0.95	0.94	0.95	0.95	0.79	0.89	0.79	0.91	0.83
Adaboost	actual	0.57	0.18	0.28	0.71	0.47	0.19	0.42	0.23	0.78	0.96
Adaboost	smote	0.50	0.33	0.40	0.71	0.49	0.27	0.55	0.27	0.77	0.90
Adaboost	adasyn	0.48	0.22	0.30	0.70	0.44	0.18	0.45	0.21	0.77	0.93
Adaboost	tomeklink	0.55	0.28	0.37	0.71	0.49	0.25	0.51	0.27	0.78	0.93
Adaboost	enn	0.38	0.58	0.46	0.71	0.53	0.24	0.64	0.26	0.68	0.71
Adaboost	smote+tomek	0.52	0.23	0.32	0.70	0.46	0.21	0.47	0.23	0.77	0.93
Adaboost	smote+enn	0.35	0.62	0.45	0.71	0.53	0.22	0.64	0.24	0.65	0.66
XG Boost	actual	0.43	0.25	0.32	0.66	0.43	0.18	0.47	0.19	0.75	0.90
XG Boost	smote	0.43	0.29	0.35	0.65	0.44	0.20	0.51	0.20	0.75	0.88
XG Boost	adasyn	0.54	0.29	0.37	0.71	0.50	0.26	0.51	0.28	0.78	0.93
XG Boost	tomeklink	0.52	0.09	0.15	0.71	0.41	0.09	0.29	0.14	0.77	0.98
XG Boost	enn	0.34	0.60	0.44	0.67	0.52	0.20	0.63	0.22	0.64	0.65
XG Boost	smote+tomek	0.55	0.26	0.35	0.70	0.49	0.24	0.49	0.27	0.78	0.94
XG Boost	smote+enn	0.35	0.64	0.45	0.70	0.54	0.22	0.64	0.24	0.64	0.64
LGBM	actual	0.56	0.25	0.35	0.70	0.49	0.24	0.48	0.26	0.78	0.94
LGBM	smote	0.48	0.30	0.37	0.70	0.47	0.23	0.52	0.24	0.76	0.90
LGBM	adasyn	0.47	0.28	0.35	0.70	0.46	0.21	0.50	0.22	0.76	0.91
LGBM	tomeklink	0.50	0.03	0.06	0.71	0.38	0.03	0.17	0.08	0.77	0.99
LGBM	enn	0.34	0.56	0.42	0.68	0.50	0.19	0.61	0.20	0.65	0.67
LGBM	smote+tomek	0.48	0.27	0.35	0.70	0.46	0.22	0.50	0.23	0.76	0.91
LGBM	smote+enn	0.34	0.63	0.45	0.69	0.53	0.21	0.64	0.23	0.64	0.64
CatBoost	actual	0.58	0.11	0.18	0.71	0.45	0.12	0.32	0.18	0.78	0.98
CatBoost	smote	0.46	0.37	0.41	0.71	0.49	0.26	0.57	0.26	0.75	0.87
CatBoost	adasyn	0.48	0.34	0.40	0.71	0.49	0.26	0.55	0.26	0.76	0.89
CatBoost	tomeklink	0.59	0.21	0.31	0.70	0.49	0.21	0.45	0.25	0.78	0.96
CatBoost	enn	0.38	0.61	0.47	0.71	0.54	0.26	0.66	0.27	0.68	0.70
CatBoost	smote+tomek	0.40	0.29	0.34	0.66	0.43	0.18	0.50	0.18	0.74	0.87
CatBoost	smote+enn	0.35	0.67	0.46	0.70	0.55	0.22	0.65	0.25	0.63	0.62
GBM	actual	0.63	0.17	0.27	0.71	0.50	0.19	0.41	0.25	0.79	0.97
GBM	smote	0.46	0.25	0.32	0.68	0.44	0.19	0.48	0.21	0.76	0.91
GBM	adasyn	0.47	0.24	0.32	0.69	0.44	0.19	0.47	0.20	0.76	0.92
GBM	tomeklink	0.58	0.13	0.21	0.71	0.45	0.14	0.35	0.19	0.78	0.97
GBM	enn	0.36	0.59	0.45	0.69	0.52	0.22	0.63	0.24	0.66	0.68
GBM	smote+tomek	0.46	0.26	0.33	0.69	0.45	0.20	0.49	0.21	0.76	0.91
GBM	smote+enn	0.32	0.76	0.45	0.69	0.57	0.19	0.63	0.23	0.58	0.52
SVM	actual	0.68	0.07	0.12	0.67	0.48	0.08	0.26	0.16	0.78	0.99
SVM	smote	0.35	0.04	0.07	0.64	0.30	0.03	0.20	0.05	0.76	0.98
SVM	adasyn	0.31	0.45	0.37	0.62	0.44	0.13	0.56	0.13	0.64	0.70
SVM	tomeklink	0.35	0.37	0.36	0.62	0.44	0.17	0.54	0.17	0.70	0.80
SVM	enn	0.41	0.08	0.13	0.67	0.35	0.06	0.27	0.09	0.76	0.97
SVM	smote+tomek	0.34	0.37	0.35	0.61	0.43	0.15	0.54	0.15	0.69	0.79
SVM	smote+enn	0.31	0.34	0.32	0.63	0.40	0.11	0.51	0.11	0.67	0.77

**Table 6:** Test-set performance of ExtraTrees with SMOTE+ENN using 1,000-replicate nonparametric bootstrap (n = 672).

Metric	Point estimate	95% CI
Precision	0.92	0.90–0.94
Recall	0.95	0.93–0.97
F1-score	0.94	0.92–0.96
Accuracy	0.91	0.89–0.94
Specificity	0.83	0.80–0.87
ROC-AUC	0.95	0.93–0.97
PR-AUC	0.95	0.93–0.97
Cohen’s κ	0.79	0.75–0.83
MCC	0.79	0.75–0.83
G-mean	0.89	0.86–0.92

### Classification efficacy and confusion matrix

**Fig 3:**
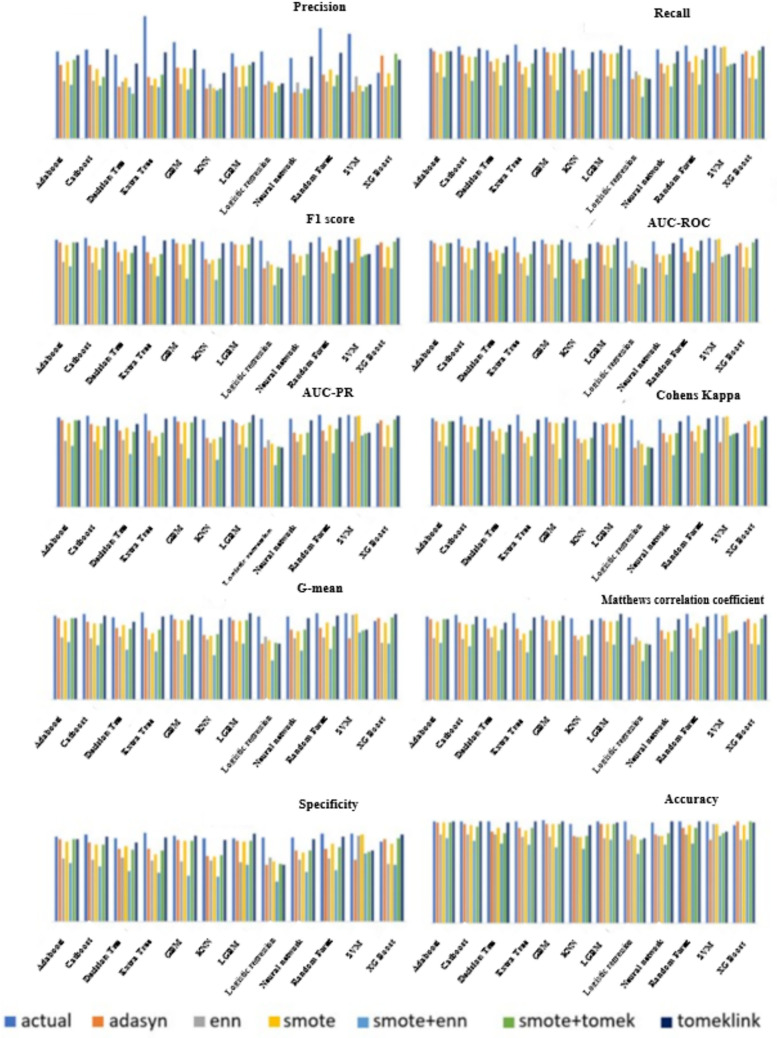
Performance of the machine learning algorithms.

Across classifiers, training on the original imbalanced data produced inflated accuracy and specificity but markedly poor minority sensitivity (low recall and F1), evidencing majority-class bias (Table 3; Fig 3). For example, ExtraTrees (Actual) achieved Accuracy = 0.78, Specificity = 1.00, Recall = 0.04 and F1 = 0.08, consistent with its confusion matrix on the original test split (Table 4). After applying SMOTE+ENN, ExtraTrees improved substantially F1 = 0.94, Recall = 0.95, Cohen’s κ = 0.79, MCC = 0.79 and G-mean = 0.89 in agreement with the corresponding SMOTE+ENN confusion matrix (Table 5). These matched matrix–metric pairs ensure internal coherence. Cross-model medians (Friedman ranks) indicated that TomekLinks tended to yield the highest Accuracy and Specificity overall (Supplementary Table S2E in S4 Appendix) whereas on the independent test set ExtraTrees with SMOTE+ENN achieved the highest Accuracy among our models (0.91 vs. 0.78 on the original data), underscoring that balancing improved clinically relevant discrimination.

**Table 4:** 2 × 2 confusion matrix of ExtraTrees model predictions integrated with original sample.

	Predicted Negative	Predicted Positive
**Actual Negative**	518	2
**Actual Positive**	146	6

**Table 5:** 2 × 2 confusion matrix of ExtraTrees model predictions integrated with SMOTEENN class balancing technique.

	Predicted Negative	Predicted Positive
**Actual Negative**	185	27
**Actual Positive**	20	440

### Global comparison of class-balancing techniques

ML algorithms exhibited varying performance across balancing techniques, motivating formal statistical testing. Except for AUC-PR, most metrics were non-normal by Anderson–Darling (Supplementary S2A Table in S4 Appendix), so ANOVA was restricted to AUC-PR. Repeated-measures ANOVA on AUC-PR showed significant differences across techniques (Supplementary S2B Table in S4 Appendix), and Tukey’s HSD identified SMOTE+ENN and ENN as significantly superior for AUC-PR in multiple pairwise contrasts (Supplementary S2C Table in S4 Appendix). For the remaining non-normal metrics, Friedman tests showed significant omnibus differences (Supplementary S2D Table). Post-hoc rank summaries indicated SMOTE+ENN led G-mean, Recall, and F1 (ENN second) while TomekLinks ranked highest for Accuracy, Specificity, Precision, and AUROC (Supplementary Table S2E in S4 Appendix).

Boxplots of AUCPR, F1, Recall, Precision, Specificity, G-mean, Accuracy and AUC-ROC (Fig 4–11) illustrate these trends: SMOTE+ENN achieved the highest Recall, F1 and AUC-PR, whereas the original data scored highest in Accuracy and Specificity.

**Fig 4:**
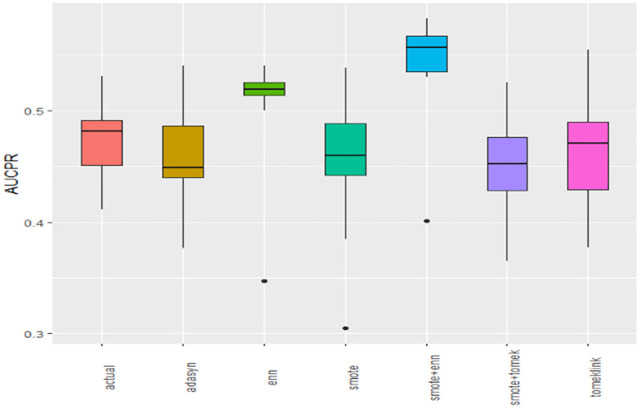
The Boxplot of the original data and class balancing techniques based on AUC-PR.

**Fig 5:**
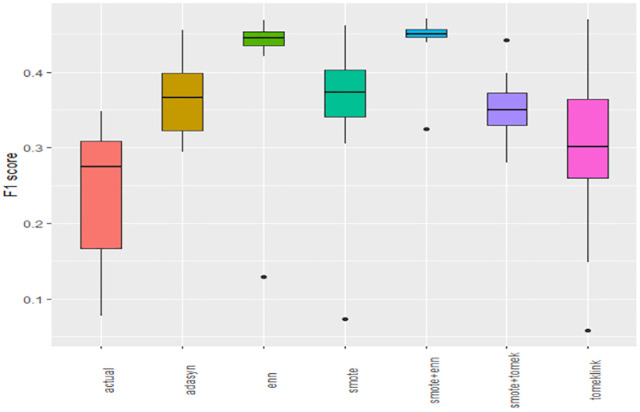
The Boxplot of the original data and class balancing techniques based on F1 scores.

**Fig 6:**
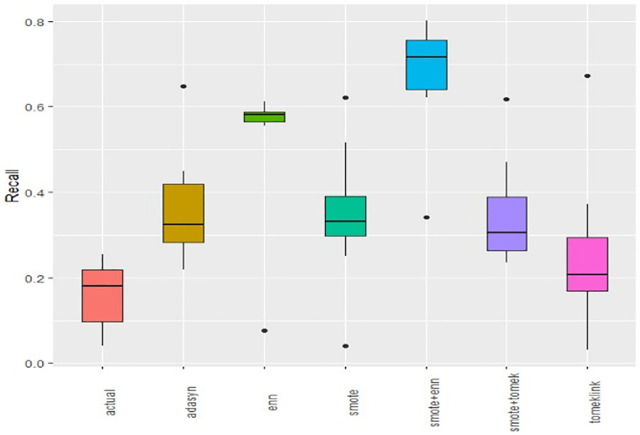
The Boxplot of the original data and class balancing techniques based on Recall.

**Fig 7:**
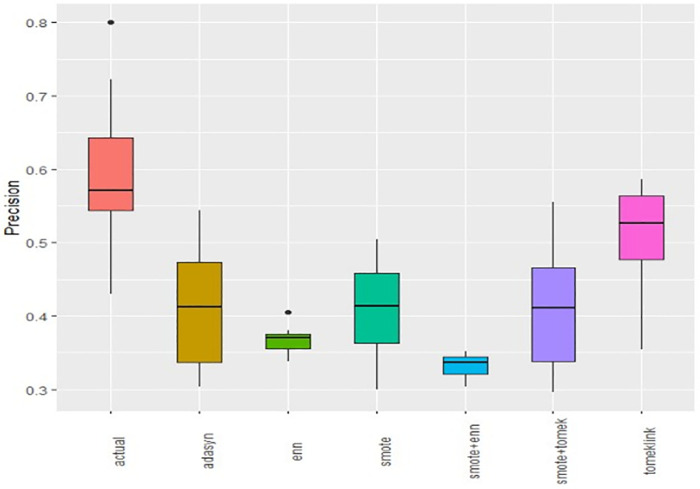
The Boxplot of the original data and class balancing techniques based on Precision.

**Fig 8:**
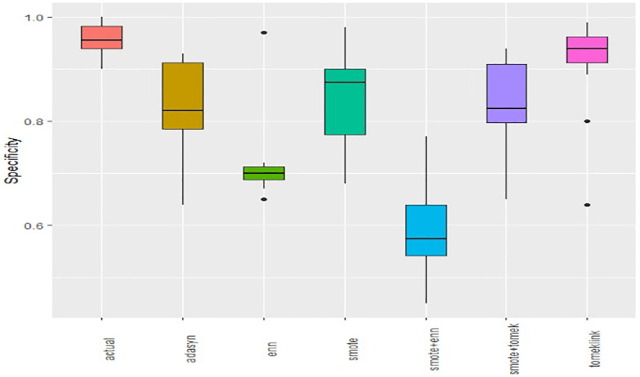
The Boxplot of the original data and class balancing techniques based on Specificity.

**Fig 9:**
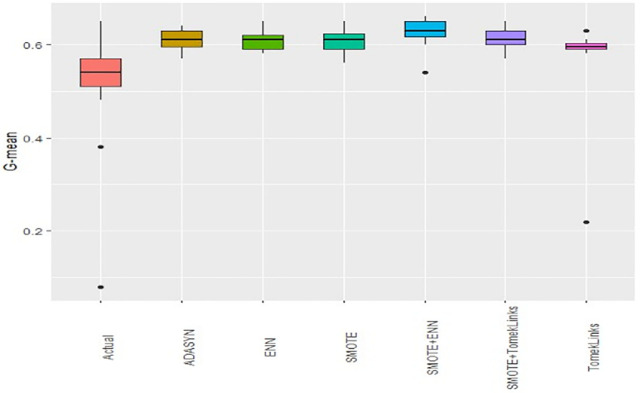
The Boxplot of the original data and class balancing techniques based on G-mean.

**Fig 10:**
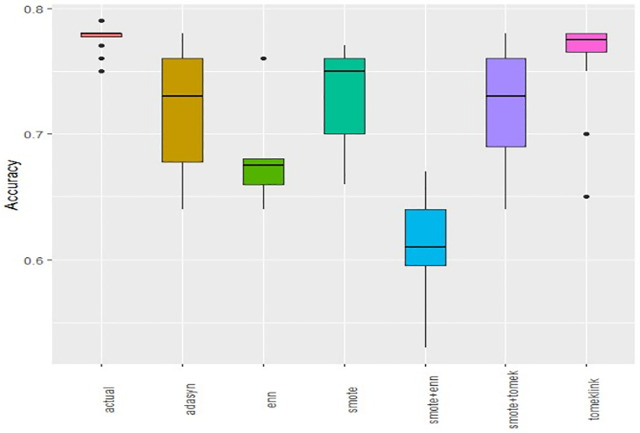
The Boxplot of the original data and class balancing techniques based on Accuracy.

**Fig 11:**
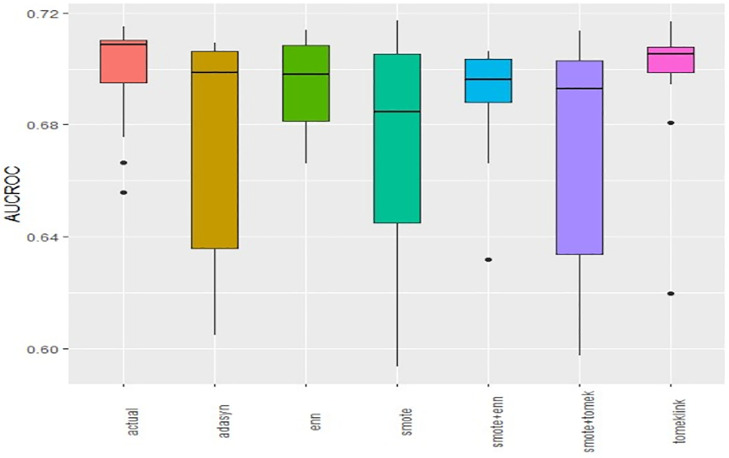
The Boxplot of the original data and class balancing techniques based on AUCROC.

### Feature importance

**Fig 12:**
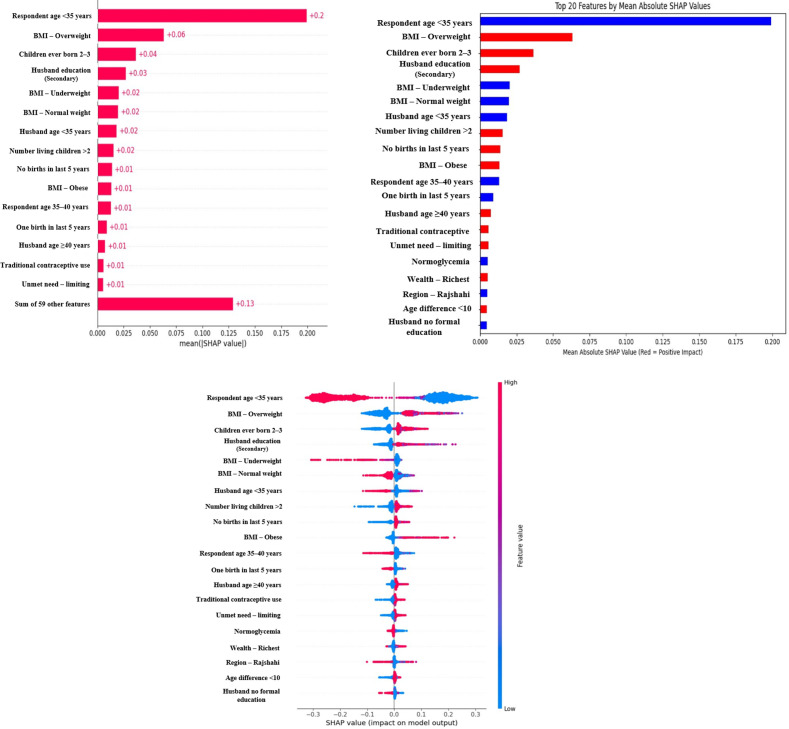
a. The factors importance of hypertension among married women in Bangladesh. b. The classification of positive and negative predictors of hypertension of married women in Bangladesh. c. The impact of the factors of married women's hypertension in Bangladesh.

Integrating SHAP with a trained model enabled analysis of global and local predictors. Figure 12a shows the most important features; Fig 12b classifies the top 20 factors by direction of association; Fig 12c presents a SHAP summary plot combining feature importance and effects.

In Fig 12a, respondent age < 35 years was the most influential factor, followed by overweight, having two to three ever-born children, and husband/partner with secondary education. Additional contributors included underweight, normal weight, husband/partner <35 years, > 2 living children, no births in the last five years and obesity.

Fig 12b indicates negative associations (blue) for age < 35, underweight/normal weight, husband/partner <35 years, respondent age 35–40, one child in last five years, normoglycemia, residence in Rajshahi and no formal education for husband/partner. Positive associations (red) included overweight, two to three ever-born children, husband/partner with secondary education, > 2 living children, no births in five years, obesity, husband/partner >40, traditional contraceptive use, unmet need for limiting, richest quintile and age difference ≥10 years.

Fig 12c shows these effects at the individual level: blue points (low SHAP values) correspond to features decreasing predicted risk, while red/purple points (high SHAP values) correspond to features increasing predicted risk.

### Internal overfitting diagnostics

Repeated stratified five-fold CV yielded an average F1 of 0.934 ± 0.012; nested CV showed an outer-fold mean F1 of 0.965 ± 0.010; the held-out test F1 was 0.9447 (Supplementary Table S3A in S4 Appendix). Learning-curve behavior showed convergence between training and validation performance as sample size increased, supporting strong generalization while acknowledging that optimism is possible without external validation (S1 Fig).

### Test-set performance with bootstrap confidence intervals

On the independent test set (n = 672), ExtraTrees + SMOTE+ENN achieved Precision 0.92 (95% CI 0.90–0.94), Recall 0.95 (0.93–0.97), F1 0.94 (0.92–0.96), Accuracy 0.91 (0.89–0.94), Specificity 0.83 (0.80–0.87), ROC-AUC 0.95 (0.93–0.97), PR-AUC 0.95 (0.93–0.97), Cohen’s κ 0.79 (0.75–0.83), MCC 0.79 (0.75–0.83) and G-mean 0.89 (0.86–0.92) (Table 6).

### Multicollinearity

Diagnostics indicated structurally inflated VIFs under one-hot encoding. Correlation screening revealed expected dependencies (e.g., respondent age < 35 vs. husband age ≥ 40, ρ = −0.82; mutually exclusive recent-birth categories, ρ = −0.89), along with dependencies among BMI categories, parity, and living-children counts. These reflect structural collinearities inherent to categorical coding. Tree-based models such as ExtraTrees are robust to these correlations and predictive performance was unaffected (Supplementary Table S5 in S4 Appendix).

### Sample-flow transparency and class balance across stages

A CONSORT-style accounting of the analytic sample is shown in Supplementary Table S4A in S4 Appendix. No rows were lost to missingness; subsequent splits yielded the training and test sets used for modeling. Stratified 5-fold CV preserved the original training prevalence (23.1% hypertensive) in each fold (Supplementary Table S4B in S4 Appendix). Class balancing (e.g., SMOTE+ENN) was applied inside the training portion of each fold only; the final ExtraTrees model was refit on the full training set using SMOTE+ENN, yielding a minority-dominant training balance (Supplementary Table S4C in in S4 Appendix). The independent test set (n = 672) remained untouched.

### Calibration and thresholding

The ExtraTrees model was well calibrated (Brier score = 0.0626; S2 Fig). The Youden-optimal decision threshold was 0.55, at which Youden’s J was 0.859 (Supplementary Table S3B in S4 Appendix). Unless noted, confusion matrices and test-set metrics were computed at threshold 0.55.

Threshold consistency: Unless noted, the confusion matrix and all bootstrap test-set metrics were computed at the 0.55 decision threshold.

### Fairness/ stratified performance

Subgroup analyses were generally strong, though some divisions showed slightly lower Accuracy and F1 despite perfect discrimination (ROC-AUC = 1.0). For example, in Rajshahi, Accuracy and F1 were modestly reduced relative to overall performance (Supplementary Table S6 in S4 Appendix).

## Discussion

This study systematically evaluated 12 machine-learning (ML) algorithms with six class balancing strategies, integrating SHAP (SHapley Additive exPlanations) to identify factors associated with hypertension among married women in Bangladesh. Extra Trees combined with SMOTE+ENN (Synthetic Minority Over-sampling Technique + Edited Nearest Neighbors) achieved the best performance (F1 = 0.94; AUC-PR = 0.95). Gains were largest for recall, F1, G-mean and AUC-PR metrics better suited to imbalanced data while precision, specificity, accuracy and AUC-ROC were comparatively stable across samplers (Figs 4–11).

Our findings align with global work yet add context-specific insights. An Ethiopian stacking/XGBoost model reported slightly higher performance (F1 = 96.5%, AUC = 0.97) on a much smaller sample (n = 612) emphasizing clinical/lifestyle predictors [97]. In contrast, our socially grounded features parity, spousal education, contraceptive use performed strongly in a nationally representative cohort. Similarly, studies from Malaysia, South Korea, Japan and Norway reported AUCs ≈ 0.78–0.87 using largely clinical markers, whereas our sociodemographic model reached AUC-ROC = 0.95 [37,98–100]. Innovative approaches using wearable ECG (AUC = 0.83) or echocardiography (AUC = 0.87) show promise but have limited population-level generalizability [101,102]. A quantum-enhanced ML study reported very high scores (F1 = 98.9%, accuracy = 98.4%), but our results underscore that carefully tuned classical ML remains highly effective and interpretable for population-specific public-health applications [103]. Quantitatively, our AUC-ROC exceeded several large clinical models by +0.08 to +0.17 absolute AUC (0.95 vs. 0.78–0.87) and our accuracy was + 13 percentage points higher than Asadullah et al. (91% vs. 78%) [70].

Why emphasize PR over ROC? Precision–recall curves are more informative when the positive class is rare and accuracy can be misleadingly high due to the majority class; F1 and AUC-PR therefore provide a truer picture of minority-class performance [104–108]. Across algorithms, ensembles especially Extra Trees with SMOTE+ENN were most effective for imbalanced health data, consistent with prior reports of SMOTE+ENN paired with Random Forest, XGBoost, LightGBM, or stacked models [109–113]. The analytic sample (N = 4,253; ~ 23.1% hypertensive) provided sufficient positive events for supervised learning. Although several sociodemographic variables are correlated (e.g., age, parity, recent births; BMI categories), tree-based ensembles are robust to redundancy; sensitivity analyses that dropped one variable per correlated cluster left the test F1 unchanged within bootstrap uncertainty, supporting stability while preserving program-relevant interpretability.

SHAP analyses consistently highlighted age, parity, recent births, contraceptive use and spousal education as influential predictors (Figs 12a–c). Younger age (<35 years) was protective, whereas women aged 35–40 or with husbands ≥40 years showed higher risk. The positive association with husbands’ secondary education counter to expectation may reflect socioeconomic stressors or confounding with wealth and fertility; this hypothesis warrants social-epidemiological follow-up. Our results corroborate prior Bangladesh-based work linking contraceptive use, women’s age, husband’s education and number of living children to hypertension risk, though earlier studies did not focus specifically on married women [114–117]

Given high recall at the Youden-optimal threshold (0.55), health systems could embed risk scoring in digital registers (e.g., eRegistries) and during family-planning or postpartum visits to triage married women for blood-pressure checks. Three actionable levers emerge: (i) integrate BP screening into contraceptive counselling and postpartum follow-up; (ii) engage husbands/partners via brief education or SMS nudges where partner age/spousal education elevate risk; and (iii) target high-parity households for home BP monitoring and referral. Geographic gradients (e.g., higher risk in Rajshahi) support district-tailored outreach. Diet-focused strategies (e.g., green coffee supplementation; DASH/Mediterranean patterns) offer complementary, population-specific interventions [118,119].

SHAP values explain model associations, not causal effects; correlated social and fertility indicators may act as proxies. Apparent positive associations (e.g., husbands’ secondary education) could reflect unmeasured confounding (wealth, occupation, stress). Ethical deployment should address fairness (monitor subgroup performance gaps), transparency (document model versioning and thresholds) and potential unintended consequences (e.g., stigma or resource diversion). Thresholds should be calibrated to local prevalence and the relative costs of false negatives vs. false positives.

Internal checks repeated CV, nested CV and bootstrap Cis showed stable performance with tight uncertainty (see Results); however, training solely on BDHS-2017–18 may still inflate apparent performance. We have pre-specified external validation on BDHS-2022, applying identical preprocessing and the pre-set decision threshold (see Methods) to evaluate discrimination, calibration and transportability. This will test robustness in a later, post-COVID cohort and under any instrument changes. Findings do not generalize to unmarried women.

## Conclusion

Using BDHS 2017–18 data, we compared 12 ML algorithms and six class balancing methods for predicting hypertension among married women. Extra Trees + SMOTE+ENN was optimal (F1 = 0.94; AUC-PR = 0.95). SHAP surfaced actionable, context-specific predictors women’s age, contraceptive use, parity, spousal education and household headship underscoring the value of social and demographic information when clinical data are scarce. Metrics tailored to imbalance (F1, AUC-PR) were more informative than accuracy or AUC-ROC alone.

These models can support screening and targeting in eRegistries and digital health platforms, enabling gender-sensitive, resource-aware outreach in LMICs. Policymakers can leverage findings for spousal-education initiatives, parity-focused home BP programs and region-specific strategies.

Priorities include external validation with BDHS 2022; prospective studies; integration of clinical/biometric markers; and exploration of transfer learning, cross-country adaptation and multi-omics integration**.** Linking models to real-time eRegistry streams could enable adaptive thresholding and continuous monitoring. Extending analyses to unmarried women and other under-represented groups will refine generalizability.

### Strengths and limitations

#### Strengths

(i) First Bangladesh study (to our knowledge) to combine class-balancing and ML for hypertension prediction specifically among married women; (ii) transparent feature attribution via SHAP; (iii) rigorous evaluation with repeated CV, nested-CV, test-set bootstrapping and comparative statistics across class balancing strategies; (iv) concrete policy pathways (integration with family-planning/postpartum services, spousal engagement, district tailoring).

### Limitations

(i) No external validation yet; BDHS may under-sample marginalized groups; findings do not extend to unmarried women; (ii) important biomedical/genetic/environmental determinants were unavailable; (iii) cross-sectional design limits causal inference SHAP explains predictions, not causes; (iv) fairness audits revealed subgroup gaps requiring monitoring and mitigation; (v) training-time class balancing alters class balance relative to the population accordingly, we calibrated thresholds on the untouched test set and reported bootstrap CIs to reduce optimism; (vi) deployment should account for gender-specific barriers in Bangladesh (mobility constraints, caregiving burden, norms around clinic attendance). Another limitation is the lack of external validation at the time of analysis, as BDHS 2022 was not released then. Since the dataset is now available, we plan to validate our deployed system using BDHS 2022, though its limited feature scope prevents full integration into model training.

## Supporting information

S1 Appendix List of class balancing techniques. (DOCX)

S2 Appendix Evaluation of machine learning algorithms. (DOCX)

S3 Appendix Evaluation of machine learning algorithms. (DOCX)

S4 Appendix Supplementary Tables. (DOCX)

S1 Fig Learning curve of the ExtraTrees model for predicting hypertension among married women in Bangladesh. (DOCX)

S2 Fig Reliability plot of the ExtraTrees model for predicting hypertension among married women in Bangladesh. (DOCX)

## References

[1] Hay SI, Abajobir AA, Abate KH, Abbafati C, Abbas KM, Abd-Allah F, et al. Global, regional, and national disability-adjusted life-years (DALYs) for 333 diseases and injuries and healthy life expectancy (HALE) for 195 countries and territories, 1990–2016: a systematic analysis for the Global Burden of Disease Study 2016. 2017;390(10100):1260–344. 10.1016/S0140-6736(17)32130-X PMC5605707 28919118

[2] Rahman MA, Parvez M, Halder HR, Yadav UN, Mistry SK. Prevalence of and factors associated with prehypertension and hypertension among Bangladeshi young adults: An analysis of the Bangladesh Demographic and Health Survey 2017–18. Clinical Epidemiology and Global Health. 2021;12:100912. doi: 10.1016/j.cegh.2021.100912

[3] Forouzanfar MH, Liu P, Roth GA, Ng M, Biryukov S, Marczak L. Global burden of hypertension and systolic blood pressure of at least 110 to 115 mm Hg, 1990-2015. JAMA. 2017;317(2):165–82. 28097354 10.1001/jama.2016.19043

[4] Ghosh PK, Harun MGD, Shanta IS, Islam A, Jannat KKE, Mannan H. Prevalence and determinants of hypertension among older adults: A comparative analysis of the 6th and 8th national health surveys of Bangladesh. PLoS One. 2023;18(10):e0292989. doi: 10.1371/journal.pone.0292989 37844103 PMC10578599

[5] Kibria GMA, Swasey K, Choudhury A, Burrowes V, Stafford KA, Uddin SMI, et al. The new 2017 ACC/AHA guideline for classification of hypertension: changes in prevalence of hypertension among adults in Bangladesh. J Hum Hypertens. 2018;32(8–9):608–16. doi: 10.1038/s41371-018-0080-z 29899377 PMC6487869

[6] Farrukh F, Abbasi A, Jawed M, Almas A, Jafar T, Virani SS, et al. Hypertension in Women: A South-Asian Perspective. Frontiers in Cardiovascular Medicine. 2022;9. 10.3389/fcvm.2022.880374 PMC9399392 36035921

[7] Kearney PM, Whelton M, Reynolds K, Muntner P, Whelton PK, He J. Global burden of hypertension: analysis of worldwide data. Lancet. 2005;365(9455):217–23. doi: 10.1016/S0140-6736(05)17741-1 15652604

[8] Rahimi K, Emdin CA, MacMahon S. The epidemiology of blood pressure and its worldwide management. Circ Res. 2015;116(6):925–36. doi: 10.1161/CIRCRESAHA.116.304723 25767281

[9] National Institute of Population Research and Training DBM a AD. Bangladesh demographic and health survey 2011. Calverton, Maryland, U.S.A.: ICF International. 2011. https://dhsprogram.com/pubs/pdf/fr265/fr265.pdf

[10] National Institute of Population Research and Training. Bangladesh Demographic and Health Survey 2017-18. 2020. https://dhsprogram.com/pubs/pdf/FR344/FR344.pdf

[11] Razzaque A, Nahar L, Mustafa AHMG, Ahsan KZ, Islam MS, Yunus MJAP. Sociodemographic differentials of selected noncommunicable diseases risk factors among adults in Matlab, Bangladesh: findings from a WHO STEPS survey. J Public Health. 2011;23(2):183–91. 10.1177/1010539510392743 21159696

[12] Tareque MI, Koshio A, Tiedt AD, Hasegawa T. Are the rates of hypertension and diabetes higher in people from lower socioeconomic status in Bangladesh? Results from a nationally representative survey. PLoS One. 2015;10(5):e0127954. doi: 10.1371/journal.pone.0127954 26017066 PMC4446365

[13] Khan MN, Oldroyd JC, Chowdhury EK, Hossain MB, Rana J, Renzetti S, et al. Prevalence, awareness, treatment, and control of hypertension in Bangladesh: Findings from National Demographic and Health Survey, 2017-2018. J Clin Hypertens (Greenwich). 2021;23(10):1830–42. doi: 10.1111/jch.14363 34492733 PMC8678656

[14] Azeez O, Kulkarni A, Kuklina EV, Kim SY, Cox S. Hypertension and Diabetes in Non-Pregnant Women of Reproductive Age in the United States. Prev Chronic Dis. 2019;16:E146. doi: 10.5888/pcd16.190105 31651378 PMC6824149

[15] Tuoyire DA, Ayetey H. GENDER DIFFERENCES IN THE ASSOCIATION BETWEEN MARITAL STATUS AND HYPERTENSION IN GHANA. J Biosoc Sci. 2019;51(3):313–34. doi: 10.1017/S0021932018000147 29781417

[16] Segawa HK, Uematsu H, Dorji N, Wangdi U, Dorjee C, Yangchen P, et al. Gender with marital status, cultural differences, and vulnerability to hypertension: Findings from the national survey for noncommunicable disease risk factors and mental health using WHO STEPS in Bhutan. PLoS One. 2021;16(8):e0256811. doi: 10.1371/journal.pone.0256811 34464428 PMC8407566

[17] Son M, Heo YJ, Hyun H-J, Kwak HJ. Effects of Marital Status and Income on Hypertension: The Korean Genome and Epidemiology Study (KoGES). J Prev Med Public Health. 2022;55(6):506–19. doi: 10.3961/jpmph.22.264 36475316 PMC9742404

[18] Holt-Lunstad J, Birmingham W, Jones BQ. Is there something unique about marriage? The relative impact of marital status, relationship quality, and network social support on ambulatory blood pressure and mental health. Ann Behav Med. 2008;35(2):239–44. doi: 10.1007/s12160-008-9018-y 18347896

[19] Hong T, Xie S, Liu X, Wu J, Chen G. Do Machine Learning Approaches Perform Better Than Regression Models in Mapping Studies? A Systematic Review. Value Health. 2025;28(5):800–11. doi: 10.1016/j.jval.2024.12.010 39922301

[20] Saxena A, Sharma S, Kumar Johari P, Pandey A, Kumar S. A fair and interpretable deep learning approach for healthcare access prediction in underserved communities. Discov Artif Intell. 2025;5(1). doi: 10.1007/s44163-025-00425-3

[21] Shen S, Qi W, Li S, Zeng J, Liu X, Zhu X, et al. Mapping the landscape of machine learning in chronic disease management: A comprehensive bibliometric study. Digit Health. 2025;11:20552076251361614. doi: 10.1177/20552076251361614 40727621 PMC12301648

[22] Dhanda SS, Panwar D, Lin C-C, Sharma TK, Rastogi D, Bindewari S, et al. Advancement in public health through machine learning: a narrative review of opportunities and ethical considerations. J Big Data. 2025;12(1). doi: 10.1186/s40537-025-01201-x

[23] El-Geneedy M, El-Din Moustafa H, Khater H, Abd-Elsamee S, Gamel SA. A comprehensive explainable AI approach for enhancing transparency and interpretability in stroke prediction. Sci Rep. 2025;15(1):26048. doi: 10.1038/s41598-025-11263-9 40681594 PMC12274279

[24] Kumar D, Balraj K, Seth S, Vashista S, Ramteke M, Rathore AS. An improved machine learning-based prediction framework for early detection of events in heart failure patients using mHealth. Health Technol. 2024;14(3):495–512. doi: 10.1007/s12553-024-00832-z

[25] Parihar A, Khan R, Kumar A, Kaushik AK, Gohel H. Machine learning and deep learning based AI tools for development of diagnostic tools. Computational approaches for novel therapeutic and diagnostic designing to mitigate SARS-CoV-2 infection. Academic Press. 2022. p. 399–420.

[26] Mancia G, Cappuccio FP, Burnier M, Coca A, Persu A, Borghi C, et al. Perspectives on improving blood pressure control to reduce the clinical and economic burden of hypertension. J Intern Med. 2023;294(3):251–68. doi: 10.1111/joim.13678 37401044

[27] Tsoi K, Yiu K, Lee H, Cheng H-M, Wang T-D, Tay J-C, et al. Applications of artificial intelligence for hypertension management. J Clin Hypertens (Greenwich). 2021;23(3):568–74. doi: 10.1111/jch.14180 33533536 PMC8029548

[28] Chaikijurajai T, Laffin LJ, Tang WHW. Artificial Intelligence and Hypertension: Recent Advances and Future Outlook. American Journal of Hypertension. 2020;33(11):967–74. 32615586 10.1093/ajh/hpaa102 PMC7608522

[29] Silva GFS, Fagundes TP, Teixeira BC, Chiavegatto Filho ADP. Machine Learning for Hypertension Prediction: A Systematic Review. Current Hypertension Reports. 2022;24(11):523–33. 35731335 10.1007/s11906-022-01212-6

[30] Padmanabhan S, Tran TQB, Dominiczak AF. Artificial Intelligence in Hypertension. Circulation Research. 2021;128(7):1100–18. 33793339 10.1161/CIRCRESAHA.121.318106

[31] Hasselström J, Zarrinkoub R, Holmquist C, Hjerpe P, Ljungman C, Qvarnström M, et al. The Swedish Primary Care Cardiovascular Database (SPCCD): 74 751 hypertensive primary care patients. Blood Press. 2014;23(2):116–25. doi: 10.3109/08037051.2013.814829 23914944

[32] Ji W, Zhang Y, Cheng Y, Wang Y, Zhou Y. Development and validation of prediction models for hypertension risks: A cross-sectional study based on 4,287,407 participants. Front Cardiovasc Med. 2022;9:928948. doi: 10.3389/fcvm.2022.928948 36225955 PMC9548597

[33] Tayefi M, Esmaeili H, Saberi Karimian M, Amirabadi Zadeh A, Ebrahimi M, Safarian M, et al. The application of a decision tree to establish the parameters associated with hypertension. Comput Methods Programs Biomed. 2017;139:83–91. doi: 10.1016/j.cmpb.2016.10.020 28187897

[34] Islam MM, Alam MJ, Maniruzzaman M, Ahmed NAMF, Ali MS, Rahman MJ, et al. Predicting the risk of hypertension using machine learning algorithms: A cross sectional study in Ethiopia. PLoS One. 2023;18(8):e0289613. doi: 10.1371/journal.pone.0289613 37616271 PMC10449142

[35] Chowdhury MZI, Leung AA, Walker RL, Sikdar KC, O’Beirne M, Quan H, et al. A comparison of machine learning algorithms and traditional regression-based statistical modeling for predicting hypertension incidence in a Canadian population. Sci Rep. 2023;13(1):13. doi: 10.1038/s41598-022-27264-x 36593280 PMC9807553

[36] Aish M, Ghafoor A, Nasim F, Ali K, Akhter S, Azeem S. Improving stroke prediction accuracy through machine learning and synthetic minority over-sampling. Journal of Computing & Biomedical Informatics. 2024;7.

[37] Schjerven FE, Ingeström EML, Steinsland I, Lindseth F. Development of risk models of incident hypertension using machine learning on the HUNT study data. Sci Rep. 2024;14(1):5609. doi: 10.1038/s41598-024-56170-7 38454041 PMC10920790

[38] Chang W, Liu Y, Xiao Y, Yuan X, Xu X, Zhang S, et al. A Machine-Learning-Based Prediction Method for Hypertension Outcomes Based on Medical Data. Diagnostics (Basel). 2019;9(4):178. doi: 10.3390/diagnostics9040178 31703364 PMC6963807

[39] Du Z, Yang Y, Zheng J, Li Q, Lin D, Li Y, et al. Accurate Prediction of Coronary Heart Disease for Patients With Hypertension From Electronic Health Records With Big Data and Machine-Learning Methods: Model Development and Performance Evaluation. JMIR Med Inform. 2020;8(7):e17257. doi: 10.2196/17257 32628616 PMC7381262

[40] Kourou K, Exarchos TP, Exarchos KP, Karamouzis MV, Fotiadis DI. Machine learning applications in cancer prognosis and prediction. Comput Struct Biotechnol J. 2014;13:8–17. doi: 10.1016/j.csbj.2014.11.005 25750696 PMC4348437

[41] Selya AS, Anshutz D. Machine learning for the classification of obesity from dietary and physical activity patterns. Advanced Data Analytics in Health. 2018. p. 77–97.

[42] Panaretos D, Koloverou E, Dimopoulos AC, Kouli G-M, Vamvakari M, Tzavelas G, et al. A comparison of statistical and machine-learning techniques in evaluating the association between dietary patterns and 10-year cardiometabolic risk (2002-2012): the ATTICA study. Br J Nutr. 2018;120(3):326–34. doi: 10.1017/S0007114518001150 29789037

[43] Premsagar P, Aldous C, Esterhuizen TM, Gomes BJ, Gaskell JW, Tabb DL. Comparing conventional statistical models and machine learning in a small cohort of South African cardiac patients. Informatics in Medicine Unlocked. 2022;34:101103. doi: 10.1016/j.imu.2022.101103

[44] Hu P, Li Y, Liu Y, Guo G, Gao X, Su Z, et al. Comparison of Conventional Logistic Regression and Machine Learning Methods for Predicting Delayed Cerebral Ischemia After Aneurysmal Subarachnoid Hemorrhage: A Multicentric Observational Cohort Study. Front Aging Neurosci. 2022;14:857521. doi: 10.3389/fnagi.2022.857521 35783143 PMC9247265

[45] Jerez JM, Molina I, García-Laencina PJ, Alba E, Ribelles N, Martín M, et al. Missing data imputation using statistical and machine learning methods in a real breast cancer problem. Artif Intell Med. 2010;50(2):105–15. doi: 10.1016/j.artmed.2010.05.002 20638252

[46] Xing Y, Wang J, Zhao Z, Gao A. Combination data mining methods with new medical data to predicting outcome of coronary heart disease. In: Convergence Information Technology, International Conference on, 2007. 868–72.

[47] Ture M, Kurt I, Turhankurum A, Ozdamar K. Comparing classification techniques for predicting essential hypertension. Expert Systems with Applications. 2005;29(3):583–8. doi: 10.1016/j.eswa.2005.04.014

[48] Wu X, Yuan X, Wang W, Liu K, Qin Y, Sun X, et al. Value of a Machine Learning Approach for Predicting Clinical Outcomes in Young Patients With Hypertension. Hypertension. 2020;75(5):1271–8. doi: 10.1161/HYPERTENSIONAHA.119.13404 32172622

[49] Assi S, Jayabalan M, Parakh V, Assi J, Al Hamid A, Obe DA-J. Predicting incidence of stroke via supervised machine learning methods on class imbalanced data. Non-Invasive Health Systems based on Advanced Biomedical Signal and Image Processing. CRC Press. 2024. p. 128–44.

[50] Ijaz M, Alfian G, Syafrudin M, Rhee J. Hybrid Prediction Model for Type 2 Diabetes and Hypertension Using DBSCAN-Based Outlier Detection, Synthetic Minority Over Sampling Technique (SMOTE), and Random Forest. Applied Sciences. 2018;8(8):1325. doi: 10.3390/app8081325

[51] Alsmariy R, Healy G, Abdelhafez H. Predicting Cervical Cancer using Machine Learning Methods. IJACSA. 2020;11(7). doi: 10.14569/ijacsa.2020.0110723

[52] Sihag G, Yadav P, Vijay V, Delcroix V, Siebert X, Yadav SK, et al. Advantages of oversampling techniques: a case study in risk factors for fall prediction. In: 2021.

[53] Muraru MM, Simó Z, Iantovics LB. Cervical cancer prediction based on imbalanced data using machine learning algorithms. Preprints. 2024.

[54] Chai SS, Goh KL, Cheah WL, Chang YHR, Ng GW. Hypertension prediction in adolescents using anthropometric measurements: do machine learning models perform equally well?. Applied Sciences. 2022;12(3):1600.

[55] Shuja M, Mittal S, Zaman M. Effective Prediction of Type II Diabetes Mellitus Using Data Mining Classifiers and SMOTE. 2020:195–211.

[56] Wang K, Tian J, Zheng C, Yang H, Ren J, Li C, et al. Improving Risk Identification of Adverse Outcomes in Chronic Heart Failure Using SMOTE+ENN and Machine Learning. Risk Manag Healthc Policy. 2021;14:2453–63. doi: 10.2147/RMHP.S310295 34149290 PMC8206455

[57] Zheng H, Sherazi SWA, Lee JY. A cost-sensitive deep neural network-based prediction model for the mortality in acute myocardial infarction patients with hypertension on imbalanced data. Front Cardiovasc Med. 2024;11:1276608. doi: 10.3389/fcvm.2024.1276608 38566962 PMC10986180

[58] Matondang N, Surantha N. Effects of oversampling SMOTE in the classification of hypertensive dataset. Adv Sci Technol Eng Syst. 2020;5(4).

[59] Huang X, Cao T, Chen L, Wu H, Li J, Tan Z. Predicting Stroke Risk in a Chinese Hypertensive Population Using Machine Learning. 2021.

[60] Alabdallah A, Pashami S, Rögnvaldsson T, Ohlsson M. SurvSHAP: a proxy-based algorithm for explaining survival models with SHAP. In: 2022.

[61] Cakiroglu C, Demir S, Hakan Ozdemir M, Latif Aylak B, Sariisik G, Abualigah L. Data-driven interpretable ensemble learning methods for the prediction of wind turbine power incorporating SHAP analysis. Expert Systems with Applications. 2024;237:121464. doi: 10.1016/j.eswa.2023.121464

[62] Luo H, Xiang C, Zeng L, Li S, Mei X, Xiong L, et al. SHAP based predictive modeling for 1 year all-cause readmission risk in elderly heart failure patients: feature selection and model interpretation. Scientific Reports. 2024;14(1):17728. 39085442 10.1038/s41598-024-67844-7 PMC11291677

[63] Xiaomao X, Xudong Z, Yuanfang W. A Comparison of Feature Selection Methodology for Solving Classification Problems in Finance. J Phys: Conf Ser. 2019;1284(1):012026. doi: 10.1088/1742-6596/1284/1/012026

[64] Raufi B, Longo L. Comparing ANOVA and PowerShap Feature Selection Methods via Shapley Additive Explanations of Models of Mental Workload Built with the Theta and Alpha EEG Band Ratios. BioMedInformatics. 2024;4(1):853–76. doi: 10.3390/biomedinformatics4010048

[65] Ejiyi CJ, Qin Z, Ukwuoma CC, Nneji GU, Monday HN, Ejiyi MB. Comparative performance analysis of Boruta, SHAP, and Borutashap for disease diagnosis: A study with multiple machine learning algorithms. Network: Computation in Neural Systems. :1–38. 10.1080/0954898X.2024.2331506 38511557

[66] Asadi R, Khattak A, Vashani H, Almujibah HR, Rabie H, Asadi S, et al. Self-Paced Ensemble-SHAP Approach for the Classification and Interpretation of Crash Severity in Work Zone Areas. Sustainability. 2023;15(11):9076. doi: 10.3390/su15119076

[67] Tran VQ, Byeon H. Predicting dementia in Parkinson’s disease on a small tabular dataset using hybrid LightGBM-TabPFN and SHAP. Digit Health. 2024;10:20552076241272585. doi: 10.1177/20552076241272585 39968191 PMC11833816

[68] Amin P. Feature importance in predicting clinical outcome: statistics vs. explainable artificial intelligence. bioRxiv. 2024;2024:21.604467.

[69] Lee Y, Kim K, Seo J. CLE-SH: Comprehensive Literal Explanation package for SHapley values by statistical validity. arXiv preprint. 2024. doi: arXiv:240912578

[70] Asadullah Md, Hossain MdM, Rahaman S, Amin MS, Sumy MstSA, Parh MdYA, et al. Evaluation of machine learning techniques for hypertension risk prediction based on medical data in Bangladesh. IJEECS. 2023;31(3):1794. doi: 10.11591/ijeecs.v31.i3.pp1794-1802

[71] Islam M, Alam J, Kumar S, Islam A, Khan MR, Rabby S. Development and validation of a nomogram model for predicting the risk of hypertension in Bangladesh. Heliyon. 2024;10(22). 10.1016/j.heliyon.2024.e40246 PMC11600071 39605842

[72] Islam MM, Rahman MJ, Chandra Roy D, Tawabunnahar M, Jahan R, Ahmed NAMF, et al. Machine learning algorithm for characterizing risks of hypertension, at an early stage in Bangladesh. Diabetes Metab Syndr. 2021;15(3):877–84. doi: 10.1016/j.dsx.2021.03.035 33892404

[73] Islam SMS, Talukder A, Awal MA, Siddiqui MMU, Ahamad MM, Ahammed B, et al. Machine Learning Approaches for Predicting Hypertension and Its Associated Factors Using Population-Level Data From Three South Asian Countries. Front Cardiovasc Med. 2022;9:839379. doi: 10.3389/fcvm.2022.839379 35433854 PMC9008259

[74] Parvin S, Akter S, Hossain MI, Ali MS, Soni MSM. Residential variations in hypertension prevalence and trends among adults in Bangladesh. Res Health Serv Reg. 2024;3(1):3. doi: 10.1007/s43999-024-00040-2 39177903 PMC11281750

[75] Siddiquee T. Machine learning approaches for estimating prevalence of undiagnosed hypertension among Bangladeshi adults: evidence from a nationwide survey. Journal of Hypertension. 2023;75:1–10.

[76] Ghosh PK, Islam MA, Haque MA, Tariqujjaman M, Das NC, Ali M, et al. Identifying predictors and assessing causal effect on hypertension risk among adults using Double Machine Learning models: Insights from Bangladesh Demographic and Health Survey. PLoS Comput Biol. 2025;21(7):e1013211. doi: 10.1371/journal.pcbi.1013211 40601770 PMC12338952

[77] Silvey S, Liu J. Sample Size Requirements for Popular Classification Algorithms in Tabular Clinical Data: Empirical Study. J Med Internet Res. 2024;26:e60231. doi: 10.2196/60231 39689306 PMC11688588

[78] Baeza-Delgado C, Cerdá Alberich L, Carot-Sierra JM, Veiga-Canuto D, Martínez de Las Heras B, Raza B, et al. A practical solution to estimate the sample size required for clinical prediction models generated from observational research on data. Eur Radiol Exp. 2022;6(1):22. doi: 10.1186/s41747-022-00276-y 35641659 PMC9156610

[79] Infante G, Miceli R, Ambrogi F. Sample size and predictive performance of machine learning methods with survival data: A simulation study. Stat Med. 2023;42(30):5657–75. doi: 10.1002/sim.9931 37947168

[80] Al Kibria GM, Burrowes V, Choudhury A, Sharmeen A, Swasey K. Sex differences in prevalence and associated factors of prehypertension and hypertension among Bangladeshi adults. Int J Cardiol Hypertens. 2019;1:100006. doi: 10.1016/j.ijchy.2019.100006 33447740 PMC7803050

[81] Chowdhury MAB, Islam M, Rahman J, Uddin MT, Haque MR, Uddin MJJ. Changes in prevalence and risk factors of hypertension among adults in Bangladesh: An analysis of two waves of nationally representative surveys. PLOS ONE. 2021;16(12):e0259507. 10.1371/journal.pone.0259507 PMC8638884 34855768

[82] Hossain M, Khan M, Ababneh F, Shaw JJ. Identifying factors influencing contraceptive use in Bangladesh: evidence from BDHS 2014 data. BMC Public Health. 2018;18(1):1–14. 10.1186/s12889-018-5098-1 PMC5789662 29378546

[83] Khan MN, Islam MM, Islam RM. Association Between Contraception Use, Diabetes and Hypertension: Findings from Bangladesh Demographic and Health Survey. 2022. 10.1186/s12905-022-01822-x PMC9202138 35705977

[84] Hossain MB, Khan MN, Oldroyd JC, Rana J, Magliago DJ, Chowdhury EK, et al. Prevalence of, and risk factors for, diabetes and prediabetes in Bangladesh: Evidence from the national survey using a multilevel Poisson regression model with a robust variance. PLOS Glob Public Health. 2022;2(6):e0000461. doi: 10.1371/journal.pgph.0000461 36962350 PMC10021925

[85] Islam R, Khan M, Oldroyd J, Rana J, Chowdhury E, Karim M, et al. Prevalence of diabetes and prediabetes among Bangladeshi adults and associated factors: Evidence from the Demographic and Health Survey, 2017-18. 2021.

[86] Organization WH. Definition and diagnosis of diabetes mellitus and intermediate hyperglycaemia: report of a WHO/IDF consultation. 2006.

[87] Zong X, Wang H, Yang L, Guo Y, Zhao M, Magnussen CG, et al. Maternal Pre-pregnancy Body Mass Index Categories and Infant Birth Outcomes: A Population-Based Study of 9 Million Mother-Infant Pairs. Front Nutr. 2022;9:789833. doi: 10.3389/fnut.2022.789833 35252291 PMC8891137

[88] Lemoine A, Tounian P. Childhood anemia and iron deficiency in sub-Saharan Africa - risk factors and prevention: A review. Arch Pediatr. 2020;27(8):490–6. doi: 10.1016/j.arcped.2020.08.004 32950332

[89] Aksoy S, Haralick RM. Feature normalization and likelihood-based similarity measures for image retrieval. Pattern Recognition Letters. 2001;22(5):563–82. doi: 10.1016/s0167-8655(00)00112-4

[90] Byeon HJI. Development of a physical impairment prediction model for Korean elderly people using synthetic minority over-sampling technique and XGBoost. JoACS. 2021;12(1).

[91] Byeon HJI. Predicting the depression of the South Korean elderly using SMOTE and an imbalanced binary dataset. JoACS, Applications. 2021;12(1).

[92] Hanafy M, Ming RJ. Improving imbalanced data classification in auto insurance by the data level approaches. J Appl Comput Sci. 2021;12(6).

[93] Geurts P, Ernst D, Wehenkel L. Extremely randomized trees. Mach Learn. 2006;63(1):3–42. doi: 10.1007/s10994-006-6226-1

[94] Biau G, Scornet E. A random forest guided tour. TEST. 2016;25(2):197–227. doi: 10.1007/s11749-016-0481-7

[95] Touw WG, Bayjanov JR, Overmars L, Backus L, Boekhorst J, Wels M, et al. Data mining in the Life Sciences with Random Forest: a walk in the park or lost in the jungle?. Brief Bioinform. 2013;14(3):315–26. doi: 10.1093/bib/bbs034 22786785 PMC3659301

[96] Bergstra J, Bengio YJ. Random search for hyper-parameter optimization. JMLR. 2012;13(2).

[97] Sifat IK, Kibria MK. Optimizing hypertension prediction using ensemble learning approaches. PLoS One. 2024;19(12):e0315865. doi: 10.1371/journal.pone.0315865 39715219 PMC11666061

[98] Sanaudi R, Zakaria ZA, Khairulisam AA, Ibrahim N, Ul Saufie AZ. Unveil the Features Influencing Hypertension Adults in Malaysia Using Machine Learning Models. Malaysian Journal of Medicine & Health Sciences. 2024;20(6).

[99] Seo J-W, Lee S, Yim MH. Machine Learning Approach for Predicting Hypertension Based on Body Composition in South Korean Adults. Bioengineering (Basel). 2024;11(9):921. doi: 10.3390/bioengineering11090921 39329663 PMC11428396

[100] Tanaka M, Akiyama Y, Mori K, Hosaka I, Endo K, Ogawa T, et al. Machine learning-based analyses of contributing factors for the development of hypertension: a comparative study. Clin Exp Hypertens. 2025;47(1):2449613. doi: 10.1080/10641963.2025.2449613 39773295

[101] Angelaki E, Barmparis GD, Fragkiadakis K, Maragkoudakis S, Zacharis E, Plevritaki A, et al. Diagnostic performance of single-lead electrocardiograms for arterial hypertension diagnosis: a machine learning approach. J Hum Hypertens. 2025;39(1):58–65. doi: 10.1038/s41371-024-00969-4 39424986

[102] Leha A, Hellenkamp K, Unsöld B, Mushemi-Blake S, Shah AM, Hasenfuß G, et al. A machine learning approach for the prediction of pulmonary hypertension. PLoS One. 2019;14(10):e0224453. doi: 10.1371/journal.pone.0224453 31652290 PMC6814224

[103] Donmez TB, Kutlu M. Explainable quantum-enhanced machine learning for hypertension prediction. Eur Phys J Spec Top. 2025. doi: 10.1140/epjs/s11734-025-01629-5

[104] Saito T, Rehmsmeier M. The precision-recall plot is more informative than the ROC plot when evaluating binary classifiers on imbalanced datasets. PLoS One. 2015;10(3):e0118432. doi: 10.1371/journal.pone.0118432 25738806 PMC4349800

[105] Akosa JS. Predictive accuracy: a misleading performance measure for highly imbalanced data. 2017.

[106] Alam TM, Shaukat K, Hameed IA, Luo S, Sarwar MU, Shabbir S, et al. An Investigation of Credit Card Default Prediction in the Imbalanced Datasets. IEEE Access. 2020;8:201173–98. doi: 10.1109/access.2020.3033784

[107] Delgado R, Tibau X-A. Why Cohen’s Kappa should be avoided as performance measure in classification. PLoS One. 2019;14(9):e0222916. doi: 10.1371/journal.pone.0222916 31557204 PMC6762152

[108] Jeni LA, Cohn JF, De La Torre F. Facing imbalanced data--recommendations for the use of performance metrics. In: 2013. 10.1109/ACII.2013.47 PMC4285355 25574450

[109] Fitriyani NL, Syafrudin M, Alfian G, Yang C k, Rhee J, Ulyah SM. Chronic Disease Prediction Model Using Integration of DBSCAN, SMOTE-ENN, and Random Forest. In: 22-23 June 2022, 2022.

[110] Muntasir Nishat M, Faisal F, Jahan Ratul I, Al-Monsur A, Ar-Rafi AM, Nasrullah SM, et al. A Comprehensive Investigation of the Performances of Different Machine Learning Classifiers with SMOTE-ENN Oversampling Technique and Hyperparameter Optimization for Imbalanced Heart Failure Dataset. Scientific Programming. 2022;2022:1–17. doi: 10.1155/2022/3649406

[111] Parthasarathy S, Jayaraman V, R JPP. Predicting heart failure using SMOTE-ENN-XGBoost. In: 2023.

[112] Tang J, Wang X, Wan H, Lin C, Shao Z, Chang Y, et al. Joint modeling strategy for using electronic medical records data to build machine learning models: an example of intracerebral hemorrhage. BMC Med Inform Decis Mak. 2022;22(1):278. doi: 10.1186/s12911-022-02018-x 36284327 PMC9594939

[113] Ullah Z, Saleem F, Jamjoom M, Fakieh B, Kateb F, Ali AM, et al. Detecting High-Risk Factors and Early Diagnosis of Diabetes Using Machine Learning Methods. Comput Intell Neurosci. 2022;2022:2557795. doi: 10.1155/2022/2557795 36210985 PMC9536939

[114] Ahammed B, Maniruzzaman M, Talukder A, Ferdausi F. Prevalence and Risk Factors of Hypertension Among Young Adults in Albania. High Blood Press Cardiovasc Prev. 2021;28(1):35–48. doi: 10.1007/s40292-020-00419-5 33113094

[115] Weare AR, Feng Z, McGrath N. The prevalence of hypertension and hypertension control among married Namibian couples. PLoS One. 2023;18(8):e0289788. doi: 10.1371/journal.pone.0289788 37561676 PMC10414666

[116] Khan MN, Islam MM, Islam RM. Pattern of contraceptive use among reproductive-aged women with diabetes and/or hypertension: findings from Bangladesh Demographic and Health Survey. BMC Womens Health. 2022;22(1):230. doi: 10.1186/s12905-022-01822-x 35705977 PMC9202138

[117] Bista B, Dhungana RR, Chalise B, Pandey AR. Prevalence and determinants of non-communicable diseases risk factors among reproductive aged women of Nepal: Results from Nepal Demographic Health Survey 2016. PLoS One. 2020;15(3):e0218840. doi: 10.1371/journal.pone.0218840 32176883 PMC7075700

[118] Morvaridi M, Rayyani E, Jaafari M, Khiabani A, Rahimlou M. The effect of green coffee extract supplementation on cardio metabolic risk factors: a systematic review and meta-analysis of randomized controlled trials. J Diabetes Metab Disord. 2020;19(1):645–60. doi: 10.1007/s40200-020-00536-x 32550217 PMC7271291

[119] Rahimlou M, Grau N, Banaie-Jahromi N, Taheri M, Khosravi A, Mavrommatis Y, et al. Association of adherence to the dietary approach to stop hypertension and Mediterranean diets with blood pressure in a non-hypertensive population: Results from Isfahan Salt Study (ISS). Nutr Metab Cardiovasc Dis. 2022;32(1):109–16. doi: 10.1016/j.numecd.2021.09.029 34893410

