# Correction: Predicting hypertension and identifying most important factors among married women in Bangladesh using machine learning approach

**DOI:** 10.1371/journal.pone.0342021

**Published:** 2026-01-29

**Authors:** Novel Chandra Das, Probir Kumar Ghosh, Md. Alamgir Hossain, Uddip Acharjee Shuvo, Nipa Rani Talukder, Fatema Khatun, Mohammad Ziaul Islam Chowdhury

The Introduction heading has been misspelled. The correct spelling is Introduction.
