# Supplementary material for: Predicting hypertension and identifying most important factors among married women in Bangladesh using machine learning approach
Source: PLoS One. 2025 Oct 30;20(10):e0335442. doi: 10.1371/journal.pone.0335442 (PMC12574887; doi:10.1371/journal.pone.0335442)
Supplement: S1 Appendix — (DOCX) [file pone.0335442.s001.docx]

**S1 Appendix: List of class balanced techniques**

**Synthetic Minority Oversampling Techniques (SMOTE):**

The resampling technique, which is proposed (1) to improve the classification of minority classes in imbalanced data. This approach considers samples of the feature space for each target class and its nearest neighbors, then new sample are produced by combining features of the target case with features of its neighbor. The new instances are not copying of existing minority samples.

**Adaptive Synthetic Sampling (ADASYN):**

ADASYN is proposed(2) to use a weighted distribution for different minority class cases according to their level of difficulty in learning. With more artificial cases generated for minority class cases that are harder to learn than minority cases that are easier to learn.

**Tomek Links (TLs):**

The approach demonstrates a link between the two nearest neighbors from opposite class while each case is nearest to other. Then Tomek Links approach looks for such link and removes the majority cases of the link(3) (4)

**Edited Nearest Neighbor (ENN):**

The ENN approach is developed by Wilson (5), where each cases is tested applying k-NN with the rest of the sample in this method. The observation and its k-NN are removed from the dataset if there is difference between observation’s k-NN’s majority class and observation’s class.

**SMOTE-TomekLinks:**

The approach is combination of over-sampling and under-sampling where SMOTE represent over-sampling technique and TomekLinks represent Under-sampling technique(3).

**SMOTE-ENN:**

The approach is combination of over-sampling and under-sampling where SMOTE represent over-sampling technique and Edited Nearest Neighbor (ENN) represent Under-sampling technique (3).

Reference

1. Chawla NV, Bowyer KW, Hall LO, Kegelmeyer WP. SMOTE: synthetic minority over-sampling technique. Journal of artificial intelligence research. 2002;16:321-57.

2. He H, Bai Y, Garcia EA, Li S, editors. ADASYN: Adaptive synthetic sampling approach for imbalanced learning. 2008 IEEE international joint conference on neural networks (IEEE world congress on computational intelligence); 2008: Ieee.

3. Batista GE, Prati RC, Monard MCJASen. A study of the behavior of several methods for balancing machine learning training data. 2004;6(1):20-9.

4. Tomek I. Two modifications of CNN. 1976.

5. Wilson DLJIToS, Man,, Cybernetics. Asymptotic properties of nearest neighbor rules using edited data. 1972(3):408-21.
