# Supplementary material for: Predicting hypertension and identifying most important factors among married women in Bangladesh using machine learning approach
Source: PLoS One. 2025 Oct 30;20(10):e0335442. doi: 10.1371/journal.pone.0335442 (PMC12574887; doi:10.1371/journal.pone.0335442)
Supplement: S2 Appendix — (DOCX) [file pone.0335442.s002.docx]

**S2 Appendix: Evaluation of machine learning algorithms**

1. Logistic Regression

Logistic Regression is a supervised learning algorithm used for binary classification tasks. It predicts the probability that a given input belongs to a particular category using the logistic function, which maps predicted values to a range between 0 and 1. The model is optimized by minimizing a loss function such as cross-entropy loss.

2. Decision Trees

Decision Trees are non-parametric models that split the dataset into subsets based on feature values. Each node represents a feature, and branches denote decision rules, leading to leaf nodes that represent outcomes. The tree is built using metrics like Gini Impurity or Information Gain to ensure optimal splits.

3. K-Nearest Neighbors (KNN)

KNN is a non-parametric, instance-based learning algorithm. For a given input, it identifies the k nearest data points in the feature space and assigns the most common class label among them. Distance metrics such as Euclidean, Manhattan, or Minkowski distance are typically used.

4. Random Forest

Random Forest is an ensemble method based on Decision Trees. It builds multiple trees during training and combines their outputs through majority voting (for classification) or averaging (for regression). Randomness is introduced by using bootstrap samples and random feature selection, improving generalization.

5. Extra Trees (Extremely Randomized Trees)

Extra Trees are similar to Random Forest but differ in their construction. Instead of optimizing split points, Extra Trees choose splits at random. This increases diversity in the ensemble and can lead to faster training while reducing overfitting.

6. AdaBoost (Adaptive Boosting)

AdaBoost is a boosting algorithm that combines multiple weak learners, typically Decision Trees, to create a strong learner. It assigns higher weights to misclassified samples, focusing on harder examples in subsequent iterations. The final prediction is a weighted sum of the individual learners.

7. XGBoost (Extreme Gradient Boosting)

XGBoost is an advanced gradient boosting algorithm designed for speed and performance. It improves upon traditional Gradient Boosting by incorporating regularization (L1 and L2) to prevent overfitting, parallel processing, and efficient handling of missing values.

8. CatBoost

CatBoost is a gradient boosting algorithm specifically optimized for categorical features. It eliminates the need for extensive preprocessing of categorical variables by employing techniques like Ordered Boosting and target statistics, reducing the risk of overfitting and bias.

9. Gradient Boosting Machine (GBM)

GBM builds an additive model in a forward stage-wise manner. It optimizes a differentiable loss function by combining weak learners, typically Decision Trees. Each subsequent tree corrects errors from the previous ones, iteratively improving performance.

10. LightGBM

LightGBM is a gradient boosting framework that uses a histogram-based approach for faster training and reduced memory usage. It supports leaf-wise tree growth, which can result in deeper trees and better accuracy for some datasets. LightGBM is well-suited for large datasets.

11. Neural Networks (Multilayer Perceptron - MLP)

MLP is a class of feedforward neural networks consisting of multiple layers of neurons. Each neuron applies a weighted sum of its inputs followed by an activation function (e.g., ReLU, Sigmoid, Tanh). MLP is trained using backpropagation and gradient descent to minimize the loss function.

12. Support Vector Machines (SVM)

SVM is a supervised learning algorithm used for classification and regression tasks. It finds the hyperplane that maximizes the margin between data points of different classes. For non-linearly separable data, SVM employs kernel functions (e.g., RBF, Polynomial) to project data into higher dimensions.
