# Supplementary material for: Predicting hypertension and identifying most important factors among married women in Bangladesh using machine learning approach
Source: PLoS One. 2025 Oct 30;20(10):e0335442. doi: 10.1371/journal.pone.0335442 (PMC12574887; doi:10.1371/journal.pone.0335442)
Supplement: S3 Appendix — (DOCX) [file pone.0335442.s003.docx]

**S3 Appendix: Evaluation of machine learning algorithms**

**Confusion matrix:** A confusion matrix or an error matrix describes the performance of a classification model on a set of test data for which the true values are known where output can be two or more classes.

| **Actual** | **Predicted** | | |
| --- | --- | --- | --- |
|  |  | **Yes** | **No** |
|  | **Yes** | **TP** | **FN** |
|  | **No** | **FP** | **TN** |

**True positives (TP) –** Positive predictions are actually positive.

**True negatives (TN) –** Negative predictions are actually negative.

**False positives (FP)–** Positive predictions are actually negative which is also known as Type I error.

**False negatives (FN)–** Negative predictions are actually positive which is also known as Type II error.

**Table : Evaluation metric scores.**

| Indicator | Formula | Description |
| --- | --- | --- |
| Matthews correlation coefficient | Matthews correlation coefficient=$\frac{\left( TP\times TN \right)-(FP\times FN)}{\sqrt{(TP+FP)\times(TP+FN)\times(TN+FP)\times(TN+FN)}}$ | It measures the differences between actual values and predicted values and is equivalent to the chi-square statistics. |
| Cohens-kappa | Cohens-kappa=$\frac{2\times\left( TP\times TN \right)-(FN\times FP)}{\left( TP+FP \right)\times\left( TN+FP \right)+(TP+FN)\times(FN+TN)}$ | The score measures the model exceeded random predictions in terms of accuracy. |
| F1-score | f1-score=$\frac{2\times Precesion\times Recall}{Precesion+Recall}$ | Representation of the harmonic mean between precision and recall. |
| G-mean | G-mean=$\sqrt{Recall\times Specificity}$ | Simultaneously, TP rate and TN rate are kept relatively balanced, after maximizing both rates. |
| Recall/True positive rate (TPR) | Recall/True positive rate (TPR)=$\frac{TP}{TP+FN}$ | The metric used to measure the ratio of positive patterns that are correctly classified. |
| False positive rate (FPR) | False positive rate (FPR)=$\frac{FP}{FP+TN}$ | It measures the ratio of type I error that are correctly classified. |
| Specificity | Specificity=$\frac{TN}{TN+FP}$ | The metric used to measure the ratio of negative patterns that are correctly classified. |
| Precision | Precision=$\frac{TP}{TP+FP}$ | The score is used to evaluate the positive patterns that are correctly predicted from the total predicted patterns in a positive class. |
| Accuracy | Accuracy=$\frac{TP+TN}{TP+FP+TN+FN}$ | It measures the proportion of correct predictions over the total number of cases estimated. |

**AUC-ROC**: Area under ROC curve is evaluation matrix to measure the performance of a classification where receiver operating characteristics (ROC) curve plot two parameters true positive rate (TPR) and false positive rate (FPR).

**AUC-PR**: Area under the precision recall curve (AUC-PR) provide the score of classification model where precision-recall (PR) curve plot two parameters precision and recall.

**Statistical test**

As there is a possibility to find the score from the evaluation metric may be misleading that’s why it is important, using statistical significance test to determine optimal model or technique. To handle the issue, the following statistical test were considered for the study,

**Anderson-Darling test**: To check the dataset is normally distributed or not, we used Anderson-Darling test. Where, Null hypothesis defined that data follow normal distribution. And, the null hypothesis was rejected if the p-value is less than 0.05.

**One-way repeated measure ANOVA:** One-way repeated measure ANOVA is utilized to determine the overall difference among class balanced techniques.

**Tukey’s HSD test**: Tukey’s HSD test is used to classify the significance difference among specific group of class balanced techniques.

**Friedman test**: A non-parametric test are used to find the difference among group. The test is used instead of one-way ANOVA where normality assumptions are violated.

The Friedman test rank each classifier’s data for each imbalanced technique, then consider the ranks value(1).

Reference

1. Friedman M. A comparison of alternative tests of significance for the problem of m rankings. The annals of mathematical statistics. 1940;11(1):86-92.
