# Supplementary material for: Predicting hypertension and identifying most important factors among married women in Bangladesh using machine learning approach
Source: PLoS One. 2025 Oct 30;20(10):e0335442. doi: 10.1371/journal.pone.0335442 (PMC12574887; doi:10.1371/journal.pone.0335442)
Supplement: S4 Appendix — (DOCX) [file pone.0335442.s004.docx]

**Supplementary Tables**

# Contents

• Supplementary Table S1. Class distributions before/after resampling

• Supplementary Table S2. Statistical testing across metrics and samplers (Table S2A–S2E)

• Supplementary Table S3. Validation, calibration, and threshold (Table S3A–S3B)

• Supplementary Table S4. Sample flow and class balance (Table S4A–S4C)

• Supplementary Table S5. Correlations among key predictors

• Supplementary Table S6. Stratified subgroup performance

**Supplementary Table S1. Class distributions in the training set before and after resampling.**

| **Method** | **Normotensive (%)** | **Hypertension (%)** | **Total** |
| --- | --- | --- | --- |
| Original | 2,754 (76.9) | 827 (23.1) | 3,581 |
| SMOTE | 2,754 (50.0) | 2,754 (50.0) | 5,508 |
| ADASYN | 2,754 (50.0) | 2,754 (50.0) | 5,508 |
| Tomek Links | 2,523 (75.3) | 827 (24.7) | 3,350 |
| ENN | 1,533 (65.0) | 827 (35.0) | 2,360 |
| SMOTE + Tomek | 2,721 (50.0) | 2,721 (50.0) | 5,442 |
| SMOTE + ENN | 1,281 (36.7) | 2,210 (63.3) | 3,491 |

**Supplementary Table S2. Statistical testing across metrics and samplers.**

Table S2A. Anderson–Darling normality tests for each metric.

| Metric | Test Statistic | p-value |
| --- | --- | --- |
| Matthews Correlation Coefficient | 3.1817 | <0.05 |
| Cohen’s kappa | 2.4338 | <0.05 |
| AUCPR | 0.31492 | 0.5374 |
| Accuracy | 3.1183 | <0.05 |
| G-mean | 2.3604 | <0.05 |
| Specificity | 1.9971 | <0.05 |
| Recall | 1.4823 | <0.05 |
| Precision | 2.3018 | <0.05 |
| F1 score | 2.7472 | <0.05 |
| AUCROC | 6.8793 | < 0.05 |

Table S2B. One-way repeated-measures ANOVA on AUCPR.

|  | DF | Sum of square | Mean square | F value | P value |
| --- | --- | --- | --- | --- | --- |
| AUCPR | 6 | 0.08431 | 0.014052 | 9.532 | <0.05 |

Table S2C. Tukey’s HSD pairwise comparisons for AUCPR.

|  | Mean difference | 95% Confidence interval | |  |
| --- | --- | --- | --- | --- |
|  |  | Lower bound | Upper bound | P-value |
| Actual - (SMOTE+ENN) | -0.06974 | -0.11741 | -0.022067 | 0.0006 |
| ADASYN- ENN | -0.04852 | -0.09619 | -0.000851 | 0.0434 |
| ADASYN - (SMOTE+ENN) | -0.08606 | -0.13373 | -0.038395 | <.0001 |
| ENN - SMOTE | 0.05240 | 0.00473 | 0.100066 | 0.0221 |
| ENN - (SMOTE+Tomek) | 0.05374 | 0.00607 | 0.101405 | 0.0173 |
| SMOTE - (SMOTE+ENN) | -0.08994 | -0.13761 | -0.042272 | <.0001 |
| (SMOTE+ENN) - (SMOTE+Tomek) | 0.09128 | 0.04361 | 0.138949 | <.0001 |
| (SMOTE+ENN) - Tomek | 0.08139 | 0.03372 | 0.129057 | <.0001 |

Table S2D. Friedman omnibus tests across metrics.

| Friedman tests | Chi-squared score | DF | p-value |
| --- | --- | --- | --- |
| Matthews Correlation Coefficient | 4.8929 | 6 | 0.5576 |
| Cohen’s kappa | 7 | 6 | 0.3208 |
| AUCPR | 31.179 | 6 | <0.05 |
| Accuracy | 52.031 | 6 | <0.05 |
| G-mean | 39.78 | 6 | <0.05 |
| Recall | 46.929 | 6 | <0.05 |
| F1 score | 36.75 | 6 | <0.05 |
| AUCROC | 25.036 | 6 | <0.05 |
| Specificity | 52.831 | 6 | <0.05 |
| Precision | 43.964 | 6 | <0.05 |

Table S2E. Median performance and rank sums by resampling technique (Friedman post-hoc summary).

|  | RANK | RESAMPLING TECHNIQUES | SUM OF RANKS | MEDIAN |
| --- | --- | --- | --- | --- |
| Accuracy | 1 | TomekLinks | 66 | 0.775 |
|  | 2 | SMOTE | 47.5 | 0.75 |
|  | 3 | SMOTE+ TomekLinks | 45.5 | 0.73 |
|  | 4 | ADASYN | 44.5 | 0.73 |
|  | 5 | ENN | 26.5 | 0.675 |
|  | 6 | SMOTE+ENN | 13.5 | 0.61 |
| G-mean | 1 | SMOTE+ ENN | 60 | 0.64 |
|  | 2 | ENN | 58 | 0.63 |
|  | 3 | SMOTE+ TomekLinks | 42.5 | 0.505 |
|  | 4 | SMOTE | 40.5 | 0.54 |
|  | 5 | ADASYN | 34.5 | 0.53 |
|  | 6 | TomekLinks | 26.5 | 0.445 |
| Recall | 1 | SMOTE+ ENN | 69 | 0.717 |
|  | 2 | ENN | 53 | 0.582 |
|  | 3 | SMOTE | 39 | 0.332 |
|  | 4 | ADASYN | 35 | 0.324 |
|  | 5 | SMOTE+ TomekLinks | 31 | 0.306 |
|  | 6 | TomekLinks | 25 | 0.207 |
| F1 score | 1 | SMOTE+ ENN | 61 | 0.450 |
|  | 2 | ENN | 56 | 0.446 |
|  | 3 | SMOTE | 39 | 0.374 |
|  | 4 | ADASYN | 35 | 0.366 |
|  | 5 | SMOTE+ TomekLinks | 32 | 0.350 |
|  | 6 | TomekLinks | 29 | 0.301 |
| AUROC | 1 | TomekLinks | 57 | 0.705 |
|  | 2 | ENN | 52 | 0.698 |
|  | 3 | SMOTE | 41 | 0.685 |
|  | 4 | SMOTE+ ENN | 40 | 0.696 |
|  | 5 | ADASYN | 33 | 0.699 |
|  | 6 | SMOTE+ TomekLinks | 29 | 0.693 |
| Precision | 1 | TomekLinks | 67 | 0.526 |
|  | 2 | SMOTE | 45 | 0.414 |
|  | 3 | ADASYN | 41 | 0.412 |
|  | 3 | SMOTE+ TomekLinks | 41 | 0.410 |
|  | 5 | ENN | 38 | 0.370 |
|  | 6 | SMOTE+ ENN | 19 | 0.337 |
| Specificity | 1 | TomekLinks | 64.5 | 0.94 |
|  | 2 | SMOTE+ TomekLinks | 47 | 0.825 |
|  | 3 | ADASYN | 46.5 | 0.82 |
|  | 4 | SMOTE | 45 | 0.875 |
|  | 5 | ENN | 30.5 | 0.7 |
|  | 6 | SMOTE+ ENN | 13.5 | 0.575 |

**Supplementary Table S3. Model validation, calibration, and decision threshold.**

Table S3A. Cross-validation and nested-CV results (ExtraTrees).

| **Metric** | **Score / Mean ± SD** |
| --- | --- |
| Training F1-score | 0.9689 |
| Cross-validation F1 (5-fold) | 0.934 ± 0.012 |
| Nested CV outer-fold F1 | 0.965 ± 0.010 |
| Cross-validation F1 (mean ± sd) | 0.9358 ± 0.0113 |
| Test F1-score | 0.9447 |

Table S3B. Calibration metrics and threshold (Youden-optimal = 0.55).

| **Metric** | **Value** |
| --- | --- |
| Brier score | 0.0626 |
| Youden’s J | 0.859 |

**Supplementary Table S4. Sample flow and class balance across stages.**

Table S4A. CONSORT-style analytic sample flow.

| **Stage** | **N** |
| --- | --- |
| Raw rows | 4253 |
| After drop-na | 4253 |
| Train set | 3581 |
| Test set | 672 |

Table S4B. Class prevalence per fold (training set, pre-resampling).

| **Fold** | **n_fold** | **Hypertensive (%)** | **Non-hypertensive (%)** |
| --- | --- | --- | --- |
| 1 | 716 | 165 (23.0) | 551 (77.0) |
| 2 | 716 | 165 (23.0) | 551 (77.0) |
| 3 | 716 | 165 (23.0) | 551 (77.0) |
| 4 | 716 | 166 (23.2) | 550 (76.8) |
| 5 | 717 | 166 (23.2) | 551 (76.8) |

Table S4C. Final training distribution after SMOTE+ENN (post-resampling).

| **Class** | **Frequency (%)** |
| --- | --- |
| Hypertension | 2,210 (63.3) |
| Normotensive | 1,281 (36.7) |

**Supplementary Table S5. Correlations among key predictors.**

| **Predictor 1** | **Predictor 2** | **Spearman ρ** | **Comment** |
| --- | --- | --- | --- |
| Respondent’s age <35 | Husband’s age ≥40 | –0.82 | Strong inverse age dependency |
| No children in last 5 years | One child in last 5 years | –0.89 | Structural birth-history collinearity |
| Respondent’s age <35 | Husband’s age <35 | 0.55 | Positive age-matching correlation |
| BMI Overweight | BMI Normal weight | –0.69 | Within-BMI category correlation |
| Living children ≥2 | Ever born ≥2–3 children | >0.80 | Structural parity–fertility overlap |

**Supplementary Table S6. Stratified subgroup performance (example division vs. overall).**

| **Subgroup** | **Value** | **N** | **Acc** | **F1** | **Sens** | **Spec** | **ROC-AUC** | **PR-AUC** | **Acc gap** | **F1 gap** |
| --- | --- | --- | --- | --- | --- | --- | --- | --- | --- | --- |
| Division | 3 (Rajshahi) | 109 | 0.844 | 0.915 | 0.843 | 1 | 1 | 1 | –0.086 | –0.035 |

Notes: Unless stated otherwise, performance metrics and confusion matrices were computed at the Youden-optimal threshold of 0.55.
