# Supplementary material for: Predicting hypertension and identifying most important factors among married women in Bangladesh using machine learning approach
Source: PLoS One. 2025 Oct 30;20(10):e0335442. doi: 10.1371/journal.pone.0335442 (PMC12574887; doi:10.1371/journal.pone.0335442)
Supplement: S2 Fig — (DOCX) [file pone.0335442.s006.docx]

**Supplementary Figure 2**


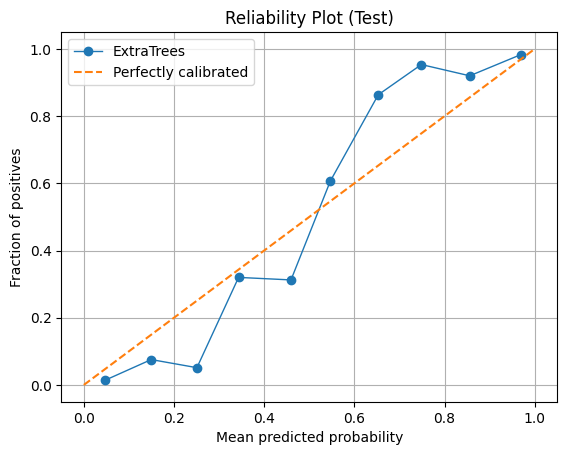


S2 Fig. Reliability plot of the ExtraTrees model for predicting hypertension among married women in Bangladesh.
