# Supplementary material for: Predicting hypertension and identifying most important factors among married women in Bangladesh using machine learning approach
Source: PLoS One. 2025 Oct 30;20(10):e0335442. doi: 10.1371/journal.pone.0335442 (PMC12574887; doi:10.1371/journal.pone.0335442)
Supplement: Attachment — Submitted filename: PONE-D-25-05724_R2.pdf [file pone.0335442.s012.pdf]

PLOS ONE

Predicting Hypertension and Identifying most important Factors among Married Women in Bangladesh using Machine Learning Approach.

--Manuscript Draft--

**Manuscript Number:**	PONE-D-25-05724R2
**Article Type:**	Research Article
**Full Title:**	Predicting Hypertension and Identifying most important Factors among Married Women in Bangladesh using Machine Learning Approach.
**Short Title:**	Machine learning for hypertension predictive factors among Bangladeshi women
**Corresponding Author:**	Novel Chandra Das<br>ICDDRB: International Centre for Diarrhoeal Disease Research Bangladesh<br>Dhaka, BANGLADESH
**Keywords:**	Bangladesh; BDHS; Hypertension; machine learning; Tukey's HSD test; Friedman test; class balanced techniques; SHAP
**Abstract:**	Introduction: Hypertension is a leading contributor to maternal and cardiometabolic morbidity in Bangladesh. We developed and interpreted machine-learning (ML) models to predict hypertension and rank associated factors among married women with the goal of informing targeted screening and policy in low-resource settings.<br>Methods: We analyzed 4,253 married women from the nationally representative BDHS 2017–18 survey (hypertension prevalence: 23.1%). Twelve ML algorithms were trained under six class-balancing strategies with hyperparameters tuned via random search. Validation used a hold-out test set (80/20) and repeated stratified k-fold cross-validation; bootstrap confidence intervals were estimated for the selected model. Model performance was compared with parametric and non-parametric tests. To interpret results, SHAP was used to rank the top 20 predictors and visualize feature effects. Models quantify associations rather than causation.<br>Results: The Extra Trees classifier with SMOTE+ENN achieved the best discrimination (F1 = 0.94; AUC-PR = 0.95; ROC-AUC = 0.95). Compared with the original imbalanced training, minority-class detection improved substantially (Extra Trees F1 increased from 0.08 to 0.94; recall from 0.04 to 0.95) while accuracy and ROC-AUC remained relatively stable across samplers. Statistical testing favored SMOTE+ENN for recall, F1, G-mean and AUC-PR. SHAP identified age, parity, recent births, contraceptive use, spousal education and BMI as key predictors. Younger age (<35 years) and normal/underweight status were protective, while parity ≥2–3, husbands' age ≥40 years and overweight/obesity increased risk.<br>Conclusions: An interpretable ensemble model built primarily on sociodemographic and behavioral variables supplemented by limited biometric markers (BMI, glucose) can accurately flag hypertensive risk among married women in Bangladesh. Findings support programmatic integration of risk scores into eRegistries, routine blood pressure checks in family planning and postpartum visits, husband-focused education/SMS interventions and prioritization of high-parity households in high-risk regions. External validation on BDHS-2022 is planned to assess generalizability.
**Order of Authors:**	Novel Chandra Das
	Probir Kumar Ghosh
	Md. Alamgir Hossain
	Uddip Acharjee Shuvo
	Nipa Rani Talukder
	Fatema Khatun
	Mohammad Ziaul Islam Chowdhury
**Response to Reviewers:**	The Editor,<br>We are grateful for the thoughtful and constructive comments provided on our manuscript. We have carefully addressed each point raised and made the necessary revisions to improve the clarity, rigor, and reproducibility of our work. Below, we provide

a detailed, point-by-point response to each of the comments.

Academic Editor comments
Comments: Please include the following items when submitting your revised manuscript:
•A rebuttal letter that responds to each point raised by the academic editor and reviewer(s). You should upload this letter as a separate file labeled 'Response to Reviewers'.
•A marked-up copy of your manuscript that highlights changes made to the original version. You should upload this as a separate file labeled 'Revised Manuscript with Track Changes'.
•An unmarked version of your revised paper without tracked changes. You should upload this as a separate file labeled 'Manuscript'.
"Response: Thank you for the opportunity to revise our manuscript. In accordance with your instructions, we have submitted the following materials:
Response to Reviewers – A detailed rebuttal letter that addresses each point raised by the academic editor and reviewers.
Revised Manuscript with Track Changes – A marked-up version of the manuscript highlighting all revisions made in response to the reviewers' comments.
Manuscript – A clean, unmarked version of the revised manuscript without tracked changes."

Reviewers' comments:

Reviewer #4: Detailed Comments
Comment: Title and Abstract
The title is clear and descriptive, effectively capturing the dual focus on evaluating ML algorithms and identifying hypertension factors. It could perhaps be tightened for conciseness, something like "Machine Learning Approaches to Predict and Rank Hypertension Risk Factors Among Married Women in Bangladesh" – but that's minor. The abstract is generally well-structured, summarizing the introduction, methods, results, and conclusion. I like how it highlights key metrics (e.g., F1 score jumping from 8% to 94% with SMOTE+ENN) and the top positive/negative factors from SHAP. However, there are a few awkward phrasings that make it feel a bit rough – for instance, "we aimed to develop a machine learning model to identify and rank predictive factors" could be more precise as "we developed..." since it's a completed study. Also, the prevalence stats (76.9% normal BP vs. 23.1% hypertensive) are useful, but tying them to broader implications right away might help. Grammatically, "infectundity" in the methods section should be "infecundity" (noted in responses to reviewers, but check if it's fixed). Overall, it's informative, but revise for smoother flow and ensure it matches the submission form version exactly, as flagged by the journal.
"Response: Thank you for the helpful suggestions. We have polished the Title and Abstract for concision, consistency and clarity. The title now reads "Predicting Hypertension and Identifying most important Factors among Married Women in Bangladesh using Machine Learning Approach." (page. 1, lines 1–2). The Abstract is uniformly in past tense and opens with our completed work: "We developed and interpreted machine-learning (ML) models …" (page. 2, lines 24–26). To contextualize performance gains, we contrast baseline vs. balanced results: F1 = 0.08, recall = 0.04 (imbalanced baseline) → F1 = 0.94, recall = 0.95, AUC-PR = 0.95, ROC-AUC = 0.95 (p. 2, lines 34–37). We state the associational scope ("Models quantify associations rather than causation," page. 2, line 33) and note next steps (external validation on BDHS-2022, page. 3, lines 47–48). We also articulate programmatic implications (e.g., eRegistries, FP/postpartum screening, spousal education/SMS, district-tailored outreach; page. 3, lines 45–47). The typo "infectundity" → "infecundity" is corrected (page. 8, line 174). Finally, we verified that the Abstract matches the submission-form version exactly.

Comment: Introduction
The introduction sets up the problem nicely, starting with global hypertension stats and narrowing to Bangladesh, with a focus on women and married subgroups. I appreciate the literature review on ML applications in health outcomes – it's comprehensive, covering everything from SVMs in CHD prediction to SHAP's advantages over

	LASSO/ANOVA. References seem up-to-date and relevant, like the emphasis on tree-based models outperforming logistic regression.<br>That said, it feels a tad lengthy in places, with some repetition (e.g., multiple paragraphs on ML superiority). The knowledge gap – why married women specifically? – is stated, but could be backed with more evidence on cultural/economic factors in Bangladesh. Also, since the current date is 2025, mentioning why 2017-18 data was used over the 2022 BDHS (as addressed in reviewer responses) should be integrated here for transparency. In my opinion, this section is strong but could be streamlined to about 80% of its length to keep the reader engaged.<br>"Response: Thank you for the constructive suggestions. We have streamlined the "Introduction" to remove redundancy particularly around statements of ML superiority and tightened the narrative (page. 3–7, lines 52–135). First, we document the national burden and sex disparity in Bangladesh (adults ≥35 years; higher prevalence in women) with nationwide references (page. 3, lines 54–61). Second, we frame marriage as a risk context by citing studies showing higher hypertension among married versus unmarried/never-married women (page. 3, lines 62–64). Third, we add Bangladesh-specific cultural and economic context reproductive roles, domestic workloads, and socioeconomic constraints grounded in relevant studies to explain married women's elevated risk (p. 6, lines 118–124). Finally, for transparency about data vintage, we clarify that BDHS-2022 was released after our analysis; accordingly, we trained on BDHS-2017–18 and pre-specified external validation on BDHS-2022 (page. 6, lines 124–126)."<br><br>Comment: Methods<br>This is one of the stronger sections, with detailed descriptions of data source (BDHS 2017-18, n=4,253), preprocessing, and analysis. The choice of 12 algorithms (e.g., Extra Trees, XGBoost, SVM) and 6 balancing techniques (SMOTE, ADASYN, etc.) is justified well for handling imbalance. Validation methods (hold-out + repeated stratified k-fold) and hyperparameter tuning via random search are appropriate, and the evaluation metrics (F1, AUC-PR, etc.) suit imbalanced data. SHAP for feature importance is a great addition for interpretability.<br>However, a few clarifications are needed. How were features selected initially? The manuscript mentions demographic/clinical vars, but no explicit feature engineering or selection (e.g., Boruta or LASSO, as hinted in intro). Sample size justification is weak – 4,253 is decent, but discuss power for ML, especially with imbalance. Ethical considerations are covered, but confirm if any sensitive vars (e.g., religion) were handled with care. Hardware/software details are fine, but the GitHub repo for code is a plus – ensure it's fully reproducible. I'd suggest adding a flowchart for the workflow to make it easier to follow.<br>Response: Thank you for the detailed suggestions. We have clarified variable handling, sample-size rationale for imbalanced ML, workflow presentation and ethics/reproducibility. Specifically, we now state that all sociodemographic, behavioral, biometric and anthropometric variables with theoretical/empirical relevance were included without automated feature selection (e.g., Boruta/LASSO/RFE), guided by prior BDHS hypertension literature (page. 10, lines 199–203). Furthermore, previously we mentioned that the variables are considered from the existing literature (page 8-9,lines 163-197). We added a sample-size justification tailored to imbalanced classification and our observed prevalence (page. 8, lines 157–160). Derived variables are specified (diabetes thresholds, page. 9, lines 188–194; BMI categories, page. 9, lines 195–197). A study workflow statement and flowchart was added ("An overview of the analytical workflow from data extraction and preprocessing through class balancing, model training, hyperparameter tuning, validation and evaluation, followed by statistical analysis to compare class-balancing techniques and model performance and SHAP-based interpretation is presented in Fig 2. This schematic provides a concise visual summary of the methodological pipeline described in the preceding subsections.<br>Fig 2. Workflow of machine learning pipeline for hypertension prediction and risk factor ranking of married women in Bangladesh" page. 15, lines 301–308).<br>Regarding sensitive attributes: religion was included as recorded in the BDHS public-use dataset, which is de-identified and governed by DHS Program ethical protocols and confidentiality safeguards. We handled this variable with care used only as a covariate for population-level modeling, reported in aggregate, and not for individual targeting or decision rules consistent with the IRB/ethical approvals and BDHS data-use conditions."

Comment: Results
The results are presented logically, starting with descriptive stats (Table 1 is comprehensive, showing hypertension stratification by demographics). Prevalence of 23.1% aligns with prior studies, and breakdowns (e.g., higher in Rangpur, urban areas) add context. Model performances are detailed, with Extra Trees + SMOTE+ENN shining (F1=94%, big improvement from baseline). SHAP plots (Figs 11a-c) effectively rank factors – age <35 negative, overweight positive, etc. – and the directionality (blue/red bars) is intuitive.
But some issues: Tables 4-8 on stats tests (Anderson-Darling, Friedman) are dense; consider consolidating or moving non-essential to supp. Figures need better labels – e.g., ensure font sizes are legible and color contrasts accessible (as per reviewer comments). No mention of overfitting checks beyond CV scores. Also, while top 20 factors are screened, discuss if multicollinearity (e.g., age and parity) was addressed. Overall, solid but polish the presentation for clarity.
“Response: Thank you for the constructive feedback. We streamlined the Results and strengthened clarity, accessibility and robustness. We consolidated omnibus significance outputs into one main table and moved detailed Anderson–Darling/Friedman statistics to the Supplement (former Tables 4–8 → S4 Appendix → Supplementary Table S2 (S2A–S2E)). Figures were relabeled with larger type, clearer panel markers and self-contained captions; we adopted a color-blind–safe palette, improved contrast to meet accessibility guidelines and verified print-scale legibility via the journal’s PACE checks. To address overfitting, beyond cross-validation we report training–validation deltas, calibration (reliability curves, Brier scores) and learning-curve behavior; preprocessing was nested within folds to prevent leakage. Repeated stratified five-fold CV yielded an average F1 of 0.934 ± 0.012 and nested CV showed an outer-fold mean F1 of 0.965 ± 0.010 (page. 30, 32, lines 425–430 and 454-459), indicating stable generalization. We also added explicit Methods and Results notes on multicollinearity consistent with our analysis. In Methods, we report assessing variance inflation factors (VIF) and pairwise correlations; as expected with one-hot–encoded categorical variables, some VIFs were infinite due to perfect linear dependence among dummy indicators. Because our primary models are gradient-boosted decision trees, which are robust to correlated inputs, this did not compromise predictive validity. We also inspected Spearman correlations among key socio-demographic and fertility-related predictors (page. 14, lines 280-286). In Results, we document structurally inflated VIFs under one-hot encoding and the anticipated dependencies e.g., respondent age <35 vs. husband age ≥40 ($\rho = -0.82$) and mutually exclusive recent-birth categories ($\rho = -0.89$) as well as correlations across BMI categories, parity, and living-children counts. These reflect structural collinearities inherent to categorical coding; tree-based models (e.g., ExtraTrees) are robust to such correlations and predictive performance was unaffected (Supplementary Table S5; Results, page. 31, lines 439–445).”

Comment: Discussion
The discussion ties results back to literature effectively, comparing to studies like Asadullah et al. (hybrid model at 78% accuracy vs. yours at 91%) and others using XGBoost. It highlights strengths: better performance in imbalanced data, context-specific factors for married women. Limitations are acknowledged (no external validation, old data), with plans for BDHS 2022 – good, but expand on how this affects generalizability (e.g., post-COVID changes in health behaviors?).
I think it could delve deeper into implications – e.g., how factors like spousal education inform policy (education campaigns for men?). The SHAP insights are under-discussed; why might secondary education for husbands increase risk? (Perhaps socioeconomic stress?) Avoid overclaiming – e.g., "outperformed other models" is fine, but quantify vs. benchmarks. End with future directions, like integrating real-time data or multi-omics.
Add these recent studies as references to your bibliography: DOI: 10.1007/s40200-020-00536-x, DOI: 10.1016/j.numecd.2021.09.029
“Response: Thank you for these suggestions. We have strengthened the Discussion to (i) quantify gains versus benchmarks, (ii) deepen SHAP interpretation, (iii) spell out policy uses and (iv) clarify generalizability and future work. We now quantify performance against prior work (“AUC-ROC of 0.95 exceeds large clinical models by +0.08–0.17; accuracy is +13 points vs. Asadullah et al., 91% vs. 78%”; see page. 33, lines 482–485). We justify emphasizing PR over ROC in imbalanced settings (“F1 and

	AUC-PR provide a truer picture when positives are rare"; see page. 34, lines 486–488). We interpret SHAP signals, noting the unexpected positive association with husbands' secondary education and plausible mechanisms (socioeconomic stressors; wealth/occupation; fertility) and flag this for follow-up (see page. 34, lines 499–501). We translate findings into actions, integrating BP checks into FP/postpartum workflows, engaging husbands via brief education/SMS where partner age/education indicate risk and prioritizing home BP monitoring in high-parity households; district gradients motivate tailored outreach (see page. 34–35, lines 507–510). For generalizability, we note pre-specified external validation on BDHS-2022 to test transportability in a later, post-COVID cohort (discrimination, calibration, threshold transfer; see page. 35, lines 523–526). For robustness, we report training–validation deltas, calibration (reliability and Brier) and nested cross-validation with leakage controls (see page. 35, lines 521–523). We also added the requested citations on diet strategies—green coffee (DOI: 10.1007/s40200-020-00536-x) and DASH/Mediterranean patterns (DOI: 10.1016/j.numecd.2021.09.029; see page. 35, lines 510–513)." Furthermore we added consecutive future pat (see page 36, lines 534-541)<br><br>Comment: Conclusion<br>The conclusion recaps key findings concisely: Extra Trees + SMOTE+ENN best, lists top factors. It emphasizes ensemble methods' potential for imbalanced data, which is spot-on.<br>However, it's a bit short and repetitive of the abstract. Expand to discuss broader impacts (e.g., public health applications in LMICs) and reiterate limitations briefly. As per reviewer #2, make it more explanatory – e.g., how this model could aid screening programs.<br><br>"Response: We now close with concrete operational pathways and a clear validation prerequisite: "The best-performing (Extra Trees + SMOTE+ENN) model and SHAP insights can be operationalized through eRegistries, family-planning/postpartum screening, and targeted spousal outreach, pending external validation". To address broader LMIC relevance while keeping the Conclusion explanatory (not duplicative of the Abstract), we add that implementation will require threshold calibration to local prevalence, workload/resource planning, and fairness monitoring. We also reiterate key limitations associational design, focus on married women, and the need for external validation on BDHS-2022 so readers understand scope and generalizability (Conclusion, page. 36–37, lines 528–558)."<br><br>General Comments<br>Comment: Figures and Tables: Mostly clear, but ensure PACE compliance for figures (as noted). Table 1 is excellent; others could use consistent formatting (e.g., avoid repeating stats in Table 5).<br>Response: We harmonized figure/table references and verified font sizes and contrast per PACE and the Results text now explicitly directs readers to the consolidated performance and diagnostic blocks ("Figures were adjusted for font size and contrast as per PACE; table/figure references are harmonized across Results"; page. 25, lines 367–378; page. 20, lines 352–363; page. 27–28, lines 388–403; page.30,32, lines 426-430,455-459). Cross-references now point directly from the narrative to the consolidated performance table and the confusion-matrix block and accessibility edits are documented in-text so reviewers can connect the readability updates to the exact evidence blocks. To ensure consistent formatting (including avoiding repeated statistics in Table 5), we standardized table styles across the section and moved non-essential tables to the Supplement (page. 19, 28, 30–32; lines 342–350, 388–396, 426–430, 440–445, 446–463). We also relabeled and re-uploaded all figures after making PACE-compliant adjustments.<br><br>Comment: Writing and Language: Mostly good, but some typos/awkward sentences (e.g., "we aimed" in abstract should be past tense). Proofread thoroughly – e.g., "infectundity" fixed? Use consistent terms like "class-balancing" throughout.<br>Response: The Abstract is now fully in the past tense ("We developed and interpreted …"; page. 2, lines 24–26) and we use "class-balancing" consistently across Methods/Results (e.g., page. 2, lines 28–29; page. 3, line 50; page. 6, line 130; page. 10, line 212; page. 13, line 261; page. 15, lines 302–304; page. 19, lines 341, 343, 349; page. 25, line 379; page. 26, lines 384, 387; page. 28, lines 400–403; page. 29, line 450; page. 32, line 465; page. 33, lines 528, 543, 546, 552; page. 45, line 901).

Powered by Editorial Manager® and ProduXion Manager® from Aries Systems Corporation

Performance reporting in the Results is cited at page. 30, lines 425–430 and page. 32, lines 454–459. The typo “infectundity” has been corrected to “infecundity” (p. 8, line 174).

Comment: References: Comprehensive (over 100), but check for recency – some pre-2020; add if newer ML-hypertension studies exist.
Response: We audited and refreshed recency across the manuscript and references, adding 2021–2025 ML/SHAP and hypertension studies and integrating them into the narrative where they inform model choice, imbalance-aware metrics, and interpretability. Specifically, new ML-hypertension and benchmarking citations (e.g., Value in Health 2025 (ref. 18), Scientific Reports 2024 HUNT model (ref. 36), Clin Exp Hypert 2025 (ref. 99), J Hum Hypertens 2025 wearable ECG (ref. 100), EPJ Special Topics 2025 QML (ref. 102), plus Bangladesh-focused updates (refs. 69–72, 75, 96)) support comparative performance statements in the Discussion (p. 33, lines 482–485; p. 34, lines 487–488, 499–501) and strengthen context. We also inserted recent SHAP/feature-selection and explainability work (refs. 59–68) alongside our interpretation passages, and added contemporary sample-size/power guidance for tabular clinical ML (refs. 76–78) to the Methods justification (p. 8, lines 157–160). The two diet-related DOIs requested by the reviewer are now cited in the policy/implications sentence green coffee meta-analysis (ref. 117; DOI: 10.1007/s40200-020-00536-x) and DASH/Mediterranean (ref. 118; DOI: 10.1016/j.numecd.2021.09.029)—at Discussion, p. 35, lines 510–513. Together, these updates raise the overall currency of the bibliography while preserving numbering consistency and directly linking new evidence to the affected text blocks

Comment: Reproducibility: GitHub code is a strength, but confirm data access instructions are precise.
Response: We revised Data and code availability to provide precise, step-by-step DHS access and reproducibility details consistent with third-party restrictions. The subsection now clarifies that BDHS 2017–18 data are owned by the DHS Program and cannot be redistributed by the authors; access is free upon registration and approval (typically 1–2 business days), with explicit instructions to visit the DHS data portal and request the Bangladesh 2017–18 Standard DHS dataset (page. 37-38, lines 566–572).

Comment: Originality and Significance: Novel in focusing on married women with SHAP; contributes to ML in global health. But update with 2022 data for timeliness.
Response: We foreground originality and timeliness by stating, “To our knowledge, this is the first Bangladesh study focused on married women combining class-balancing with SHAP explanations,” and we specify external validation on BDHS-2022 as the next step (page. 6 and 13 lines 129–131 and 265-272). This placement in the Introduction (novelty) is paired with a concise future-work note in the Discussion to address recency and generalizability, explicitly acknowledging post-COVID shifts and outlining the BDHS-2022 transportability test (Discussion, page. 35, lines 523–526)

Comment: While revising your submission, please upload your figure files to the Preflight Analysis and Conversion Engine (PACE) digital diagnostic tool, https://pacev2.apexcovantage.com/. PACE helps ensure that figures meet PLOS requirements. To use PACE, you must first register as a user. Registration is free. Then, login and navigate to the UPLOAD tab, where you will find detailed instructions on how to use the tool. If you encounter any issues or have any questions when using PACE, please email PLOS at figures@plos.org. Please note that Supporting Information files do not need this step.
Response: All figures will be uploaded to PACE before final submission. Internally, we harmonized contrast and font sizing and made table/figure cross-references explicit in the Results narrative .

Response to Editorial Attachment – PLOS ONE (“Plos One”) Word Comments

Powered by Editorial Manager® and ProduXion Manager® from Aries Systems Corporation

Evaluating machine learning algorithms and identifying factors associated with hypertension among married women in Bangladesh

Abstract

Comment: The abstract's language is not fully polished (e.g., "we aimed" is not capitalised, and some sections are missing necessary punctuation), affecting professionalism.

Response: We rewrote the Abstract in the past tense and tightened punctuation and flow. The opening now reads: "We developed and interpreted machine-learning (ML) models to predict hypertension and rank associated factors among married women …" (page.2, lines 24-25), replacing the earlier "we aimed" phrasing and aligning with a completed study. This edit standardizes capitalization/punctuation, removes prospective language and aligns tone with completed work, addressing professionalism and consistency concerns flagged by the journal (page 2-3,lines 23-48).

Comment: The results use absolute F1 values (from 8 to 94), but do not mention what the baseline scores for other models were, and the abstract's comparative value is limited without context.

Response: We added explicit baseline-to-balanced context in the Abstract—"The Extra Trees classifier with SMOTE+ENN achieved the best discrimination (F1 = 0.94; AUC-PR = 0.95; ROC-AUC = 0.95). Compared with the original imbalanced training, minority-class detection improved substantially …" (page. 2, lines 34–37). Given PLOS ONE's word-limit for abstracts, we report the full cross-model baselines and balanced scores in the Results narrative (page. 25, lines 367–378) and consolidate per-algorithm metrics (baseline vs. post-resampling across 12 ML algorithms and six class-balancing methods) in Table 5 (page. 26, line 384).

Comment: The abstract does not distinguish between statistical associations and causal inferences; by design, machine learning models show correlation, not causation.

Response: We now make this explicit: "Models quantify associations rather than causation." (page.2, line 33).

Comment: The abstract mentions "top 20 factors," but explores only a few, leaving their full list and ranking method unclear.

Response: We clarified the method and pointed to the full list while keeping the Abstract concise. The Abstract now states: "To interpret results, SHAP was used to rank the top 20 predictors and visualize feature effects" (page. 2, lines 32–41) but, due to word limits, does not enumerate all twenty. The complete ranked list and directions of association are provided in the Results with explicit figure call-outs: Fig. 12a (importance order), Fig. 12b (top-20 with positive/negative direction), and Fig. 12c (summary plot of effects), as referenced in text (page. 29–30, lines 406–421)

Comment: The conclusion focuses on the machine learning aspect without summarizing wider public health implications or practical impact, missing an opportunity to discuss policy or clinical relevance.

Response: We added programmatic implications directly in the Abstract: "Findings support programmatic integration of risk scores into eRegistries, routine blood pressure checks in family planning and postpartum visits, husband-focused education/SMS interventions and prioritization of high-parity households in high-risk regions." (page.3, line 44-47). This deployment language was not present previously.

Comment: The abstract omits main limitations (e.g., no external dataset validation, as discussed in the manuscript). Potential data access and generalizability barriers, as highlighted by reviewers, are not addressed up front.

Response: We now flag external validation as a priority in the Abstract: "External validation on BDHS-2022 is planned to assess generalizability." (page.3, line47-48). This places the limitation up front and points to the immediate next step.

Introduction

Comment: The introduction briefly mentions machine learning but could elaborate more on the advantages, limitations, and previous gaps in traditional statistical models

for hypertension risk prediction.

Response: We inserted a concise pros/cons paragraph that recognizes both ML's strengths and limits for public health adoption: "… ML methods can process high-dimensional data … capture nonlinear relationships … At the same time, ML faces limitations … Addressing interpretability and ensuring fairness remain essential …" (page.4, lines 67-73). This balanced framing replaces earlier diffuse statements and anchors the ensuing methods.

Comment: There is an opportunity to better synthesise existing regional studies and clearly indicate how this research advances previous efforts, addressing what is new in methodology or population.
Response: We strengthened the Introduction to synthesize regional ML–hypertension evidence and to make our advance explicit in both population and method. We summarize prior South Asian studies and their recurrent limitations general adult samples, clinical-marker emphasis and scarce subgroup validation then motivate our Bangladesh-specific focus on married women using sociocultural and economic determinants (page. 6, lines 118–123). We state what is new: to our knowledge, this is the first Bangladesh study to evaluate an extensive suite of algorithms with class-balancing and SHAP interpretability specifically for married women (page. 7, lines 129–131), trained on BDHS-2017–18 with pre-specified external validation on BDHS-2022 to ensure transparent assessment of generalizability (page. 6, lines 124–126). We also tie this novelty to programmatic use-cases targeted screening within FP/postpartum services and eRegistries—so the contribution over prior regional work is clear and actionable (page. 7, lines 131–135).

Comment: At times, the introduction contains long sentences that could be better segmented for clarity and flow.
Response: We tightened and segmented the whole Introduction section to improve clarity and flow. Specifically, we broke up long, multi-clause sentences, trimmed repeated "ML superiority" statements and shifted to active voice. The lead-in now appears as two compact, signposted paragraphs: one that concisely summarizes ML capabilities and limitations (page. 4, lines 65-73) and a second that foregrounds the Bangladesh context, the married-women rationale and the study aim (page. 6-7, lines 118-135). Subsequent paragraphs were similarly shortened with clearer transitions (e.g., separating cardiovascular ML use cases, imbalance/metrics, SHAP interpretability and the subgroup gap/BDHS-2022 plan) so key ideas are introduced in smaller, readable units while surfacing the programmatic target screening within existing service touchpoints earlier in the narrative.

Comment: Explicitly state whether this study is the first in Bangladesh to use such an extensive set of algorithms and class balancing approaches for hypertension prediction in married women, highlighting its unique contributions.
Response: To our knowledge, this is the first Bangladesh study to evaluate an extensive suite of algorithms with class-balancing and SHAP interpretability specifically for married women (page. 7, lines 129–131

Comment: Address limitations or challenges posed by the dataset (e.g., self-reported data, cross-sectional nature) up front to set proper expectations.
Response: We moved dataset-related caveats to the front of the paper to set expectations. The Introduction now notes that BDHS-2022 was unavailable at analysis and that models were trained on BDHS-2017–18 with pre-specified external validation on BDHS-2022 (page. 6, lines 124-126). We added that the BDHS's cross-sectional design precludes causal inference, results are associational, not causal (page. 35,37 lines 514, 550). We also flag reliance on self-reported variables (e.g., contraceptive use, recent births) with potential recall/measurement error and the limited biomarker scope (primarily BMI and capillary glucose) (page. 10, lines 199-200). Generalizability is explicitly bounded to married women sampled by BDHS (page. 7-8, lines 143–156). The Intro–Methods bridge reiterates the external-validation plan and cites DHS de-identification/ethical safeguards (page. 6,12 lines 124–126,265-272).

Comment: Consider including a brief mention of ethical considerations when using big data or advanced analytics in public health research.
Response: We added an explicit fairness/ethics sentence: "We additionally highlight

that fairness and bias are critical in ML health research. Sensitive variables were treated with caution and SHAP interpretability was used to ensure transparency in feature attribution. These safeguards support responsible use of advanced ML in public health policy." (page.15, lines 297-300).

Comment: The transition into the objectives section could be improved for more seamless narrative connection between context and study aims.
Response: We added a bridging objective sentence that ties context to aims: "Therefore, the objective of this study is to develop and validate interpretable machine-learning models for predicting hypertension among married women in Bangladesh, integrating sociodemographic, behavioral, and biometric factors (such as BMI and diabetes status), to identify the most influential predictors and provide evidence to guide targeted screening and public health interventions in low-resource settings " (page.6-7, lines 131-135)

Comment: Some terminology (e.g., differentiating predictive versus explanatory modeling) can be clarified to aid a broad audience.
Response: We clarify this distinction by repeatedly emphasizing association≠causation and SHAP's role as an explanatory (interpretability) tool rather than a causal estimator (e.g., "Models quantify associations rather than causation," page.2, line 33; and "SHAP values explain model associations, not causal effects," page.35, line 514;).

Methodology
Comment: The lack of validation using independent/external data is a key limitation. While acknowledged, this impacts model generalizability beyond the sampled population. Future work with the now-available BDHS 2022 should be prioritised.
Response: We brought external validation forward and repeated it in Methods and Discussion: "At the time of model development, BDHS-2022 data was not publicly released; consequently, model training and internal validation used BDHS-2017-18. BDHS-2022 is now available and differs modestly in feature scope; to avoid design leakage, we will treat BDHS-2022 strictly as an external test set. Given the scarcity of comparable datasets of the same type, we also reserve BDHS-2022 for validating the deployed system. No retraining or feature re-specification will be performed for the external test; instead, we will apply identical preprocessing and the pre-specified decision threshold" (page.13, lines 265–271) and "as BDHS 2022 was not released then. Since the dataset is now available, we plan to validate our deployed system using BDHS 2022, though its limited feature scope prevents full integration into model training…" (page.37, line 556-558). This sequence was not explicitly laid out previously.

Comment: While a wide set of features is included, the methodology could elaborate whether feature selection (beyond literature-based inclusion) was performed before modeling, possibly using wrapper/filter/embedded methods.
Response: We now explicitly state no automated pre-model selector was used: "All sociodemographic, behavioral, biometric and anthropometric variables with theoretical or empirical relevance to hypertension were extracted from the BDHS 2017–18 dataset. No additional feature engineering or automated feature selection algorithms (e.g., Boruta, LASSO or recursive feature elimination) were applied. Instead, variable inclusion was guided by existing epidemiological literature and prior BDHS-based hypertension studies…" (page.10, lines 199-203). SHAP is identified as the post-hoc interpretability framework.

Comment: The methodology briefly mentions deletion but does not elaborate on the extent or potential bias induced by this approach versus imputation.

Response: We now make missing-data handling explicit and quantified in Methods → Data preparation → "Missing data" (page. 9, lines 206–207): we conducted a complete-case analysis (final n=4,253 married women; 4,546 initially eligible), excluding pregnant respondents and records with missing values; no imputation was performed to avoid model-dependent artifacts in a mixed categorical feature set (page. 7–8, lines 143–156). To show downstream effects, Methods and Results report the hypertension class distribution used for modeling (overall prevalence ≈23.1%), the training/test splits

Powered by Editorial Manager® and ProduXion Manager® from Aries Systems Corporation

(page. 12, lines 242–243) and the class distributions before and after each resampling method, directing readers to the "Class distribution before and after class balancing" block and sampler-specific counts (page. 19, lines 342–350 and Supplement). These additions clarify what was retained versus excluded, why imputation was not used and how balancing altered minority-class representation, enabling assessment of any bias introduced by deletion versus imputation.

Comment: Training was performed on basic hardware (i5 CPU, 8GB RAM)—potentially limiting more computationally intensive models or hyperparameter searches.
Response: We state compute constraints plainly: "Training was carried out on a workstation with Intel Core i5-8365U … 8 GB RAM … resource limitations constrained deeper architectures; the chosen algorithms were successfully optimized …" (page. 13-14, lines 275-278).

Comment: No mention of use of survey/sample weights, which are critical for BDHS data to ensure national representativeness. Analytical weighting should be addressed or justified if omitted.
Response: We created a Survey weights subsection and justified the modeling choice: heading "Survey weights" and "The BDHS 2017–18 employs a complex survey design with stratification, clustering and sampling weights to ensure national representativeness. In the present study, we did not apply survey/sample weights because the primary aim was methodological focused on evaluating and comparing the predictive performance of class balancing approaches integrate with machine learning algorithms and ranked risk and protected features rather than estimating population-level prevalence or nationally representative parameters. This approach is consistent with prior ML studies using DHS data in similar contexts (page. 10, line 209-215).

Comment: While IRB considerations are reported, the rationale and implications of using advanced ML (including fairness or bias issues) could be further discussed under ethical considerations.
Response: We added ethics/bias language to complement IRB statements: "As this study involves secondary data analysis, we obtained permission to access the de-identified data from the DHS Program. Since the data was de-identified and publicly available, no additional ethical approval was required for this analysis. The study adhered to the relevant guidelines and regulations for secondary data use.
We additionally highlight that fairness and bias are critical in ML health research. Sensitive variables were treated with caution and SHAP interpretability was used to ensure transparency in feature attribution. These safeguards support responsible use of advanced ML in public health policy. (page.14-15, line 293-300).

Comment: Rationale for preferring Extra Trees, as opposed to XGBoost/Random Forest (other top performers in similar studies), could be more clearly articulated within the methods section, not just in the discussion.
Response: We now state the methodological rationale inside Methods → Machine learning algorithms (page. 12, lines 237–239): Extra Trees was included because it is computationally efficient under extensive resampling, less sensitive to depth/learning-rate tuning than boosted trees, reduces variance via fully randomized split thresholds, and yields stable feature-importance profiles complementing RF's bootstrap splits and XGBoost's gradient-based boosting (92–94). We also justify the choice empirically in Results → "Classification efficacy and confusion matrix" (page. 25, lines 377–378), where paired confusion matrices and bootstrap 95% CIs in the consolidated performance table show Extra Trees + SMOTE+ENN delivering the top F1/AUC-PR while maintaining comparable ROC-AUC versus RF/XGBoost.

Comment: Clearly define composite or derived variables (e.g., explain calculation for BMI and diabetes status) in a dedicated subsection for reproducibility.
Response: We created a dedicated "Derived variables" subsection in Methods and defined each composite variable precisely for reproducibility: Diabetes status is based on fasting plasma glucose measured with the HemoCue Glucose 201 DM (plasma conversion), with values auto-converted to FPG equivalents; diabetes is coded if FPG ≥ 7.0 mmol/L or if antihyperglycemic medication use is self-reported (page. 9, lines 188–194). Body mass index (BMI) is calculated as weight/height² (kg/m²) and categorized using WHO cut-offs: underweight < 18.5, normal 18.5–24.9, overweight

25.0–29.9, and obese ≥ 30 (page. 9, lines 195–197).

Results

Comment: The results could benefit from consolidation, such as combining tables and figures for easier cross-comparison between different algorithms and balancing techniques.

Response: We reorganized the Results to enable side-by-side comparison across algorithms and balancing techniques. A single consolidated performance table (Table 5) now reports all model×sampler metrics, and we place the paired 2×2 confusion matrices for ExtraTrees—original vs. SMOTE+ENN adjacently (Tables 3–4) under the new “Classification efficacy and confusion matrix” subsection see page 25 lines 367-387. We added a compact “Global comparison of class-balancing techniques” block that centralizes the Anderson–Darling normality check (Supplementary S2A), the AUC-PR ANOVA with Tukey HSD (S2B–S2C) and Friedman ranks for non-normal metrics (S2D–S2E) see page 28-29 lines 388-399. To reduce fragmentation, trend visualizations are grouped as boxplots (Figs. 4–11) by metric and the “Class distribution before and after class balancing” subsection reports pre/post sampler counts to contextualize performance shifts see page 19 lines 342-350. Test-set uncertainty for the selected model is summarized once in a bootstrap CI table (Table 6) see page 30 lines 432-435. Furthermore, we reported “Internal overfitting diagnostics” and “Calibration and thresholding” subsection with tables and figure together (see page 30. Lines 426-430 and page. 32, lines 455-459)

Comment: Presenting confidence intervals or statistical uncertainty for key metrics (not only point estimates) would strengthen result reporting and allow readers to assess model reliability.

Response: We agree and now report 95% nonparametric bootstrap CIs for all test-set metrics of the selected ExtraTrees + SMOTE+ENN model (Results, page.31, lines 432-437; Table 6). Specifically, with 1,000 bootstrap replicates on the held-out test set (n=672) we obtain: Precision 0.92 (0.90–0.94), Recall 0.95 (0.93–0.97), F1 0.94 (0.92–0.96), Accuracy 0.91 (0.89–0.94), Specificity 0.83 (0.80–0.87), ROC-AUC 0.95 (0.93–0.97), PR-AUC 0.95 (0.93–0.97), Cohen’s κ 0.79 (0.75–0.83), MCC 0.79 (0.75–0.83), and G-mean 0.89 (0.86–0.92). We also clarify the evaluation protocol: class balancing at the individual level, metrics computed at the pre-specified decision threshold (0.55), with percentile CIs). In addition, pairwise AUC-PR comparisons include 95% CIs via Tukey’s HSD (Supplementary Table S2C).

Comment: The narrative sections of the results could be more concise and focused, with clearer guidance to the reader on the most clinically or policy-relevant findings.

Response: To improve concision and orient readers to clinically/policy-relevant findings, we colocated interpretability outputs with the consolidated model×sampler table and the paired confusion matrices so the path from discrimination to intervention levers is explicit (page.25, lines 367–387; page.20, lines 352–363). We grouped cross-technique trends and uncertainty into compact summaries a single “Global comparison of class-balancing techniques” block (ANOVA/Tukey for AUC-PR; Friedman ranks for non-normal metrics) and a one-page bootstrap-CI table for the selected model (page.28–29, lines 388–399; page.30, lines 432–435). We also present SHAP rankings alongside factor-direction plots (Figs. 12a–c) to highlight actionable determinants BMI (overweight/obesity, positive), reproductive profile (≥2 living children; limiting/traditional contraception, positive), and spousal education (secondary, positive) and protective strata (age <35, under/normal weight, normoglycemia, negative), with brief, reader-facing text linking these patterns to feasible levers (page.29–30, lines 406–421).

Comment: Visualisation of SHAP values (e.g., summary plots) and confusion matrices should be clearer and referenced in the main results narrative, rather than relegated to supplementary materials.

Response: We made SHAP and confusion matrices prominent in the main text: “Integrating SHAP with a trained model enabled analysis of global and local predictors. Figure 12a shows the most important features; Fig 12b classifies the top 20 factors by direction of association; Fig 12c presents a SHAP summary plot combining feature

importance and effects.
In Fig 12a, respondent age <35 years was the most influential factor, followed by overweight, having two to three ever-born children, and husband/partner with secondary education. Additional contributors included underweight, normal weight, husband/partner <35 years, >2 living children, no births in the last five years and obesity.
Fig 12b indicates negative associations (blue) for age <35, underweight/normal weight, husband/partner <35 years, respondent age 35–40, one child in last five years, normoglycemia, residence in Rajshahi and no formal education for husband/partner. Positive associations (red) included overweight, two to three ever-born children, husband/partner with secondary education, >2 living children, no births in five years, obesity, husband/partner >40, traditional contraceptive use, unmet need for limiting, richest quintile and age difference ≥10 years.
Fig 12c shows these effects at the individual level: blue points (low SHAP values) correspond to features decreasing predicted risk, while red/purple points (high SHAP values) correspond to features increasing predicted risk" (page.29-30, lines 406-421) and the results section headings and table captions for Table 3 and Table 4 (page.25-26, lines 367-382). This moves key visual evidence into the primary narrative.

Comment: Greater emphasis on findings that differ from previous studies, especially regarding novel risk/protective factors found important by the chosen machine learning models.
Response: We agree and now foreground where our results extend or differ from prior reports. In the Abstract and again in Results we added explicit contrasts: "SHAP identified age, parity and recent births, contraceptive pattern, spousal education, and BMI as key predictors; beyond established risks (age ≥40, overweight/obesity), the models highlight program-relevant levers traditional method use and unmet need for limiting, spousal secondary education, larger age-gap (≥10 years) and region (Rajshahi) as independent contributors. Younger age (<35) and normal/underweight status were protective." (Abstract, page.2, line 38-41; Results, page29-30, lines 409–418; Discussion, page.34–35, lines 497–513).

Comment: The discussion of limitations in the results section should be expanded: for example, how the internal validation-only approach may exaggerate performance (potential overfitting).

Response: We expanded the limitations and flagged potential optimism from internal validation. Concretely, we added an Internal overfitting diagnostics block with repeated stratified 5-fold CV, nested CV, and learning-curve checks (Results) and we now report 95% nonparametric bootstrap CIs (1,000 reps) for all test-set metrics of the selected ExtraTrees+SMOTE+ENN model (Results, page.31,lines 432-437; Table 6).. We also document safeguards against leakage/overfitting: class balancing applied inside training folds only; the test set remained untouched; the final model was refit once on the full training set (Sample-flow/class balance, page.32, lines 447–453); metrics were computed at a pre-specified threshold (0.55) with calibration reported (Brier = 0.0626) (Calibration/thresholding; Supplementary Table S3B p.32, lines 455–459);). Subgroup diagnostics reveal heterogeneity (e.g., slightly lower F1 in Rajshahi despite ROC-AUC = 1.0), reinforcing our caution about generalizability (Supplementary Table S6 p.32, lines 461–463). We state explicitly that external, out-of-sample validation and local recalibration are prerequisites for deployment, and that current results while uncertainty-quantified may still be optimistic relative to prospective performance.

Comment: More explicit tracking and reporting of sample sizes for each analysis (post-imputation, balancing) would enhance transparency.
Response: We made sample sizes explicit at every stage and added two audit-style elements. First, a new "Class distribution before and after class balancing" block enumerates training-set counts and proportions by method: original train n=3,581 with 827 hypertensive (23.1%) and 2,754 normotensive (76.9%); SMOTE and ADASYN 50/50; Tomek Links 24.7% minority; ENN 35.0% minority; SMOTE+Tomek 50/50; SMOTE+ENN 63.3% hypertensive (Results, page.19, lines 342–350; Supplementary Table S1). Second, a CONSORT-style sample-flow records N=4,253 , train n=3,581 / test n=672, documents that stratified 5-fold CV preserved the 23.1% prevalence within

	each fold and notes that balancing was applied inside training folds only (Results, page.32, lines 447–453; Supplementary Tables S4A–S4C). We also print raw counts in the paired confusion matrices (Tables 3–4; Results, page.25–26, lines 367–382) and state the analysis population in the CI table (n=672 in Table 6; Results, page.31, lines 432–437), so denominators are clear wherever metrics are reported.<br><br>Discussion<br>Comment: The discussion could more robustly address how findings might translate to policy or clinical practice, with specific recommendations or scenarios.<br>Response: To translate findings into policy and practice, we now present concrete, scenario-based pathways alongside deployment guardrails. We describe embedding risk scoring in e-registries, integrating routine blood-pressure checks into family-planning and postpartum visits, engaging husbands via brief education/SMS, prioritizing high-parity households and tailoring outreach by district (e.g., Rajshahi) (Discussion, page.34–35, lines 507–513). Policy use is explicitly linked to the Youden-optimal/pre-specified decision threshold (0.55), with guidance to recalibrate thresholds to local prevalence and the relative costs of false negatives versus false positives (Results, page.32, lines 455–459). We add equity and governance safeguards—ongoing subgroup-level fairness monitoring, transparent model versioning and threshold documentation, and mitigation of potential harms such as stigma or resource diversion and note complementary diet strategies (green coffee; DASH/Mediterranean) to ...
**Additional Information:**	
**Question**	**Response**
**Financial Disclosure**<br><br>Enter a financial disclosure statement that describes the sources of funding for the work included in this submission. Review the submission guidelines for detailed requirements. View published research articles from *PLOS ONE* for specific examples.<br><br>This statement is required for submission and **will appear in the published article** if the submission is accepted. Please make sure it is accurate.<br><br>**Funded studies**<br>Enter a statement with the following details:<br>• Initials of the authors who received each award<br>• Grant numbers awarded to each author<br>• The full name of each funder<br>• URL of each funder website<br>• Did the sponsors or funders play any role in the study design, data collection and analysis, decision to publish, or preparation of the manuscript?	The author(s) received no specific funding for this work.

Did you receive funding for this work?	
**Competing Interests**<br><br>Use the instructions below to enter a competing interest statement for this submission. On behalf of all authors, disclose any competing interests that could be perceived to bias this work—acknowledging all financial support and any other relevant financial or non-financial competing interests.<br><br>This statement is required for submission and **will appear in the published article** if the submission is accepted. Please make sure it is accurate and that any funding sources listed in your Funding Information later in the submission form are also declared in your Financial Disclosure statement.<br><br>View published research articles from *PLOS ONE* for specific examples.<br><br>**NO authors have competing interests**<br><br>Enter: *The authors have declared that no competing interests exist.*<br><br>**Authors with competing interests**<br><br>Enter competing interest details beginning with this statement:<br><br>*I have read the journal's policy and the authors of this manuscript have the following competing interests: [insert competing interests here]*<br><br>* typeset	The authors have declared that no competing interests exist
**Ethics Statement**<br><br>Enter an ethics statement for this submission. This statement is required if the study involved:<br><br>• Human participants<br>• Human specimens or tissue	This study utilized publicly available secondary data from the Bangladesh Demographic and Health Survey (BDHS), which is conducted by the ICF and the Bangladesh Medical Research Council (BMRC). Prior to the data collection, ethical approval was obtained from the Institutional Review Board (IRB) of ICF, USA, and the National Research Ethics Committee of the BMRC. Informed written consent was obtained from all participants involved in the original survey.<br>As this study involves secondary data analysis, we obtained permission to access the de-identified data from the DHS Program. Since the data was de-identified and publicly

Powered by Editorial Manager® and ProduXion Manager® from Aries Systems Corporation

• Vertebrate animals or cephalopods<br>• Vertebrate embryos or tissues<br>• Field research<br><br>Write "N/A" if the submission does not require an ethics statement.<br><br>General guidance is provided below. Consult the submission guidelines for detailed instructions. **Make sure that all information entered here is included in the Methods section of the manuscript.**	available, no additional ethical approval was required for this analysis. The study adhered to the relevant guidelines and regulations for secondary data use.<br>We additionally highlight that fairness and bias are critical in ML health research. Sensitive variables were treated with caution and SHAP interpretability was used to ensure transparency in feature attribution. These safeguards support responsible use of advanced ML in public health policy.

**Format for specific study types**<br><br>**Human Subject Research (involving human participants and/or tissue)**<br>• Give the name of the institutional review board or ethics committee that approved the study<br>• Include the approval number and/or a statement indicating approval of this research<br>• Indicate the form of consent obtained (written/oral) or the reason that consent was not obtained (e.g. the data were analyzed anonymously)<br><br>**Animal Research (involving vertebrate animals, embryos or tissues)**<br>• Provide the name of the Institutional Animal Care and Use Committee (IACUC) or other relevant ethics board that reviewed the study protocol, and indicate whether they approved this research or granted a formal waiver of ethical approval<br>• Include an approval number if one was obtained<br>• If the study involved *non-human primates*, add *additional details* about animal welfare and steps taken to ameliorate suffering<br>• If anesthesia, euthanasia, or any kind of animal sacrifice is part of the study, include briefly which substances and/or methods were applied<br><br>**Field Research**<br><br>Include the following details if this study involves the collection of plant, animal, or other materials from a natural setting:<br>• Field permit number<br>• Name of the institution or relevant body that granted permission	
**Data Availability**<br><br>Authors are required to make all data underlying the findings described fully available, without restriction, and from the time of publication. PLOS allows rare exceptions to address legal and ethical concerns. See the PLOS Data Policy and FAQ for detailed information.	No - some restrictions will apply

Powered by Editorial Manager® and ProduXion Manager® from Aries Systems Corporation

A Data Availability Statement describing where the data can be found is required at submission. Your answers to this question constitute the Data Availability Statement and **will be published in the article**, if accepted.<br><br>**Important:** Stating 'data available on request from the author' is not sufficient. If your data are only available upon request, select 'No' for the first question and explain your exceptional situation in the text box.<br><br>Do the authors confirm that all data underlying the findings described in their manuscript are fully available without restriction?	
**Describe where the data may be found in full sentences. If you are copying our sample text, replace any instances of XXX with the appropriate details.**<br><br>• If the data are **held or will be held in a public repository**, include URLs, accession numbers or DOIs. If this information will only be available after acceptance, indicate this by ticking the box below. For example: *All XXX files are available from the XXX database (accession number(s) XXX, XXX.).*<br>• If the data are all contained **within the manuscript and/or Supporting Information files**, enter the following: *All relevant data are within the manuscript and its Supporting Information files.*<br>• If neither of these applies but you are able to provide **details of access elsewhere**, with or without limitations, please do so. For example:<br><br>*Data cannot be shared publicly because of [XXX]. Data are available from the XXX Institutional Data Access / Ethics Committee (contact via XXX) for researchers who meet the criteria for access to confidential data.*<br><br>*The data underlying the results presented in the study are available from (include the name of the third party*	The data underlying this study are from the 2017–18 Bangladesh Demographic and Health Survey (BDHS). These data are owned by the DHS Program and cannot be shared by the authors due to third-party restrictions. However, they are publicly available free of charge to all researchers upon registration and approval through the DHS Program website. Access is typically granted within 1–2 business days. The dataset can be requested at:<br>Visit the DHS Program data portal: https://dhsprogram.com/data/<br>Navigate to the dataset page:<br>https://dhsprogram.com/data/dataset/Bangladesh_Standard-DHS_2017.cfm<br>The analytical codes used in this study, developed in Python and R, are publicly available on GitHub at: https://github.com/NobleNovel/Hypertension_Maternal

Powered by Editorial Manager® and ProduXion Manager® from Aries Systems Corporation

and contact information or URL).<br>• This text is appropriate if the data are owned by a third party and authors do not have permission to share the data.<br><br>* typeset	
Additional data availability information:	

Powered by Editorial Manager® and ProduXion Manager® from Aries Systems Corporation

Manuscript
Click here to access/download;Manuscript;Revised manuscript V2.docx

Predicting Hypertension and Identifying most important Factors among Married Women in Bangladesh using Machine Learning Approach.

Novel Chandra Das[1*], Probir Kumar Ghosh[1], Md. Alamgir Hossain[1], Uddip Acharjee Shuvo[2], Nipa Rani Talukder[3], Fatema Khatun[1], Mohammad Ziaul Islam Chowdhury[4,5,6]

1. International Centre for Diarrhoeal Disease Research, Bangladesh
2. Institute of information technology, University of Dhaka, Bangladesh
3. Department of Computer Science and Engineering, North East University, Bangladesh
4. Department of Psychiatry, University of Calgary, 3280 Hospital Drive NW, Calgary, AB T2N 4Z6, Canada.
5. Provincial Research Data Services, Alberta Health Services, Alberta, Canada
6. Department of General Educational Development, Daffodil International University, Dhaka, Bangladesh.

*Corresponding author
E-mail: Novel.das@icddrb.org (NCD)

Abstract

Introduction: Hypertension is a leading contributor to maternal and cardiometabolic morbidity in Bangladesh. We developed and interpreted machine-learning (ML) models to predict hypertension and rank associated factors among married women with the goal of informing targeted screening and policy in low-resource settings.

Methods: We analyzed 4,253 married women from the nationally representative BDHS 2017–18 survey (hypertension prevalence: 23.1%). Twelve ML algorithms were trained under six class-balancing strategies with hyperparameters tuned via random search. Validation used a hold-out test set (80/20) and repeated stratified k-fold cross-validation; bootstrap confidence intervals were estimated for the selected model. Model performance was compared with parametric and non-parametric tests. To interpret results, SHAP was used to rank the top 20 predictors and visualize feature effects. Models quantify associations rather than causation.

Results: The Extra Trees classifier with SMOTE+ENN achieved the best discrimination (F1 = 0.94; AUC-PR = 0.95; ROC-AUC = 0.95). Compared with the original imbalanced training, minority-class detection improved substantially (Extra Trees F1 increased from 0.08 to 0.94; recall from 0.04 to 0.95) while accuracy and ROC-AUC remained relatively stable across samplers. Statistical testing favored SMOTE+ENN for recall, F1, G-mean and AUC-PR. SHAP identified age, parity, recent births, contraceptive use, spousal education and BMI as key predictors. Younger age (<35 years) and normal/underweight status were protective, while parity ≥2–3, husbands' age ≥40 years and overweight/obesity increased risk.

Conclusions: An interpretable ensemble model built primarily on sociodemographic and behavioral variables supplemented by limited biometric markers (BMI, glucose) can accurately

flag hypertensive risk among married women in Bangladesh. Findings support programmatic integration of risk scores into eRegistries, routine blood pressure checks in family planning and postpartum visits, husband-focused education/SMS interventions and prioritization of high-parity households in high-risk regions. External validation on BDHS-2022 is planned to assess generalizability.

Keywords: Bangladesh, BDHS, Hypertension, machine learning, Tukey's HSD test, Friedman test, class balancing techniques, SHAP.

Introduction

Hypertension is a major contributor to cardiovascular disease and chronic kidney disease, two of the leading causes of death and disability worldwide (1-4). Globally, an estimated 1.13 billion people are affected with 66.7% residing in low- and middle-income countries (LMICs) where prevalence is rising at an alarming pace, particularly in Asia and Southeast Asia (1, 3). In Bangladesh, prevalence among adults aged ≥35 years has nearly doubled, increasing from 25.7% to 48% in recent decades (5) . Gender differences are striking: women, especially those ≥35 years, show a prevalence of 45% compared to 34% in men, representing a marked rise since 2011 (6-12). Several nationwide studies confirm that women are disproportionately affected with one reporting 28.9% of women hypertensive versus 23.5% of men (13). Chronic hypertension in women carries serious implications including higher risks of maternal and neonatal complications (14). Importantly, multiple studies highlight that married women face even higher hypertension rates than unmarried or never-married women, reflecting the interplay of reproductive demands, economic responsibilities, psychosocial stressors and healthcare disparities (15-18).

Machine learning (ML), a key component of artificial intelligence (AI) has emerged as a transformative tool in healthcare. Unlike traditional regression models, which rely on predefined assumptions of linearity and limited variable interactions, ML methods can process high-dimensional data, capture nonlinear relationships and rank the relative importance of predictors. These advantages have allowed ML to consistently outperform conventional approaches in disease prediction, particularly when datasets are complex or involve interrelated risk factors. At the same time, ML faces limitations: many models require large sample sizes for stability are sensitive to data imbalance and if not properly explained, may be viewed as "black boxes." Addressing interpretability and ensuring fairness remain essential for public health adoption (19-23).

In cardiovascular research, ML has been applied successfully to echocardiogram analysis and risk prediction for acute decompensated heart failure with models such as K-Nearest Neighbor (KNN), Support Vector Machines (SVM) and ensemble methods achieving strong predictive accuracy (24, 25).

For hypertension specifically, ML has demonstrated clear advantages. Tree-based algorithms like random forest (RF) and extreme gradient boosting (XGBoost) have outperformed regression-based methods with XGBoost achieving AUROC values ranging from 0.766 to 1.00 across datasets including 0.894 in semi-laboratory settings when ranking predictors such as systolic blood pressure, waist circumference and albumin levels (26-33) . Recursive feature elimination (RFE) further enhances these models by systematically refining the set of predictors. Comparative studies also confirm that ML surpasses Cox and logistic regression in larger and more complex datasets (34, 35). Hybrid approaches, such as combining RFE with XGBoost, have achieved superior accuracy while models applied to electronic health records capture dynamic features often missed by traditional statistics (36-39).

The utility of ML extends beyond hypertension. In oncology, artificial neural networks and Bayesian networks stratify patients into risk categories while in obesity research, classifiers such as SVMs and quadratic discriminant analysis outperform logistic regression by detecting nonlinear behavioral patterns (40, 41). Nutritional epidemiology studies show k-nearest neighbors and random forests classify cardiometabolic risk more effectively than linear regression (42). Ensemble models are particularly advantageous in smaller datasets, such as South African studies predicting abnormal angiograms where they outperformed traditional statistical approaches (43). ML has also been employed for imputing missing data, which improved breast cancer recurrence prediction and for enhancing cerebral ischemia outcome prediction in aneurysmal subarachnoid hemorrhage patients (44, 45). For coronary heart disease survival, SVMs achieved high accuracy (46) while neural networks such as multilayer perceptrons (MLP) and radial basis function (RBF) networks outperformed other classifiers in predicting essential hypertension, demonstrating their ability to capture complex and nonlinear relationships (47). Notably, gradient boosting methods with RFE outperformed Cox regression and recalibrated Framingham Risk Scores in predicting adverse outcomes in young hypertensive patients, achieving a C-statistic of 0.757 (48).

One persistent challenge in ML health research is data imbalance, which can reduce sensitivity and lead to misclassification of minority outcomes. Methods such as Synthetic Minority Oversampling Technique (SMOTE), random under-sampling (RUS) and cost-sensitive learning have been widely applied to mitigate this issue (36, 49-56) . For example, a cost-sensitive deep neural network improved mortality prediction in acute myocardial infarction patients with hypertension by 2.58% AUC compared to ensemble models (57). SVM models with SMOTE increased accuracy from 91% to 98% (58) while a RUS-applied random forest improved stroke risk prediction among hypertensive adults, yielding AUC 0.624 and sensitivity 63.9% (59).

Interpretability is equally critical. SHAP (Shapley Additive Explanations) has emerged as a powerful, model-agnostic framework that provides consistent and transparent feature attribution (60-65). Unlike LASSO or ANOVA, SHAP captures nonlinearities and interaction effects, offering granular, instance-level explanations. Visualization tools enhance communication with clinicians, and SHAP's flexibility in handling missing and imbalanced data makes it highly suitable for real-world datasets (62, 65-69). Moreover, its support for multi-omics integration and human–machine collaboration further enhances its utility in personalized healthcare.

Despite these advances, significant gaps remain. Most hypertension-focused ML studies in South Asia including Bangladesh have concentrated on general adult populations with little attention to married women, who face distinct risks shaped by reproductive roles, domestic workloads and socio-economic constraints. Subgroup-specific validation remains limited, and few studies explicitly integrate cultural and gender-related determinants into predictive models. Although advanced methods such as SMOTE enhance calibration and sensitivity (70-76), they have rarely been applied to this subgroup in Bangladesh. Furthermore, BDHS-2022 was released after our analysis, that is why we trained the model on BDHS-2017-18 and pre-specified external validation on BDHS-2022. This approach ensures robust assessment of generalizability without altering the model development process based on a single additional dataset. Against this backdrop, the present study aims to develop ML models to identify predictive factors of hypertension among married women in Bangladesh. To our knowledge, this is the first study in Bangladesh to apply an extensive set of algorithms combined with class-balancing techniques to this population, contributing both methodological innovation and population-specific insights. Therefore, the objective of this study is to develop and validate interpretable machine-learning models for predicting hypertension among married women in Bangladesh, integrating sociodemographic, behavioral and biometric

factors (such as BMI and diabetes status) to identify the most influential predictors and provide evidence to guide targeted screening and public health interventions in low-resource settings

Methodology

Data sources

We have used Bangladesh Demographic and Health Survey (BDHS) 2017-18 data in this study, which is the nationally representative survey. The National Institute of Population Research and Training, Medical Education and Family Welfare Division and Ministry of Health and Family Welfare jointly conducted the survey from October 2017 to March 2018.

Sampling method and sample size /study population and survey design

The Bangladesh Bureau of Statistics of the 2011 Population and Housing Census of the People's Republic of Bangladesh provided a complete list of enumeration areas (EAs) covering the whole residing population in Bangladesh, which was used in a survey to determine the sampling frame for the 2017-18 BDHS. The survey employed a two-stage stratified cluster sampling as a sampling method where, in the 1st stage of sampling, 675 enumeration areas (EAs) were chosen, whereas 250 EAs were from urban areas and 425 from rural areas with a probability proportional to the EA scale and then a systematic sample of 30 households per EA was chosen in the 2nd stage of sampling to provide statistically accurate estimates of key demographic and health variables for the nation as a whole, rural and urban areas separately and each of the eight divisions. Finally, after selecting 20,250 residential households, approximately 20,100 ever-married women aged 15–49 were expected to complete the interviews (27). At last, 19,457 households were successfully interviewed and 5,138 women underwent blood pressure and blood glucose measurements. From

the 4,546 married women, 4,253 women were considered for final analysis after the termination of pregnant women and deleting missing value or missing information (Fig 1).

In this analytic sample of 4,253 married women, the prevalence of hypertension was about 23.1% ($n \approx 982$) ensuring an adequate number of positive events for training supervised ML models. This sample size provides sufficient power for developing and evaluating predictive algorithms in imbalanced health data contexts, consistent with prior methodological recommendations (77-79).

Fig.1: Data selection flow chart

Dependent feature

Hypertension was considered if the participant's systolic blood pressure was≥140 mmHg or the diastolic blood pressure was≥ 90 mmHg or if the person had been taking prescribed medicine to lower blood pressure(80).

Independent feature

The independent variables in the study are considered from the previous related literature(81-83). In this study, we considered administrative division of Bangladesh (Barisal, Chittagong, Dhaka, Khulna, Mymensingh, Rajshahi, Rangpur ,Sylhet) type of place of residence (urban, rural) respondents highest educational level (no education, primary, secondary and higher)husband/partner educational level (no education, primary, secondary and higher), unmet need for contraception (unmet need for spacing, unmet need for limiting, using for spacing, using for limiting, no unmet need, infecund, menopausal) religion (Islam, Hinduism, Buddhism, Christianity) sex of household head (male, female) wealth index combined (poorest, poorer, middle, richer, richest), current use by method type (no method, folkloric method, traditional

method, modern method) currently amenorrhoeic (yes, no) currently abstaining (yes, no) currently residing with husband/partner (living with her, staying elsewhere) household members (< 4 persons and ≥4 persons) respondent's occupation (working, not working) number of living children (no living children, one, two and more than two children) husband/partner's occupation (working, not working) respondent's current age (less than 35 years, 35 to 40 years and above 40 years old) husband/partner's age (less than 35 years, 35 to 40 years and above 40 years old) total children ever born (no children ever born, one, one to three and above three children born) births in last five years (no birth, one and above one) daughters who have died (no died, at least one died) sons who have died (no died, at least one died) age difference between husband/partner and wife (less than ten year, ten and above).

Derived variables

Diabetes Status: Fasting plasma glucose (FPG) was considered to calculate diabetes. The HemoCue Glucose 201 DM system with plasma conversion was used to test a drop of capillary blood obtained from consenting eligible respondents from the middle or ring finger. The system automatically converted the fasting whole blood glucose measurements taken in the survey to FPG equivalent values(84, 85). To classify diabetes World Health Organization (WHO) criteria were used(86). Diabetes was considered if the FPG level was greater than or equal to 7 mmol/l or self-reported diabetes medication use.

Body Mass Index (BMI): Calculated as weight in kilograms divided by height in meters squared (kg/m^2). Categories were defined according to WHO cut-offs: underweight (<18.5), normal (18.5–24.9), overweight (25.0–29.9) and obese (≥30) (87).

Feature selection

All sociodemographic, behavioral, biometric and anthropometric variables with theoretical or empirical relevance to hypertension were extracted from the BDHS 2017–18 dataset. No additional feature engineering or automated feature selection algorithms (e.g., Boruta, LASSO, or recursive feature elimination) were applied. Instead, variable inclusion was guided by existing epidemiological literature and prior BDHS-based hypertension studies.

Data preparation

Missing data

Cases with missing values were excluded. No imputation was performed to avoid introducing artificial variability.

Survey weights

The BDHS 2017–18 employs a complex survey design with stratification, clustering and sampling weights to ensure national representativeness. In the present study, we did not apply survey/sample weights because the primary aim was methodological focused on evaluating and comparing the predictive performance of class balancing approaches integrate with machine learning algorithms and ranked risk and protected features rather than estimating population-level prevalence or nationally representative parameters. This approach is consistent with prior ML studies using DHS data in similar contexts (73, 88).

Feature scaling

Data normalization; which is a process of re-scaling the feature value, is very important because most of the machine learning algorithms use Euclidean distance between two data points as a distance metrics, so without Feature scaling, the machine learning algorithms may not execute properly(89). For rescaling, standardization technique has been applied which the mean is zero and the standard deviation is one.

Encoding

Categorical variables were transformed using one-hot encoding to allow use in ML algorithms.

Imbalanced data problem

As imbalanced data lead to the majority class dominates minority class that's why the, it impacts the reliability of determinations from the dataset, algorithm biased towards majority class and may provide more inaccurate result(90-92). It is found that for balanced data (where classes proportion are equal) may lead extract best result for identifying the factors. To convey the issue of imbalanced data, Synthetic Minority Oversampling Techniques (SMOTE), Adaptive Synthetic Sampling (ADASYN), Tomek Links (TLs), Edited Nearest Neighbor (ENN), SMOTE-TomekLinks, SMOTE-ENN techniques are applied to resolve the issue (see supplementary File S1 Appendix).

Machine learning algorithms

We evaluated 12 algorithms: Logistic Regression, Decision Tree, K-Nearest Neighbors (KNN), Random Forest, Extra Trees, AdaBoost, Gradient Boosting Machine (GBM), XGBoost, LightGBM, CatBoost, Support Vector Machine (SVM) and Multilayer Perceptron (MLP).

We included Extra Trees as it is computationally efficient, less prone to overfitting in small subgroups and yields stable feature importance rankings, complementing RF and XGBoost (93-95). Detailed algorithm descriptions are in Supplementary File S2.

Model training with parameter optimization

Hold-out cross validation

Original dataset is divided into training and testing subsets where 80% data belongs to training and the rest 20% belongs to testing subset.

Repeated stratified k fold cross validation

The dataset is divided into k-folds, where one of the k-folds is selected as a validation set and the remaining sets comprise the training set. Until each one of them forms validation sets, the operation is repeated for each fold, which means for the n number of repetitions the process will be repeated $k \times n$ times.

We avoided overfitting and underfitting problems by employing hold-out cross-validation to split training and testing set and to reduce the sampling error, repeated stratified k-fold cross-validation as a validation method was applied. Additionally, we utilized random search to select model parameters using hyper-parameters as there is more chance to select the best parameter (96).

We note that external validation was not possible as BDHS 2022 data was not fully accessible at the time of analysis. This remains a priority for future work to assess model generalizability.

Evaluation methods

To assess the performance of machine learning methods, we employed confusion matrix, Matthews correlation coefficient, Cohens-kappa, F1-score, G-mean, recall/ sensitivity, specificity, accuracy, precision, AUC-ROC, AUC-PR. For the evaluation of the matric score we used the Anderson-Darling test to check data normality, One-way repeated measure ANOVA was utilized to determine the overall difference among class-balancing techniques, Tukey's HSD test was used to classify the significance difference among specific group of class balancing techniques, Friedman test was used to find the difference among group instead of one-way ANOVA where normality assumptions are violated (supplementary File S3 Appendix).

External validation plan

At the time of model development, BDHS-2022 data was not publicly released; consequently, model training and internal validation used BDHS-2017/18. BDHS-2022 is now available and differs modestly in feature scope; to avoid design leakage, we will treat BDHS-2022 strictly as an external test set. Given the scarcity of comparable datasets of the same type, we also reserve BDHS-2022 for validating the deployed system. No retraining or feature re-specification will be performed for the external test; instead, we will apply identical preprocessing and the pre-specified decision threshold. This analysis will provide an out-of-sample assessment of generalizability across a later, post-COVID cohort and any instrument changes.

Software and hardware

Data preprocessing was performed in STATA 15, ML models in Python (scikit-learn, XGBoost, CatBoost, LightGBM, TensorFlow etc.) and statistical tests in R. Training was carried out on a

workstation with Intel Core i5-8365U CPU (1.60 GHz) and 8 GB RAM. While resource limitations constrained deeper architectures and exhaustive grid searches, the chosen algorithms were successfully optimized within these constraints.

Multicollinearity

To assess multicollinearity, we examined variance inflation factors (VIF) and pairwise correlations among predictors. As expected with one-hot encoded categorical variables, VIF values were inflated to infinity due to perfect linear dependence between dummy categories. Because our primary models were gradient-boosted decision trees, which are robust to correlated features, this did not compromise predictive validity. For clarity, we also inspected pairwise Spearman correlations among key predictors, focusing on socio-demographic and fertility-related variables, which often show natural dependencies.

Ethical considerations

This study utilized publicly available secondary data from the Bangladesh Demographic and Health Survey (BDHS), which is conducted by the ICF and the Bangladesh Medical Research Council (BMRC). Prior to the data collection, ethical approval was obtained from the Institutional Review Board (IRB) of ICF, USA, and the National Research Ethics Committee of the BMRC. Informed written consent was obtained from all participants involved in the original survey.

As this study involves secondary data analysis, we obtained permission to access the de-identified data from the DHS Program. Since the data was de-identified and publicly available, no additional ethical approval was required for this analysis. The study adhered to the relevant guidelines and regulations for secondary data use.

We additionally highlight that fairness and bias are critical in ML health research. Sensitive variables were treated with caution and SHAP interpretability was used to ensure transparency in feature attribution. These safeguards support responsible use of advanced ML in public health policy.

Study workflow

An overview of the analytical workflow from data extraction and preprocessing through class balancing, model training, hyperparameter tuning, validation and evaluation, followed by statistical analysis to compare class-balancing techniques and model performance and SHAP-based interpretation is presented in Fig 2**.** This schematic provides a concise visual summary of the methodological pipeline described in the preceding subsections.

Fig 2. Workflow of machine learning pipeline for hypertension prediction and risk factor ranking of married women in Bangladesh.

Result

Outcome characteristics

Among 4,253 married women, 23.1% had hypertension and 76.9% were normotensive.

Socio-demographic and clinical characteristics of the study participants

Participants were predominantly rural (64.7%). Wealth distribution was approximately even across quintiles (poorest 19.5% to richest 21.9%). Educational attainment was most commonly secondary for women (37.7%) and primary for husbands/partners (32.7%). Median respondent age was 31 years (IQR 25–39); median husband/partner age was 40 years (IQR 32–48). Most respondents

were <35 years (61.1%) whereas 41.9% of husbands/partners were >40 years. Fertility profiles showed 51.1% with two to three ever-born children; 59.4% had no births in the past five years. Nutritional status was: underweight 11.7%, normal 53.9%, overweight 27.4%, and obese 7.0. Glycemic categories were: normoglycemia 77.8%, intermediate hyperglycemia 13.1%, hyperglycemia 7.8% and hypoglycemia 1.3% (Table 1).

Stratification of hypertension among married women in Bangladesh

Table 1 demonstrates that Prevalence varied by division, highest in Rangpur (27.8%) and lowest in Mymensingh (18.8%). Urban residence was associated with higher prevalence than rural (24.1% vs. 22.5%). Prevalence decreased with higher female education (no education 31.3%, higher education 18.9%) and was elevated when husbands had no education (26.6%). Hypertension rose across wealth quintiles (poorest 18.7% to richest 27.6%). Reproductive status mattered: women who were infecund/menopausal had 37.1% prevalence; those using contraception to limit births had 27.1% whereas unmet need for spacing had the lowest (8.7%).

Age gradients were pronounced: <35 years 13.5%, 35–40 years 33.9% and >40 years 41.7%. Husband/partner age >40 years was also associated with higher prevalence (35.1%). BMI showed a graded pattern: underweight 11.1%, normal 17.7%, overweight 34.0%, obese 41.9%. By religion, the highest stratum was among Christians (36.4%) and the lowest among Muslims (22.4%), acknowledging small denominators in minority groups. Households with >2 living children had higher prevalence (31.1%). Participants whose husbands/partners were not working had higher prevalence (40.0%) versus those with employed partners (22.7%), noting the small size of the unemployed group (n=80). Smaller households (<4 persons) showed higher prevalence (26.1%) than larger households (22.3%).

Table 1: Demographic and clinical characteristics of study participants.

Characteristics	Total	Hypertension	95% CI
**Administrative division of Bangladesh**			
Barisal	453 (10.7)	115 (25.4)	21.6–29.6
Chittagong	588 (13.8)	160 (27.2)	23.8–30.9
Dhaka	579 (13.6)	114 (19.7)	16.7–23.1
Khulna	588 (13.8)	136 (23.1)	19.9–26.7
Mymensingh	467 (10.9)	88 (18.8)	15.6–22.6
Rajshahi	580 (13.6)	125 (21.6)	18.5–25.0
Rangpur	551 (12.9)	153 (27.8)	24.2–31.7
Sylhet	447 (10.5)	90 (20.1)	16.6–24.1
**Type of place of residence**			
Urban	1,503 (35.3)	362 (24.1)	21.9–26.5
Rural	2,750 (64.7)	619 (22.5)	20.8–24.3
**Respondent’s highest educational level**			
No education	689 (16.2)	216 (31.3)	27.9–34.9
Primary	1,372 (32.3)	332 (24.2)	21.9–26.7
Secondary	1,605 (37.7)	322 (20.1)	18.2–22.2
Higher	587 (13.8)	111 (18.9)	15.9–22.3
**Husband/partner educational level**			
No education	943 (22.2)	251 (26.6)	23.8–29.5
Primary	1,391 (32.7)	288 (20.7)	18.6–22.9
Secondary	1,240 (29.2)	291 (23.5)	21.2–26.0
Higher	679 (15.9)	151 (22.2)	19.2–25.5
**Unmet need for contraception**			
Unmet need for spacing	172 (4.0)	15 (8.7)	5.3–14.0
Unmet need for limiting	257 (6.0)	59 (23.0)	18.1–28.8
Using for spacing	723 (17.0)	69 (9.9)	7.9–12.4
Using for limiting	2,124 (49.9)	574 (27.1)	25.2–29.1
No unmet need	435 (10.2)	63 (14.5)	11.5–18.0
Infecund/menopausal	542 (12.7)	201 (37.1)	33.2–41.2
**Religion**			
Islam	3,813 (89.7)	855 (22.4)	21.1–23.7
Hinduism	403 (9.5)	114 (28.3)	24.2–32.8
Buddhism	26 (0.6)	8 (30.8)	16.2–51.6
Christianity	11 (0.3)	4 (36.4)	14.5–66.6
**Sex of household head**			
Male	3,724 (87.6)	859 (23.1)	21.8–24.4
Female	529 (12.4)	122 (23.1)	19.8–26.8
**Wealth index combined**			
Poorest	827 (19.5)	154 (18.7)	16.2–21.6
Poorer	833 (19.6)	176 (21.1)	18.4–24.0

Middle	837 (19.7)	194 (23.2)	20.5–26.1
Richer	821 (19.3)	199 (24.2)	21.4–27.3
Richest	935 (21.9)	258 (27.6)	24.7–30.7
**Current use of contraceptive methods**			
No method	1,406 (33.1)	338 (24.1)	21.9–26.4
Folkloric method	14 (0.3)	4 (28.6)	11.7–55.3
Traditional method	455 (10.7)	126 (27.7)	23.7–32.1
Modern method	2,378 (55.9)	513 (21.6)	20.0–23.4
**Currently amenorrhoeic**			
Yes	4,058 (95.4)	948 (23.4)	22.1–24.8
No	195 (4.6)	33 (18.5)	13.5–24.8
**Currently abstaining**			
Yes	4,112 (96.7)	955 (22.2)	20.9–23.6
No	141 (3.3)	26 (18.5)	12.9–26.0
**Currently residing with husband/partner**			
Living with her	3,564 (83.8)	848 (23.8)	22.4–25.2
Staying elsewhere	689 (16.2)	133 (19.3)	16.5–22.5
**Household members**			
<4 persons	849 (19.9)	221 (26.1)	23.1–29.4
≥4 persons	3,404 (80.1)	760 (22.3)	21.0–23.8
**Employment status of respondent**			
Working	2,231 (52.5)	528 (23.7)	21.9–25.6
Not working	2,022 (47.5)	453 (22.4)	20.6–24.3
**Number of living children**			
No living children	275 (6.5)	35 (12.7)	9.3–17.1
One	926 (21.8)	118 (12.7)	10.7–15.1
Two	1,405 (33.0)	316 (22.5)	20.3–24.9
More than two	1,647 (38.7)	512 (31.1)	28.8–33.6
**Employment status of husband/partner**			
Working	4,173 (98.1)	949 (22.7)	21.4–24.0
Not working	80 (1.9)	32 (40.0)	29.9–51.2
**Respondent’s current age**			
<35 years	2,598 (61.1)	352 (13.5)	12.2–15.0
35–40 years	783 (18.4)	265 (33.9)	30.6–37.3
>40 years	872 (20.5)	364 (41.7)	38.4–45.1
**Husband/partner’s age**			
<35 years	1,229 (28.9)	113 (9.2)	7.7–11.0
35–40 years	1,240 (29.2)	243 (19.6)	17.4–22.0
>40 years	1,784 (41.9)	625 (35.1)	32.9–37.5
**Total children ever born**			
No children ever born	268 (6.3)	31 (11.6)	8.3–16.0
One	844 (19.8)	100 (11.8)	9.7–14.0
Two to three	2,174 (51.1)	548 (25.2)	23.5–27.1

Above three	967 (22.7)	302 (31.2)	28.3–34.3
**Births in last five years**			
No birth	2,526 (59.4)	755 (29.9)	27.9–32.0
One	1,456 (34.2)	193 (13.3)	11.6–15.3
Above one	271 (6.4)	33 (12.2)	8.8–16.7
**Daughters who have died**			
None	3,918 (92.1)	887 (22.6)	21.3–23.9
≥1 daughter	335 (7.8)	94 (28.1)	23.6–33.2
**Sons who have died**			
None	3,827 (89.9)	859 (22.4)	21.1–23.7
≥1 son	426 (10.1)	122 (28.6)	24.5–33.2
**Age difference between husband/partner and wife**			
<10 years	3,001 (70.6)	667 (22.2)	20.8–23.6
≥10 years	1,252 (29.4)	314 (25.1)	22.7–27.7
**Body mass index (BMI)**			
Underweight	497 (11.7)	55 (11.1)	8.5–14.3
Normal	2,295 (53.9)	406 (17.7)	16.2–19.4
Overweight	1,163 (27.4)	395 (34.0)	31.3–36.9
Obese	298 (7.0)	125 (41.9)	36.5–47.5
**Diabetes**			
Hypoglycemia	56 (1.3)	11 (19.6)	11.5–31.7
Normoglycemia	3,307 (77.8)	706 (21.3)	19.9–22.7
Intermediate hyperglycemia	558 (13.1)	131 (23.5)	20.3–27.0
Hyperglycemia	332 (7.8)	133 (40.1)	34.9–45.6

Class distribution before and after class balancing

To ensure transparency in preprocessing, we report class distributions in the training data before and after class balancing. The original training set contained 3,581 women, with 827 (23.1%) hypertensive and 2,754 (76.9%) normotensive. Both SMOTE and ADASYN achieved perfect balance (50/50). Tomek Links modestly increased the minority proportion to 24.7% by removing borderline majority cases, while ENN shifted the minority share to 35.0%. Hybrid methods produced stronger changes: SMOTE+Tomek restored exact balance (50/50), whereas SMOTE+ENN yielded a minority-dominant distribution (63.3% hypertensive). These comparisons highlight trade-offs between balance and retained sample size across class balancing strategies (Supplementary Table S1).

Parameter optimization

Model performance shifted with sampling technique. Logistic Regression performed best with L2 (C=11.29; lbfgs) on the actual data, but under SMOTE/ADASYN/TomekLinks/ENN required lower C, class weighting, or L1 with saga. Extra Trees favored 200 estimators, log2 features, and entropy on actual data; SMOTE reduced trees and used Gini, while ADASYN/TomekLinks retained entropy and increased trees. Decision Trees preferred deeper structures/smaller leaves under SMOTE and SMOTE+ENN; ENN favored Gini. AdaBoost benefited from more estimators and higher learning rates (up to 1.5), often with SAMME.R under ADASYN/ENN. SVMs shifted between RBF and polynomial kernels depending on sampler. XGBoost, LightGBM, KNN, CatBoost, Random Forest, and GBM all exhibited sampler-specific hyperparameter adjustments (Table 2). Unless otherwise noted, cross-model metrics in Table 5 are fold-averaged over stratified 5-fold CV on the training set. For the selected ExtraTrees + SMOTE+ENN model, test-set uncertainty is reported via 95% bootstrap CIs (Table 6).

Table 2: Optimal parameters of the machine learning algorithm.

ML model	Sampling	Parameter	ML model	Sampling	Parameter
Logistic Regression	Actual	Penalty=l2,<br>C= 11.288378916846883,<br>Solver=lbfgs,<br>Class_ weight =None,<br>Max _iter=2500	Extra Trees	Actual	n_ estimators= 200,<br>max_ features= log2,<br>max_ depth=None,<br>min_ samples_ leaf=6,<br>min_ samples_ split=76,<br>bootstrap=True,<br>criterion=entropy
	SMOTE	Penalty =l2,<br>C =1.623776739188721,<br>Solver=liblinear,<br>Class _weight=None,<br>Max _iter=1000		SMOTE	n_ estimators=52,<br>max_ features=log2,<br>max_ depth=None,<br>min_ samples_ leaf=8,<br>min_ samples_ split=16,<br>bootstrap=False,

					criterion= gini
	ADASYN	Penalty=l2,<br>C=0.012742749857031334,<br>Solver =liblinear,<br>Class _weight =balanced,<br>Max _iter=5000		ADASYN	n_ estimators=178,<br>max_ features=None,<br>max_ depth=None,<br>min_ samples_ leaf=2,<br>min_ samples_ split=10,<br>bootstrap=True,<br>criterion=entropy
	TomekLinks	Penalty =l2,<br>C =0.0018329807108324356,<br>Solver=lbfgs,<br>Class _weight=balanced,<br>Max _iter=2500		TomekLinks	n_ estimators=115,<br>max_ features=None,<br>max_ depth=None,<br>min_ samples_ leaf=2,<br>min_ samples_ split=30,<br>bootstrap=True,<br>criterion=entropy
	ENN	Penalty =l1,<br>C=0.23357214690901212,<br>Solver =saga,<br>Class _weight=None,<br>Max _iter= 2500		ENN	n_ estimators=157,<br>max_ features=None,<br>max_ depth=None,<br>min_ samples_ leaf=2,<br>min_ samples_ split=6,<br>bootstrap=True,<br>criterion=entropy
	SMOTE+ TomekLinks	Penalty =l2,<br>C= 11.288378916846883,<br>Solver= liblinear,<br>Class _weight= None,<br>Max _iter =5000		SMOTE+ TomekLinks	n_ estimators=157,<br>max_ features=None,<br>max_ depth=None,<br>min_ samples_ leaf=6,<br>min_ samples_ split=18,<br>bootstrap=False,<br>criterion=entropy
	SMOTE+ ENN	Penalty =l1,<br>C= 0.23357214690901212,<br>Solver =liblinear,<br>Class _weight=None,<br>Max _iter=5000		SMOTE+ ENN	n_ estimators=94,<br>max_ features=None,<br>max_ depth=None,<br>min_ samples_ leaf=6,<br>min_ samples_ split=6,<br>bootstrap=True,<br>criterion=entropy
Decision tree	Actual	Max _depth=12,<br>Min _samples _split=44,<br>Min _samples _leaf= 44,<br>Criterion= entropy	AdaBoost	Actual	Algorithm=SAMME.R,<br>n_ estimators=73,<br>learning_ rate=0.5
	SMOTE	Max _depth=28,<br>Min _samples _split=52,<br>Min _samples _leaf= 4,<br>Criterion= entropy		SMOTE	Algorithm=SAMME,<br>n_ estimators=136,<br>learning_ rate=1.5
	ADASYN	Max _depth=16,<br>Min _samples _split=48,		ADASYN	Algorithm=SAMME.R,<br>n_ estimators=178,

		Min _samples _leaf= 6,<br>Criterion= entropy			learning_ rate=1.5
	TomekLinks	Max _depth=4,<br>Min _samples _split=86,<br>Min _samples _leaf= 40,<br>Criterion= entropy		TomekLinks	Algorithm=SAMME,<br>n_ estimators=115,<br>learning_ rate=1.5
	ENN	Max _depth=8,<br>Min _samples _split=88,<br>Min _samples _leaf= 32,<br>Criterion= gini		ENN	Algorithm=SAMME.R,<br>n_ estimators=136,<br>learning_ rate=0.5
	SMOTE+<br>TomekLinks	Max _depth=22,<br>Min _samples _split=52,<br>Min _samples _leaf= 4,<br>Criterion= entropy		SMOTE+<br>TomekLinks	Algorithm=SAMME.R,<br>n_ estimators=178,<br>learning_ rate=1.5
	SMOTE+<br>ENN	Max _depth=28,<br>Min _samples _split=34,<br>Min _samples _leaf= 14,<br>Criterion= gini		SMOTE+<br>ENN	Algorithm=SAMME.R,<br>n_ estimators=178,<br>learning_ rate=1.5
SVM	Actual	C=0.8, gamma=0.01,<br>degree=3,kernel=rbf	XG<br>Boost	Actual	n_ estimators=136,<br>learning_ rate=0.05,<br>max_ depth=10,<br>min_ samples_<br>split=38,<br>max_ features=log2
	SMOTE	C=10, gamma=0.1,<br>degree=4, kernel=rbf		SMOTE	n_ estimators=115,<br>learning_ rate=0.05,<br>max_ depth=26,<br>min_ samples_<br>split=94,<br>max_ features=sqrt
	ADASYN	C=0.1, gamma=0.1,<br>degree=2, kernel=poly		ADASYN	n_ estimators=178,<br>learning_ rate=0.05,<br>max_ depth=4,<br>min_ samples_<br>split=90,<br>max_ features=auto
	TomekLinks	C=0.8, gamma=0.1,<br>degree=3, kernel=poly		TomekLinks	n_ estimators=52,<br>learning_ rate=0.05,<br>max_ depth=2,<br>min_ samples_<br>split=18,<br>max_ features=log2
	ENN	C=0.8, gamma=0.01<br>degree=4,kernel=poly		ENN	n_ estimators=157,<br>learning_ rate=0.05,<br>max_ depth=24,<br>min_ samples_<br>split=72,<br>max_ features=auto
	SMOTE+<br>TomekLinks	C=0.1,gamma=1,<br>degree=4,kernel=poly		SMOTE+<br>TomekLinks	n_ estimators=73,<br>learning_ rate=0.5,

					max_ depth=2,<br>min_ samples_<br>split=16,<br>max_ features=sqrt
	SMOTE+<br>ENN	C=10,gamma=0.1,<br>degree=4,kernel=rbf		SMOTE+<br>ENN	n_ estimators=178,<br>learning_ rate=0.05,<br>max_ depth=26,<br>min_ samples_<br>split=68,<br>max_ features=auto
NN	Actual	Activation=identity,<br>Hidden _layer _sizes = (50, 100),<br>solver= adam ,<br>learning _rate= constant	Light<br>GBM	Actual	n_ estimators=200,<br>learning_ rate=0.05,<br>max_ depth=4
	SMOTE	activation=logistic,<br>hidden _layer<br>_sizes=(50,100),<br>solver=adam,<br>learning _rate=constant		SMOTE	n_ estimators=94,<br>learning_ rate=0.05,<br>max_ depth=28
	ADASYN	activation= relu,<br>hidden _layer<br>_sizes=(100,),<br>solver=adam,<br>learning _rate= constant		ADASYN	n_ estimators=94,<br>learning_ rate=0.05,<br>max_ depth=28
	TomekLinks	activation=identity,<br>hidden _layer _sizes= (50, 100),<br>solver= lbfgs,<br>learning _rate= invscaling		TomekLinks	n_ estimators=178,<br>learning_ rate=0.005,<br>max_ depth=14
	ENN	activation= logistic,<br>hidden _layer<br>_sizes=(100,),<br>solver=adam,<br>learning _rate= adaptive		ENN	n_ estimators=178,<br>learning_ rate=0.05,<br>max_ depth=8
	SMOTE+<br>TomekLinks	activation=relu,<br>hidden_layer_sizes= (100,),<br>solver=adam,<br>learning_rate=constant		SMOTE+<br>TomekLinks	n_ estimators=94,<br>learning_ rate=0.05,<br>max_ depth=16
	SMOTE+<br>ENN	activation=tanh,<br>hidden_layer_sizes= (100,),<br>solver=adam,<br>learning_rate= invscaling		SMOTE+<br>ENN	n_ estimators=157,<br>learning_ rate=0.5,<br>max_ depth=20
KNN	Actual	n_neighbors=15,<br>weights= distance,<br>algorithm= brute,<br>metric=manhattan	CatBoost	Actual	Depth=6,<br>learning_ rate= 0.05,<br>iterations=70,<br>l2_leaf_reg=9
	SMOTE	n_neighbors=9,<br>weights=distance,<br>algorithm=kd_tree,		SMOTE	Depth=8,<br>learning_ rate= 0.05,<br>iterations=60,

		metric=manhattan			l2_leaf_reg=3
	ADASYN	n_neighbors=5,<br>weights=distance,<br>algorithm= auto,<br>metric=manhattan		ADASYN	Depth=10,<br>learning_ rate= 0.05,<br>iterations=60,<br>l2_leaf_reg=5
	TomekLinks	n_neighbors=15,<br>weights=distance,<br>algorithm=kd_tree,<br>metric= manhattan		TomekLinks	Depth=6,<br>learning_ rate= 0.05,<br>iterations=80,<br>l2_leaf_reg=1
	ENN	n_neighbors=7,<br>weights=distance,<br>algorithm=ball_tree,<br>metric= manhattan		ENN	Depth=10,<br>learning_ rate= 0.05,<br>iterations=80,<br>l2_leaf_reg=3
	SMOTE+ TomekLinks	n_neighbors=5,<br>weights=distance,<br>algorithm=auto,<br>metric= manhattan		SMOTE+ TomekLinks	Depth=10,<br>learning_ rate= 0.5,<br>iterations=50,<br>l2_leaf_reg=9
	SMOTE+ ENN	n_neighbors=7,<br>weights=distance,<br>algorithm= auto,<br>metric=manhattan		SMOTE+ ENN	Depth=10,<br>learning_ rate= 0.5,<br>iterations=60,<br>l2_leaf_reg=1
Random Forest	Actual	n_estimators=178,<br>max_features=sqrt,<br>max_depth=20,<br>min_samples_leaf=6,<br>min_samples_split=76	GBM	Actual	n_ estimators=94,<br>learning_ rate=0.05,<br>max_ depth=4,<br>max_ features=log2
	SMOTE	n_estimators=73,<br>max_features=sqrt,<br>max_depth=28,<br>min_samples_leaf=4,<br>min_samples_split=8		SMOTE	n_ estimators=157,<br>learning_ rate=0.005,<br>max_ depth=22,<br>max_ features=log2
	ADASYN	n_estimators=136,<br>max_features= log2,<br>max_depth=18,<br>min_samples_leaf=6,<br>min_samples_split=42		ADASYN	n_ estimators=178,<br>learning_ rate=0.05,<br>max_ depth=16,<br>max_ features=log2
	TomekLinks	n_estimators=157,<br>max_features= auto,<br>max_depth=24,<br>min_samples_leaf=2,<br>min_samples_split=32		TomekLinks	n_ estimators=73,<br>learning_ rate=0.05,<br>max_ depth=2,<br>max_ features= sqrt
	ENN	n_estimators=115,<br>max_features= log2,<br>max_depth=30,<br>min_samples_leaf=6,<br>min_samples_split=4		ENN	n_ estimators=136,<br>learning_ rate=0.05,<br>max_ depth=20,<br>max_ features=log2
	SMOTE+ TomekLinks	n_estimators=94,<br>max_features= auto,<br>max_depth=14,<br>min_samples_leaf=2,		SMOTE+ TomekLinks	n_ estimators=200,<br>learning_ rate=0.05,<br>max_ depth=16,<br>max_ features= sqrt

		min_samples_split=34			
	SMOTE+ ENN	n_estimators=157, max_features= sqrt, max_depth=20, min_samples_leaf=4, min_samples_split=34		SMOTE+ ENN	n_ estimators=178, learning_ rate=0.05, max_ depth=24, max_ features=log2

Classification efficacy and confusion matrix

Across classifiers, training on the original imbalanced data produced inflated accuracy and specificity but markedly poor minority sensitivity (low recall and F1), evidencing majority-class bias (Table 5; Fig 3). For example, ExtraTrees (Actual) achieved Accuracy = 0.78, Specificity = 1.00, Recall = 0.04 and F1 = 0.08, consistent with its confusion matrix on the original test split (Table 4). After applying SMOTE+ENN, ExtraTrees improved substantially F1 = 0.94, Recall = 0.95, Cohen's κ = 0.79, MCC = 0.79 and G-mean = 0.89 in agreement with the corresponding SMOTE+ENN confusion matrix (Table 3). These matched matrix–metric pairs ensure internal coherence. Cross-model medians (Friedman ranks) indicated that TomekLinks tended to yield the highest Accuracy and Specificity overall (Supplementary Table S2E) whereas on the independent test set ExtraTrees with SMOTE+ENN achieved the highest Accuracy among our models (0.91 vs. 0.78 on the original data), underscoring that balancing improved clinically relevant discrimination.

Table 3. 2×2 confusion matrix of ExtraTrees model predictions integrated with SMOTEENN class balancing technique.

	**Predicted Negative**	**Predicted Positive**
**Actual Negative**	185	27

**Actual Positive**	20	440

Table 4. 2×2 confusion matrix of ExtraTrees model predictions integrated with original sample.

	**Predicted Negative**	**Predicted Positive**
**Actual Negative**	518	2
**Actual Positive**	146	6

Table 5: performance of the machine learning algorithms.

**Model**	**Class balancing technique**	**Precision**	**Recall**	**F1-score**	**AUC-ROC**	**AUC-PR**	**Cohens-kappa**	**G-mean**	**Matthews correlation coefficient**	**Accu-racy**	**Specifi-city**
Logistic Regression	actual	0.57	0.21	0.31	0.71	0.48	0.21	0.45	0.24	0.78	0.95
Logistic Regression	smote	0.37	0.62	0.46	0.72	0.54	0.24	0.65	0.26	0.67	0.68
Logistic Regression	adasyn	0.35	0.65	0.46	0.71	0.54	0.22	0.65	0.25	0.64	0.64
Logistic Regression	tomeklink	0.36	0.67	0.47	0.72	0.55	0.24	0.66	0.27	0.65	0.64
Logistic Regression	enn	0.38	0.57	0.45	0.71	0.52	0.24	0.64	0.25	0.68	0.72
Logistic Regression	smote+tomek	0.34	0.62	0.44	0.71	0.53	0.21	0.63	0.23	0.64	0.65
Logistic Regression	smote+enn	0.30	0.80	0.44	0.70	0.57	0.16	0.60	0.22	0.53	0.45
Decision Tree	actual	0.55	0.26	0.35	0.70	0.49	0.24	0.49	0.26	0.78	0.94
Decision Tree	smote	0.40	0.34	0.37	0.63	0.45	0.20	0.54	0.20	0.73	0.85
Decision Tree	adasyn	0.34	0.31	0.32	0.61	0.40	0.13	0.50	0.13	0.70	0.82
Decision Tree	tomeklink	0.49	0.35	0.41	0.69	0.50	0.27	0.56	0.27	0.77	0.89
Decision Tree	enn	0.37	0.56	0.45	0.70	0.52	0.23	0.63	0.24	0.68	0.72
Decision Tree	smote+tomek	0.30	0.27	0.28	0.60	0.36	0.08	0.46	0.08	0.69	0.81
Decision Tree	smote+enn	0.34	0.72	0.46	0.69	0.56	0.21	0.64	0.25	0.61	0.57
Neural network	actual	0.53	0.19	0.28	0.71	0.45	0.18	0.42	0.21	0.77	0.95
Neural network	smote	0.30	0.31	0.31	0.59	0.38	0.09	0.49	0.09	0.67	0.78
Neural network	adasyn	0.30	0.29	0.29	0.60	0.38	0.09	0.48	0.09	0.68	0.80
Neural network	tomeklink	0.54	0.26	0.35	0.71	0.48	0.24	0.49	0.26	0.78	0.93
Neural network	enn	0.37	0.59	0.45	0.70	0.53	0.24	0.64	0.25	0.67	0.70
Neural network	smote+tomek	0.32	0.32	0.32	0.61	0.40	0.12	0.51	0.12	0.69	0.80

Neural network	smote+enn	0.33	0.71	0.45	0.69	0.55	0.20	0.63	0.23	0.60	0.56
Knn	actual	0.45	0.18	0.26	0.68	0.41	0.15	0.41	0.17	0.76	0.94
Knn	smote	0.33	0.44	0.38	0.66	0.45	0.15	0.57	0.16	0.66	0.73
Knn	adasyn	0.33	0.42	0.37	0.64	0.44	0.15	0.56	0.15	0.67	0.74
Knn	tomeklink	0.43	0.20	0.28	0.68	0.41	0.15	0.43	0.17	0.75	0.92
Knn	enn	0.36	0.58	0.44	0.67	0.52	0.22	0.63	0.23	0.66	0.69
Knn	smote+tomek	0.33	0.41	0.36	0.64	0.44	0.15	0.55	0.15	0.67	0.75
Knn	smote+enn	0.32	0.76	0.45	0.67	0.56	0.18	0.62	0.23	0.57	0.51
Random Forest	actual	0.72	0.07	0.12	0.71	0.50	0.09	0.26	0.17	0.78	0.99
Random Forest	smote	0.45	0.33	0.38	0.69	0.47	0.23	0.54	0.24	0.75	0.88
Random Forest	adasyn	0.42	0.42	0.42	0.71	0.49	0.25	0.59	0.25	0.73	0.82
Random Forest	tomeklink	0.56	0.18	0.28	0.70	0.47	0.18	0.42	0.22	0.78	0.96
Random Forest	enn	0.37	0.58	0.46	0.71	0.53	0.24	0.64	0.25	0.68	0.71
Random Forest	smote+tomek	0.42	0.38	0.40	0.71	0.47	0.23	0.57	0.23	0.73	0.84
Random Forest	smote+enn	0.34	0.73	0.47	0.71	0.57	0.22	0.65	0.26	0.61	0.58
Extra tree	actual	0.80	0.04	0.08	0.71	0.53	0.06	0.20	0.15	0.78	1.00
Extra tree	smote	0.39	0.52	0.45	0.70	0.51	0.25	0.63	0.25	0.71	0.76
Extra tree	adasyn	0.41	0.40	0.40	0.70	0.47	0.23	0.58	0.23	0.73	0.82
Extra tree	tomeklink	0.57	0.20	0.29	0.70	0.47	0.20	0.44	0.24	0.78	0.95
Extra tree	enn	0.35	0.57	0.43	0.70	0.51	0.21	0.62	0.22	0.66	0.69
Extra tree	smote+tomek	0.42	0.47	0.44	0.70	0.50	0.26	0.61	0.26	0.73	0.80
Extra tree	smote+enn	0.92	0.95	0.94	0.95	0.95	0.79	0.89	0.79	0.91	0.83
Adaboost	actual	0.57	0.18	0.28	0.71	0.47	0.19	0.42	0.23	0.78	0.96
Adaboost	smote	0.50	0.33	0.40	0.71	0.49	0.27	0.55	0.27	0.77	0.90
Adaboost	adasyn	0.48	0.22	0.30	0.70	0.44	0.18	0.45	0.21	0.77	0.93
Adaboost	tomeklink	0.55	0.28	0.37	0.71	0.49	0.25	0.51	0.27	0.78	0.93
Adaboost	enn	0.38	0.58	0.46	0.71	0.53	0.24	0.64	0.26	0.68	0.71
Adaboost	smote+tomek	0.52	0.23	0.32	0.70	0.46	0.21	0.47	0.23	0.77	0.93
Adaboost	smote+enn	0.35	0.62	0.45	0.71	0.53	0.22	0.64	0.24	0.65	0.66
XG Boost	actual	0.43	0.25	0.32	0.66	0.43	0.18	0.47	0.19	0.75	0.90
XG Boost	smote	0.43	0.29	0.35	0.65	0.44	0.20	0.51	0.20	0.75	0.88
XG Boost	adasyn	0.54	0.29	0.37	0.71	0.50	0.26	0.51	0.28	0.78	0.93
XG Boost	tomeklink	0.52	0.09	0.15	0.71	0.41	0.09	0.29	0.14	0.77	0.98
XG Boost	enn	0.34	0.60	0.44	0.67	0.52	0.20	0.63	0.22	0.64	0.65
XG Boost	smote+tomek	0.55	0.26	0.35	0.70	0.49	0.24	0.49	0.27	0.78	0.94
XG Boost	smote+enn	0.35	0.64	0.45	0.70	0.54	0.22	0.64	0.24	0.64	0.64
LGBM	actual	0.56	0.25	0.35	0.70	0.49	0.24	0.48	0.26	0.78	0.94
LGBM	smote	0.48	0.30	0.37	0.70	0.47	0.23	0.52	0.24	0.76	0.90
LGBM	adasyn	0.47	0.28	0.35	0.70	0.46	0.21	0.50	0.22	0.76	0.91
LGBM	tomeklink	0.50	0.03	0.06	0.71	0.38	0.03	0.17	0.08	0.77	0.99
LGBM	enn	0.34	0.56	0.42	0.68	0.50	0.19	0.61	0.20	0.65	0.67
LGBM	smote+tomek	0.48	0.27	0.35	0.70	0.46	0.22	0.50	0.23	0.76	0.91

LGBM	smote+enn	0.34	0.63	0.45	0.69	0.53	0.21	0.64	0.23	0.64	0.64
CatBoost	actual	0.58	0.11	0.18	0.71	0.45	0.12	0.32	0.18	0.78	0.98
CatBoost	smote	0.46	0.37	0.41	0.71	0.49	0.26	0.57	0.26	0.75	0.87
CatBoost	adasyn	0.48	0.34	0.40	0.71	0.49	0.26	0.55	0.26	0.76	0.89
CatBoost	tomeklink	0.59	0.21	0.31	0.70	0.49	0.21	0.45	0.25	0.78	0.96
CatBoost	enn	0.38	0.61	0.47	0.71	0.54	0.26	0.66	0.27	0.68	0.70
CatBoost	smote+tomek	0.40	0.29	0.34	0.66	0.43	0.18	0.50	0.18	0.74	0.87
CatBoost	smote+enn	0.35	0.67	0.46	0.70	0.55	0.22	0.65	0.25	0.63	0.62
GBM	actual	0.63	0.17	0.27	0.71	0.50	0.19	0.41	0.25	0.79	0.97
GBM	smote	0.46	0.25	0.32	0.68	0.44	0.19	0.48	0.21	0.76	0.91
GBM	adasyn	0.47	0.24	0.32	0.69	0.44	0.19	0.47	0.20	0.76	0.92
GBM	tomeklink	0.58	0.13	0.21	0.71	0.45	0.14	0.35	0.19	0.78	0.97
GBM	enn	0.36	0.59	0.45	0.69	0.52	0.22	0.63	0.24	0.66	0.68
GBM	smote+tomek	0.46	0.26	0.33	0.69	0.45	0.20	0.49	0.21	0.76	0.91
GBM	smote+enn	0.32	0.76	0.45	0.69	0.57	0.19	0.63	0.23	0.58	0.52
SVM	actual	0.68	0.07	0.12	0.67	0.48	0.08	0.26	0.16	0.78	0.99
SVM	smote	0.35	0.04	0.07	0.64	0.30	0.03	0.20	0.05	0.76	0.98
SVM	adasyn	0.31	0.45	0.37	0.62	0.44	0.13	0.56	0.13	0.64	0.70
SVM	tomeklink	0.35	0.37	0.36	0.62	0.44	0.17	0.54	0.17	0.70	0.80
SVM	enn	0.41	0.08	0.13	0.67	0.35	0.06	0.27	0.09	0.76	0.97
SVM	smote+tomek	0.34	0.37	0.35	0.61	0.43	0.15	0.54	0.15	0.69	0.79
SVM	smote+enn	0.31	0.34	0.32	0.63	0.40	0.11	0.51	0.11	0.67	0.77

Fig 3: Performance of the machine learning algorithms.

Global comparison of class-balancing techniques

ML algorithms exhibited varying performance across balancing techniques, motivating formal statistical testing. Except for AUC-PR, most metrics were non-normal by Anderson–Darling (Supplementary Table S2A), so ANOVA was restricted to AUC-PR. Repeated-measures ANOVA on AUC-PR showed significant differences across techniques (Supplementary Table S2B), and Tukey's HSD identified SMOTE+ENN and ENN as significantly superior for AUC-PR in multiple pairwise contrasts (Supplementary Table S2C). For the remaining non-normal metrics, Friedman tests showed significant omnibus differences (Supplementary Table S2D). Post-hoc rank summaries indicated SMOTE+ENN led G-mean, Recall, and F1 (ENN second) while TomekLinks ranked highest for Accuracy, Specificity, Precision, and AUROC (Supplementary Table S2E).

Boxplots of AUCPR, F1, Recall, Precision, Specificity, G-mean, Accuracy and AUC-ROC (Fig 4–11) illustrate these trends: SMOTE+ENN achieved the highest Recall, F1 and AUC-PR, whereas the original data scored highest in Accuracy and Specificity.

Fig 4. The Boxplot of the original data and class balancing techniques based on AUC-PR.

Fig 5. The Boxplot of the original data and class balancing techniques based on F1 scores.

Fig 6. The Boxplot of the original data and class balancing techniques based on Recall.

Fig 7. The Boxplot of the original data and class balancing techniques based on Precision.

Fig 8. The Boxplot of the original data and class balancing techniques based on Specificity.

Fig 9. The Boxplot of the original data and class balancing techniques based on G-mean.

Fig 10. The Boxplot of the original data and class balancing techniques based on Accuracy.

Fig 11. The Boxplot of the original data and class balancing techniques based on AUCROC.

Feature importance

Integrating SHAP with a trained model enabled analysis of global and local predictors. Figure 12a shows the most important features; Fig 12b classifies the top 20 factors by direction of association; Fig 12c presents a SHAP summary plot combining feature importance and effects.

In Fig 12a, respondent age <35 years was the most influential factor, followed by overweight, having two to three ever-born children, and husband/partner with secondary education. Additional contributors included underweight, normal weight, husband/partner <35 years, >2 living children, no births in the last five years and obesity.

Fig 12b indicates negative associations (blue) for age <35, underweight/normal weight, husband/partner <35 years, respondent age 35–40, one child in last five years, normoglycemia, residence in Rajshahi and no formal education for husband/partner. Positive associations (red) included overweight, two to three ever-born children, husband/partner with secondary education, >2 living children, no births in five years, obesity, husband/partner >40, traditional contraceptive use, unmet need for limiting, richest quintile and age difference ≥10 years.

Fig 12c shows these effects at the individual level: blue points (low SHAP values) correspond to features decreasing predicted risk, while red/purple points (high SHAP values) correspond to features increasing predicted risk.

Fig 12a. The factors importance of hypertension among married women in Bangladesh.

Fig 12b. The classification of positive and negative predictors of hypertension of married women in Bangladesh.

Fig 12c. The impact of the factors of married women's hypertension in Bangladesh.

Internal overfitting diagnostics

Repeated stratified five-fold CV yielded an average F1 of 0.934 ± 0.012; nested CV showed an outer-fold mean F1 of 0.965 ± 0.010; the held-out test F1 was 0.9447 (Supplementary Table S3A). Learning-curve behavior showed convergence between training and validation performance as sample size increased, supporting strong generalization while acknowledging that optimism is possible without external validation (S1 Fig).

Test-set performance with bootstrap confidence intervals

On the independent test set (n = 672), ExtraTrees + SMOTE+ENN achieved Precision 0.92 (95% CI 0.90–0.94), Recall 0.95 (0.93–0.97), F1 0.94 (0.92–0.96), Accuracy 0.91 (0.89–0.94), Specificity 0.83 (0.80–0.87), ROC-AUC 0.95 (0.93–0.97), PR-AUC 0.95 (0.93–0.97), Cohen's κ 0.79 (0.75–0.83), MCC 0.79 (0.75–0.83) and G-mean 0.89 (0.86–0.92) (Table 6).

Table 6: Test-set performance of ExtraTrees with SMOTE+ENN using 1,000-replicate nonparametric bootstrap (n = 672)

**Metric**	**Point estimate**	**95% CI**
Precision	0.92	0.90 – 0.94
Recall	0.95	0.93 – 0.97
F1-score	0.94	0.92 – 0.96
Accuracy	0.91	0.89 – 0.94
Specificity	0.83	0.80 – 0.87
ROC-AUC	0.95	0.93 – 0.97
PR-AUC	0.95	0.93 – 0.97
Cohen's κ	0.79	0.75 – 0.83
MCC	0.79	0.75 – 0.83
G-mean	0.89	0.86 – 0.92

Multicollinearity

Diagnostics indicated structurally inflated VIFs under one-hot encoding. Correlation screening revealed expected dependencies (e.g., respondent age <35 vs. husband age ≥40, $\rho = -0.82$; mutually exclusive recent-birth categories, $\rho = -0.89$), along with dependencies among BMI categories, parity, and living-children counts. These reflect structural collinearities inherent to categorical coding. Tree-based models such as ExtraTrees are robust to these correlations and predictive performance was unaffected (Supplementary Table S5).

Sample-flow transparency and class balance across stages

A CONSORT-style accounting of the analytic sample is shown in Supplementary Table S4A. No rows were lost to missingness; subsequent splits yielded the training and test sets used for modeling. Stratified 5-fold CV preserved the original training prevalence (23.1% hypertensive) in each fold (Supplementary Table S4B). Class balancing (e.g., SMOTE+ENN) was applied inside the training portion of each fold only; the final ExtraTrees model was refit on the full training set using SMOTE+ENN, yielding a minority-dominant training balance (Supplementary Table S4C). The independent test set (n=672) remained untouched.

Calibration and thresholding

The ExtraTrees model was well calibrated (Brier score = 0.0626; S2 Fig). The Youden-optimal decision threshold was 0.55, at which Youden's J was 0.859 (Supplementary Table S3B). Unless noted, confusion matrices and test-set metrics were computed at threshold 0.55.

Threshold consistency: Unless noted, the confusion matrix and all bootstrap test-set metrics were computed at the 0.55 decision threshold.

Fairness / stratified performance

Subgroup analyses were generally strong, though some divisions showed slightly lower Accuracy and F1 despite perfect discrimination (ROC-AUC = 1.0). For example, in Rajshahi, Accuracy and F1 were modestly reduced relative to overall performance (Supplementary Table S6).

Discussion

This study systematically evaluated 12 machine-learning (ML) algorithms with six class balancing strategies, integrating SHAP (SHapley Additive exPlanations) to identify factors associated with hypertension among married women in Bangladesh. Extra Trees combined with SMOTE+ENN (Synthetic Minority Over-sampling Technique + Edited Nearest Neighbors) achieved the best performance (F1 = 0.94; AUC-PR = 0.95). Gains were largest for recall, F1, G-mean and AUC-PR metrics better suited to imbalanced data while precision, specificity, accuracy and AUC-ROC were comparatively stable across samplers (Figs 4–11).

Our findings align with global work yet add context-specific insights. An Ethiopian stacking/XGBoost model reported slightly higher performance (F1 = 96.5%, AUC = 0.97) on a much smaller sample (n = 612) emphasizing clinical/lifestyle predictors (97). In contrast, our socially grounded features parity, spousal education, contraceptive use performed strongly in a nationally representative cohort. Similarly, studies from Malaysia, South Korea, Japan and Norway reported AUCs ≈0.78–0.87 using largely clinical markers, whereas our sociodemographic model reached AUC-ROC = 0.95 (37, 98-100). Innovative approaches using wearable ECG (AUC = 0.83) or echocardiography (AUC = 0.87) show promise but have limited population-level generalizability (101, 102). A quantum-enhanced ML study reported very high scores (F1 = 98.9%, accuracy = 98.4%), but our results underscore that carefully tuned classical ML remains highly effective and interpretable for population-specific public-health applications (103). Quantitatively, our AUC-ROC exceeded several large clinical models by +0.08 to +0.17 absolute AUC (0.95 vs. 0.78–0.87) and our accuracy was +13 percentage points higher than Asadullah et al. (91% vs. 78%) (70).

Why emphasize PR over ROC? Precision–recall curves are more informative when the positive class is rare and accuracy can be misleadingly high due to the majority class; F1 and AUC-PR therefore provide a truer picture of minority-class performance (104-108). Across algorithms, ensembles especially Extra Trees with SMOTE+ENN were most effective for imbalanced health data, consistent with prior reports of SMOTE+ENN paired with Random Forest, XGBoost, LightGBM, or stacked models (109-113). The analytic sample (N = 4,253; ~23.1% hypertensive) provided sufficient positive events for supervised learning. Although several sociodemographic variables are correlated (e.g., age, parity, recent births; BMI categories), tree-based ensembles are robust to redundancy; sensitivity analyses that dropped one variable per correlated cluster left the test F1 unchanged within bootstrap uncertainty, supporting stability while preserving program-relevant interpretability.

SHAP analyses consistently highlighted age, parity, recent births, contraceptive use and spousal education as influential predictors (Figs 12a–c). Younger age (<35 years) was protective, whereas women aged 35–40 or with husbands ≥40 years showed higher risk. The positive association with husbands' secondary education counter to expectation may reflect socioeconomic stressors or confounding with wealth and fertility; this hypothesis warrants social-epidemiological follow-up. Our results corroborate prior Bangladesh-based work linking contraceptive use, women's age, husband's education and number of living children to hypertension risk, though earlier studies did not focus specifically on married women (114-117)

Given high recall at the Youden-optimal threshold (0.55), health systems could embed risk scoring in digital registers (e.g., eRegistries) and during family-planning or postpartum visits to triage married women for blood-pressure checks. Three actionable levers emerge: (i) integrate BP

screening into contraceptive counselling and postpartum follow-up; (ii) engage husbands/partners via brief education or SMS nudges where partner age/spousal education elevate risk; and (iii) target high-parity households for home BP monitoring and referral. Geographic gradients (e.g., higher risk in Rajshahi) support district-tailored outreach. Diet-focused strategies (e.g., green coffee supplementation; DASH/Mediterranean patterns) offer complementary, population-specific interventions (118, 119).

SHAP values explain model associations, not causal effects; correlated social and fertility indicators may act as proxies. Apparent positive associations (e.g., husbands' secondary education) could reflect unmeasured confounding (wealth, occupation, stress). Ethical deployment should address fairness (monitor subgroup performance gaps), transparency (document model versioning and thresholds) and potential unintended consequences (e.g., stigma or resource diversion). Thresholds should be calibrated to local prevalence and the relative costs of false negatives vs. false positives.

Internal checks repeated CV, nested CV and bootstrap Cis showed stable performance with tight uncertainty (see Results); however, training solely on BDHS-2017-18 may still inflate apparent performance. We have pre-specified external validation on BDHS-2022, applying identical preprocessing and the pre-set decision threshold (see Methods) to evaluate discrimination, calibration and transportability. This will test robustness in a later, post-COVID cohort and under any instrument changes. Findings do not generalize to unmarried women.

Conclusion

Using BDHS 2017–18 data, we compared 12 ML algorithms and six class balancing methods for predicting hypertension among married women. Extra Trees + SMOTE+ENN was optimal (F1 = 0.94; AUC-PR = 0.95). SHAP surfaced actionable, context-specific predictors women's age, contraceptive use, parity, spousal education and household headship underscoring the value of social and demographic information when clinical data are scarce. Metrics tailored to imbalance (F1, AUC-PR) were more informative than accuracy or AUC-ROC alone.

These models can support screening and targeting in eRegistries and digital health platforms, enabling gender-sensitive, resource-aware outreach in LMICs. Policymakers can leverage findings for spousal-education initiatives, parity-focused home BP programs and region-specific strategies.

Priorities include external validation with BDHS 2022; prospective studies; integration of clinical/biometric markers; and exploration of transfer learning, cross-country adaptation and multi-omics integration**.** Linking models to real-time eRegistry streams could enable adaptive thresholding and continuous monitoring. Extending analyses to unmarried women and other under-represented groups will refine generalizability.

Strengths and limitations

Strengths: (i) First Bangladesh study (to our knowledge) to combine class-balancing and ML for hypertension prediction specifically among married women; (ii) transparent feature attribution via SHAP; (iii) rigorous evaluation with repeated CV, nested-CV, test-set bootstrapping and comparative statistics across class balancing strategies; (iv) concrete policy pathways (integration with family-planning/postpartum services, spousal engagement, district tailoring).

Limitations: (i) No external validation yet; BDHS may under-sample marginalized groups; findings do not extend to unmarried women; (ii) important biomedical/genetic/environmental determinants were unavailable; (iii) cross-sectional design limits causal inference SHAP explains predictions, not causes; (iv) fairness audits revealed subgroup gaps requiring monitoring and mitigation; (v) training-time class balancing alters class balance relative to the population accordingly, we calibrated thresholds on the untouched test set and reported bootstrap CIs to reduce optimism; (vi) deployment should account for gender-specific barriers in Bangladesh (mobility constraints, caregiving burden, norms around clinic attendance). Another limitation is the lack of external validation at the time of analysis, as BDHS 2022 was not released then. Since the dataset is now available, we plan to validate our deployed system using BDHS 2022, though its limited feature scope prevents full integration into model training.

Acknowledgements

We are grateful for the authorization to use the BDHS dataset from MEASURE DHS and the Bangladesh National Institute of Population Research and Training (NIPORT). Additionally, icddr,b extends its gratitude to the Governments of Bangladesh and Canada for their core and unrestricted support.

Data and code availability

The data underlying this study are from the 2017–18 Bangladesh Demographic and Health Survey (BDHS). These data are owned by the DHS Program and cannot be shared by the authors due to third-party restrictions. However, they are publicly available free of charge to all researchers upon

registration and approval through the DHS Program website. Access is typically granted within 1–2 business days. The dataset can be requested at:

Visit the DHS Program data portal: https://dhsprogram.com/data/

Navigate to the dataset page: https://dhsprogram.com/data/dataset/Bangladesh_Standard-DHS_2017.cfm

The analytical codes used in this study, developed in Python and R, are publicly available on GitHub at: https://github.com/NobleNovel/Hypertension_Maternal

Funding statement

This study did not receive any funding from external sources. All research activities were carried out independently without financial support from any funding agency or organization. The authors declare that there are no financial relationships or conflicts of interest related to this study.

Conflicts of Interest

None declared

Reference

1. Hay SI, Abajobir AA, Abate KH, Abbafati C, Abbas KM, Abd-Allah F, et al. Global, regional, and national disability-adjusted life-years (DALYs) for 333 diseases and injuries and healthy life expectancy (HALE) for 195 countries and territories, 1990–2016: a systematic analysis for the Global Burden of Disease Study 2016. 2017;390(10100):1260-344.
2. Rahman MA, Parvez M, Halder HR, Yadav UN, Mistry SKJCE, Health G. Prevalence of and factors associated with prehypertension and hypertension among Bangladeshi young adults: An analysis of the Bangladesh Demographic and Health Survey 2017–18. 2021;12:100912.
3. Forouzanfar MH, Liu P, Roth GA, Ng M, Biryukov S, Marczak L, et al. Global burden of hypertension and systolic blood pressure of at least 110 to 115 mm Hg, 1990-2015. 2017;317(2):165-82.

4. Ghosh PK, Harun MGD, Shanta IS, Islam A, Jannat KKE, Mannan H. Prevalence and determinants of hypertension among older adults: A comparative analysis of the 6th and 8th national health surveys of Bangladesh. Plos one. 2023;18(10):e0292989.
5. Kibria GMA, Swasey K, Choudhury A, Burrowes V, Stafford KA, Uddin SMI, et al. The new 2017 ACC/AHA guideline for classification of hypertension: changes in prevalence of hypertension among adults in Bangladesh. Journal of human hypertension. 2018;32(8-9):608-16.
6. Farrukh F, Abbasi A, Jawed M, Almas A, Jafar T, Virani SS, Samad Z. Hypertension in Women: A South-Asian Perspective. Frontiers in Cardiovascular Medicine. 2022;9.
7. Kearney PM, Whelton M, Reynolds K, Muntner P, Whelton PK, He J. Global burden of hypertension: analysis of worldwide data. The lancet. 2005;365(9455):217-23.
8. Rahimi K, Emdin CA, MacMahon S. The epidemiology of blood pressure and its worldwide management. Circulation research. 2015;116(6):925-36.
9. National Institute of Population Research and Training Dhaka BMaAD, Bangladesh MEASURE DHS ICF International Calverton, Maryland, U.S.A. . BANGLADESH DEMOGRAPHIC AND HEALTH SURVEY 2011 [Available from: https://dhsprogram.com/pubs/pdf/fr265/fr265.pdf.
10. National Institute of Population Research and Training Medical Education and Family Welfare Division Ministry of Health and Family Welfare Dhaka BTDPIR, Maryland, USA. Bangladesh Demographic and Health Survey 2017-18 October 2020 [Available from: https://dhsprogram.com/pubs/pdf/FR344/FR344.pdf.
11. Razzaque A, Nahar L, Mustafa AHMG, Ahsan KZ, Islam MS, Yunus MJAPJoPH. Sociodemographic differentials of selected noncommunicable diseases risk factors among adults in Matlab, Bangladesh: findings from a WHO STEPS survey. 2011;23(2):183-91.
12. Tareque MI, Koshio A, Tiedt AD, Hasegawa TJPo. Are the rates of hypertension and diabetes higher in people from lower socioeconomic status in Bangladesh? Results from a nationally representative survey. 2015;10(5):e0127954.
13. Khan MN, Oldroyd JC, Chowdhury EK, Hossain MB, Rana J, Renzetti S, Islam RM. Prevalence, awareness, treatment, and control of hypertension in Bangladesh: Findings from National Demographic and Health Survey, 2017-2018. Journal of clinical hypertension (Greenwich, Conn). 2021;23(10):1830-42.
14. Azeez O, Kulkarni A, Kuklina EV, Kim SY, Cox S. Hypertension and Diabetes in Non-Pregnant Women of Reproductive Age in the United States. Preventing chronic disease. 2019;16:E146.
15. Tuoyire DA, Ayetey H. Gender differences in the association between marital status and hypertension in Ghana. Journal of biosocial science. 2019;51(3):313-34.
16. Segawa HK, Uematsu H, Dorji N, Wangdi U, Dorjee C, Yangchen P, et al. Gender with marital status, cultural differences, and vulnerability to hypertension: Findings from the national survey for noncommunicable disease risk factors and mental health using WHO STEPS in Bhutan. Plos one. 2021;16(8):e0256811.
17. Son M, Heo YJ, Hyun H-J, Kwak HJ. Effects of Marital Status and Income on Hypertension: The Korean Genome and Epidemiology Study (KoGES). Journal of Preventive Medicine and Public Health. 2022;55(6):506.
18. Holt-Lunstad J, Birmingham W, Jones BQ. Is there something unique about marriage? The relative impact of marital status, relationship quality, and network social support on ambulatory blood pressure and mental health. Annals of behavioral medicine. 2008;35(2):239-44.
19. Hong T, Xie S, Liu X, Wu J, Chen G. Do Machine Learning Approaches Perform Better Than Regression Models in Mapping Studies? A Systematic Review. Value in Health. 2025;28(5):800-11.
20. Saxena A, Sharma S, Kumar Johari P, Pandey A, Kumar S. A fair and interpretable deep learning approach for healthcare access prediction in underserved communities. Discover Artificial Intelligence. 2025;5(1):185.

21. Shen S, Qi W, Li S, Zeng J, Liu X, Zhu X, et al. Mapping the landscape of machine learning in chronic disease management: A comprehensive bibliometric study. Digit Health. 2025;11:20552076251361614.

22. Dhanda SS, Panwar D, Lin C-C, Sharma TK, Rastogi D, Bindewari S, et al. Advancement in public health through machine learning: a narrative review of opportunities and ethical considerations. Journal of Big Data. 2025;12(1):154.

23. El-Geneedy M, El-Din Moustafa H, Khater H, Abd-Elsamee S, Gamel SA. A comprehensive explainable AI approach for enhancing transparency and interpretability in stroke prediction. Scientific Reports. 2025;15(1):26048.

24. Kumar D, Balraj K, Seth S, Vashista S, Ramteke M, Rathore AS. An improved machine learning-based prediction framework for early detection of events in heart failure patients using mHealth. Health and Technology. 2024;14(3):495-512.

25. Lepakshi VA. Chapter 18 - Machine Learning and Deep Learning based AI Tools for Development of Diagnostic Tools. In: Parihar A, Khan R, Kumar A, Kaushik AK, Gohel H, editors. Computational Approaches for Novel Therapeutic and Diagnostic Designing to Mitigate SARS-CoV-2 Infection: Academic Press; 2022. p. 399-420.

26. Mancia G, Cappuccio FP, Burnier M, Coca A, Persu A, Borghi C, et al. Perspectives on improving blood pressure control to reduce the clinical and economic burden of hypertension. J Intern Med. 2023;294(3):251-68.

27. Tsoi K, Yiu K, Lee H, Cheng HM, Wang TD, Tay JC, et al. Applications of artificial intelligence for hypertension management. Journal of clinical hypertension (Greenwich, Conn). 2021;23(3):568-74.

28. Chaikijurajai T, Laffin LJ, Tang WHW. Artificial Intelligence and Hypertension: Recent Advances and Future Outlook. American Journal of Hypertension. 2020;33(11):967-74.

29. Silva GFS, Fagundes TP, Teixeira BC, Chiavegatto Filho ADP. Machine Learning for Hypertension Prediction: a Systematic Review. Current Hypertension Reports. 2022;24(11):523-33.

30. Padmanabhan S, Tran TQB, Dominiczak AF. Artificial Intelligence in Hypertension. Circulation Research. 2021;128(7):1100-18.

31. Hasselström J, Zarrinkoub R, Holmquist C, Hjerpe P, Ljungman C, Qvarnström M, et al. The Swedish Primary Care Cardiovascular Database (SPCCD): 74 751 hypertensive primary care patients. Blood Pressure. 2014;23(2):116-25.

32. Ji W, Zhang Y, Cheng Y, Wang Y, Zhou Y. Development and validation of prediction models for hypertension risks: A cross-sectional study based on 4,287,407 participants. Front Cardiovasc Med. 2022;9:928948.

33. Tayefi M, Esmaeili H, Saberi Karimian M, Amirabadi Zadeh A, Ebrahimi M, Safarian M, et al. The application of a decision tree to establish the parameters associated with hypertension. Comput Methods Programs Biomed. 2017;139:83-91.

34. Islam MM, Alam MJ, Maniruzzaman M, Ahmed N, Ali MS, Rahman MJ, Roy DC. Predicting the risk of hypertension using machine learning algorithms: A cross sectional study in Ethiopia. PLoS One. 2023;18(8):e0289613.

35. Chowdhury MZI, Leung AA, Walker RL, Sikdar KC, O'Beirne M, Quan H, Turin TC. A comparison of machine learning algorithms and traditional regression-based statistical modeling for predicting hypertension incidence in a Canadian population. Scientific Reports. 2023;13(1):13.

36. Aish M, Ghafoor A, Nasim F, Ali K, Akhter S, Azeem S. Improving Stroke Prediction Accuracy through Machine Learning and Synthetic Minority Over-sampling. Journal of Computing & Biomedical Informatics. 2024;07.

37. Schjerven FE, Ingeström EML, Steinsland I, Lindseth F. Development of risk models of incident hypertension using machine learning on the HUNT study data. Scientific Reports. 2024;14(1):5609.

38. Chang W, Liu Y, Xiao Y, Yuan X, Xu X, Zhang S, Zhou S. A machine-learning-based prediction method for hypertension outcomes based on medical data. Diagnostics. 2019;9(4):178.
39. Du Z, Yang Y, Zheng J, Li Q, Lin D, Li Y, et al. Accurate Prediction of Coronary Heart Disease for Patients With Hypertension From Electronic Health Records With Big Data and Machine-Learning Methods: Model Development and Performance Evaluation. JMIR Med Inform. 2020;8(7):e17257.
40. Kourou K, Exarchos TP, Exarchos KP, Karamouzis MV, Fotiadis DI. Machine learning applications in cancer prognosis and prediction. Comput Struct Biotechnol J. 2015;13:8-17.
41. Selya AS, Anshutz D. Machine learning for the classification of obesity from dietary and physical activity patterns. Advanced Data Analytics in Health. 2018:77-97.
42. Panaretos D, Koloverou E, Dimopoulos AC, Kouli G-M, Vamvakari M, Tzavelas G, et al. A comparison of statistical and machine-learning techniques in evaluating the association between dietary patterns and 10-year cardiometabolic risk (2002–2012): the ATTICA study. British Journal of Nutrition. 2018;120(3):326-34.
43. Premsagar P, Aldous C, Esterhuizen TM, Gomes BJ, Gaskell JW, Tabb DL. Comparing conventional statistical models and machine learning in a small cohort of South African cardiac patients. Informatics in Medicine Unlocked. 2022;34:101103.
44. Hu P, Li Y, Liu Y, Guo G, Gao X, Su Z, et al. Comparison of Conventional Logistic Regression and Machine Learning Methods for Predicting Delayed Cerebral Ischemia After Aneurysmal Subarachnoid Hemorrhage: A Multicentric Observational Cohort Study. Front Aging Neurosci. 2022;14:857521.
45. Jerez JM, Molina I, García-Laencina PJ, Alba E, Ribelles N, Martín M, Franco L. Missing data imputation using statistical and machine learning methods in a real breast cancer problem. Artificial Intelligence in Medicine. 2010;50(2):105-15.
46. Xing Y, Wang J, Zhao Z, Gao a. Combination Data Mining Methods with New Medical Data to Predicting Outcome of Coronary Heart Disease. Convergence Information Technology, International Conference on. 2007;0:868-72.
47. Ture M, Omurlu I, Kurum A, Ozdamar K. Comparing classification techniques for predicting essential hypertension. Expert Systems with Applications. 2005;29:583-8.
48. Wu X, Yuan X, Wang W, Liu K, Qin Y, Sun X, et al. Value of a machine learning approach for predicting clinical outcomes in young patients with hypertension. Hypertension. 2020;75(5):1271-8.
49. Assi S, Jayabalan M, Parakh V, Assi J, Al Hamid A, Obe DA-J. Predicting Incidence of Stroke via Supervised Machine Learning Methods on Class Imbalanced Data. Non-Invasive Health Systems based on Advanced Biomedical Signal and Image Processing: CRC Press; 2024. p. 128-44.
50. Ijaz MF, Alfian G, Syafrudin M, Rhee J. Hybrid Prediction Model for Type 2 Diabetes and Hypertension Using DBSCAN-Based Outlier Detection, Synthetic Minority Over Sampling Technique (SMOTE), and Random Forest. Applied Sciences. 2018;8(8):1325.
51. Alsmariy R, Healy G, Abdelhafez H. Predicting cervical cancer using machine learning methods. International Journal of Advanced Computer Science and Applications. 2020;11(7).
52. Sihag G, Yadav P, Vijay V, Delcroix V, Siebert X, Yadav SK, Puisieux F, editors. Advantages of oversampling techniques: a case study in risk factors for fall prediction. International Conference on Information and Communication Technologies for Ageing Well and e-Health; 2021: Springer.
53. Muraru MM, Simó Z, Iantovics LB. Cervical Cancer Prediction based on Imbalanced Data using Machine Learning Algorithms. Preprints: Preprints; 2024.
54. Chai SS, Goh KL, Cheah WL, Chang YHR, Ng GW. Hypertension Prediction in Adolescents Using Anthropometric Measurements: Do Machine Learning Models Perform Equally Well? Applied Sciences. 2022;12(3):1600.
55. Shuja M, Mittal S, Zaman M. Effective Prediction of Type II Diabetes Mellitus Using Data Mining Classifiers and SMOTE. 2020. p. 195-211.

56. Wang K, Tian J, Zheng C, Yang H, Ren J, Li C, et al. Improving risk identification of adverse outcomes in chronic heart failure using SMOTE+ ENN and machine learning. Risk management and healthcare policy. 2021:2453-63.
57. Zheng H, Sherazi SWA, Lee JY. A cost-sensitive deep neural network-based prediction model for the mortality in acute myocardial infarction patients with hypertension on imbalanced data. Frontiers in Cardiovascular Medicine. 2024;11.
58. Matondang N, Surantha N. Effects of oversampling SMOTE in the classification of hypertensive dataset. Adv Sci Technol Eng Syst. 2020;5(4).
59. Huang X, Cao T, Chen L, Wu H, Li J, Tan Z, et al. Predicting Stroke Risk in a Chinese Hypertensive Population Using Machine Learning. 2021.
60. Alabdallah A, Pashami S, Rögnvaldsson T, Ohlsson M, editors. SurvSHAP: a proxy-based algorithm for explaining survival models with SHAP. 2022 IEEE 9th international conference on data science and advanced analytics (DSAA); 2022: IEEE.
61. Cakiroglu C, Demir S, Hakan Ozdemir M, Latif Aylak B, Sariisik G, Abualigah L. Data-driven interpretable ensemble learning methods for the prediction of wind turbine power incorporating SHAP analysis. Expert Systems with Applications. 2024;237:121464.
62. Luo H, Xiang C, Zeng L, Li S, Mei X, Xiong L, et al. SHAP based predictive modeling for 1 year all-cause readmission risk in elderly heart failure patients: feature selection and model interpretation. Scientific Reports. 2024;14(1):17728.
63. Xiaomao X, Xudong Z, Yuanfang W. A Comparison of Feature Selection Methodology for Solving Classification Problems in Finance. Journal of Physics: Conference Series. 2019;1284(1):012026.
64. Raufi B, Longo L. Comparing ANOVA and PowerShap Feature Selection Methods via Shapley Additive Explanations of Models of Mental Workload Built with the Theta and Alpha EEG Band Ratios. BioMedInformatics. 2024;4(1):853-76.
65. Ejiyi CJ, Qin Z, Ukwuoma CC, Nneji GU, Monday HN, Ejiyi MB, et al. Comparative performance analysis of Boruta, SHAP, and Borutashap for disease diagnosis: A study with multiple machine learning algorithms. Network: Computation in Neural Systems.1-38.
66. Asadi R, Khattak A, Vashani H, Almujibah HR, Rabie H, Asadi S, Dimitrijevic B. Self-Paced Ensemble-SHAP Approach for the Classification and Interpretation of Crash Severity in Work Zone Areas. Sustainability. 2023;15(11):9076.
67. Tran VQ, Byeon H. Predicting dementia in Parkinson's disease on a small tabular dataset using hybrid LightGBM–TabPFN and SHAP. DIGITAL HEALTH. 2024;10:20552076241272585.
68. Amin P. Feature Importance in Predicting Clinical Outcome: Statistics vs. Explainable Artificial Intelligence. bioRxiv. 2024:2024.07. 21.604467.
69. Lee Y, Kim K, Seo J. CLE-SH: Comprehensive Literal Explanation package for SHapley values by statistical validity. arXiv preprint arXiv:240912578. 2024.
70. Asadullah M, Hossain MM, Rahaman S, Amin MS, Sumy MSA, Parh MYA, Hossain MA. Evaluation of machine learning techniques for hypertension risk prediction based on medical data in Bangladesh. Indonesian Journal of Electrical Engineering and Computer Science. 2023;31(3):1794-802.
71. Islam M, Alam J, Kumar S, Islam A, Khan MR, Rabby S, et al. Development and validation of a nomogram model for predicting the risk of hypertension in Bangladesh. Heliyon. 2024;10(22).
72. Islam MM, Rahman MJ, Chandra Roy D, Tawabunnahar M, Jahan R, Ahmed N, Maniruzzaman M. Machine learning algorithm for characterizing risks of hypertension, at an early stage in Bangladesh. Diabetes Metab Syndr. 2021;15(3):877-84.
73. Islam SMS, Talukder A, Awal MA, Siddiqui MMU, Ahamad MM, Ahammed B, et al. Machine Learning Approaches for Predicting Hypertension and Its Associated Factors Using Population-Level Data From Three South Asian Countries. Front Cardiovasc Med. 2022;9:839379.

74. Parvin S, Akter S, Hossain MI, Ali MS, Soni MSM. Residential variations in hypertension prevalence and trends among adults in Bangladesh. Research in Health Services & Regions. 2024;3(1):3.
75. Siddiquee T. Machine Learning Approaches for Estimating Prevalence of Undiagnosed Hypertension among Bangladeshi Adults: Evidence from a Nationwide Survey.
76. Ghosh PK, Islam MA, Haque MA, Tariqujjaman M, Das NC, Ali M, et al. Identifying predictors and assessing causal effect on hypertension risk among adults using Double Machine Learning models: Insights from Bangladesh Demographic and Health Survey. PLoS Comput Biol. 2025;21(7):e1013211.
77. Silvey S, Liu J. Sample Size Requirements for Popular Classification Algorithms in Tabular Clinical Data: Empirical Study. J Med Internet Res. 2024;26:e60231.
78. Baeza-Delgado C, Cerdá Alberich L, Carot-Sierra JM, Veiga-Canuto D, Martínez de las Heras B, Raza B, Martí-Bonmatí L. A practical solution to estimate the sample size required for clinical prediction models generated from observational research on data. European Radiology Experimental. 2022;6(1):22.
79. Infante G, Miceli R, Ambrogi F. Sample size and predictive performance of machine learning methods with survival data: A simulation study. Statistics in Medicine. 2023;42(30):5657-75.
80. Al Kibria GM, Burrowes V, Choudhury A, Sharmeen A, Swasey KJIJoCH. Sex differences in prevalence and associated factors of prehypertension and hypertension among Bangladeshi adults. 2019;1:100006.
81. Chowdhury MAB, Islam M, Rahman J, Uddin MT, Haque MR, Uddin MJJPo. Changes in prevalence and risk factors of hypertension among adults in Bangladesh: An analysis of two waves of nationally representative surveys. 2021;16(12):e0259507.
82. Hossain M, Khan M, Ababneh F, Shaw JJBph. Identifying factors influencing contraceptive use in Bangladesh: evidence from BDHS 2014 data. 2018;18(1):1-14.
83. Khan MN, Islam MM, Islam RM. Association Between Contraception Use, Diabetes and Hypertension: Findings from Bangladesh Demographic and Health Survey. 2022.
84. Hossain MB, Khan MN, Oldroyd JC, Rana J, Magliago DJ, Chowdhury EK, et al. Prevalence of, and risk factors for, diabetes and prediabetes in Bangladesh: Evidence from the national survey using a multilevel Poisson regression model with a robust variance. 2022;2(6):e0000461.
85. Islam R, Khan M, Oldroyd J, Rana J, Chowdhury E, Karim M, Hossain MJM. Prevalence of diabetes and prediabetes among Bangladeshi adults and associated factors: Evidence from the Demographic and Health Survey, 2017-18. 2021.
86. Organization WH. Definition and diagnosis of diabetes mellitus and intermediate hyperglycaemia: report of a WHO/IDF consultation. 2006.
87. Zong X, Wang H, Yang L, Guo Y, Zhao M, Magnussen CG, Xi B. Maternal Pre-pregnancy Body Mass Index Categories and Infant Birth Outcomes: A Population-Based Study of 9 Million Mother-Infant Pairs. Front Nutr. 2022;9:789833.
88. Lemoine A, Tounian P. Childhood anemia and iron deficiency in sub-Saharan Africa - risk factors and prevention: A review. Arch Pediatr. 2020;27(8):490-6.
89. Aksoy S, Haralick RM. Feature normalization and likelihood-based similarity measures for image retrieval. Pattern recognition letters. 2001;22(5):563-82.
90. Byeon HJIJoACS, Applications. Development of a physical impairment prediction model for Korean elderly people using synthetic minority over-sampling technique and XGBoost. 2021;12(1).
91. Byeon HJIJoACS, Applications. Predicting the depression of the South Korean elderly using SMOTE and an imbalanced binary dataset. 2021;12(1).
92. Hanafy M, Ming RJIJoACS, Applications. Improving imbalanced data classification in auto insurance by the data level approaches. 2021;12(6).
93. Geurts P, Ernst D, Wehenkel L. Extremely randomized trees. Machine Learning. 2006;63(1):3-42.
94. Biau G, Scornet E. A random forest guided tour. TEST. 2016;25(2):197-227.

95. Touw WG, Bayjanov JR, Overmars L, Backus L, Boekhorst J, Wels M, van Hijum SAFT. Data mining in the Life Sciences with Random Forest: a walk in the park or lost in the jungle? Briefings in Bioinformatics. 2012;14(3):315-26.
96. Bergstra J, Bengio YJJomlr. Random search for hyper-parameter optimization. 2012;13(2).
97. Sifat IK, Kibria MK. Optimizing hypertension prediction using ensemble learning approaches. PLoS One. 2024;19(12):e0315865.
98. Sanaudi R, Zakaria ZA, Khairulisam AA, Ibrahim N, Ul Saufie AZ. Unveil the Features Influencing Hypertension Adults in Malaysia using Machine Learning Models. Malaysian Journal of Medicine & Health Sciences. 2024;20(6).
99. Seo J-W, Lee S, Yim MH. Machine Learning Approach for Predicting Hypertension Based on Body Composition in South Korean Adults. Bioengineering. 2024;11(9):921.
100. Tanaka M, Akiyama Y, Mori K, Hosaka I, Endo K, Ogawa T, et al. Machine learning-based analyses of contributing factors for the development of hypertension: a comparative study. Clinical and Experimental Hypertension. 2025;47(1):2449613.
101. Angelaki E, Barmparis GD, Fragkiadakis K, Maragkoudakis S, Zacharis E, Plevritaki A, et al. Diagnostic performance of single-lead electrocardiograms for arterial hypertension diagnosis: a machine learning approach. Journal of human hypertension. 2025;39(1):58-65.
102. Leha A, Hellenkamp K, Unsöld B, Mushemi-Blake S, Shah AM, Hasenfuß G, Seidler T. A machine learning approach for the prediction of pulmonary hypertension. PloS one. 2019;14(10):e0224453.
103. Donmez TB, Kutlu M. Explainable quantum-enhanced machine learning for hypertension prediction. The European Physical Journal Special Topics. 2025:1-13.
104. Saito T, Rehmsmeier MJPo. The precision-recall plot is more informative than the ROC plot when evaluating binary classifiers on imbalanced datasets. 2015;10(3):e0118432.
105. Akosa JS, editor Predictive Accuracy : A Misleading Performance Measure for Highly Imbalanced Data2017.
106. Alam TM, Shaukat K, Hameed IA, Luo S, Sarwar MU, Shabbir S, et al. An investigation of credit card default prediction in the imbalanced datasets. 2020;8:201173-98.
107. Delgado R, Tibau X-AJPo. Why Cohen's Kappa should be avoided as performance measure in classification. 2019;14(9):e0222916.
108. Jeni LA, Cohn JF, De La Torre F, editors. Facing imbalanced data--recommendations for the use of performance metrics. 2013 Humaine association conference on affective computing and intelligent interaction; 2013: IEEE.
109. Fitriyani NL, Syafrudin M, Alfian G, Yang Ck, Rhee J, Ulyah SM, editors. Chronic Disease Prediction Model Using Integration of DBSCAN, SMOTE-ENN, and Random Forest. 2022 ASU International Conference in Emerging Technologies for Sustainability and Intelligent Systems (ICETSIS); 2022 22-23 June 2022.
110. Muntasir Nishat M, Faisal F, Jahan Ratul I, Al-Monsur A, Ar-Rafi AM, Nasrullah SM, et al. A Comprehensive Investigation of the Performances of Different Machine Learning Classifiers with SMOTE-ENN Oversampling Technique and Hyperparameter Optimization for Imbalanced Heart Failure Dataset. Scientific Programming. 2022;2022:3649406.
111. Parthasarathy S, Jayaraman V, R JPP, editors. Predicting Heart Failure using SMOTE-ENN-XGBoost. 2023 International Conference on Intelligent Data Communication Technologies and Internet of Things (IDCIoT); 2023 5-7 Jan. 2023.
112. Tang J, Wang X, Wan H, Lin C, Shao Z, Chang Y, et al. Joint modeling strategy for using electronic medical records data to build machine learning models: an example of intracerebral hemorrhage. BMC Medical Informatics and Decision Making. 2022;22(1):278.

113. Ullah Z, Saleem F, Jamjoom M, Fakieh B, Kateb F, Ali AM, Shah B. Detecting High-Risk Factors and Early Diagnosis of Diabetes Using Machine Learning Methods. Computational Intelligence and Neuroscience. 2022;2022:2557795.

114. Ahammed B, Maniruzzaman M, Talukder A, Ferdausi F. Prevalence and risk factors of hypertension among young adults in Albania. High Blood Pressure & Cardiovascular Prevention. 2021;28:35-48.

115. Weare AR, Feng Z, McGrath N. The prevalence of hypertension and hypertension control among married Namibian couples. Plos one. 2023;18(8):e0289788.

116. Khan MN, Islam MM, Islam RM. Pattern of contraceptive use among reproductive-aged women with diabetes and/or hypertension: findings from Bangladesh Demographic and Health Survey. BMC Women's Health. 2022;22(1):230.

117. Bista B, Dhungana RR, Chalise B, Pandey AR. Prevalence and determinants of non-communicable diseases risk factors among reproductive aged women of Nepal: Results from Nepal Demographic Health Survey 2016. PloS one. 2020;15(3):e0218840.

118. Morvaridi M, Rayyani E, Jaafari M, Khiabani A, Rahimlou M. The effect of green coffee extract supplementation on cardio metabolic risk factors: a systematic review and meta-analysis of randomized controlled trials. J Diabetes Metab Disord. 2020;19(1):645-60.

119. Rahimlou M, Grau N, Banaie-Jahromi N, Taheri M, Khosravi A, Mavrommatis Y, Mohammadifard N. Association of adherence to the dietary approach to stop hypertension and Mediterranean diets with blood pressure in a non-hypertensive population: Results from Isfahan Salt Study (ISS). Nutr Metab Cardiovasc Dis. 2022;32(1):109-16.

Supporting Information

S1 Appendix: List of class balancing techniques

S2 Appendix: Evaluation of machine learning algorithms

S3 Appendix: Evaluation of machine learning algorithms

S4 Appendix: Supplementary Tables

S1 Fig.: Learning curve of the ExtraTrees model for predicting hypertension among married women in Bangladesh.

S2 Fig.: Reliability plot of the ExtraTrees model for predicting hypertension among married women in Bangladesh.

Figure

[Click here to access/download;Figure;Revise_Figures_PACE.docx]

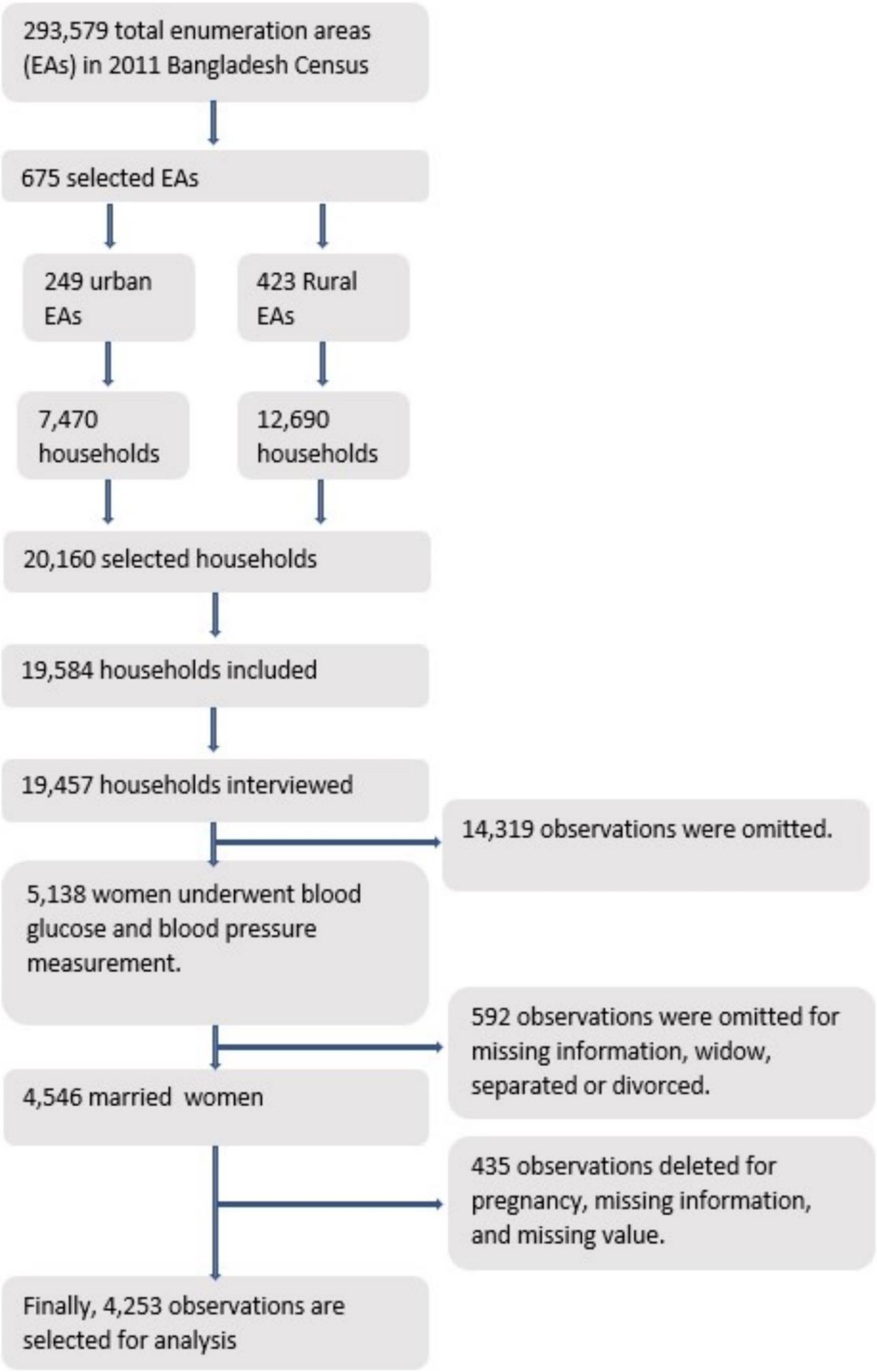

Fig.1: Data selection flow chart

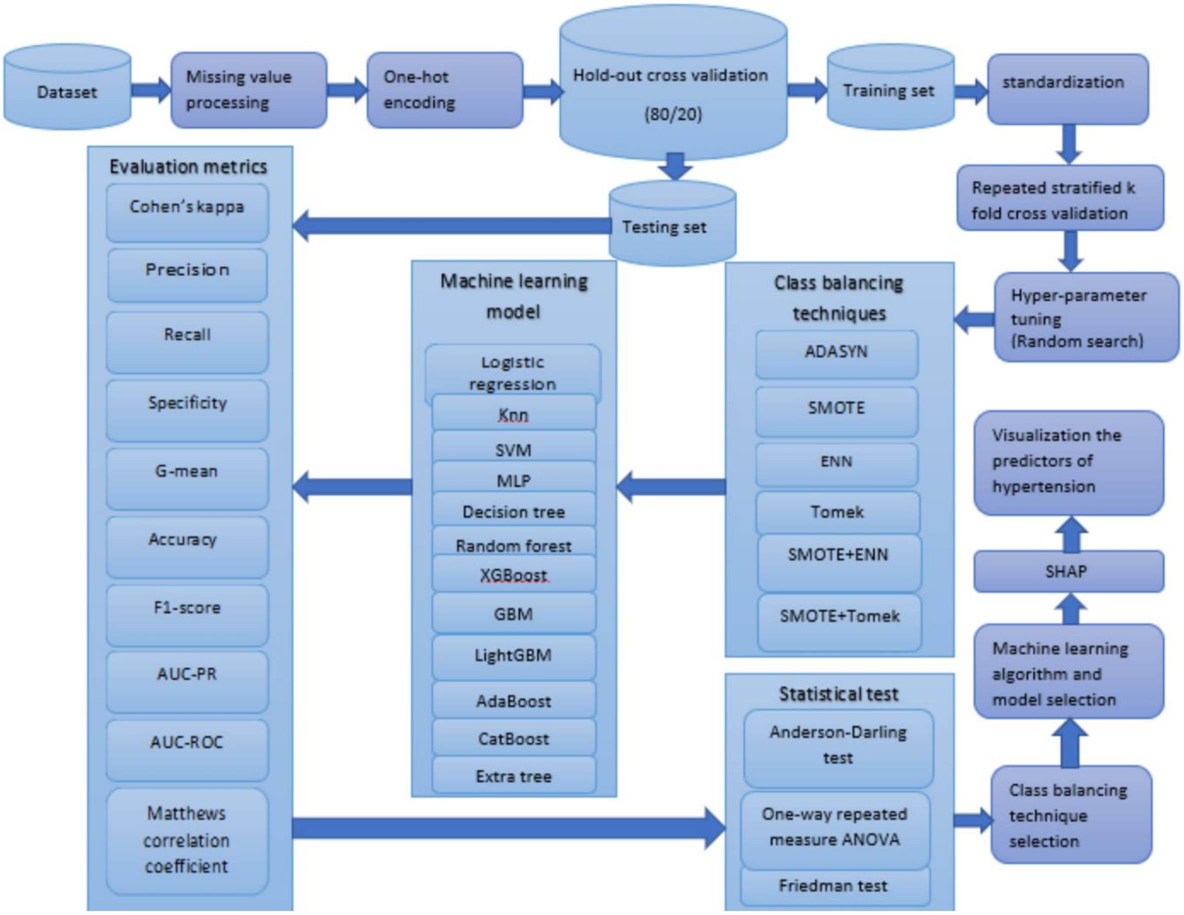

Fig 2. Workflow of machine learning pipeline for hypertension prediction and risk factor ranking of married women in Bangladesh.

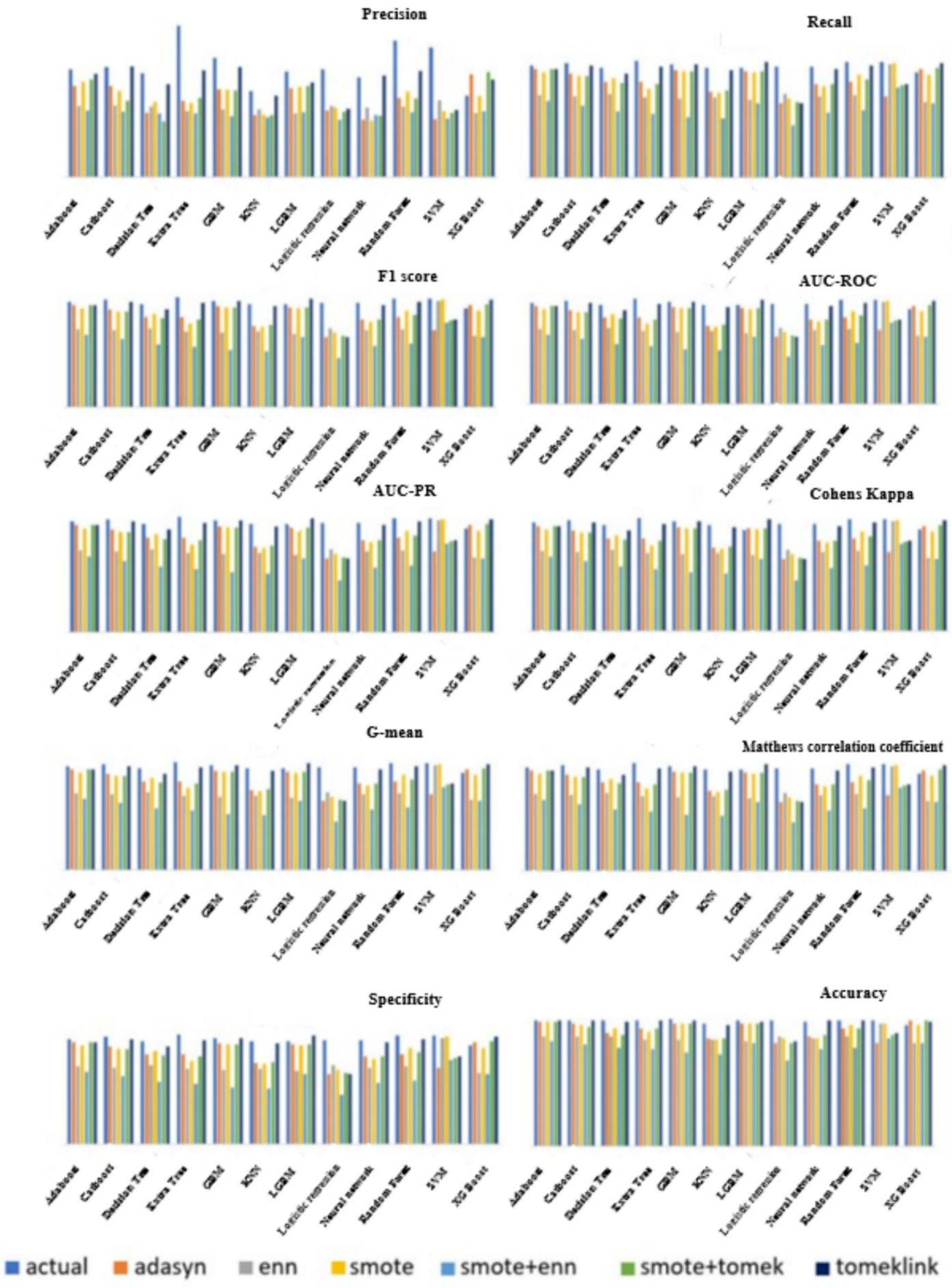

Fig 3: Performance of the machine learning algorithms.

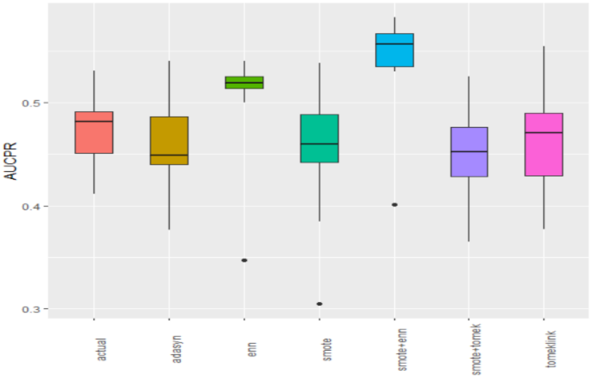

Fig 4. The Boxplot of the original data and class balancing techniques based on AUC-PR.

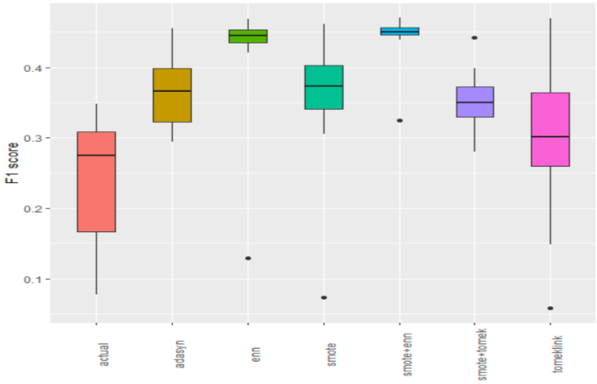

Fig 5. The Boxplot of the original data and class balancing techniques based on F1 scores.

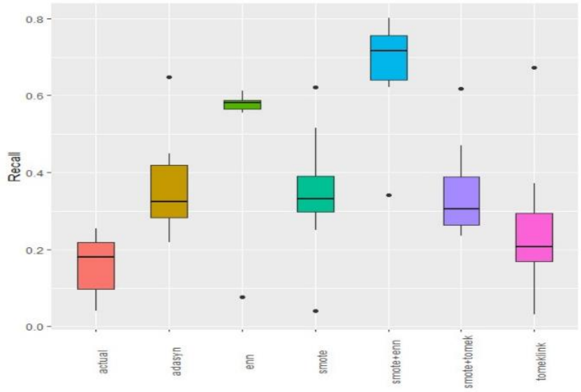

Fig 6. The Boxplot of the original data and class balancing techniques based on Recall.

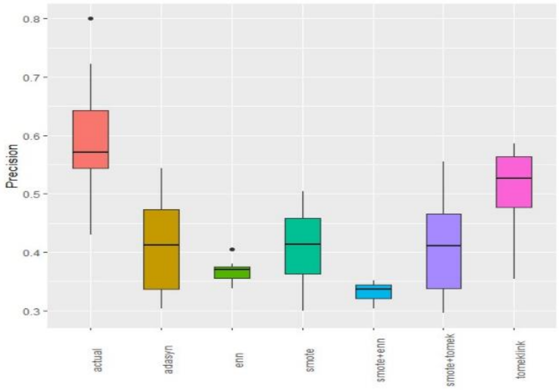

Fig 7. The Boxplot of the original data and class balancing techniques based on Precision.

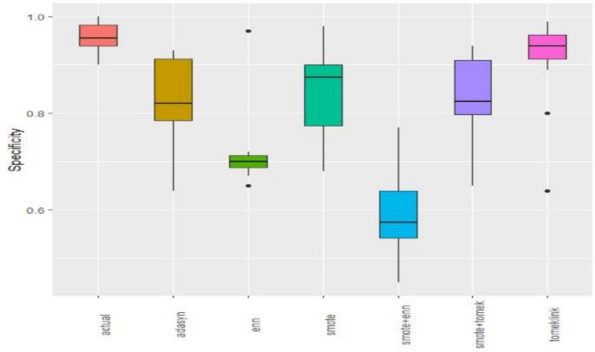

Fig 8. The Boxplot of the original data and class balancing techniques based on Specificity.

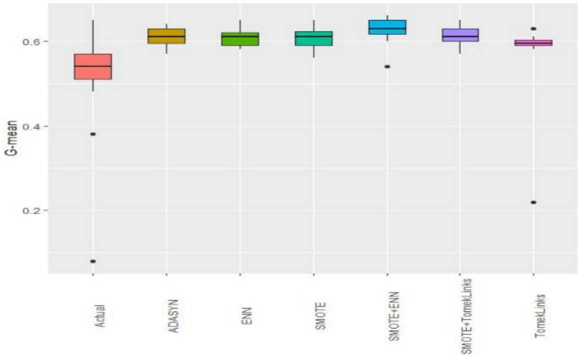

Fig 9. The Boxplot of the original data and class balancing techniques based on G-mean.

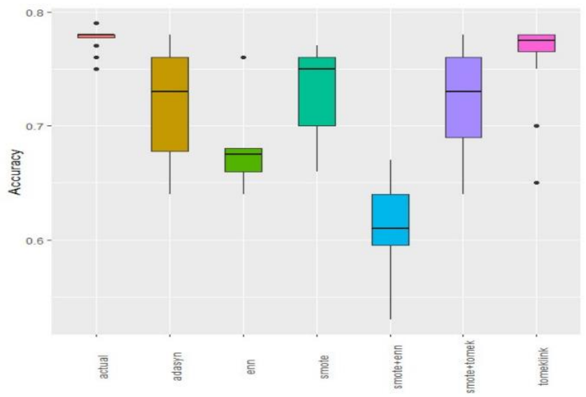

Fig 10. The Boxplot of the original data and class balancing techniques based on Accuracy.

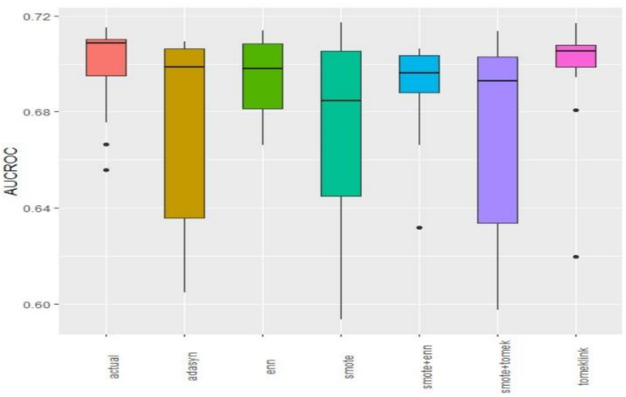

Fig 11. The Boxplot of the original data and class balancing techniques based on AUCROC.

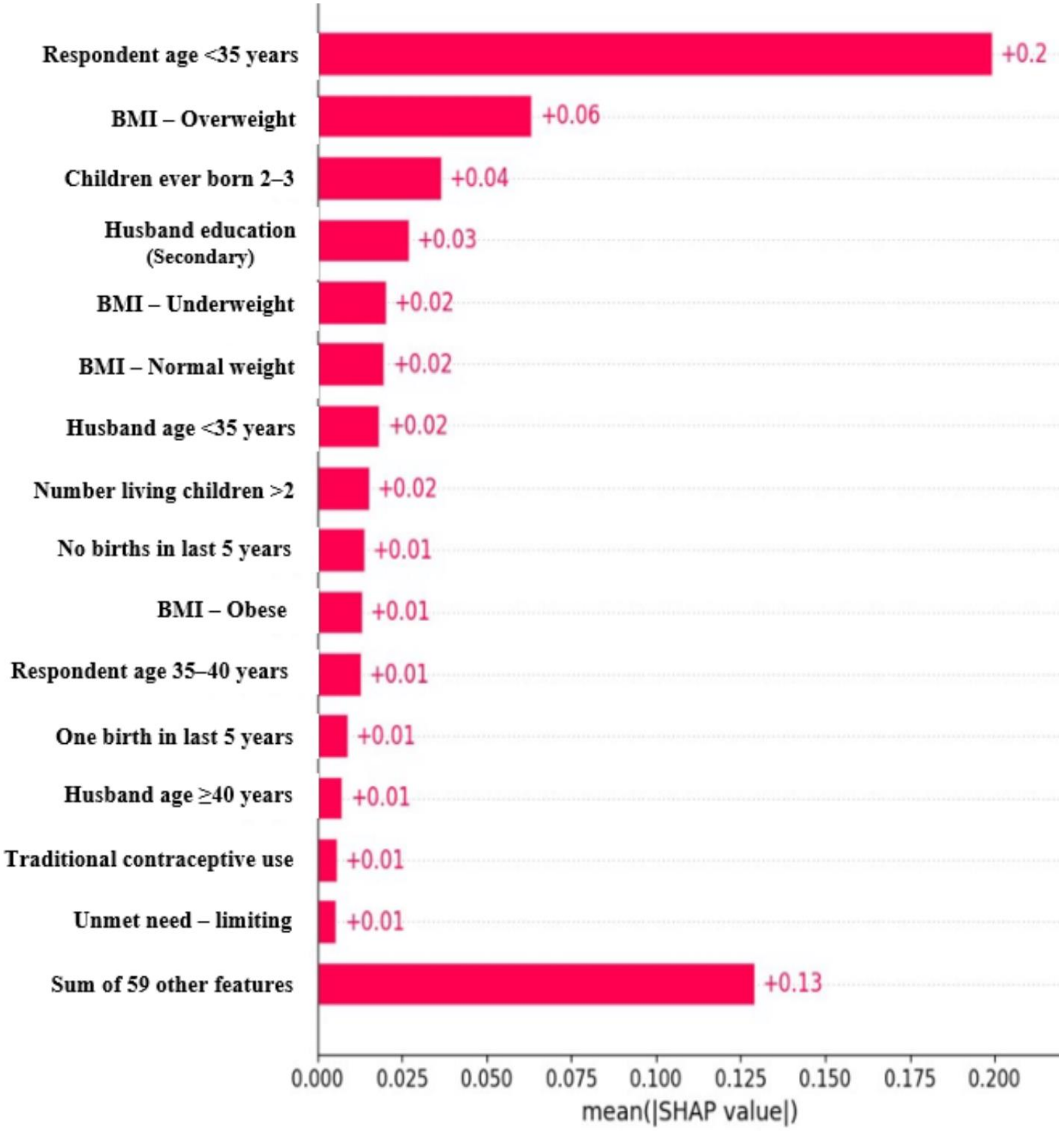

Fig 12a. The factors importance of hypertension among married women in Bangladesh.

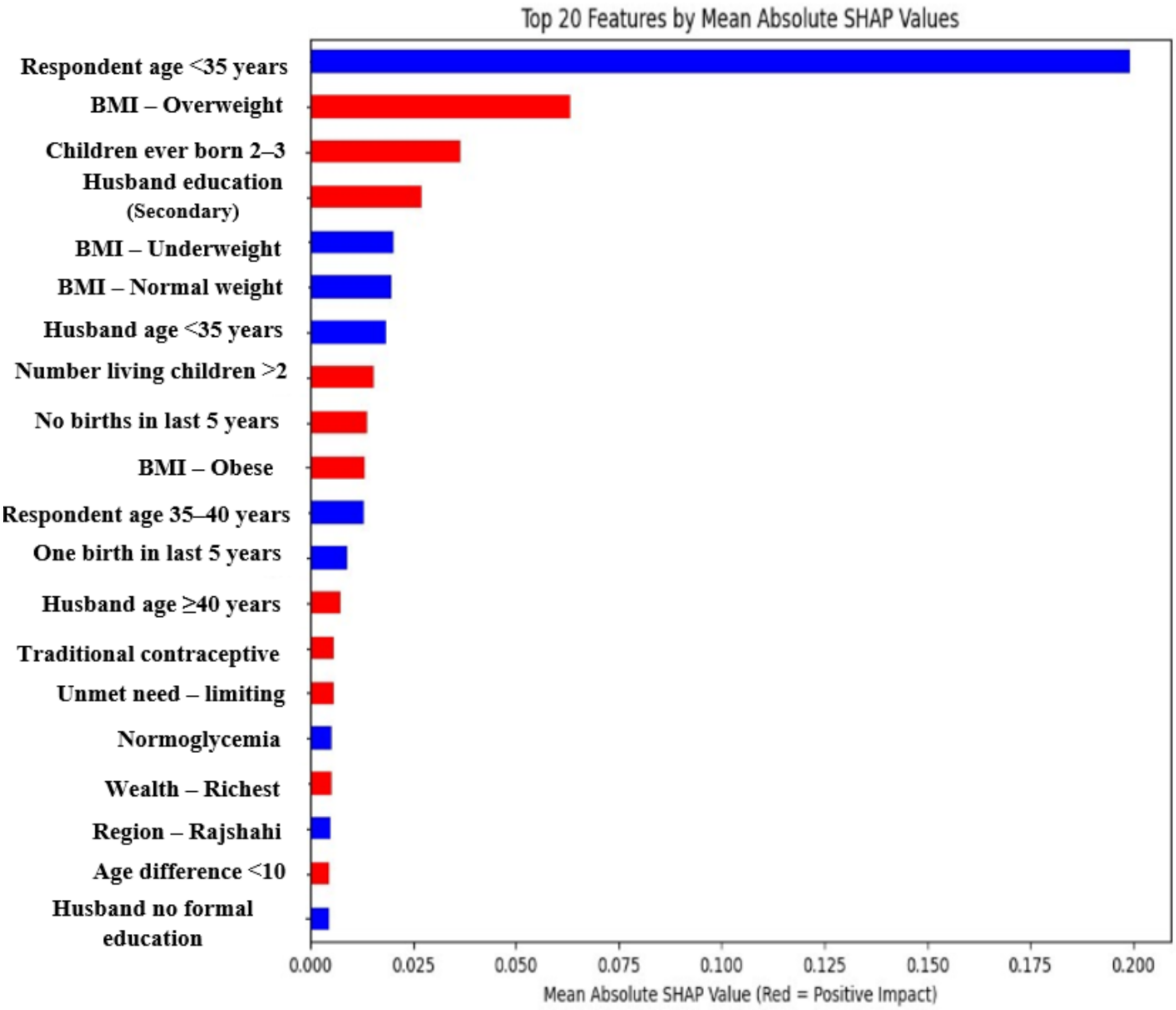

Fig. 12b. The classification of positive and negative predictors of hypertension of married women in Bangladesh

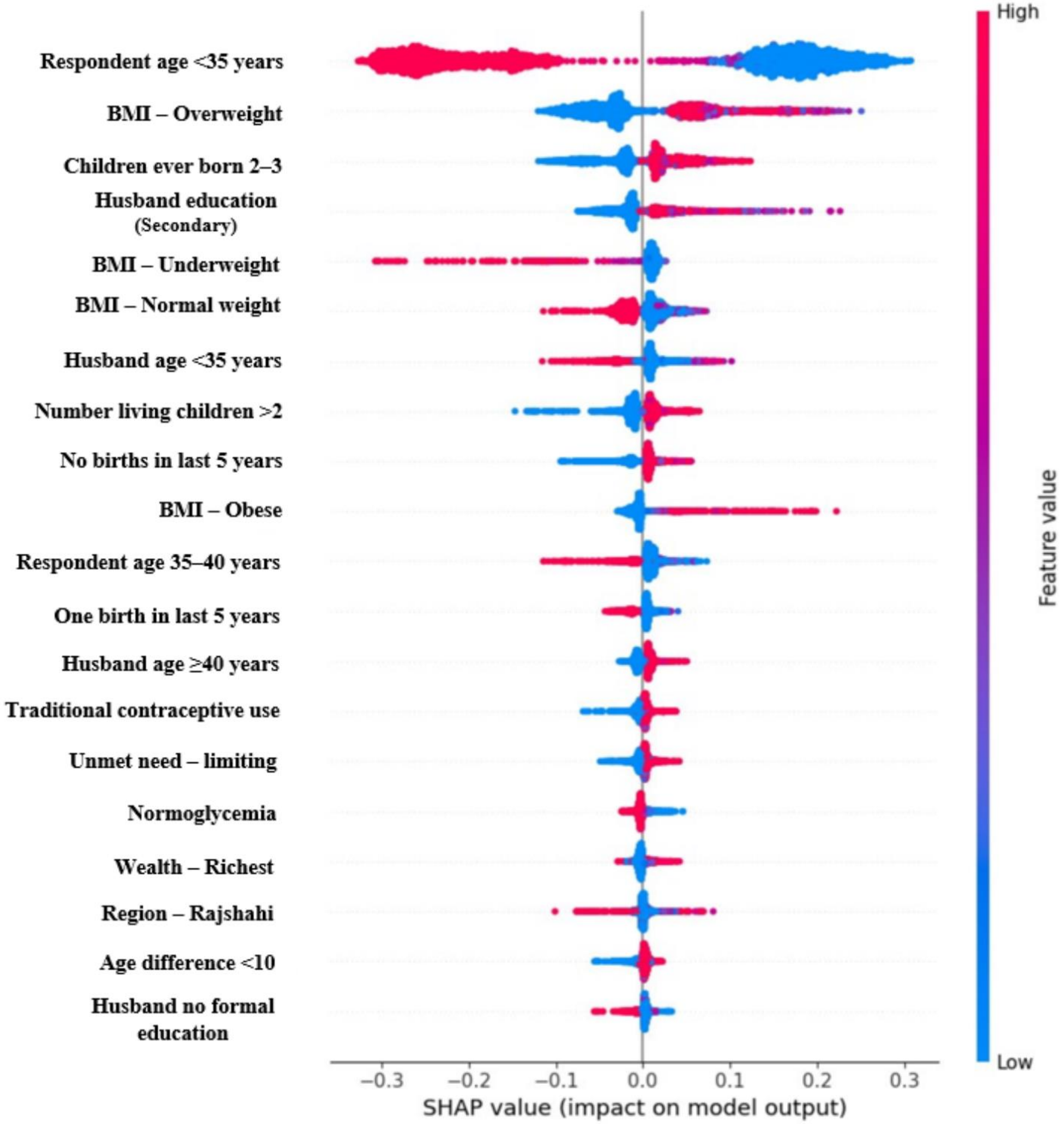

Fig 12c. The impact of the factors of married women's hypertension in Bangladesh.

Click here to access/download
Supporting Information
S1 Appendix.docx

Click here to access/download
Supporting Information
S2 Appendix.docx

Click here to access/download
Supporting Information
S3 Appendix.docx

Supplementary Figure 2

Click here to access/download
Supporting Information
S2 Fig.docx

Supplementary Tables

Click here to access/download
Supporting Information
S4 Appendix.docx

Supplementary Figure 1

Click here to access/download
Supporting Information
S1 Fig.docx

~~Evaluating machine learning algorithms~~Predicting Hypertension and ~~identifying factors associated with hypertension~~Identifying most important Factors among ~~married women~~Married Women in Bangladesh using Machine Learning Approach.

Novel Chandra Das[1*], Probir Kumar Ghosh[1], Md. Alamgir Hossain[1], Uddip Acharjee Shuvo[2], Nipa Rani Talukder[3], Fatema Khatun[1], Mohammad Ziaul Islam Chowdhury[4,5,6]

1. International Centre for Diarrhoeal Disease Research, Bangladesh
2. Institute of information technology, University of Dhaka, Bangladesh
3. Department of Computer Science and Engineering, North East University, Bangladesh
4. Department of Psychiatry, University of Calgary, 3280 Hospital Drive NW, Calgary, AB T2N 4Z6, Canada.
5. Provincial Research Data Services, Alberta Health Services, Alberta, Canada
6. Department of General Educational Development, Daffodil International University, Dhaka, Bangladesh.

*Corresponding author

E-mail: Novel.das@icddrb.org (NCD)

Formatted: Font: 14 pt, Font color: Text 1

Formatted: Heading 1, Justified

Formatted: Font: 14 pt, Font color: Text 1

Formatted: Font: 14 pt, Font color: Text 1

Formatted: Font: 14 pt, Font color: Text 1

Formatted: Justified

Abstract

Introduction: Hypertension is a ~~prevalent global health threat that intensifies the risk of various life-threatening~~ leading contributor to maternal and ~~newborn outcomes. we aimed to develop a machine learning model to identify and rank predictive factors of hypertension among married women~~ cardiometabolic morbidity in Bangladesh. We developed and interpreted machine-learning (ML) models to predict hypertension and rank associated factors among married women with the goal of informing targeted screening and policy in low-resource settings.

~~**Methodology**: The data was obtained from the Bangladesh Demographic and Health Survey, 2017–18, which included~~**Methods:** We analyzed 4,253 married women ~~in Bangladesh. To assess the predictive factors of~~ from the nationally representative BDHS 2017–18 survey (hypertension ~~among married women in Bangladesh, we employed 12 machine learning algorithms and 6 class-balancing techniques where each of the~~prevalence: 23.1%). Twelve ML algorithms were ~~evaluated by applying original and~~trained under six class-~~balanced dataset. We have used~~ balancing strategies with hyperparameters tuned via random search. Validation used a hold-out test set (80/20) and repeated stratified k-fold cross-validation~~ as a model validation technique. Furthermore, parameters~~; bootstrap confidence intervals were estimated for the selected ~~by hyper-parameter tuning utilizing random search optimization. To differentiate the predictive~~model. Model performance ~~of class-balanced technique, we used various~~ was compared with parametric and non-parametric ~~statistical~~ tests. ~~Eventually, we used~~To interpret results, SHAP ~~(SHapley Additive exPlanations) method~~ was used to ~~visualize the importance factors and impact of the individual factor on hypertension of married women.~~

Formatted: Left, Line spacing: Multiple 1.08 li

Formatted: Space Before: Auto, After: Auto

Formatted: Font: Bold

Formatted: Font color: Auto, Pattern: Clear

~~**Results**: In Bangladesh, 76.9% of married women had normal blood pressure, in contrast to 23.1% who had hypertension. The Extra tree algorithm showed an excellent improvement where F1 score increased from 8% to 94% for SMOTE+ENN class balanced technique. However,~~rank the top 20 ~~factors were screened using the model, revealing that 11 factors, most notably being overweight, having two or three children, and the husband or partner having attained secondary education are positively associated with hypertension. In contrast, 9 factors, including the respondent's current age below 35 years, being underweight or of normal weight, and having a husband or partner under 35 years of age, are negatively associated with hypertension~~predictors and visualize feature effects. Models quantify associations rather than causation.

Formatted: Space Before: Auto, After: Auto

Results: The Extra Trees classifier with SMOTE+ENN achieved the best discrimination (F1 = 0.94; AUC-PR = 0.95; ROC-AUC = 0.95). Compared with the original imbalanced training, minority-class detection improved substantially (Extra Trees F1 increased from 0.08 to 0.94; recall from 0.04 to 0.95) while accuracy and ROC-AUC remained relatively stable across samplers. Statistical testing favored SMOTE+ENN for recall, F1, G-mean and AUC-PR. SHAP identified age, parity, recent births, contraceptive use, spousal education and BMI as key predictors. Younger age (<35 years) and normal/underweight status were protective, while parity ≥2–3, husbands' age ≥40 years and overweight/obesity increased risk.

Conclusions: An interpretable ensemble model built primarily on sociodemographic and behavioral variables supplemented by limited biometric markers (BMI, glucose) can accurately flag hypertensive risk among married women in Bangladesh. Findings support programmatic integration of risk scores into eRegistries, routine blood pressure checks in family planning and postpartum visits, husband-focused education/SMS interventions and prioritization of high-parity

households in high-risk regions. External validation on BDHS-2022 is planned to assess generalizability.

Conclusion: Through a comprehensive evaluation, our study demonstrates that the Extra Tree algorithm, integrated with SMOTE+ENN class balancing, outperformed other models. Additionally, we identified key predictive factors associated with hypertension among married women in Bangladesh through the development of the model.

Keywords: Bangladesh, BDHS, Hypertension, machine learning, Tukey's HSD test, Friedman test, class balancedbalancing techniques, SHAP.

Introduction

Hypertension is a crucial risk factor formajor contributor to cardiovascular disease and chronic kidney disease which has a vital role in, two of the leading causes of death and disability worldwide deaths and dysfunction(1-34). RoughlyGlobally, an estimated 1.13 billion people worldwide were diagnosed with hypertension,are affected with 66.7% (roughly) livingresiding in low- and middle-income countries and growing(LMICs) where prevalence is rising at an alarming pace, particularly in Asia and Southeast Asia (1, 3) . The. In Bangladesh, prevalence of hypertension among Bangladeshi people over the age of adults aged ≥35 years has risennearly doubled, increasing from 25.7% to 48%% in recent decades (45) . Furthermore, hypertension in women is increasing day by day and it is higher than in men in LMIC including South Asia . Gender differences are striking: women, especially those ≥35 years, show a prevalence of 45% compared to 34% in men, representing a marked rise since 2011 (5-76-12) .Females aged 35 and older have a higher prevalence of hypertension (45%) than males (34%), a significant increase from the previous

Formatted: Font: 18 pt

report in 2011(8, 9) . Several studies conducted in Bangladesh have reported that the percentage of females with hypertension is higher than that of males(10, 11). Another study, based on a nationwide survey in Bangladesh of adults aged≥18 years, estimated that the proportion of women being hypertensive (28.9%) is higher than men (23.5%). Several nationwide studies confirm that women are disproportionately affected with one reporting 28.9% of women hypertensive versus 23.5% of men (13). Chronic hypertension in women carries serious implications including higher risks of maternal and neonatal complications (14). Importantly, multiple studies highlight that married women face even higher hypertension rates than unmarried or never-married women, reflecting the interplay of reproductive demands, economic responsibilities, psychosocial stressors and healthcare disparities (1215-18). Chronic hypertension among women is responsible for increasing the risk of different life-threatening maternal and newborn outcomes.

Machine learning (ML), a key component of artificial intelligence (AI) has emerged as a transformative tool in healthcare. Unlike traditional regression models, which rely on predefined assumptions of linearity and limited variable interactions, ML methods can process high-dimensional data, capture nonlinear relationships and rank the relative importance of predictors. These advantages have allowed ML to consistently outperform conventional approaches in disease prediction, particularly when datasets are complex or involve interrelated risk factors. At the same time, ML faces limitations: many models require large sample sizes for stability are sensitive to data imbalance and if not properly explained, may be viewed as “black boxes.” Addressing interpretability and ensuring fairness remain essential for public health adoption (1319-23). Furthermore, multiple studies have indicated that hypertension rates are elevated among married women when compared to unmarried, never married women or men.

In cardiovascular research, ML has been applied successfully to echocardiogram analysis and risk prediction for acute decompensated heart failure with models such as K-Nearest Neighbor (KNN), Support Vector Machines (SVM) and ensemble methods achieving strong predictive accuracy (14-1724, 25).

Machine learning (ML) has revolutionized healthcare by enhancing disease prediction and diagnosis through advanced pattern detection in large datasets. Applications in cardiovascular research, such as echocardiogram analysis and risk prediction for acute decompensated heart failure (ADHF), highlight its effectiveness, with models like K-Nearest Neighbor achieving high precision and recall. ML also supports personalized medicine by ranking key health parameters and leveraging deep learning (DL) for diagnostics, including COVID-19 imaging . For hypertension specifically, ML has demonstrated clear advantages. Tree-based algorithms like random forest (RF) and extreme gradient boosting (XGBoost) have outperformed regression-based methods with XGBoost achieving AUROC values ranging from 0.766 to 1.00 across datasets including 0.894 in semi-laboratory settings when ranking predictors such as systolic blood pressure, waist circumference and albumin levels (18, 1926-33). However, hypertension remains a significant public health issue, with many patients undiagnosed or inadequately treated despite established management guidelines. Barriers such as low adherence, treatment inertia, and system-level challenges exacerbate the issue of poor blood pressure control. Machine learning (ML), a key component of artificial intelligence (AI), offers transformative strategies to address these gaps by enabling predictive analytics and personalized interventions. Recent studies demonstrate ML's ability to optimize treatment plans, improve patient monitoring, and identify nuanced risk factors. Machine learning models like Support Vector Machines (SVM) and XGBoost consistently achieve high performance metrics, including AUROC scores between 0.766 and 1.00, for hypertension

prediction. Recursive feature elimination techniques further refine these models by ranking critical variables, enabling more targeted approaches. Additionally, innovative applications, such as integrating wearable sensors and smartphone-enabled monitoring, allow for continuous blood pressure tracking and enhance real-world feasibility . Recursive feature elimination (RFE) further enhances these models by systematically refining the set of predictors. Comparative studies also confirm that ML surpasses Cox and logistic regression in larger and more complex datasets (20-2534, 35) . Moreover, machine learning has demonstrated significant potential in predicting hypertension and identifying key risk factors with high precision. Tree-based ML models, including extreme gradient boosting (XGBoost) and random forest, have consistently outperformed traditional logistic regression in predictive accuracy. Notably, XGBoost has achieved an area under the ROC curve (AUC) of 0.894 in semi-laboratory analyses, emphasizing its ability to rank critical factors such as systolic blood pressure, waist circumference, and albumin levels . Hybrid approaches, such as combining RFE with XGBoost, have achieved superior accuracy while models applied to electronic health records capture dynamic features often missed by traditional statistics (26, 2736-39). Additionally, machine learning models have been utilized in resource-limited settings to predict hypertension risk using easily accessible factors, enabling early intervention prioritization..

The utility of ML extends beyond hypertension. In oncology, artificial neural networks and Bayesian networks stratify patients into risk categories while in obesity research, classifiers such as SVMs and quadratic discriminant analysis outperform logistic regression by detecting nonlinear behavioral patterns (2840, 41). One study compared ML algorithms to traditional models like Cox regression, in predicting hypertension incidence in moderate datasets but excels with larger, more complex datasets(29).

Machine learning (ML) has proven its effectiveness across diverse health research areas, offering significant improvements over traditional statistical approaches in predicting and classifying health outcomes, in cancer research, ML methods such as Artificial Neural Networks and Bayesian Networks have effectively classified patients into risk groups, improving disease understanding. Similarly, in obesity prediction, classifiers like SVMs and quadratic discriminant analysis identified patterns in dietary and physical activity behaviors, providing better accuracy than logistic regression (30, 31). ML also excelled in nutritional epidemiology, with k-nearest neighbors and random forests offering superior classification of cardiometabolic health risk compared to linear regression (32). In smaller datasets, ensemble models outperformed traditional approaches, such as in predicting abnormal angiograms in South African studies(33). ML has also shown its utility in imputing missing data for breast cancer recurrence prediction and improving outcomes in cerebral ischemia prediction in aneurysmal subarachnoid hemorrhage cases (34, 35). In hypertension, ML models using electronic health records have captured dynamic features, outperforming traditional methods (36). For coronary heart disease (CHD) survival prediction, ML techniques like Support Vector Machines (SVM) demonstrated high accuracy(37). Furthermore, Neural networks like MLP and RBF outperformed other classification techniques in predicting essential hypertension, handling complex data more effectively than traditional methods(38). Extreme gradient boosting and recursive feature elimination, outperformed traditional Cox regression and the recalibrated Framingham Risk Score in predicting adverse outcomes in young hypertensive patients, achieving a C statistic of 0.757(39). a hybrid model combining recursive feature elimination and classification algorithms, particularly XGBoost, achieved high prediction accuracy for hypertension outcomes, surpassing traditional methods(40). Moreover, XGBoost and

~~SVM in identifying hypertension risk using a range of clinical and behavioral data, with fewer features than traditional models(41).~~

~~In machine learning (ML) applications for Prognostic assessments, particularly concerning chronic conditions like hypertension and heart disease, the quality of the data utilized plays a critical role in model performance. Balanced datasets significantly enhance predictive accuracy and reliability~~ . Nutritional epidemiology studies show k-nearest neighbors and random forests classify cardiometabolic risk more effectively than linear regression (42). Ensemble models are particularly advantageous in smaller datasets, such as South African studies predicting abnormal angiograms where they outperformed traditional statistical approaches (43). ML has also been employed for imputing missing data, which improved breast cancer recurrence prediction and for enhancing cerebral ischemia outcome prediction in aneurysmal subarachnoid hemorrhage patients (44, 45). For coronary heart disease survival, SVMs achieved high accuracy (46) while neural networks such as multilayer perceptrons (MLP) and radial basis function (RBF) networks outperformed other classifiers in predicting essential hypertension, demonstrating their ability to capture complex and nonlinear relationships (47). Notably, gradient boosting methods with RFE outperformed Cox regression and recalibrated Framingham Risk Scores in predicting adverse outcomes in young hypertensive patients, achieving a C-statistic of 0.757 (48).

One persistent challenge in ML health research is data imbalance, which can reduce sensitivity and lead to misclassification of minority outcomes. Methods such as Synthetic Minority Oversampling Technique (SMOTE), random under-sampling (RUS) and cost-sensitive learning have been widely applied to mitigate this issue (36, 49-56) . For example, a cost-sensitive deep neural network improved mortality prediction in acute myocardial infarction patients with hypertension by 2.58% AUC compared to ensemble models (57). SVM models with SMOTE

increased accuracy from 91% to 98% (58) while a RUS-applied random forest improved stroke risk prediction among hypertensive adults, yielding AUC 0.624 and sensitivity 63.9% (59).

Interpretability is equally critical. SHAP (Shapley Additive Explanations) has emerged as a powerful, model-agnostic framework that provides consistent and transparent feature attribution (60-65). Unlike LASSO or ANOVA, SHAP captures nonlinearities and interaction effects, offering granular, instance-level explanations. Visualization tools enhance communication with clinicians, and SHAP's flexibility in handling missing and imbalanced data makes it highly suitable for real-world datasets (42-4462, 65-69). The integration of resampling methods not only boosts classification ability but also fosters better generalization of models in clinical settings (45, 46). As study highlights the detrimental impact of data imbalance on predictive performance using cervical cancer datasets exhibit skewed distributions that lead to misclassification of minority classes (47). Similarly, Similarly, A cost-sensitive deep neural network that effectively addresses the skewed class distribution in mortality risk assessment for acute myocardial infarction patients with hypertension, it outperformed traditional ML and ensemble models with a 2.58% AUC improvement (48). Moreover, leveraging balanced datasets enables more accurate identification of risk factors, ultimately supporting healthcare professionals in making informed decisions, in Parkinson's disease datasets with skewed distributions, these techniques have improved accuracy to 98.2% with Support Vector Machine (SVM) models and achieved high specificity (99%) with K-Nearest Neighbors (KNN) (49, 50).The effective integration of the Synthetic Minority Over-sampling Technique (SMOTE) to balance class distributions in datasets, thereby improving the predictive capabilities of the machine learning model such as, Hybrid Prediction Model (HPM), random forest for heart failure, diabetes and hypertension (43, 44, 51-53). Similarly, Employing the Synthetic Minority Oversampling Technique (SMOTE) in a Support Vector Machine model

significantly boosts accuracy from 91% to 98%, underscoring the effectiveness of balancing techniques in hypertension classification (54). Moreover, A study reveals that implementing random under sampling (RUS) enhances the predictive capabilities of random forest models in stroke risk assessment among hypertensive adults. The RUS applied RF model, incorporating laboratory variables, achieved superior performance compared to null models and other data-balancing techniques, with an AUC of 0.624 and sensitivity of 63.9% (55). Additionally, SHAP (SHapley Additive exPlanations) offers several advantages over conventional statistical methods by providing model-agnostic interpretability, allowing it to explain both simple and complex machine learning models (56, 57). Unlike traditional statistical approaches that rely on linear models, SHAP captures non-linear patterns and complex feature interactions, ensuring granular, instance-level insights beyond global coefficients(56, 58, 59) . It provides consistent and accurate feature attribution through Shapley values, addressing limitations like interaction effects and heterogeneity overlooked by methods such as LASSO or ANOVA (58-60). SHAP's visual tools (e.g. force and summary plots) further enhance interpretability for domain experts (58, 61) while its flexibility with missing and imbalanced data makes it suitable for real-world applications (62, 63). Additionally, SHAP’s ability to reveal hidden features, support multi-omics data analysis, and facilitate human-machine collaboration enhances both model performance and personalized decision-making in fields like healthcare (58, 61, 64, 65).

Machine learning (ML) has emerged as a transformative tool in hypertension research, enabling the identification of predictive factors and the analysis of high-dimensional, complex datasets. Comparative studies leveraging nationally representative datasets from South Asia, including Bangladesh, Nepal, and India, have demonstrated the superior performance of advanced ML algorithms such as gradient boosting (GB), random forest (RF), and support vector machine

~~(SVM) over traditional statistical methods, including logistic regression. For instance, the integration of SVM recursive feature elimination (SVMRFE) with GB achieved an area under the curve (AUC) of 0.669 and a recall of 97.92% for hypertension prediction, effectively prioritizing risk factors such as age, BMI, and diabetes mellitus. Despite these methodological advancements, a critical knowledge gap persists regarding the application of predictive models to specific subpopulations, particularly married women in Bangladesh. Prior investigations have predominantly focused on generalized adult cohorts, overlooking the distinct health and socio-cultural determinants relevant to married women, including reproductive health complexities, economic responsibilities, and disparities in healthcare accessibility. Advanced feature selection methods such as LASSO and Boruta algorithms, coupled with data balancing techniques like the Synthetic Minority Oversampling Technique (SMOTE), have been shown to enhance model calibration, improve sensitivity, and refine the ranking of feature importance. However, a lack of robust subgroup validation and generalizability remains a significant limitation in the existing body of research (66-71). Therefore, the objective of this study is to develop a machine learning model for identifying the predictive factors of hypertension among married women in Bangladesh. To achieve this, we explored optimal algorithms in combination with class-balancing techniques, utilizing data from the Bangladesh Demographic and Health Survey (BDHS). Finally, we ranked and identified both the positive and negative predictive factors associated with hypertension among this population.~~

. Moreover, its support for multi-omics integration and human–machine collaboration further enhances its utility in personalized healthcare.

Despite these advances, significant gaps remain. Most hypertension-focused ML studies in South Asia including Bangladesh have concentrated on general adult populations with little attention to

married women, who face distinct risks shaped by reproductive roles, domestic workloads and socio-economic constraints. Subgroup-specific validation remains limited, and few studies explicitly integrate cultural and gender-related determinants into predictive models. Although advanced methods such as SMOTE enhance calibration and sensitivity (70-76), they have rarely been applied to this subgroup in Bangladesh. Furthermore, BDHS-2022 was released after our analysis, that is why we trained the model on BDHS-2017-18 and pre-specified external validation on BDHS-2022. This approach ensures robust assessment of generalizability without altering the model development process based on a single additional dataset. Against this backdrop, the present study aims to develop ML models to identify predictive factors of hypertension among married women in Bangladesh. To our knowledge, this is the first study in Bangladesh to apply an extensive set of algorithms combined with class-balancing techniques to this population, contributing both methodological innovation and population-specific insights. Therefore, the objective of this study is to develop and validate interpretable machine-learning models for predicting hypertension among married women in Bangladesh, integrating sociodemographic, behavioral and biometric factors (such as BMI and diabetes status) to identify the most influential predictors and provide evidence to guide targeted screening and public health interventions in low-resource settings

Methodology

Data sources

We have used Bangladesh Demographic and Health Survey (BDHS) 2017-18 data in this study, which is the nationally representative survey. The National Institute of Population Research and Training, Medical Education and Family Welfare Division, and Ministry of Health and Family Welfare jointly conducted the survey from October 2017 to March 2018.

Sampling method and sample size /study population and survey design

The Bangladesh Bureau of Statistics of the 2011 Population and Housing Census of the People's Republic of Bangladesh provided a complete list of enumeration areas (EAs) covering the whole residing population in Bangladesh, which was used in a survey to determine the sampling frame for the 2017-18 BDHS. The survey employed a two-stage stratified cluster sampling as a sampling method where, in the 1st stage of sampling, 675 enumeration areas (EAs) were chosen, whereas 250 EAs were from urban areas and 425 from rural areas, with a probability proportional to the EA scale, and then a systematic sample of 30 households per EA was chosen in the 2nd stage of sampling to provide statistically accurate estimates of key demographic and health variables for the nation as a whole, rural and urban areas separately, and each of the eight divisions. Finally, after selecting 20,250 residential households, approximately 20,100 ever-married women aged 15–49 were expected to complete the interviews (27). At last, 19,457 households were successfully interviewed, and 5,138 women underwent blood pressure and blood glucose measurements. From the 4,546 married women, 4,253 women were considered for final analysis after the termination of pregnant women and deleting missing value or missing information (Fig 1).

Formatted: Font: Times New Roman, 12 pt

In this analytic sample of 4,253 married women, the prevalence of hypertension was about 23.1% (n ≈ 982) ensuring an adequate number of positive events for training supervised ML models. This sample size provides sufficient power for developing and evaluating predictive algorithms in imbalanced health data contexts, consistent with prior methodological recommendations (77-79).

Fig.1: Data selection flow chart

Dependent feature

Hypertension was considered if the participant’s systolic blood pressure was≥140 mmHg or the diastolic blood pressure was≥ 90 mmHg or if the person had been taking prescribed medicine to lower blood pressure(~~72~~80).

Field Code Changed

Independent feature

The independent variables in the study are considered from the previous related literature(~~73-75~~81-83). In this study, we considered administrative division of Bangladesh (Barisal, Chittagong, Dhaka, Khulna, Mymensingh, Rajshahi, Rangpur ,Sylhet) type of place of residence (urban, rural) respondents highest educational level (no education, primary, secondary and higher)husband/partner educational level (no education, primary, secondary and higher), unmet need for contraception (unmet need for spacing, unmet need for limiting, using for spacing, using for limiting, no unmet need, infecund, menopausal) religion (Islam, Hinduism, Buddhism, Christianity) sex of household head (male, female) wealth index combined (poorest, poorer, middle, richer, richest), current use by method type (no method, folkloric method, traditional method, modern method) currently amenorrhoeic (yes, no) currently abstaining (yes, no) currently residing with husband/partner (living with her, staying elsewhere) household members (< 4 persons and ≥4 persons) respondent's occupation (working, not working) number of living children (no living children, one, two and more than two children) husband/partner's occupation (working, not working) respondent's current age (less than 35 years, 35 to 40 years and above 40 years old) husband/partner's age (less than 35 years, 35 to 40 years and above 40 years old) total children ever born (no children ever born, one, one to three and above three children born) births in last five years (no birth, one and above one) daughters who have died (no died, at least one died) sons

who have died (no died, at least one died) age difference between husband/partner and wife (less than ten year, ten and above).

Derived variables

Diabetes Status: Fasting plasma glucose (FPG) was considered to calculate diabetes. The HemoCue Glucose 201 DM system with plasma conversion was used to test a drop of capillary blood obtained from consenting eligible respondents from the middle or ring finger. The system automatically converted the fasting whole blood glucose measurements taken in the survey to FPG equivalent values~~(76, 77).~~(84, 85). To classify diabetes World Health Organization (WHO) criteria were used~~(78).~~(86). Diabetes was considered if the FPG level was greater than or equal to 7 mmol/l or self-reported diabetes medication use.

Body ~~mass index~~**Mass Index (BMI**~~) is classified into four categories~~**):** Calculated as weight in kilograms divided by height in meters squared (kg/m²). Categories were defined according to WHO cut-offs: underweight ~~(BMI<~~(<18.5), normal (~~BMI>=~~18.5~~ & BMI<=~~ –24.9), overweight (~~BMI >24.9 & BMI <=~~25.0–29.9) and obese ~~(BMI >29.9)(79)~~(≥30) (87).

Feature selection

All sociodemographic, behavioral, biometric and anthropometric variables with theoretical or empirical relevance to hypertension were extracted from the BDHS 2017–18 dataset. No additional feature engineering or automated feature selection algorithms (e.g., Boruta, LASSO, or recursive feature elimination) were applied. Instead, variable inclusion was guided by existing epidemiological literature and prior BDHS-based hypertension studies.

Formatted: Strong

Formatted: Normal (Web)

Formatted: Strong

Data preparation

Missing data

Cases with missing values were excluded. No imputation was performed to avoid introducing artificial variability.

Survey weights

The BDHS 2017–18 employs a complex survey design with stratification, clustering and sampling weights to ensure national representativeness. In the present study, we did not apply survey/sample weights because the primary aim was methodological focused on evaluating and comparing the predictive performance of class balancing approaches integrate with machine learning algorithms and ranked risk and protected features rather than estimating population-level prevalence or nationally representative parameters. This approach is consistent with prior ML studies using DHS data in similar contexts (73, 88).

Feature scaling

Data normalization; which is a process of re-scaling the feature value, is very important because most of the machine learning algorithms use Euclidean distance between two data points as a distance metrics, so without Feature scaling, the machine learning algorithms may not execute properly~~(80).~~(89). For rescaling, standardization technique has been applied which the mean is zero and the standard deviation is one.

~~One-hot encoding for categorical features~~

~~We used~~Encoding

Categorical variables were transformed using one-hot encoding ~~technique~~ to ~~split categorical variables into multiple binary variables with a view to utilizing data as an input of machine learning~~allow use in ML algorithms.

Formatted: Space Before: Auto, After: Auto, Line spacing: single

Formatted: Font: 12 pt

Imbalanced data problem

As imbalanced data lead to the majority class dominates minority class that's why the, it impacts the reliability of determinations from the dataset, algorithm biased towards majority class and may provide more inaccurate result(~~81-83~~90-92). It is found that for balanced data (where classes proportion are equal) may lead extract best result for identifying the factors. To convey the issue of imbalanced data, Synthetic Minority Oversampling Techniques (SMOTE), Adaptive Synthetic Sampling (ADASYN), Tomek Links (TLs), Edited Nearest Neighbor (ENN), SMOTE-TomekLinks, SMOTE-ENN techniques are applied to resolve the issue (see supplementary File S1 Appendix).

Machine learning algorithms

We evaluated ~~several machine learning~~12 algorithms ~~incorporating with different class-balancing techniques to identify the best-performing model for predicting hypertension outcomes among married women in Bangladesh. We utilized~~: Logistic Regression, Decision ~~Trees, and~~Tree, K-Nearest Neighbors (KNN), Random Forest, Extra Trees, AdaBoost, ~~XGBoost, CatBoost,~~ Gradient Boosting Machine (GBM), ~~and~~XGBoost, LightGBM~~. Additionally, we explored Neural Networks using a~~ , CatBoost, Support Vector Machine (SVM) and Multilayer Perceptron (MLP~~) architecture~~).

We included Extra Trees as it is computationally efficient, less prone to overfitting in small subgroups and yields stable feature importance rankings, complementing RF and ~~Support Vector Machines (SVM). A detailed description of each~~XGBoost (93-95). Detailed algorithm ~~can be found~~descriptions are in ~~supplementary file~~Supplementary File S2~~ Appendix .~~.

Formatted: Space Before: Auto, After: Auto

Formatted: Font: 12 pt, Font color: Auto

Model training with parameter optimization

Hold-out cross validation

Original dataset is divided into training and testing subsets where 80% data belongs to training and the rest 20% belongs to testing subset.

Repeated stratified k fold cross validation

The dataset is divided into k-folds, where one of the k-folds is selected as a validation set and the remaining sets comprise the training set. Until each one of them forms validation sets, the operation is repeated for each fold, which means for the n number of repetitions the process will be repeated k×n times.

We avoided overfitting and underfitting problems by employing hold-out cross-validation to split training and testing set and to reduce the sampling error, repeated stratified k-fold cross-validation as a validation method was applied. Additionally, we utilized random search to select model parameters using hyper-parameters as there is more chance to select the best parameter (~~84~~96).

Field Code Changed

We note that external validation was not possible as BDHS 2022 data was not fully accessible at the time of analysis. This remains a priority for future work to assess model generalizability.

Evaluation methods

To assess the performance of machine learning methods, we employed confusion matrix, Matthews correlation coefficient, Cohens-kappa, F1-score, G-mean, recall/ sensitivity, specificity, accuracy, precision, AUC-ROC, AUC-PR. For the evaluation of the matric score we used the Anderson-Darling test to check data normality, One-way repeated measure ANOVA was utilized to determine the overall difference among class-balancing techniques, Tukey's HSD test was used to classify the significance difference among specific group of class ~~balanced~~balancing techniques, Friedman test was used to find the difference among group instead of one-way ANOVA where normality assumptions are violated (supplementary File S3 Appendix~~-~~).

External validation plan

At the time of model development, BDHS-2022 data was not publicly released; consequently, model training and internal validation used BDHS-2017/18. BDHS-2022 is now available and differs modestly in feature scope; to avoid design leakage, we will treat BDHS-2022 strictly as an external test set. Given the scarcity of comparable datasets of the same type, we also reserve BDHS-2022 for validating the deployed system. No retraining or feature re-specification will be performed for the external test; instead, we will apply identical preprocessing and the pre-specified decision threshold. This analysis will provide an out-of-sample assessment of generalizability across a later, post-COVID cohort and any instrument changes.

Software and hardware

~~Variable management and categorization were~~ Data preprocessing was performed ~~using~~in STATA 15, ~~with machine learning tasks implemented~~ML models in Python (scikit-learn, XGBoost,

Formatted: Space Before: Auto, After: Auto

CatBoost, LightGBM, TensorFlow etc.) and statistical tests ~~conducted~~ in R. ~~Model training~~Training was carried out on a workstation ~~equipped~~ with ~~a 1.60 GHz~~ Intel Core i5-8365U CPU (1.60 GHz) and 8 GB ~~of~~ RAM. While resource limitations constrained deeper architectures and exhaustive grid searches, the chosen algorithms were successfully optimized within these constraints.

Multicollinearity

To assess multicollinearity, we examined variance inflation factors (VIF) and pairwise correlations among predictors. As expected with one-hot encoded categorical variables, VIF values were inflated to infinity due to perfect linear dependence between dummy categories. Because our primary models were gradient-boosted decision trees, which are robust to correlated features, this did not compromise predictive validity. For clarity, we also inspected pairwise Spearman correlations among key predictors, focusing on socio-demographic and fertility-related variables, which often show natural dependencies.

Ethical ~~Considerations~~considerations

Formatted: Left

This study utilized publicly available secondary data from the Bangladesh Demographic and Health Survey (BDHS), which is conducted by the ICF and the Bangladesh Medical Research Council (BMRC). Prior to the data collection, ethical approval was obtained from the Institutional Review Board (IRB) of ICF, USA, and the National Research Ethics Committee of the BMRC. Informed written consent was obtained from all participants involved in the original survey.

As this study involves secondary data analysis, we obtained permission to access the de-identified data from the DHS Program. Since the data was de-identified and publicly available, no additional

ethical approval was required for this analysis. The study adhered to the relevant guidelines and regulations for secondary data use.

We additionally highlight that fairness and bias are critical in ML health research. Sensitive variables were treated with caution and SHAP interpretability was used to ensure transparency in feature attribution. These safeguards support responsible use of advanced ML in public health policy.

Study workflow

An overview of the analytical workflow from data extraction and preprocessing through class balancing, model training, hyperparameter tuning, validation and evaluation, followed by statistical analysis to compare class-balancing techniques and model performance and SHAP-based interpretation is presented in Fig 2. This schematic provides a concise visual summary of the methodological pipeline described in the preceding subsections.

Fig 2. Workflow of machine learning pipeline for hypertension prediction and risk factor ranking of married women in Bangladesh.

Result

Outcome characteristics

Among 4,253 participants, 23.1% of married women in Bangladesh were found to have , 23.1% had hypertension, while and 76.9% had normal blood pressure.were normotensive.

Socio-demographic and clinical characteristics of the study participants

The demographic and clinical characteristics of hypertension among married women in Bangladesh are illustrated in Table 1. The majority, 64.7% of participants reside inParticipants were predominantly rural areas, and 13.8% were living in the Chittagong division. Among the 4,253 participants, 19.5% were classified as (64.7%). Wealth distribution was approximately even across quintiles (poorest, while 21.9% were categorized as 19.5% to richest. Additionally, 37.7% of participants had completed 21.9%). Educational attainment was most commonly secondary education, whereas the highest education level for most of for women (37.7%) and primary for husbands/partners was primary (32.7%. Contraception use was reported by 12.7% of participants for reasons of infecundity or menopause, and 49.9% for limiting.

The respondents' ages ranged from%). Median respondent age was 31 years (IQR 25 to –39 years old, with a); median age of 31 years and Husband/partner's ages ranged from 32 to 48 years old, with a medianhusband/partner age ofwas 40 years.

(IQR 32–48). Most respondents about 61.1% were under <35 years of age, while the majority approximately (61.1%) whereas 41.9% of husbands/partners were over >40 years old. Among married women, 38.7% had more than. Fertility profiles showed 51.1% with two children, 51.1% had one to three ever-born children, and; 59.4% had no births in the past five years.

In terms of nutritional Nutritional status, 11.7% of individuals were was: underweight, 53.9% had a 11.7%, normal weight, 27.4% were53.9%, overweight, 27.4%, and 7.0% were obese. Regarding diabetes status, 1.3% of the population had hypoglycemia, 7.0. Glycemic categories were: normoglycemia 77.8% were normoglycemic, 13.1% had%, intermediate hyperglycemia, and 7.8% were hyperglycemic. 13.1%, hyperglycemia 7.8% and hypoglycemia 1.3% (Table 1).

Formatted: Font: Times New Roman, 12 pt

Stratification of hypertension among married women in Bangladesh

Table 1 demonstrates ~~demographic and clinical factors stratified~~that Prevalence varied by ~~hypertension status. Hypertension prevalence among married women in Bangladesh was~~division, highest in Rangpur (27.8%) and lowest in Mymensingh (18.8%). ~~-~~Urban ~~residents exhibit a higher proportion (24.1%) than rural counterparts (~~residence was associated~~22.5%).31.3% of Individuals were without education whereas 18.9%~~ with higher prevalence than rural (24.1% vs. 22.5%). Prevalence decreased with higher female education (no education~~. Similarly, households where the husband has no formal~~ 31.3%, higher education ~~experience~~18.9%) and was elevated ~~hypertension~~when husbands had no education (26.6~~%) compared to those with higher-educated partners (22.2%). Economic status also influences hypertension, with the wealthiest quintile exhibiting the highest proportion (27.6%) and the ~~%). Hypertension rose across wealth quintiles (poorest ~~showing the lowest (~~18.7% to richest 27.6%). Reproductive ~~factors such as infecundity or menopause elevated hypertension levels (~~status mattered: women who were infecund/menopausal had 37.1~~%). Participants ~~% prevalence; those using contraception ~~for limiting~~to limit births ~~have notably the highest hypertension (~~had 27.1~~%),~~% whereas ~~the lowest is observed among those with ~~unmet ~~needs~~need for spacing ~~births~~had the lowest (8.7%).

~~Hypertension increases with age; respondents above 40 years (41.7%) exhibit the highest levels, while those under 35 years' experience the lowest (13.5%). This trend extends to partners, with individuals whose husbands or partners are above 40 years old showing a hypertension level of 35.1%. ~~Age gradients were pronounced: <35 years 13.5%, 35–40 years 33.9% and >40 years 41.7%. Husband/partner age >40 years was also associated with higher prevalence (35.1%). BMI showed a graded pattern: underweight 11.1%, normal 17.7%, overweight 34.0%, obese 41.9%. By

Formatted: Normal (Web)

religion, the highest stratum was among Christians (36.4%) and the lowest among Muslims (22.4%), acknowledging small denominators in minority groups. Households with >2 living children had higher prevalence (31.1%). Participants whose husbands/partners were not working had higher prevalence (40.0%) versus those with employed partners (22.7%), noting the small size of the unemployed group (n=80). Smaller households (<4 persons) showed higher prevalence (26.1%) than larger households (22.3%).

Obese participants exhibited the highest hypertension proportion (41.9%) compared to underweight individuals (11.1%). In addition, participants with hypoglycemia (40.1%) demonstrate higher hypertension levels than those with normoglycemia (21.3%).

Christian participants (36.4%) show the highest levels among religious groups, while Muslims (22.4%) exhibit the lowest. Households with more than two children (31.1%) and those whose sons have died (28.6%) also experience elevated hypertension. Partners' occupations are notable; participants whose partners are unemployed show hypertension levels of 40.0%, compared to 22.7% for those with working partners.

Table 1: Demographic and clinical characteristics of study participants.

**Characteristics**	**Total**	**Hypertension**	**95% CI**
**Administrative division of Bangladesh**			
Barisal	453 (10.7)	115 (25.4)	21.6–29.6
Chittagong	588 (13.8)	160 (27.2)	23.8–30.9
Dhaka	579 (13.6)	114 (19.7)	16.7–23.1
Khulna	588 (13.8)	136 (23.1)	19.9–26.7
Mymensingh	467 (10.9)	88 (18.8)	15.6–22.6
Rajshahi	580 (13.6)	125 (21.6)	18.5–25.0

Rangpur	551 (12.9)	153 (27.8)	24.2–31.7
Sylhet	447 (10.5)	90 (20.1)	16.6–24.1
**Type of place of residence**			
~~urban~~Urban	1,503 (35.3)	362 (24.1)	21.9–26.5
~~rural~~Rural	2,750 (64.7)	619 (22.5)	20.8–24.3
**~~Respondents~~Respondent's highest educational level**			
~~no~~No education	689 (16.2)	216 (31.3)	27.9–34.9
~~primary~~Primary	1,372 (32.3)	332 (24.2)	21.9–26.7
~~secondary~~ Secondary	1,605 (37.7)	322 (20.1)	18.2–22.2
~~higher~~Higher	587 (13.8)	111 (18.9)	15.9–22.3
**Husband/partner educational level**			
~~no~~No education	943 (22.2)	251 (26.6)	23.8–29.5
~~primary~~Primary	1,391 (32.7)	288 (20.7)	18.6–22.9
~~secondary~~ Secondary	1,240 (29.2)	291 (23.5)	21.2–26.0
~~higher~~Higher	679 (15.9)	151 (22.2)	19.2–25.5
**Unmet need for contraception**			
~~unmet~~Unmet need for spacing	172 (4.0)	15 (8.7)	5.3–14.0
~~unmet~~Unmet need for limiting	257 (6.0)	59 (23.0)	18.1–28.8
~~using~~Using for spacing	723 (17.0)	69 (9.9)	7.9–12.4
~~using~~Using for limiting	2,124 (49.9)	574 (27.1)	25.2–29.1
~~no~~No unmet need	435 (10.2)	63 (14.5)	11.5–18.0
Infecund ~~or~~ /menopausal	542 (12.7)	201 (37.1)	33.2–41.2
**Religion**			
Islam	3,813 (89.7)	855 (22.4)	21.1–23.7
Hinduism	403 (9.5)	114 (28.3)	24.2–32.8
Buddhism	26 (0.6)	8 (30.8)	16.2–51.6
Christianity	11 (0.3)	4 (36.4)	14.5–66.6
**Sex of household head**			

~~male~~Male	3,724 (87.6)	859 (23.1)	21.8–24.4
~~female~~Female	529 (12.4)	122 (23.1)	19.8–26.8
**Wealth index combined**			
~~poorest~~Poorest	827 (19.5)	154 (18.7)	16.2–21.6
~~poorer~~Poorer	833 (19.6)	176 (21.1)	18.4–24.0
~~middle~~Middle	837 (19.7)	194 (23.2)	20.5–26.1
~~richer~~Richer	821 (19.3)	199 (24.2)	21.4–27.3
~~richest~~Richest	935 (21.9)	258 (27.6)	24.7–30.7
**Current use of contraceptive methods ~~among married women~~**			
~~no~~No method	1,406 (33.1)	338 (24.1)	21.9–26.4
~~folkloric~~Folkloric method	14 (0.3)	4 (28.6)	11.7–55.3
~~traditional~~Traditional method	455 (10.7)	126 (27.7)	23.7–32.1
~~modern~~Modern method	2,378 (55.9)	513 (21.6)	20.0–23.4
**Currently amenorrhoeic**			
~~yes~~Yes	4,058 (95.4)	948 (23.4)	22.1–24.8
~~no~~No	195 (4.6)	33 (18.5)	13.5–24.8
**Currently abstaining**			
~~yes~~Yes	4,112 (96.7)	955 (22.2)	20.9–23.6
~~no~~No	141 (3.3)	26 (18.5)	12.9–26.0
**Currently residing with husband/partner**			
~~living~~Living with her	3,564 (83.8)	848 (23.8)	22.4–25.2
~~staying~~Staying elsewhere	689 (16.2)	133 (19.3)	16.5–22.5
**Household members**			
<~~-~~4 persons	849 (19.9)	221 (26.1)	23.1–29.4
≥4 persons	3,404 (80.1)	760 (22.3)	21.0–23.8
**Employment status of respondent**			
~~working~~Working	2,231 (52.5)	528 (23.7)	21.9–25.6

~~not~~Not working	2,022 (47.5)	453 (22.4)	20.6–24.3
**Number of living children**			
~~no~~No living children	275 (6.5)	35 (12.7)	9.3–17.1
~~one~~One	926 (21.8)	118 (12.7)	10.7–15.1
~~two~~ Two	1,405 (33.0)	316 (22.5)	20.3–24.9
~~more~~More than two ~~children~~	1,647 (38.7)	512 (31.1)	28.8–33.6
**Employment status of husband/partner**			
~~working~~Working	4,173 (98.1)	949 (22.7)	21.4–24.0
~~not~~Not working	80 (1.9)	32 (40.0)	29.9–51.2
**~~Respondent's~~Respondent's current age**			
~~Age (Median, IQR)~~	~~31 (25,39)~~	~~38 (32,44)~~	
~~less than~~ <35 years	2,598 (61.1)	352 (13.5)	12.2–15.0
35 ~~to~~ –40 years	783 (18.4)	265 (33.9)	30.6–37.3
~~above~~ >40 years ~~old~~	872 (20.5)	364 (41.7)	38.4–45.1
**Husband/~~partner's~~partner's age**			
~~Age (Median, IQR)~~	~~40 (32,48)~~	~~45 (39,54)~~	
~~less than~~ <35 years	1,229 (28.9)	113 (9.2)	7.7–11.0
35 ~~to~~ –40 years	1,240 (29.2)	243 (19.6)	17.4–22.0
~~above~~ >40 years ~~old~~	1,784 (41.9)	625 (35.1)	32.9–37.5
**Total children ever born**			
~~no~~No children ever born	268 (6.3)	31 (11.6)	8.3–16.0
~~one~~One	844 (19.8)	100 (11.8)	9.7–14.0
~~two~~Two to three	2,174 (51.1)	548 (25.2)	23.5–27.1
~~and above~~Above three	967 (22.7)	302 (31.2)	28.3–34.3
**Births in last five years**			
~~no~~No birth	2,526 (59.4)	755 (29.9)	27.9–32.0
~~one~~ One	1,456 (34.2)	193 (13.3)	11.6–15.3
~~above~~Above one	271 (6.4)	33 (12.2)	8.8–16.7
**Daughters who have died**			
~~no died~~None	3,918 (92.1)	887 (22.6)	21.3–23.9

Formatted
Formatted
Inserted Cells
Formatted
Formatted
Formatted
Inserted Cells
Formatted
Formatted
Formatted
Formatted
Formatted
Formatted
Formatted
Formatted
Formatted
Formatted
Formatted
Formatted
Inserted Cells
Inserted Cells
Formatted
Formatted
Formatted
Formatted
Formatted
Formatted
Formatted
Inserted Cells
Inserted Cells
Formatted Table
Formatted
Inserted Cells
Formatted
Formatted
Formatted
Formatted
Formatted
Formatted
Formatted
Formatted
Formatted
Inserted Cells
Inserted Cells
Formatted
Formatted Table
Formatted
Inserted Cells
Formatted
Formatted
Formatted
Formatted
Formatted

~~at least one died~~≥1 daughter	335 (7.8)	94 (28.1)	23.6–33.2
**Sons who have died**			
~~no died~~None	3,827 (89.9)	859 (22.4)	21.1–23.7
~~at least one died~~≥1 son	426 (10.1)	122 (28.6)	24.5–33.2
**Age difference between husband/partner and wife**			
~~less than ten year~~<10 years	3,001 (70.6)	667 (22.2)	20.8–23.6
~~ten and above~~≥10 years	1,252 (29.4)	314 (25.1)	22.7–27.7
**Body mass index (BMI)**			
Underweight~~underweight~~	497 (11.7)	55 (11.1)	8.5–14.3
Normal~~normal~~	2,295 (53.9)	406 (17.7)	16.2–19.4
Overweight~~overweight~~	1,163 (27.4)	395 (34.0)	31.3–36.9
Obese~~obese~~	298 (7.0)	125 (41.9)	36.5–47.5
**Diabetes**			
Hypoglycemia	56 (1.3)	11 (19.6)	11.5–31.7
Normoglycemia	3,307 (77.8)	706 (21.3)	19.9–22.7
Intermediate hyperglycemia~~Hyperglycemia~~	558 (13.1)	131 (23.5)	20.3–27.0
Hyperglycemia~~Hypoglycemia~~	332 (7.8)	133 (40.1)	34.9–45.6

Class distribution before and after class balancing

To ensure transparency in preprocessing, we report class distributions in the training data before and after class balancing. The original training set contained 3,581 women, with 827 (23.1%) hypertensive and 2,754 (76.9%) normotensive. Both SMOTE and ADASYN achieved perfect balance (50/50). Tomek Links modestly increased the minority proportion to 24.7% by removing borderline majority cases, while ENN shifted the minority share to 35.0%. Hybrid methods produced stronger changes: SMOTE+Tomek restored exact balance (50/50), whereas

SMOTE+ENN yielded a minority-dominant distribution (63.3% hypertensive). These comparisons highlight trade-offs between balance and retained sample size across class balancing strategies (Supplementary Table S1).

Parameter optimization

Model performance shifted ~~significantly~~ with ~~different~~ sampling ~~techniques~~technique. Logistic Regression ~~achieved optimal results using~~ performed best with L2 ~~regularization~~ (C=11.29~~) and the~~; lbfgs~~ solver)~~ on the actual data, but under SMOTE~~,~~ /ADASYN~~,~~ /TomekLinks~~, and~~ /ENN~~, it~~ required lower C~~ values, balanced~~, class ~~weights~~weighting, or ~~l1 regularization~~L1 with ~~the~~ saga ~~solver~~. Extra Trees ~~performed best with~~ favored 200 estimators, log2 ~~feature selection~~features, and entropy ~~criterion~~on actual data; SMOTE reduced trees and used Gini, while ADASYN~~ and~~ /TomekLinks retained entropy and increased trees. Decision Trees ~~favored~~preferred deeper structures~~ and~~ /smaller ~~leaf sizes~~leaves under SMOTE and SMOTE+ENN~~, with~~; ENN ~~preferring~~favored Gini. AdaBoost ~~improved with~~benefited from more estimators ~~(up to 178),~~and higher learning rates (up to 1.5), ~~and~~often with SAMME.R under ADASYN~~ and~~ /ENN. SVMs ~~initially used~~shifted between RBF ~~(C=0.8) but switched to poly~~and polynomial kernels ~~and higher C under ADASYN.~~depending on sampler. XGBoost~~ configurations adapted max_depth and split thresholds based on sampling. Neural Networks varied in activation, layers, solvers, and learning rates across samplers.~~, LightGBM~~ increased depth under SMOTE while maintaining a 0.05 learning rate.~~, KNN~~ used k=15 with Manhattan distance but adjusted neighbors and algorithms post-sampling.~~, CatBoost~~ tuned iterations and l2_leaf_reg per method.~~, Random Forest~~ and GBM models favored deeper trees, altered estimator counts, and varied max_features settings, especially under~~ , and GBM all exhibited sampler-specific hyperparameter adjustments (Table 2). Unless

otherwise noted, cross-model metrics in Table 5 are fold-averaged over stratified 5-fold CV on the training set. For the selected ExtraTrees + SMOTE+ENN and SMOTE+TomekLinks (Table 2). model, test-set uncertainty is reported via 95% bootstrap CIs (Table 6).

Formatted: Default Paragraph Font

Table 2: Optimal parameters of the machine learning algorithm.

ML model	Sampling	Parameter	ML model	Sampling	Parameter
Logistic Regression	Actual	Penalty=l2, C= 11.288378916846883, Solver=lbfgs, Class_ weight =None, Max _iter=2500	Extra Trees	Actual	n_ estimators= 200, max_ features= log2, max_ depth=None, min_ samples_ leaf=6, min_ samples_ split=76, bootstrap=True, criterion=entropy
	SMOTE	Penalty =l2, C =1.623776739188721, Solver=liblinear, Class _weight=None, Max _iter=1000		SMOTE	n_ estimators=52, max_ features=log2, max_ depth=None, min_ samples_ leaf=8, min_ samples_ split=16, bootstrap=False, criterion= gini
	ADASYN	Penalty=l2, C=0.012742749857031334, Solver =liblinear, Class _weight =balanced, Max _iter=5000		ADASYN	n_ estimators=178, max_ features=None, max_ depth=None, min_ samples_ leaf=2, min_ samples_ split=10, bootstrap=True, criterion=entropy
	TomekLinks	Penalty =l2, C =0.0018329807108324356, Solver=lbfgs, Class _weight=balanced, Max _iter=2500		TomekLinks	n_ estimators=115, max_ features=None, max_ depth=None, min_ samples_ leaf=2, min_ samples_ split=30, bootstrap=True, criterion=entropy
	ENN	Penalty =l1, C=0.23357214690901212, Solver =saga, Class _weight=None, Max _iter= 2500		ENN	n_ estimators=157, max_ features=None, max_ depth=None, min_ samples_ leaf=2, min_ samples_ split=6, bootstrap=True, criterion=entropy

	SMOTE+ TomekLinks	Penalty =l2,<br>C= 11.288378916846883,<br>Solver= liblinear,<br>Class _weight= None,<br>Max _iter =5000		SMOTE+ TomekLinks	n_ estimators=157,<br>max_ features=None,<br>max_ depth=None,<br>min_ samples_ leaf=6,<br>min_ samples_ split=18,<br>bootstrap=False,<br>criterion=entropy
	SMOTE+ ENN	Penalty =l1,<br>C= 0.23357214690901212,<br>Solver =liblinear,<br>Class _weight=None,<br>Max _iter=5000		SMOTE+ ENN	n_ estimators=94,<br>max_ features=None,<br>max_ depth=None,<br>min_ samples_ leaf=6,<br>min_ samples_ split=6,<br>bootstrap=True,<br>criterion=entropy
Decision tree	Actual	Max _depth=12,<br>Min _samples _split=44,<br>Min _samples _leaf= 44,<br>Criterion= entropy	AdaBoost	Actual	Algorithm=SAMME.R,<br>n_ estimators=73,<br>learning_ rate=0.5
	SMOTE	Max _depth=28,<br>Min _samples _split=52,<br>Min _samples _leaf= 4,<br>Criterion= entropy		SMOTE	Algorithm=SAMME,<br>n_ estimators=136,<br>learning_ rate=1.5
	ADASYN	Max _depth=16,<br>Min _samples _split=48,<br>Min _samples _leaf= 6,<br>Criterion= entropy		ADASYN	Algorithm=SAMME.R,<br>n_ estimators=178,<br>learning_ rate=1.5
	TomekLinks	Max _depth=4,<br>Min _samples _split=86,<br>Min _samples _leaf= 40,<br>Criterion= entropy		TomekLinks	Algorithm=SAMME,<br>n_ estimators=115,<br>learning_ rate=1.5
	ENN	Max _depth=8,<br>Min _samples _split=88,<br>Min _samples _leaf= 32,<br>Criterion= gini		ENN	Algorithm=SAMME.R,<br>n_ estimators=136,<br>learning_ rate=0.5
	SMOTE+ TomekLinks	Max _depth=22,<br>Min _samples _split=52,<br>Min _samples _leaf= 4,<br>Criterion= entropy		SMOTE+ TomekLinks	Algorithm=SAMME.R,<br>n_ estimators=178,<br>learning_ rate=1.5
	SMOTE+ ENN	Max _depth=28,<br>Min _samples _split=34,<br>Min _samples _leaf= 14,<br>Criterion= gini		SMOTE+ ENN	Algorithm=SAMME.R,<br>n_ estimators=178,<br>learning_ rate=1.5
SVM	Actual	C=0.8, gamma=0.01,<br>degree=3,kernel=rbf	XG Boost	Actual	n_ estimators=136,<br>learning_ rate=0.05,<br>max_ depth=10,<br>min_ samples_ split=38,<br>max_ features=log2

	SMOTE	C=10, gamma=0.1, degree=4, kernel=rbf		SMOTE	n_ estimators=115, learning_ rate=0.05, max_ depth=26, min_ samples_ split=94, max_ features=sqrt
	ADASYN	C=0.1, gamma=0.1, degree=2, kernel=poly		ADASYN	n_ estimators=178, learning_ rate=0.05, max_ depth=4, min_ samples_ split=90, max_ features=auto
	TomekLinks	C=0.8, gamma=0.1, degree=3, kernel=poly		TomekLinks	n_ estimators=52, learning_ rate=0.05, max_ depth=2, min_ samples_ split=18, max_ features=log2
	ENN	C=0.8, gamma=0.01 degree=4,kernel=poly		ENN	n_ estimators=157, learning_ rate=0.05, max_ depth=24, min_ samples_ split=72, max_ features=auto
	SMOTE+ TomekLinks	C=0.1,gamma=1, degree=4,kernel=poly		SMOTE+ TomekLinks	n_ estimators=73, learning_ rate=0.5, max_ depth=2, min_ samples_ split=16, max_ features=sqrt
	SMOTE+ ENN	C=10,gamma=0.1, degree=4,kernel=rbf		SMOTE+ ENN	n_ estimators=178, learning_ rate=0.05, max_ depth=26, min_ samples_ split=68, max_ features=auto
NN	Actual	Activation=identity, Hidden _layer _sizes = (50, 100), solver= adam , learning _rate= constant	Light GBM	Actual	n_ estimators=200, learning_ rate=0.05, max_ depth=4
	SMOTE	activation=logistic, hidden _layer _sizes=(50,100), solver=adam, learning _rate=constant		SMOTE	n_ estimators=94, learning_ rate=0.05, max_ depth=28
	ADASYN	activation= relu, hidden _layer _sizes=(100,), solver=adam,		ADASYN	n_ estimators=94, learning_ rate=0.05, max_ depth=28

		learning _rate= constant			
	TomekLinks	activation=identity,<br>hidden _layer _sizes= (50, 100),<br>solver= lbfgs,<br>learning _rate= invscaling		TomekLinks	n_ estimators=178,<br>learning_ rate=0.005,<br>max_ depth=14
	ENN	activation= logistic,<br>hidden _layer _sizes=(100,),<br>solver=adam,<br>learning _rate= adaptive		ENN	n_ estimators=178,<br>learning_ rate=0.05,<br>max_ depth=8
	SMOTE+ TomekLinks	activation=relu,<br>hidden_layer_sizes= (100,),<br>solver=adam,<br>learning_rate=constant		SMOTE+ TomekLinks	n_ estimators=94,<br>learning_ rate=0.05,<br>max_ depth=16
	SMOTE+ ENN	activation=tanh,<br>hidden_layer_sizes= (100,),<br>solver=adam,<br>learning_rate= invscaling		SMOTE+ ENN	n_ estimators=157,<br>learning_ rate=0.5,<br>max_ depth=20
KNN	Actual	n_neighbors=15,<br>weights= distance,<br>algorithm= brute,<br>metric=manhattan	CatBoost	Actual	Depth=6,<br>learning_ rate= 0.05,<br>iterations=70,<br>l2_leaf_reg=9
	SMOTE	n_neighbors=9,<br>weights=distance,<br>algorithm=kd_tree,<br>metric=manhattan		SMOTE	Depth=8,<br>learning_ rate= 0.05,<br>iterations=60,<br>l2_leaf_reg=3
	ADASYN	n_neighbors=5,<br>weights=distance,<br>algorithm= auto,<br>metric=manhattan		ADASYN	Depth=10,<br>learning_ rate= 0.05,<br>iterations=60,<br>l2_leaf_reg=5
	TomekLinks	n_neighbors=15,<br>weights=distance,<br>algorithm=kd_tree,<br>metric= manhattan		TomekLinks	Depth=6,<br>learning_ rate= 0.05,<br>iterations=80,<br>l2_leaf_reg=1
	ENN	n_neighbors=7,<br>weights=distance,<br>algorithm=ball_tree,<br>metric= manhattan		ENN	Depth=10,<br>learning_ rate= 0.05,<br>iterations=80,<br>l2_leaf_reg=3
	SMOTE+ TomekLinks	n_neighbors=5,<br>weights=distance,<br>algorithm=auto,<br>metric= manhattan		SMOTE+ TomekLinks	Depth=10,<br>learning_ rate= 0.5,<br>iterations=50,<br>l2_leaf_reg=9
	SMOTE+ ENN	n_neighbors=7,<br>weights=distance,<br>algorithm= auto,<br>metric=manhattan		SMOTE+ ENN	Depth=10,<br>learning_ rate= 0.5,<br>iterations=60,<br>l2_leaf_reg=1
Random Forest	Actual	n_estimators=178,<br>max_features=sqrt,<br>max_depth=20,	GBM	Actual	n_ estimators=94,<br>learning_ rate=0.05,<br>max_ depth=4,

		min_samples_leaf=6, min_samples_split=76			max_ features=log2
	SMOTE	n_estimators=73, max_features=sqrt, max_depth=28, min_samples_leaf=4, min_samples_split=8		SMOTE	n_ estimators=157, learning_ rate=0.005, max_ depth=22, max_ features=log2
	ADASYN	n_estimators=136, max_features= log2, max_depth=18, min_samples_leaf=6, min_samples_split=42		ADASYN	n_ estimators=178, learning_ rate=0.05, max_ depth=16, max_ features=log2
	TomekLinks	n_estimators=157, max_features= auto, max_depth=24, min_samples_leaf=2, min_samples_split=32		TomekLinks	n_ estimators=73, learning_ rate=0.05, max_ depth=2, max_ features= sqrt
	ENN	n_estimators=115, max_features= log2, max_depth=30, min_samples_leaf=6, min_samples_split=4		ENN	n_ estimators=136, learning_ rate=0.05, max_ depth=20, max_ features=log2
	SMOTE+ TomekLinks	n_estimators=94, max_features= auto, max_depth=14, min_samples_leaf=2, min_samples_split=34		SMOTE+ TomekLinks	n_ estimators=200, learning_ rate=0.05, max_ depth=16, max_ features= sqrt
	SMOTE+ ENN	n_estimators=157, max_features= sqrt, max_depth=20, min_samples_leaf=4, min_samples_split=34		SMOTE+ ENN	n_ estimators=178, learning_ rate=0.05, max_ depth=24, max_ features=log2

Classification efficacy and confusion matrix

~~Across nearly all classifiers, models trained on the original imbalanced data exhibited inflated precision and specificity, but severely diminished recall, F1-score, G-mean, Matthews Correlation Coefficient (MCC), and Cohen’s Kappa, reflecting a pronounced bias towards the majority class. Classifiers such as Extra Trees, GBM, and Random Forest particularly struggled with minority class sensitivity, as evidenced by their low recall and F1-scores when trained on unbalanced data (Fig 2). Moreover, the Extra Trees classifier, despite achieving a high accuracy of 0.78 on the~~

imbalanced dataset, recorded a mere F1-score of 0.08, recall of 0.04, and MCC of 0.15 – clearly indicating ineffective detection of the minority class. However, the application of SMOTE+ENN significantly improved its performance, raising the F1-score to 0.94, recall to 0.95, and MCC to 0.79, alongside notable gains in G-mean (0.89) and Cohen's Kappa (0.79). Similar improvements were observed for Random Forest, which improved from an F1-score of 0.12 and recall of 0.07 to 0.47 and 0.73, respectively, under the same balancing strategy. Ensemble models such as CatBoost, XGBoost, and GBM also exhibited marked enhancements in composite metrics and recall following resampling interventions (Table 3).

Table 3: performance of the machine learning algorithms.

Across classifiers, training on the original imbalanced data produced inflated accuracy and specificity but markedly poor minority sensitivity (low recall and F1), evidencing majority-class bias (Table 5; Fig 3). For example, ExtraTrees (Actual) achieved Accuracy = 0.78, Specificity = 1.00, Recall = 0.04 and F1 = 0.08, consistent with its confusion matrix on the original test split (Table 4). After applying SMOTE+ENN, ExtraTrees improved substantially F1 = 0.94, Recall = 0.95, Cohen's κ = 0.79, MCC = 0.79 and G-mean = 0.89 in agreement with the corresponding SMOTE+ENN confusion matrix (Table 3). These matched matrix–metric pairs ensure internal coherence. Cross-model medians (Friedman ranks) indicated that TomekLinks tended to yield the highest Accuracy and Specificity overall (Supplementary Table S2E) whereas on the independent test set ExtraTrees with SMOTE+ENN achieved the highest Accuracy among our models (0.91 vs. 0.78 on the original data), underscoring that balancing improved clinically relevant discrimination.

Table 3. 2×2 confusion matrix of ExtraTrees model predictions integrated with SMOTEENN class balancing technique.

~~Model~~	~~Class balanced technique~~ **Predicted Negative**	~~Precision~~ **Predicted Positive**	~~Recall~~	~~F1-score~~	~~AUC-ROC~~	~~AUC-PR~~	~~Cohens-kappa~~	~~G-mean~~	~~Matthews correlation coefficient~~	~~Accuracy~~	~~Specificity~~
**Actual Negative**	185	27									
**Actual Positive**	20	440									

Table 4. 2×2 confusion matrix of ExtraTrees model predictions integrated with original sample.

	**Predicted Negative**	**Predicted Positive**
**Actual Negative**	518	2
**Actual Positive**	146	6

Table 5: performance of the machine learning algorithms.

**Model**	**Class balancing technique**	**Precision**	**Recall**	**F1-score**	**AUC-ROC**	**AUC-PR**	**Cohens-kappa**	**G-mean**	**Matthews correlation coefficient**	**Accu-racy**	**Specifi-city**
Logistic Regression	actual	0.57	0.21	0.31	0.71	0.48	0.21	0.45	0.24	0.78	0.95
Logistic Regression	smote	0.37	0.62	0.46	0.72	0.54	0.24	0.65	0.26	0.67	0.68
Logistic Regression	adasyn	0.35	0.65	0.46	0.71	0.54	0.22	0.65	0.25	0.64	0.64
Logistic Regression	tomeklink	0.36	0.67	0.47	0.72	0.55	0.24	0.66	0.27	0.65	0.64

Deleted Cells
Formatted: Justified, Line spacing: Double
Deleted Cells
Deleted Cells
Deleted Cells
Formatted Table
Formatted: Font: 12 pt, Not Bold, Font color: Auto
Deleted Cells
Deleted Cells
Deleted Cells
Deleted Cells
Deleted Cells
Formatted: Font: 12 pt, Font color: Auto
Formatted: Font: 12 pt, Font color: Auto

Logistic Regression	enn	0.38	0.57	0.45	0.71	0.52	0.24	0.64	0.25	0.68	0.72
Logistic Regression	smote+tomek	0.34	0.62	0.44	0.71	0.53	0.21	0.63	0.23	0.64	0.65
Logistic Regression	smote+enn	0.30	0.80	0.44	0.70	0.57	0.16	0.60	0.22	0.53	0.45
Decision Tree	actual	0.55	0.26	0.35	0.70	0.49	0.24	0.49	0.26	0.78	0.94
Decision Tree	smote	0.40	0.34	0.37	0.63	0.45	0.20	0.54	0.20	0.73	0.85
Decision Tree	adasyn	0.34	0.31	0.32	0.61	0.40	0.13	0.50	0.13	0.70	0.82
Decision Tree	tomeklink	0.49	0.35	0.41	0.69	0.50	0.27	0.56	0.27	0.77	0.89
Decision Tree	enn	0.37	0.56	0.45	0.70	0.52	0.23	0.63	0.24	0.68	0.72
Decision Tree	smote+tomek	0.30	0.27	0.28	0.60	0.36	0.08	0.46	0.08	0.69	0.81
Decision Tree	smote+enn	0.34	0.72	0.46	0.69	0.56	0.21	0.64	0.25	0.61	0.57
Neural network	actual	0.53	0.19	0.28	0.71	0.45	0.18	0.42	0.21	0.77	0.95
Neural network	smote	0.30	0.31	0.31	0.59	0.38	0.09	0.49	0.09	0.67	0.78
Neural network	adasyn	0.30	0.29	0.29	0.60	0.38	0.09	0.48	0.09	0.68	0.80
Neural network	tomeklink	0.54	0.26	0.35	0.71	0.48	0.24	0.49	0.26	0.78	0.93
Neural network	enn	0.37	0.59	0.45	0.70	0.53	0.24	0.64	0.25	0.67	0.70
Neural network	smote+tomek	0.32	0.32	0.32	0.61	0.40	0.12	0.51	0.12	0.69	0.80
Neural network	smote+enn	0.33	0.71	0.45	0.69	0.55	0.20	0.63	0.23	0.60	0.56
Knn	actual	0.45	0.18	0.26	0.68	0.41	0.15	0.41	0.17	0.76	0.94
Knn	smote	0.33	0.44	0.38	0.66	0.45	0.15	0.57	0.16	0.66	0.73
Knn	adasyn	0.33	0.42	0.37	0.64	0.44	0.15	0.56	0.15	0.67	0.74
Knn	tomeklink	0.43	0.20	0.28	0.68	0.41	0.15	0.43	0.17	0.75	0.92
Knn	enn	0.36	0.58	0.44	0.67	0.52	0.22	0.63	0.23	0.66	0.69
Knn	smote+tomek	0.33	0.41	0.36	0.64	0.44	0.15	0.55	0.15	0.67	0.75
Knn	smote+enn	0.32	0.76	0.45	0.67	0.56	0.18	0.62	0.23	0.57	0.51
Random Forest	actual	0.72	0.07	0.12	0.71	0.50	0.09	0.26	0.17	0.78	0.99
Random Forest	smote	0.45	0.33	0.38	0.69	0.47	0.23	0.54	0.24	0.75	0.88
Random Forest	adasyn	0.42	0.42	0.42	0.71	0.49	0.25	0.59	0.25	0.73	0.82
Random Forest	tomeklink	0.56	0.18	0.28	0.70	0.47	0.18	0.42	0.22	0.78	0.96
Random Forest	enn	0.37	0.58	0.46	0.71	0.53	0.24	0.64	0.25	0.68	0.71
Random Forest	smote+tomek	0.42	0.38	0.40	0.71	0.47	0.23	0.57	0.23	0.73	0.84
Random Forest	smote+enn	0.34	0.73	0.47	0.71	0.57	0.22	0.65	0.26	0.61	0.58
Extra tree	actual	0.80	0.04	0.08	0.71	0.53	0.06	0.20	0.15	0.78	1.00
Extra tree	smote	0.39	0.52	0.45	0.70	0.51	0.25	0.63	0.25	0.71	0.76
Extra tree	adasyn	0.41	0.40	0.40	0.70	0.47	0.23	0.58	0.23	0.73	0.82
Extra tree	tomeklink	0.57	0.20	0.29	0.70	0.47	0.20	0.44	0.24	0.78	0.95
Extra tree	enn	0.35	0.57	0.43	0.70	0.51	0.21	0.62	0.22	0.66	0.69
Extra tree	smote+tomek	0.42	0.47	0.44	0.70	0.50	0.26	0.61	0.26	0.73	0.80

Extra tree	smote+enn	0.92	0.95	0.94	0.95	0.95	0.79	0.89	0.79	0.91	0.83
Adaboost	actual	0.57	0.18	0.28	0.71	0.47	0.19	0.42	0.23	0.78	0.96
Adaboost	smote	0.50	0.33	0.40	0.71	0.49	0.27	0.55	0.27	0.77	0.90
Adaboost	adasyn	0.48	0.22	0.30	0.70	0.44	0.18	0.45	0.21	0.77	0.93
Adaboost	tomeklink	0.55	0.28	0.37	0.71	0.49	0.25	0.51	0.27	0.78	0.93
Adaboost	enn	0.38	0.58	0.46	0.71	0.53	0.24	0.64	0.26	0.68	0.71
Adaboost	smote+tomek	0.52	0.23	0.32	0.70	0.46	0.21	0.47	0.23	0.77	0.93
Adaboost	smote+enn	0.35	0.62	0.45	0.71	0.53	0.22	0.64	0.24	0.65	0.66
XG Boost	actual	0.43	0.25	0.32	0.66	0.43	0.18	0.47	0.19	0.75	0.90
XG Boost	smote	0.43	0.29	0.35	0.65	0.44	0.20	0.51	0.20	0.75	0.88
XG Boost	adasyn	0.54	0.29	0.37	0.71	0.50	0.26	0.51	0.28	0.78	0.93
XG Boost	tomeklink	0.52	0.09	0.15	0.71	0.41	0.09	0.29	0.14	0.77	0.98
XG Boost	enn	0.34	0.60	0.44	0.67	0.52	0.20	0.63	0.22	0.64	0.65
XG Boost	smote+tomek	0.55	0.26	0.35	0.70	0.49	0.24	0.49	0.27	0.78	0.94
XG Boost	smote+enn	0.35	0.64	0.45	0.70	0.54	0.22	0.64	0.24	0.64	0.64
LGBM	actual	0.56	0.25	0.35	0.70	0.49	0.24	0.48	0.26	0.78	0.94
LGBM	smote	0.48	0.30	0.37	0.70	0.47	0.23	0.52	0.24	0.76	0.90
LGBM	adasyn	0.47	0.28	0.35	0.70	0.46	0.21	0.50	0.22	0.76	0.91
LGBM	tomeklink	0.50	0.03	0.06	0.71	0.38	0.03	0.17	0.08	0.77	0.99
LGBM	enn	0.34	0.56	0.42	0.68	0.50	0.19	0.61	0.20	0.65	0.67
LGBM	smote+tomek	0.48	0.27	0.35	0.70	0.46	0.22	0.50	0.23	0.76	0.91
LGBM	smote+enn	0.34	0.63	0.45	0.69	0.53	0.21	0.64	0.23	0.64	0.64
CatBoost	actual	0.58	0.11	0.18	0.71	0.45	0.12	0.32	0.18	0.78	0.98
CatBoost	smote	0.46	0.37	0.41	0.71	0.49	0.26	0.57	0.26	0.75	0.87
CatBoost	adasyn	0.48	0.34	0.40	0.71	0.49	0.26	0.55	0.26	0.76	0.89
CatBoost	tomeklink	0.59	0.21	0.31	0.70	0.49	0.21	0.45	0.25	0.78	0.96
CatBoost	enn	0.38	0.61	0.47	0.71	0.54	0.26	0.66	0.27	0.68	0.70
CatBoost	smote+tomek	0.40	0.29	0.34	0.66	0.43	0.18	0.50	0.18	0.74	0.87
CatBoost	smote+enn	0.35	0.67	0.46	0.70	0.55	0.22	0.65	0.25	0.63	0.62
GBM	actual	0.63	0.17	0.27	0.71	0.50	0.19	0.41	0.25	0.79	0.97
GBM	smote	0.46	0.25	0.32	0.68	0.44	0.19	0.48	0.21	0.76	0.91
GBM	adasyn	0.47	0.24	0.32	0.69	0.44	0.19	0.47	0.20	0.76	0.92
GBM	tomeklink	0.58	0.13	0.21	0.71	0.45	0.14	0.35	0.19	0.78	0.97
GBM	enn	0.36	0.59	0.45	0.69	0.52	0.22	0.63	0.24	0.66	0.68
GBM	smote+tomek	0.46	0.26	0.33	0.69	0.45	0.20	0.49	0.21	0.76	0.91
GBM	smote+enn	0.32	0.76	0.45	0.69	0.57	0.19	0.63	0.23	0.58	0.52
SVM	actual	0.68	0.07	0.12	0.67	0.48	0.08	0.26	0.16	0.78	0.99
SVM	smote	0.35	0.04	0.07	0.64	0.30	0.03	0.20	0.05	0.76	0.98
SVM	adasyn	0.31	0.45	0.37	0.62	0.44	0.13	0.56	0.13	0.64	0.70
SVM	tomeklink	0.35	0.37	0.36	0.62	0.44	0.17	0.54	0.17	0.70	0.80
SVM	enn	0.41	0.08	0.13	0.67	0.35	0.06	0.27	0.09	0.76	0.97
SVM	smote+tomek	0.34	0.37	0.35	0.61	0.43	0.15	0.54	0.15	0.69	0.79
SVM	smote+enn	0.31	0.34	0.32	0.63	0.40	0.11	0.51	0.11	0.67	0.77

Formatted: Font: 12 pt

Fig ~~2~~3: Performance of the machine learning algorithms.

Global comparison of class-balancing techniques

ML algorithms ~~exhibit~~exhibited varying ~~performances~~performance across ~~different class-~~balancing techniques, ~~making it challenging to identify the most suitable one for optimal scores. Employing~~motivating formal statistical ~~tests can aid in selecting the appropriate technique. Furthermore, except~~ testing. Except for AUC-PR, ~~the majority of~~ most metrics ~~display~~were non-normal ~~distributions, leading to the rejection of the null hypothesis at a significance level of 5%. This implies that~~by Anderson–Darling (Supplementary Table S2A), so ANOVA ~~tests should not be applied to these metrics excluding~~ was restricted to AUC-PR ~~(Table 4).~~

~~Table 5 indicates a~~. Repeated-measures ANOVA on AUC-PR showed significant ~~difference (P<0.001) among specific class-balancing techniques. Notably, for the AUC-PR metric,~~differences across techniques (Supplementary Table S2B), and Tukey's HSD identified SMOTE+ENN and ENN ~~techniques emerge~~ as significantly superior ~~to others (Table 6). Additionally, for other metrics, we utilized the Friedman test instead of ANOVA to compare class-~~

Formatted: Font: Not Bold

Formatted: Font: Not Bold

Formatted: Font: +Body (Calibri), 11 pt, Font color: Auto, Pattern: Clear

Formatted: Font: +Body (Calibri), 11 pt, Font color: Auto, Pattern: Clear

Formatted: Font: +Body (Calibri), 11 pt, Font color: Auto, Pattern: Clear

Formatted: Font: +Body (Calibri), 11 pt, Font color: Auto, Pattern: Clear

Formatted: Font: +Body (Calibri), 11 pt, Font color: Auto, Pattern: Clear

Formatted: Font: +Body (Calibri), 11 pt, Font color: Auto, Pattern: Clear

Formatted: Font: +Body (Calibri), 11 pt, Font color: Auto, Pattern: Clear

Formatted: Font: +Body (Calibri), 11 pt, Font color: Auto, Pattern: Clear

Formatted: Font: +Body (Calibri), 11 pt, Font color: Auto, Pattern: Clear

Formatted: Font: +Body (Calibri), 11 pt, Font color: Auto, Pattern: Clear

Formatted: Normal (Web)

balancing techniques.for AUC-PR in multiple pairwise contrasts (Supplementary Table S2C). For the remaining non-normal metrics, Friedman tests showed significant omnibus differences (Supplementary Table S2D). Post-hoc rank summaries indicated SMOTE+ENN led G-mean, Recall, and F1 (ENN second) while TomekLinks ranked highest for Accuracy, Specificity, Precision, and AUROC (Supplementary Table S2E).

Table 4: The results of the Anderson-Darling normality test.

Boxplots of AUCPR, F1, Recall, Precision, Specificity, G-mean, Accuracy and AUC-ROC (Fig 4–11) illustrate these trends: SMOTE+ENN achieved the highest Recall, F1 and AUC-PR, whereas the original data scored highest in Accuracy and Specificity.

Metric	Test Statistic	p-value	
Matthews Correlation Coefficient	3.1817	<0.05	
Cohen's kappa	2.4338	<0.05	
AUCPR	0.31492	0.5374	
Accuracy	3.1183	<0.05	
G-mean	2.3604	<0.05	
Specificity	1.9971	<0.05	
Recall	1.4823	<0.05	
Precision	2.3018	<0.05	
Fig 4. The Boxplot of the original data and class balancing techniques based on AUC-PR.	F1 scoreFig 5. The Boxplot of the original data and class balancing techniques based on F1 scores.	2.7472	<0.05
AUCROC	6.8793	< 0.05	

Table 5: One-way repeated measure ANOVA.

DFFig 6. The Boxplot of the original data and class balancing	Sum of squareFig 7. The Boxplot of the original data and class balancing	Mean square	F value	P value	

Deleted Cells

Deleted Cells

Inserted Cells

Formatted Table

Formatted: Font: 12 pt

Formatted: Font: 12 pt

Deleted Cells

Formatted Table

Deleted Cells

Deleted Cells

Deleted Cells

	techniques based on Recall.	techniques based on Precision.			
AUCPR	6	0.08431	0.014052	9.532	<0.05

Formatted: Font: 12 pt

Formatted: Font: 12 pt

Formatted: Font: 12 pt

Table 6: SMOTE family mean comparison for AUC-PR using Tukey's HSD test.

Formatted Table

Deleted Cells

Deleted Cells

Fig 8. The Boxplot of the original data and class balancing techniques based on Specificity.	Mean differenceFig 9. The Boxplot of the original data and class balancing techniques based on G-mean.	95% Confidence interval		
		Lower bound	Upper bound	P-value
Actual - (SMOTE+ENN)	-0.06974	-0.11741	-0.022067	0.0006
ADASYN- ENN	-0.04852	-0.09619	-0.000851	0.0434
ADASYN - (SMOTE+ENN)	-0.08606	-0.13373	-0.038395	<.0001
ENN - SMOTE	0.05240	0.00473	0.100066	0.0221
ENN - (SMOTE+Tomek)	0.05374	0.00607	0.101405	0.0173
SMOTE - (SMOTE+ENN)	-0.08994	-0.13761	-0.042272	<.0001
(SMOTE+ENN) - (SMOTE+Tomek)	0.09128	0.04361	0.138949	<.0001
(SMOTE+ENN) - Tomek	0.08139	0.03372	0.129057	<.0001

Formatted: Font: 12 pt

The findings from the Friedman test, as shown in Table 7, indicate that the Matthews Correlation Coefficient and Cohen's kappa suggest that the class-balancing techniques may not differ one from another. However, other results suggest that at least one technique performs differently from the rest.

From Table 8, it can be concluded that,

- Among the G-mean, Recall, and F1 scores, the SMOTE+ENN technique appears to be the most effective, followed by ENN as the second most effective.
- For Accuracy and Precision, AUC-ROC and Specificity TomekLinks could be more effective than other techniques.

Table 7: The results of the Friedman test.

Friedman tests	Chi-squared score	DF	p-value
Matthews Correlation Coefficient	4.8929	6	0.5576
Cohen's kappa	7	6	0.3208
AUCPR	31.179	6	<0.05
AccuracyFig 10. The Boxplot of the original data and class balancing techniques based on Accuracy.	52.031Fig 11. The Boxplot of the original data and class balancing techniques based on AUCROC.	6	<0.05
G-mean	39.78	6	<0.05
Recall	46.929	6	<0.05
F1 score	36.75	6	<0.05
AUCROC	25.036	6	<0.05
Specificity	52.831	6	<0.05
Precision	43.964	6	<0.05

Table 8: Additional information from Friedman test result.

	RANK	RESAMPLING TECHNIQUES	SUM OF RANKS	MEDIAN
Accuracy	1	TomekLinks	66	0.775
	2	SMOTE	47.5	0.75
	3	SMOTE+ TomekLinks	45.5	0.73
	4	ADASYN	44.5	0.73
	5	ENN	26.5	0.675
	6	SMOTE+ENN	13.5	0.61
G-mean	1	SMOTE+ ENN	60	0.64
	2	ENN	58	0.63
	3	SMOTE+ TomekLinks	42.5	0.505
	4	SMOTE	40.5	0.54
	5	ADASYN	34.5	0.53
	6	TomekLinks	26.5	0.445
Recall	1	SMOTE+ ENN	69	0.717
	2	ENN	53	0.582
	3	SMOTE	39	0.332
	4	ADASYN	35	0.324
	5	SMOTE+ TomekLinks	31	0.306
	6	TomekLinks	25	0.207
F1 score	1	SMOTE+ ENN	61	0.450
	2	ENN	56	0.446
	3	SMOTE	39	0.374
	4	ADASYN	35	0.366
	5	SMOTE+ TomekLinks	32	0.350

Formatted Table

Deleted Cells

Deleted Cells

	6	TomekLinks	29	0.301
AUROC	1	TomekLinks	57	0.705
	2	ENN	52	0.698
	3	SMOTE	41	0.685
	4	SMOTE+ ENN	40	0.696
	5	ADASYN	33	0.699
	6	SMOTE+ TomekLinks	29	0.693
Precision	1	TomekLinks	67	0.526
	2	SMOTE	45	0.414
	3	ADASYN	41	0.412
	3	SMOTE+ TomekLinks	41	0.410
	5	ENN	38	0.370
	6	SMOTE+ ENN	19	0.337
Specificity	1	TomekLinks	64.5	0.94
	2	SMOTE+ TomekLinks	47	0.825
	3	ADASYN	46.5	0.82
	4	SMOTE	45	0.875
	5	ENN	30.5	0.7
	6	SMOTE+ ENN	13.5	0.575

We investigated whether various machine learning approaches exhibit significant differences and how different class-balancing techniques enhance performance. Through plots of multiple performance metrics (Fig 3, 4, 5, 6,7,8,9 and 10) including AUCPR, F1 score, Recall, Precision, Specificity, G-mean, Accuracy, and AUC-ROC, we discerned trends. The plots revealed that the SMOTE+ENN technique yielded the highest values for Recall, F1 score, and AUC PR. Conversely, accuracy and specificity attained the highest scores for the original dataset. These observations underscore the impact of class-balancing techniques on improving specific performance metrics in machine learning models, highlighting the importance of selecting appropriate techniques based on the desired outcome.

Fig 3. The Boxplot of the original data and class balancing techniques based on AUC-PR.

Fig 4. The Boxplot of the original data and class balancing techniques based on F1 scores.

Fig 5. The Boxplot of the original data and class balancing techniques based on Recall.

Fig 6. The Boxplot of the original data and class balancing techniques based on Precision.

Fig 7. The Boxplot of the original data and class balancing techniques based on Specificity.

Fig 8. The Boxplot of the original data and class balancing techniques based on G-mean.

Fig 9. The Boxplot of the original data and class balancing techniques based on Accuracy.

Fig 10. The Boxplot of the original data and class balancing techniques based on AUCROC.

Formatted: Font: 16 pt

Feature importance

Formatted: Line spacing: Multiple 1.08 li

The integration of SHapley Additive exPlanations (SHAP) with a trained machine learning model enabled the comprehensive analysis of both local and global predictors associated with hypertension in married women in Bangladesh. Figure 11a demonstrates the most important factors associated with married women's hypertension in Bangladesh and Figure 13b reveals the top 20 factors stratified by a positive and negative association with married women hypertension in Bangladesh. However, in Figure 11b, red bars indicate positive associations, while blue bars represent negative associations with hypertension among married women in Bangladesh. Additionally, Figure 11c illustrates the SHAP summary plot that integrated feature importance with feature effects for all participants. The plotted points demonstrate the influence each feature has on individual outcomes within the dataset. The features were arranged along the y-axis in descending order based on their importance. The feature values were represented by color, with higher values appearing redder. Data points concentrated near the vertical line at zero on the Shapley value axis indicate that these predictors have minimal impact on the outcome.

Figure 11a identifies a respondent'sIntegrating SHAP with a trained model enabled analysis of global and local predictors. Figure 12a shows the most important features; Fig 12b classifies the

top 20 factors by direction of association; Fig 12c presents a SHAP summary plot combining feature importance and effects.

In Fig 12a, respondent age below <35 years aswas the most significant factor associated with hypertension among married women in Bangladesh. The second, third, and fourth most influential factors include being factor, followed by overweight, having given birth to two orto three ever-born children, and the husband or /partner attaining awith secondary education level. Other notable. Additional contributors ranked sixth, seventh, and eighth are beingincluded underweight, having a normal weight, and the husband or /partner being younger than <35 years. The ninth, tenth, and eleventh factors include having more than two , >2 living children, no children born births in the last five years, and obesity (Fig 11a)..

Figure 11b further reveals the direction of these associations. Factors such as a respondent's current Fig 12b indicates negative associations (blue) for age below <35 years, being, underweight or of /normal weight, having a husband or /partner under <35 years of, respondent age, being between 35–40 years old, having, one child in the last five years, normoglycemia, residingresidence in the Rajshahi division, and the absence ofno formal education for the husband or /partner are negatively associated with hypertension, as indicated by blue bars. In contrast, positive associations, marked by red bars, include being. Positive associations (red) included overweight, giving birthtwo to two or three ever-born children, the husband or /partner having attained with secondary education, having more than two>2 living children, having no childrenbirths in the last five years, obesity, the husband or /partner being over >40 years old, using, traditional contraceptive methods, unmet contraceptive needs (for limiting), richest and an age difference of less than 10 years between the respondent and their spouse (Fig 11b).

Formatted: Normal (Web)

Formatted: Font: +Body (Calibri), 11 pt

Formatted: Font: +Body (Calibri), 11 pt

Formatted: Font: +Body (Calibri), 11 pt

Formatted: Font: +Body (Calibri), 11 pt

Formatted: Font: +Body (Calibri), 11 pt

Formatted: Font: +Body (Calibri), 11 pt

Formatted: Font: +Body (Calibri), 11 pt

Formatted: Font: +Body (Calibri), 11 pt

Formatted: Font: +Body (Calibri), 11 pt

Formatted: Font: +Body (Calibri), 11 pt

Formatted: Font: +Body (Calibri), 11 pt

Formatted: Font: +Body (Calibri), 11 pt

Formatted: Font: +Body (Calibri), 11 pt

Formatted: Font: +Body (Calibri), 11 pt

Formatted: Font: +Body (Calibri), 11 pt

Formatted: Font: +Body (Calibri), 11 pt

Formatted: Font: +Body (Calibri), 11 pt

Formatted: Font: +Body (Calibri), 11 pt

Formatted: Font: +Body (Calibri), 11 pt

Formatted: Font: +Body (Calibri), 11 pt

Formatted: Font: +Body (Calibri), 11 pt

Formatted: Font: +Body (Calibri), 11 pt

Formatted: Font: +Body (Calibri), 11 pt

Formatted: Font: +Body (Calibri), 11 pt

Formatted: Font: +Body (Calibri), 11 pt

Formatted: Font: +Body (Calibri), 11 pt

Formatted: Font: +Body (Calibri), 11 pt

Formatted: Font: +Body (Calibri), 11 pt

Formatted: Font: +Body (Calibri), 11 pt

Formatted: Font: +Body (Calibri), 11 pt

Formatted: Font: +Body (Calibri), 11 pt

Formatted: Font: +Body (Calibri), 11 pt

Figure 11c highlights that a respondent's current age is below 35, useUnderweight, Normal weight, husband/partner's age is below 35 years, respondent age is between 35-40 years, one child in last five years, Normoglycemia, administrative division in Bangladesh (Rajshahi), absence of formal education of husband or partners are associated with low Shapley values (colored in blue) and reduced likelihood of being predicted as having hypertension. Overweight, ever born two to three children, education secondary level education of husband or partners, number of living children more than two, no children in last five years, obese, husband/partner's age 40 years, traditional method of contraceptive method, unmet need for contraception (using for limiting), unmet need for contraception (using for limiting), age difference with husband's/partners less than 10 years are associated with high Shapley values (colored in red or purple) and increased likelihood of being predicted as having hypertension among married women in Bangladesh (Fig 11c). limiting, richest quintile and age difference ≥10 years.

Formatted: Normal (Web)

Fig 12c shows these effects at the individual level: blue points (low SHAP values) correspond to features decreasing predicted risk, while red/purple points (high SHAP values) correspond to features increasing predicted risk.

Fig 11a. The factors importance of hypertension among married women in Bangladesh. recuag_1= respondent's current age below 35,bmiclass_3= Overweight,tchildborn_2= ever born two to three children,v701_2= education secondary level education of husband or partners,bmiclass_1= Underweight,bmiclass_3= Normal weight,husagec_1= husband/partner's age below 35 years,livingchild_3= number of living children more than two,birthlfyr_0= no children in last five years,bmiclass_3=

Fig 11b. The classification of positive and negative predictors of hypertension of married women in Bangladesh. recuag_1= respondent's current age below 35,bmiclass_3= Overweight,tchildborn_2= ever born two to three children,v701_2= education secondary level education of husband or partners,bmiclass_1= Underweight,bmiclass_3= Normal weight,husagec_1= husband/partner's age below

Obese,recuag_2= respondent age between 35-40 years,birthlfyr_1= One children in last five years,husagec_3= husband/partner's age 40 years,v313_3= traditional method of contraceptive method,v626a_4= unmet need for contraception (using for limiting),diabclass_w _2= Normoglycemia,v190_3=richest,v024_6= administrative division in Bangladesh (Rajshahi)

Fig 12a. The factors importance of hypertension among married women in Bangladesh.

35 years,livingchild_3= number of living children more than two,birthlfyr_0= no children in last five years,bmiclass_3= Obese,recuag_2= respondent age between 35-40 years,birthlfyr_1= One children in last five years,husagec_3= husband/partner's age 40 years,v313_3= traditional method of contraceptive method,v626a_4= unmet need for contraception (using for limiting), diabclass_w _2= Normoglycemia,v190_3=richest,v024_6= administrative division in Bangladesh (Rajshahi),agedif_0= age difference with husband's/partners less than 10 years,v701_0= absence of formal education of husband or partners

Fig 12b. The classification of positive and negative predictors of hypertension of married women in Bangladesh.

Formatted: Font: 12 pt

Formatted: Font: 12 pt

Formatted: Font: 12 pt

Fig 11c. The impact of the factors of married women's hypertension in Bangladesh. recuag_1= respondent's current age below 35,bmiclass_3= Overweight,tchildborn_2= ever born two to three children,v701_2= education secondary level education of husband or partners,bmiclass_1= Underweight,bmiclass_3= Normal weight,husagec_1= husband/partner's age below 35 years,livingchild_3= number of living children more than two,birthlfyr_0= no children in last five years,bmiclass_3= Obese,recuag_2= respondent age between 35-40 years,birthlfyr_1= One children in last five years,husagec_3= husband/partner's age 40 years,v313_3= traditional method of contraceptive method,v626a_4= unmet need for contraception (using for limiting),diabclass_w _2= Normoglycemia,v190_3=richest,v024_6= administrative division in Bangladesh (Rajshahi),agedif_0= age difference with husband's/partners less than 10 years,v701_0= absence of formal education of husband or partners

Fig 12c. The impact of the factors of married women's hypertension in Bangladesh.

Internal overfitting diagnostics

Repeated stratified five-fold CV yielded an average F1 of 0.934 ± 0.012; nested CV showed an outer-fold mean F1 of 0.965 ± 0.010; the held-out test F1 was 0.9447 (Supplementary Table S3A). Learning-curve behavior showed convergence between training and validation performance as sample size increased, supporting strong generalization while acknowledging that optimism is possible without external validation (S1 Fig).

Test-set performance with bootstrap confidence intervals

On the independent test set (n = 672), ExtraTrees + SMOTE+ENN achieved Precision 0.92 (95% CI 0.90–0.94), Recall 0.95 (0.93–0.97), F1 0.94 (0.92–0.96), Accuracy 0.91 (0.89–0.94), Specificity 0.83 (0.80–0.87), ROC-AUC 0.95 (0.93–0.97), PR-AUC 0.95 (0.93–0.97), Cohen's κ 0.79 (0.75–0.83), MCC 0.79 (0.75–0.83) and G-mean 0.89 (0.86–0.92) (Table 6).

Table 6: Test-set performance of ExtraTrees with SMOTE+ENN using 1,000-replicate nonparametric bootstrap (n = 672)

**Metric**	**Point estimate**	**95% CI**
Precision	0.92	0.90 – 0.94
Recall	0.95	0.93 – 0.97
F1-score	0.94	0.92 – 0.96
Accuracy	0.91	0.89 – 0.94
Specificity	0.83	0.80 – 0.87
ROC-AUC	0.95	0.93 – 0.97
PR-AUC	0.95	0.93 – 0.97
Cohen's κ	0.79	0.75 – 0.83
MCC	0.79	0.75 – 0.83
G-mean	0.89	0.86 – 0.92

Multicollinearity

Diagnostics indicated structurally inflated VIFs under one-hot encoding. Correlation screening revealed expected dependencies (e.g., respondent age <35 vs. husband age ≥40, ρ = −0.82; mutually exclusive recent-birth categories, ρ = −0.89), along with dependencies among BMI categories, parity, and living-children counts. These reflect structural collinearities inherent to categorical coding. Tree-based models such as ExtraTrees are robust to these correlations and predictive performance was unaffected (Supplementary Table S5).

Sample-flow transparency and class balance across stages

A CONSORT-style accounting of the analytic sample is shown in Supplementary Table S4A. No rows were lost to missingness; subsequent splits yielded the training and test sets used for modeling. Stratified 5-fold CV preserved the original training prevalence (23.1% hypertensive) in each fold (Supplementary Table S4B). Class balancing (e.g., SMOTE+ENN) was applied inside the training portion of each fold only; the final ExtraTrees model was refit on the full training set using SMOTE+ENN, yielding a minority-dominant training balance (Supplementary Table S4C). The independent test set (n=672) remained untouched.

Calibration and thresholding

The ExtraTrees model was well calibrated (Brier score = 0.0626; S2 Fig). The Youden-optimal decision threshold was 0.55, at which Youden's J was 0.859 (Supplementary Table S3B). Unless noted, confusion matrices and test-set metrics were computed at threshold 0.55.

Threshold consistency: Unless noted, the confusion matrix and all bootstrap test-set metrics were computed at the 0.55 decision threshold.

Fairness / stratified performance

Subgroup analyses were generally strong, though some divisions showed slightly lower Accuracy and F1 despite perfect discrimination (ROC-AUC = 1.0). For example, in Rajshahi, Accuracy and F1 were modestly reduced relative to overall performance (Supplementary Table S6).

Discussion

This study systematically evaluated 12 machine-learning (ML) algorithms with six class balancing strategies, integrating SHAP (SHapley Additive exPlanations) to identify factors associated with hypertension among married women in Bangladesh. Extra Trees combined with SMOTE+ENN (Synthetic Minority Over-sampling Technique + Edited Nearest Neighbors) achieved the best performance (F1 = 0.94; AUC-PR = 0.95). Gains were largest for recall, F1, G-mean and AUC-PR metrics better suited to imbalanced data while precision, specificity, accuracy and AUC-ROC were comparatively stable across samplers (Figs 4–11).

Our findings align with global work yet add context-specific insights. An Ethiopian stacking/XGBoost model reported slightly higher performance (F1 = 96.5%, AUC = 0.97) on a much smaller sample (n = 612) emphasizing clinical/lifestyle predictors (97). In contrast, our socially grounded features parity, spousal education, contraceptive use performed strongly in a nationally representative cohort. Similarly, studies from Malaysia, South Korea, Japan and Norway reported AUCs ≈0.78–0.87 using largely clinical markers, whereas our sociodemographic model reached AUC-ROC = 0.95 (37, 98-100). Innovative approaches using wearable ECG (AUC = 0.83) or echocardiography (AUC = 0.87) show promise but have limited population-level generalizability (101, 102). A quantum-enhanced ML study reported very high scores (F1 = 98.9%,

accuracy = 98.4%), but our results underscore that carefully tuned classical ML remains highly effective and interpretable for population-specific public-health applications (103). Quantitatively, our AUC-ROC exceeded several large clinical models by +0.08 to +0.17 absolute AUC (0.95 vs. 0.78–0.87) and our accuracy was +13 percentage points higher than Asadullah et al. (91% vs. 78%) (70).

Why emphasize PR over ROC? Precision–recall curves are more informative when the positive class is rare and accuracy can be misleadingly high due to the majority class; F1 and AUC-PR therefore provide a truer picture of minority-class performance (104-108). Across algorithms, ensembles especially Extra Trees with SMOTE+ENN were most effective for imbalanced health data, consistent with prior reports of SMOTE+ENN paired with Random Forest, XGBoost, LightGBM, or stacked models (109-113). The analytic sample (N = 4,253; ~23.1% hypertensive) provided sufficient positive events for supervised learning. Although several sociodemographic variables are correlated (e.g., age, parity, recent births; BMI categories), tree-based ensembles are robust to redundancy; sensitivity analyses that dropped one variable per correlated cluster left the test F1 unchanged within bootstrap uncertainty, supporting stability while preserving program-relevant interpretability.

SHAP analyses consistently highlighted age, parity, recent births, contraceptive use and spousal education as influential predictors (Figs 12a–c). Younger age (<35 years) was protective, whereas women aged 35–40 or with husbands ≥40 years showed higher risk. The positive association with husbands' secondary education counter to expectation may reflect socioeconomic stressors or confounding with wealth and fertility; this hypothesis warrants social-epidemiological follow-up. Our results corroborate prior Bangladesh-based work linking contraceptive use, women's age,

husband's education and number of living children to hypertension risk, though earlier studies did not focus specifically on married women (114-117)

Given high recall at the Youden-optimal threshold (0.55), health systems could embed risk scoring in digital registers (e.g., eRegistries) and during family-planning or postpartum visits to triage married women for blood-pressure checks. Three actionable levers emerge: (i) integrate BP screening into contraceptive counselling and postpartum follow-up; (ii) engage husbands/partners via brief education or SMS nudges where partner age/spousal education elevate risk; and (iii) target high-parity households for home BP monitoring and referral. Geographic gradients (e.g., higher risk in Rajshahi) support district-tailored outreach. Diet-focused strategies (e.g., green coffee supplementation; DASH/Mediterranean patterns) offer complementary, population-specific interventions (118, 119).

SHAP values explain model associations, not causal effects; correlated social and fertility indicators may act as proxies. Apparent positive associations (e.g., husbands' secondary education) could reflect unmeasured confounding (wealth, occupation, stress). Ethical deployment should address fairness (monitor subgroup performance gaps), transparency (document model versioning and thresholds) and potential unintended consequences (e.g., stigma or resource diversion). Thresholds should be calibrated to local prevalence and the relative costs of false negatives vs. false positives.

Internal checks repeated CV, nested CV and bootstrap Cis showed stable performance with tight uncertainty (see Results); however, training solely on BDHS-2017-18 may still inflate apparent performance. We have pre-specified external validation on BDHS-2022, applying identical preprocessing and the pre-set decision threshold (see Methods) to evaluate discrimination,

calibration and transportability. This will test robustness in a later, post-COVID cohort and under any instrument changes. Findings do not generalize to unmarried women.

Conclusion

The study has employed an integrated approach utilizing machine learning (ML) models along with the SHAP (SHapley Additive exPlanations) method applying class-balancing techniques to identify and predict associated factors of hypertension in married women in Bangladesh. By way of trial and error, it was observed that the extra tree for SMOTE+ENN (Synthetic Minority Over-sampling Technique + Edited Nearest Neighbors) class balanced technique revealed the highest F1-score and Area Under the Curve for Precision-Recall (AUC-PR) values, established its efficacy in the classification of hypertension predictors (Fig. 2). However, in contrast to the use of class balanced techniques, it emerged that the measurements related to Precision, Specificity, Accuracy, and Area Under the Receiver Operating Characteristic Curve (AUC-ROC) remained unchanged (Fig. 8, 9, 11, and 12). On the contrary, notable progress was noted in metrics such as AUC-PR, F1-score, Recall, G-mean, and, particularly notable with the SMOTE+ENN technique which achieved the highest scores in F1-score, Recall, and AUC-PR (Fig. 5,6, 7 and 10).

A study conducted in Ethiopia employing a stacking ensemble with XGBoost to predict hypertension achieved marginally higher performance (F1-score: 96.48%, AUC: 0.971) on a smaller dataset of 612 adults, whereas our Extra Trees model combined with SMOTE+ENN attained an F1-score of 94% and AUC of 0.95. Notably, while both studies used SHAP for interpretability, the Ethiopian model emphasized clinical and lifestyle predictors, in contrast to our socially grounded variables such as parity and spousal education (85). Similarly, to predict hypertension, Sanaudi et al. applied logistic regression and other traditional models to a Malaysian

Formatted: Font: 18 pt

~~cohort of 11,520 adults and reported 76.73% accuracy with clinical predictors like diabetes and hypercholesterolemia. In contrast, our more advanced modeling approach achieved higher accuracy (91%) and identified socially contextual factors through rigorous feature analysis (86). Seo et al. explored gender-specific hypertension prediction using physiological markers in 2,906 South Koreans, with their best model (Elastic Net) achieving AUCs of 0.865 for men and 0.831 for women. Conversely, our sociodemographic and behavioral model demonstrated superior performance (AUC: 0.95, F1-score: 94%) and revealed culturally relevant predictors such as contraceptive use and household headship, affirming the importance of social determinants in low-resource settings (87). Tanaka et al. examined 15,965 Japanese adults over 6.1 years to predict incident hypertension using models such as XGBoost, random forest, and artificial neural networks, with their top-performing model achieving an AUC of 0.825 based on clinical biomarkers. Yet, our cross-sectional model outperformed theirs, emphasizing the utility of balanced, non-clinical datasets when contextualized effectively (88). Angelaki et al. developed a random forest model using single-lead ECG data from 1,254 adults to predict arterial hypertension. Their model, driven by ECG morphology and BMI, achieved an AUC of 0.831 and accuracy of 75%. While their approach highlights innovation in wearable biosignal-based prediction, it lacked sociodemographic depth and involved a smaller cohort. In contrast, our model — using national survey data — yielded a higher AUC of 0.95 and accuracy of 91%, with SHAP analysis uncovering context-specific predictors such as parity and spousal education (89). Leha et al., using echocardiographic data in a small clinical cohort of 90 patients, attained an AUC of 0.87; our model, however, not only demonstrated superior predictive power (AUC: 0.95, F1-score: 94%) but also broader applicability to systemic hypertension in general populations (90). Further comparison with the HUNT study, which developed 11-year hypertension risk models for 17,852~~

Norwegians using XGBoost and Elastic Net (AUC ≈ 0.78–0.80), highlighted the strength of our model in predicting current hypertension using socially grounded predictors in a low-resource, gender-specific context (41). Lastly, a quantum-enhanced machine learning (QML) study by Donmez and Kutlu reported extremely high performance (F1-score: 98.9%, accuracy: 98.4%) using quantum feature transformation with classical classifiers. In contrast, our work applied rigorously tuned classical models to a clearly defined and nationally representative cohort. The Extra Trees + SMOTE+ENN model not only performed competitively (F1-score: 94%, AUC: 0.95) but also provided interpretable, demographically grounded insights. This comparison emphasizes that while QML holds future promise, classical ML remains highly effective when aligned with population-specific public health needs (91).

Furthermore, Previous research has demonstrated that the Precision-Recall plot is more useful for evaluating binary classifiers than the Receiver Operating Characteristic (ROC) plot(92).
Also perceived was the propensity for accuracy scores to be skewed toward majority classes, which is consistent with the results of earlier research (93, 94). Thus, F1-score was deemed preferable over accuracy for imbalanced data sets, with some researchers advocating against the use of Cohen's kappa in such scenarios (95, 96). Moreover, the efficacy of SMOTE+ENN class balanced techniques, when utilizing with ML algorithms, in elucidating risk factors for chronic diseases including hypertension was underscored by several studies (97-102). Additionally, ensemble methods such as XG Boost, Random Forest, LightGBM (LGB), and stacked models were found to perform optimally when paired with SMOTE+ENN techniques (98-102).

The SHAP (SHapley Additive exPlanations) method was employed in our study to clarify the main factors affecting the determination of hypertension in our proposed model. Our findings corroborate with existing conventional statistical based analyzed literature indicating the

significant association between contraceptive use, women's age, husband's education level, and the number of living children with hypertension risk (103-106) among reproductive aged women although the studies did not classify the association or relationship only for married women. The identification of these factors through the SHAP approach enhances the credibility of our research findings. Islam, M. M., et al. conducted two studies to address hypertension in Bangladesh. The first identified risk factors among adults aged 35 and above using LASSO and SVMRFE for feature selection, achieving a prediction accuracy of 66.98% with Gradient Boosting (68). The second emphasized hypertension's role in cardiovascular diseases, employing a hybrid machine learning model (78.17% accuracy) and individual algorithms like random forest (73.86%) (67). Asadullah, M., et al predicted associated factors among adult patients, with the hybrid model in their study achieving a prediction accuracy of 78.17% and an AUC of 86.34%. In contrast, our study targeted married women in Bangladesh, utilizing machine learning with class-balancing techniques to predict hypertension-associated factors (66). The Extra Trees algorithm outperformed others, achieving an F1 score of 94%, highlighting its efficacy in managing hypertension in specific populations.

This work highlights the efficacy of using class-balancing techniques, namely the SMOTE+ENN strategy, to find hypertensive predictors in married women in Bangladesh through machine learning models employing SHAP method. The findings emphasize the importance of considering alternative evaluation metrics such as F1-score and AUC-PR, the accordance of our results with previous research highlights the reliability of our findings and adds to a deeper understanding of the predictive factors of hypertension in the context of Bangladesh for married women. Also, it highlights the promising potential of ensemble methods in this domain, particularly in the context of imbalanced datasets.

Conclusion

In this study, we developed and rigorously evaluated machine learning (ML) models to predict hypertension among married women in Bangladesh, using nationally representative data from the 2017–18 Bangladesh Demographic and Health Survey (BDHS). We systematically compared 12 ML algorithms in combination with six class-balancing techniques to address the class imbalance present in the dataset. Our findings reveal that the Extra Trees algorithm, when paired with the SMOTE+ENN (Synthetic Minority Over-sampling Technique + Edited Nearest Neighbors) method, achieved the best predictive performance—attaining an F1-score of 94% and an AUC-PR of 0.95. This highlights the effectiveness of ensemble methods and comprehensive resampling strategies in improving prediction for imbalanced health data.

To enhance the interpretability of our model, we employed SHAP (SHapley Additive exPlanations), which enabled us to identify and rank the top 20 predictive features. Socially and demographically significant variables—including age, contraceptive use, number of living children, spousal education, and household headship—emerged as key predictors. These findings underscore the importance of behavioral and demographic indicators in hypertension risk modeling, especially in low-resource contexts where clinical or biometric data may not be readily available.

Our study contributes to the growing body of evidence supporting the application of ML in public health by: (1) demonstrating that accurate, explainable models can be built using non-clinical variables; (2) showing that such models can outperform others trained on clinical or physiological data, particularly in terms of F1-score and AUC-PR; and (3) emphasizing the critical need to use

evaluation metrics tailored for imbalanced datasets, as traditional metrics like accuracy or AUC-ROC may misrepresent model performance in such settings.

While the model's performance is strong, we recognize the importance of external validation. The release of the 2022 BDHS data will provide a valuable opportunity to test the model's generalizability across time and different population subgroups. Future directions also include exploring advanced techniques such as transfer learning and regional model adaptation to further improve scalability and applicability across similar low- and middle-income country settings.

In summary, this study demonstrates the potential of interpretable, ensemble-based ML models — augmented by robust class-balancing and feature attribution techniques—to inform context-sensitive, gender-specific screening strategies. The insights generated from our work can guide more targeted hypertension interventions and policymaking, ultimately contributing to the reduction of the hypertension burden among married women in Bangladesh and comparable populations.

Using BDHS 2017–18 data, we compared 12 ML algorithms and six class balancing methods for predicting hypertension among married women. Extra Trees + SMOTE+ENN was optimal (F1 = 0.94; AUC-PR = 0.95). SHAP surfaced actionable, context-specific predictors women’s age, contraceptive use, parity, spousal education and household headship underscoring the value of social and demographic information when clinical data are scarce. Metrics tailored to imbalance (F1, AUC-PR) were more informative than accuracy or AUC-ROC alone.

These models can support screening and targeting in eRegistries and digital health platforms, enabling gender-sensitive, resource-aware outreach in LMICs. Policymakers can leverage findings for spousal-education initiatives, parity-focused home BP programs and region-specific strategies.

Priorities include external validation with BDHS 2022; prospective studies; integration of clinical/biometric markers; and exploration of transfer learning, cross-country adaptation and multi-omics integration**.** Linking models to real-time eRegistry streams could enable adaptive thresholding and continuous monitoring. Extending analyses to unmarried women and other under-represented groups will refine generalizability.

Strengths and limitations

~~This study offers several notable strengths. To the best of our knowledge, this is the first study in Bangladesh that integrates class-balancing techniques into machine learning (ML) models to address data imbalance and examine the predictive factors of hypertension among married women in Bangladesh. Additionally, this study incorporates the SHAP (SHapley Additive exPlanations) method, which enhances the interpretability of the model by identifying the most influential predictors of hypertension at both the global and individual levels. Model performance was rigorously evaluated through both parametric and non-parametric statistical tests to select the optimal model for the dataset.~~

However, this study has some limitations too. External validation of the models was not feasible due to the unavailability of comparable datasets. Hypertension, a multifaceted medical condition, is influenced by an array of biochemical, genetic, environmental, and cognitive factors; however, we could not consider these factors in our models due to insufficient data.

Strengths: (i) First Bangladesh study (to our knowledge) to combine class-balancing and ML for hypertension prediction specifically among married women; (ii) transparent feature attribution via SHAP; (iii) rigorous evaluation with repeated CV, nested-CV, test-set bootstrapping and comparative statistics across class balancing strategies; (iv) concrete policy pathways (integration with family-planning/postpartum services, spousal engagement, district tailoring).

Limitations: (i) No external validation yet; BDHS may under-sample marginalized groups; findings do not extend to unmarried women; (ii) important biomedical/genetic/environmental determinants were unavailable; (iii) cross-sectional design limits causal inference SHAP explains predictions, not causes; (iv) fairness audits revealed subgroup gaps requiring monitoring and mitigation; (v) training-time class balancing alters class balance relative to the population accordingly, we calibrated thresholds on the untouched test set and reported bootstrap CIs to reduce optimism; (vi) deployment should account for gender-specific barriers in Bangladesh (mobility constraints, caregiving burden, norms around clinic attendance). Another limitation is the lack of external validation at the time of analysis, as BDHS 2022 was not released then. Since the dataset is now available, we plan to validate our deployed system using BDHS 2022, though its limited feature scope prevents full integration into model training.

Acknowledgements

We are grateful for the authorization to use the BDHS dataset from MEASURE DHS and the Bangladesh National Institute of Population Research and Training (NIPORT). Additionally, icddr,b extends its gratitude to the Governments of Bangladesh and Canada for their core and unrestricted support.

Data and code availability

The data ~~used in~~underlying this study are from the 2017–18 Bangladesh Demographic and Health Survey (BDHS~~), which is~~). These data are owned by the DHS Program and cannot be shared by the authors due to third-party restrictions. However, they are publicly available ~~through the DHS Program. While the DHS datasets are not open for direct download, they are accessible~~free of charge to all researchers upon registration and approval~~ – free of charge and~~ through the DHS Program website. Access is typically ~~processed~~granted within 1–2 business days.

~~To access the BDHS 2017–18~~ The dataset ~~used in this study~~can be requested at:

Formatted: Space Before: Auto, After: Auto

Visit the DHS Program data portal: https://dhsprogram.com/data/

Navigate to the dataset page: https://dhsprogram.com/data/dataset/Bangladesh_Standard-DHS_2017.cfm

~~Create an account and request access, citing the intended use.~~

The analytical codes used in this study, developed in Python and R, are publicly available on GitHub at: https://github.com/NobleNovel/Hypertension_Maternal

Funding statement

This study did not receive any funding from external sources. All research activities were carried out independently without financial support from any funding agency or organization. The authors declare that there are no financial relationships or conflicts of interest related to this study.

Conflicts of Interest

None declared

Reference

1. Hay SI, Abajobir AA, Abate KH, Abbafati C, Abbas KM, Abd-Allah F, et al. Global, regional, and national disability-adjusted life-years (DALYs) for 333 diseases and injuries and healthy life expectancy (HALE) for 195 countries and territories, 1990–2016: a systematic analysis for the Global Burden of Disease Study 2016. 2017;390(10100):1260-344.
2. Rahman MA, Parvez M, Halder HR, Yadav UN, Mistry SKJCE, Health G. Prevalence of and factors associated with prehypertension and hypertension among Bangladeshi young adults: An analysis of the Bangladesh Demographic and Health Survey 2017–18. 2021;12:100912.
3. Forouzanfar MH, Liu P, Roth GA, Ng M, Biryukov S, Marczak L, et al. Global burden of hypertension and systolic blood pressure of at least 110 to 115 mm Hg, 1990-2015. 2017;317(2):165-82.
4. Kibria GMA, Swasey K, Choudhury A, Burrowes V, Stafford KA, Uddin SMI, et al. The new 2017 ACC/AHA guideline for classification of hypertension: changes in prevalence of hypertension among adults in Bangladesh. Journal of human hypertension. 2018;32(8-9):608-16.
5. Farrukh F, Abbasi A, Jawed M, Almas A, Jafar T, Virani SS, Samad Z. Hypertension in Women: A South-Asian Perspective. Frontiers in Cardiovascular Medicine. 2022;9.
6. Kearney PM, Whelton M, Reynolds K, Muntner P, Whelton PK, He J. Global burden of hypertension: analysis of worldwide data. The lancet. 2005;365(9455):217-23.
7. Rahimi K, Emdin CA, MacMahon S. The epidemiology of blood pressure and its worldwide management. Circulation research. 2015;116(6):925-36.
8. National Institute of Population Research and Training Dhaka BMaAD, Bangladesh - MEASURE DHS ICF International Calverton, Maryland, U.S.A. . BANGLADESH DEMOGRAPHIC AND HEALTH SURVEY 2011 [Available from: https://dhsprogram.com/pubs/pdf/fr265/fr265.pdf.
9. National Institute of Population Research and Training Medical Education and Family Welfare Division Ministry of Health and Family Welfare Dhaka BTDPIR, Maryland, USA. Bangladesh Demographic and Health Survey 2017-18 October 2020 [Available from: https://dhsprogram.com/pubs/pdf/FR344/FR344.pdf.

10. Razzaque A, Nahar L, Mustafa AHMG, Ahsan KZ, Islam MS, Yunus MJAPJoPH. Sociodemographic differentials of selected noncommunicable diseases risk factors among adults in Matlab, Bangladesh: findings from a WHO STEPS survey. 2011;23(2):183-91.

11. Tareque MI, Koshio A, Tiedt AD, Hasegawa TJPo. Are the rates of hypertension and diabetes higher in people from lower socioeconomic status in Bangladesh? Results from a nationally representative survey. 2015;10(5):e0127954.

12. Khan MN, Oldroyd JC, Chowdhury EK, Hossain MB, Rana J, Renzetti S, Islam RM. Prevalence, awareness, treatment, and control of hypertension in Bangladesh: Findings from National Demographic and Health Survey, 2017-2018. Journal of clinical hypertension (Greenwich, Conn). 2021;23(10):1830-42.

13. Azeez O, Kulkarni A, Kuklina EV, Kim SY, Cox S. Hypertension and Diabetes in Non-Pregnant Women of Reproductive Age in the United States. Preventing chronic disease. 2019;16:E146.

14. Tuoyire DA, Ayetey H. Gender differences in the association between marital status and hypertension in Ghana. Journal of biosocial science. 2019;51(3):313-34.

15. Segawa HK, Uematsu H, Dorji N, Wangdi U, Dorjee C, Yangchen P, et al. Gender with marital status, cultural differences, and vulnerability to hypertension: Findings from the national survey for noncommunicable disease risk factors and mental health using WHO STEPS in Bhutan. Plos one. 2021;16(8):e0256811.

16. Son M, Heo YJ, Hyun H-J, Kwak HJ. Effects of Marital Status and Income on Hypertension: The Korean Genome and Epidemiology Study (KoGES). Journal of Preventive Medicine and Public Health. 2022;55(6):506.

17. Holt-Lunstad J, Birmingham W, Jones BQ. Is there something unique about marriage? The relative impact of marital status, relationship quality, and network social support on ambulatory blood pressure and mental health. Annals of behavioral medicine. 2008;35(2):239-44.

18. Kumar D, Balraj K, Seth S, Vashista S, Ramteke M, Rathore AS. An improved machine learning-based prediction framework for early detection of events in heart failure patients using mHealth. Health and Technology. 2024;14(3):495-512.

19. Lepakshi VA. Chapter 18 - Machine Learning and Deep Learning based AI Tools for Development of Diagnostic Tools. In: Parihar A, Khan R, Kumar A, Kaushik AK, Gohel H, editors. Computational Approaches for Novel Therapeutic and Diagnostic Designing to Mitigate SARS-CoV-2 Infection: Academic Press; 2022. p. 399-420.

20. Mancia G, Cappuccio FP, Burnier M, Coca A, Persu A, Borghi C, et al. Perspectives on improving blood pressure control to reduce the clinical and economic burden of hypertension. J Intern Med. 2023;294(3):251-68.

21. Tsoi K, Yiu K, Lee H, Cheng HM, Wang TD, Tay JC, et al. Applications of artificial intelligence for hypertension management. Journal of clinical hypertension (Greenwich, Conn). 2021;23(3):568-74.

22. Chaikijurajai T, Laffin LJ, Tang WHW. Artificial Intelligence and Hypertension: Recent Advances and Future Outlook. American Journal of Hypertension. 2020;33(11):967-74.

23. Silva GFS, Fagundes TP, Teixeira BC, Chiavegatto Filho ADP. Machine Learning for Hypertension Prediction: a Systematic Review. Current Hypertension Reports. 2022;24(11):523-33.

24. Padmanabhan S, Tran TQB, Dominiczak AF. Artificial Intelligence in Hypertension. Circulation Research. 2021;128(7):1100-18.

25. Hasselström J, Zarrinkoub R, Holmquist C, Hjerpe P, Ljungman C, Qvarnström M, et al. The Swedish Primary Care Cardiovascular Database (SPCCD): 74 751 hypertensive primary care patients. Blood Pressure. 2014;23(2):116-25.

26. Ji W, Zhang Y, Cheng Y, Wang Y, Zhou Y. Development and validation of prediction models for hypertension risks: A cross-sectional study based on 4,287,407 participants. Front Cardiovasc Med. 2022;9:928948.

27. Tayefi M, Esmaeili H, Saberi Karimian M, Amirabadi Zadeh A, Ebrahimi M, Safarian M, et al. The application of a decision tree to establish the parameters associated with hypertension. Comput Methods Programs Biomed. 2017;139:83-91.
28. Islam MM, Alam MJ, Maniruzzaman M, Ahmed N, Ali MS, Rahman MJ, Roy DC. Predicting the risk of hypertension using machine learning algorithms: A cross sectional study in Ethiopia. PLoS One. 2023;18(8):e0289613.
29. Chowdhury MZI, Leung AA, Walker RL, Sikdar KC, O'Beirne M, Quan H, Turin TC. A comparison of machine learning algorithms and traditional regression-based statistical modeling for predicting hypertension incidence in a Canadian population. Scientific Reports. 2023;13(1):13.
30. Kourou K, Exarchos TP, Exarchos KP, Karamouzis MV, Fotiadis DI. Machine learning applications in cancer prognosis and prediction. Comput Struct Biotechnol J. 2015;13:8-17.
31. Selya AS, Anshutz D. Machine learning for the classification of obesity from dietary and physical activity patterns. Advanced Data Analytics in Health. 2018:77-97.
32. Panaretos D, Koloverou E, Dimopoulos AC, Kouli G-M, Vamvakari M, Tzavelas G, et al. A comparison of statistical and machine-learning techniques in evaluating the association between dietary patterns and 10-year cardiometabolic risk (2002–2012): the ATTICA study. British Journal of Nutrition. 2018;120(3):326-34.
33. Premsagar P, Aldous C, Esterhuizen TM, Gomes BJ, Gaskell JW, Tabb DL. Comparing conventional statistical models and machine learning in a small cohort of South African cardiac patients. Informatics in Medicine Unlocked. 2022;34:101103.
34. Hu P, Li Y, Liu Y, Guo G, Gao X, Su Z, et al. Comparison of Conventional Logistic Regression and Machine Learning Methods for Predicting Delayed Cerebral Ischemia After Aneurysmal Subarachnoid Hemorrhage: A Multicentric Observational Cohort Study. Front Aging Neurosci. 2022;14:857521.
35. Jerez JM, Molina I, García-Laencina PJ, Alba E, Ribelles N, Martín M, Franco L. Missing data imputation using statistical and machine learning methods in a real breast cancer problem. Artificial Intelligence in Medicine. 2010;50(2):105-15.
36. Du Z, Yang Y, Zheng J, Li Q, Lin D, Li Y, et al. Accurate Prediction of Coronary Heart Disease for Patients With Hypertension From Electronic Health Records With Big Data and Machine-Learning Methods: Model Development and Performance Evaluation. JMIR Med Inform. 2020;8(7):e17257.
37. Xing Y, Wang J, Zhao Z, Gao a. Combination Data Mining Methods with New Medical Data to Predicting Outcome of Coronary Heart Disease. Convergence Information Technology, International Conference on. 2007;0:868-72.
38. Ture M, Omurlu I, Kurum A, Ozdamar K. Comparing classification techniques for predicting essential hypertension. Expert Systems with Applications. 2005;29:583-8.
39. Wu X, Yuan X, Wang W, Liu K, Qin Y, Sun X, et al. Value of a machine learning approach for predicting clinical outcomes in young patients with hypertension. Hypertension. 2020;75(5):1271-8.
40. Chang W, Liu Y, Xiao Y, Yuan X, Xu X, Zhang S, Zhou S. A machine-learning-based prediction method for hypertension outcomes based on medical data. Diagnostics. 2019;9(4):178.
41. Schjerven FE, Ingeström EML, Steinsland I, Lindseth F. Development of risk models of incident hypertension using machine learning on the HUNT study data. Scientific Reports. 2024;14(1):5609.
42. Aish M, Ghafoor A, Nasim F, Ali K, Akhter S, Azeem S. Improving Stroke Prediction Accuracy through Machine Learning and Synthetic Minority Over-sampling. Journal of Computing & Biomedical Informatics. 2024;07.
43. Assi S, Jayabalan M, Parakh V, Assi J, Al Hamid A, Obe DA-J. Predicting Incidence of Stroke via Supervised Machine Learning Methods on Class Imbalanced Data. Non-Invasive Health Systems based on Advanced Biomedical Signal and Image Processing: CRC Press; 2024. p. 128-44.

44. Ijaz MF, Alfian G, Syafrudin M, Rhee J. Hybrid Prediction Model for Type 2 Diabetes and Hypertension Using DBSCAN-Based Outlier Detection, Synthetic Minority Over Sampling Technique (SMOTE), and Random Forest. Applied Sciences. 2018;8(8):1325.

45. Alsmariy R, Healy G, Abdelhafez H. Predicting cervical cancer using machine learning methods. International Journal of Advanced Computer Science and Applications. 2020;11(7).

46. Sihag G, Yadav P, Vijay V, Delcroix V, Siebert X, Yadav SK, Puisieux F, editors. Advantages of oversampling techniques: a case study in risk factors for fall prediction. International Conference on Information and Communication Technologies for Ageing Well and e-Health; 2021: Springer.

47. Muraru MM, Simó Z, Iantovics LB. Cervical Cancer Prediction based on Imbalanced Data using Machine Learning Algorithms. Preprints: Preprints; 2024.

48. Zheng H, Sherazi SWA, Lee JY. A cost-sensitive deep neural network-based prediction model for the mortality in acute myocardial infarction patients with hypertension on imbalanced data. Frontiers in Cardiovascular Medicine. 2024;11.

49. Nguyen HM, Cooper EW, Kamei K. Borderline over-sampling for imbalanced data classification. Int J Knowl Eng Soft Data Paradigms. 2009;3:4-21.

50. Qasim HM, Ata O, Ansari MA, Alomary MN, Alghamdi S, Almehmadi M. Hybrid Feature Selection Framework for the Parkinson Imbalanced Dataset Prediction Problem. Medicina (Kaunas). 2021;57(11).

51. Chai SS, Goh KL, Cheah WL, Chang YHR, Ng GW. Hypertension Prediction in Adolescents Using Anthropometric Measurements: Do Machine Learning Models Perform Equally Well? Applied Sciences. 2022;12(3):1600.

52. Shuja M, Mittal S, Zaman M. Effective Prediction of Type II Diabetes Mellitus Using Data Mining Classifiers and SMOTE. 2020. p. 195-211.

53. Wang K, Tian J, Zheng C, Yang H, Ren J, Li C, et al. Improving risk identification of adverse outcomes in chronic heart failure using SMOTE+ ENN and machine learning. Risk management and healthcare policy. 2021:2453-63.

54. Matondang N, Surantha N. Effects of oversampling SMOTE in the classification of hypertensive dataset. Adv Sci Technol Eng Syst. 2020;5(4).

55. Huang X, Cao T, Chen L, Wu H, Li J, Tan Z, et al. Predicting Stroke Risk in a Chinese Hypertensive Population Using Machine Learning. 2021.

56. Alabdallah A, Pashami S, Rögnvaldsson T, Ohlsson M, editors. SurvSHAP: a proxy-based algorithm for explaining survival models with SHAP. 2022 IEEE 9th international conference on data science and advanced analytics (DSAA); 2022: IEEE.

57. Cakiroglu C, Demir S, Hakan Ozdemir M, Latif Aylak B, Sariisik G, Abualigah L. Data-driven interpretable ensemble learning methods for the prediction of wind turbine power incorporating SHAP analysis. Expert Systems with Applications. 2024;237:121464.

58. Luo H, Xiang C, Zeng L, Li S, Mei X, Xiong L, et al. SHAP based predictive modeling for 1 year all-cause readmission risk in elderly heart failure patients: feature selection and model interpretation. Scientific Reports. 2024;14(1):17728.

59. Xiaomao X, Xudong Z, Yuanfang W. A Comparison of Feature Selection Methodology for Solving Classification Problems in Finance. Journal of Physics: Conference Series. 2019;1284(1):012026.

60. Raufi B, Longo L. Comparing ANOVA and PowerShap Feature Selection Methods via Shapley Additive Explanations of Models of Mental Workload Built with the Theta and Alpha EEG Band Ratios. BioMedInformatics. 2024;4(1):853-76.

61. Ejiyi CJ, Qin Z, Ukwuoma CC, Nneji GU, Monday HN, Ejiyi MB, et al. Comparative performance analysis of Boruta, SHAP, and Borutashap for disease diagnosis: A study with multiple machine learning algorithms. Network: Computation in Neural Systems.1-38.

62. Asadi R, Khattak A, Vashani H, Almujibah HR, Rabie H, Asadi S, Dimitrijevic B. Self-Paced Ensemble-SHAP Approach for the Classification and Interpretation of Crash Severity in Work Zone Areas. Sustainability. 2023;15(11):9076.
63. Tran VQ, Byeon H. Predicting dementia in Parkinson's disease on a small tabular dataset using hybrid LightGBM–TabPFN and SHAP. DIGITAL HEALTH. 2024;10:20552076241272585.
64. Amin P. Feature Importance in Predicting Clinical Outcome: Statistics vs. Explainable Artificial Intelligence. bioRxiv. 2024:2024.07. 21.604467.
65. Lee Y, Kim K, Seo J. CLE-SH: Comprehensive Literal Explanation package for SHapley values by statistical validity. arXiv preprint arXiv:240912578. 2024.
66. Asadullah M, Hossain MM, Rahaman S, Amin MS, Sumy MSA, Parh MYA, Hossain MA. Evaluation of machine learning techniques for hypertension risk prediction based on medical data in Bangladesh. Indonesian Journal of Electrical Engineering and Computer Science. 2023;31(3):1794-802.
67. Islam M, Alam J, Kumar S, Islam A, Khan MR, Rabby S, et al. Development and validation of a nomogram model for predicting the risk of hypertension in Bangladesh. Heliyon. 2024;10(22).
68. Islam MM, Rahman MJ, Chandra Roy D, Tawabunnahar M, Jahan R, Ahmed N, Maniruzzaman M. Machine learning algorithm for characterizing risks of hypertension, at an early stage in Bangladesh. Diabetes Metab Syndr. 2021;15(3):877-84.
69. Islam SMS, Talukder A, Awal MA, Siddiqui MMU, Ahamad MM, Ahammed B, et al. Machine Learning Approaches for Predicting Hypertension and Its Associated Factors Using Population-Level Data From Three South Asian Countries. Front Cardiovasc Med. 2022;9:839379.
70. Parvin S, Akter S, Hossain MI, Ali MS, Soni MSM. Residential variations in hypertension prevalence and trends among adults in Bangladesh. Research in Health Services & Regions. 2024;3(1):3.
71. Siddiquee T. Machine Learning Approaches for Estimating Prevalence of Undiagnosed Hypertension among Bangladeshi Adults: Evidence from a Nationwide Survey.
72. Al Kibria GM, Burrowes V, Choudhury A, Sharmeen A, Swasey KJIJoCH. Sex differences in prevalence and associated factors of prehypertension and hypertension among Bangladeshi adults. 2019;1:100006.
73. Chowdhury MAB, Islam M, Rahman J, Uddin MT, Haque MR, Uddin MJJPo. Changes in prevalence and risk factors of hypertension among adults in Bangladesh: An analysis of two waves of nationally representative surveys. 2021;16(12):e0259507.
74. Hossain M, Khan M, Ababneh F, Shaw JJBph. Identifying factors influencing contraceptive use in Bangladesh: evidence from BDHS 2014 data. 2018;18(1):1-14.
75. Khan MN, Islam MM, Islam RM. Association Between Contraception Use, Diabetes and Hypertension: Findings from Bangladesh Demographic and Health Survey. 2022.
76. Hossain MB, Khan MN, Oldroyd JC, Rana J, Magliago DJ, Chowdhury EK, et al. Prevalence of, and risk factors for, diabetes and prediabetes in Bangladesh: Evidence from the national survey using a multilevel Poisson regression model with a robust variance. 2022;2(6):e0000461.
77. Islam R, Khan M, Oldroyd J, Rana J, Chowdhury E, Karim M, Hossain MJM. Prevalence of diabetes and prediabetes among Bangladeshi adults and associated factors: Evidence from the Demographic and Health Survey, 2017-18. 2021.
78. Organization WH. Definition and diagnosis of diabetes mellitus and intermediate hyperglycaemia: report of a WHO/IDF consultation. 2006.
79. Zong X, Wang H, Yang L, Guo Y, Zhao M, Magnussen CG, Xi B. Maternal Pre-pregnancy Body Mass Index Categories and Infant Birth Outcomes: A Population-Based Study of 9 Million Mother-Infant Pairs. Front Nutr. 2022;9:789833.
80. Aksoy S, Haralick RM. Feature normalization and likelihood-based similarity measures for image retrieval. Pattern recognition letters. 2001;22(5):563-82.

81. Byeon HJIJoACS, Applications. Development of a physical impairment prediction model for Korean elderly people using synthetic minority over-sampling technique and XGBoost. 2021;12(1).
82. Byeon HJIJoACS, Applications. Predicting the depression of the South Korean elderly using SMOTE and an imbalanced binary dataset. 2021;12(1).
83. Hanafy M, Ming RJIJoACS, Applications. Improving imbalanced data classification in auto insurance by the data level approaches. 2021;12(6).
84. Bergstra J, Bengio YJJomlr. Random search for hyper-parameter optimization. 2012;13(2).
85. Sifat IK, Kibria MK. Optimizing hypertension prediction using ensemble learning approaches. PLoS One. 2024;19(12):e0315865.
86. Sanaudi R, Zakaria ZA, Khairulisam AA, Ibrahim N, Ul Saufie AZ. Unveil the Features Influencing Hypertension Adults in Malaysia using Machine Learning Models. Malaysian Journal of Medicine & Health Sciences. 2024;20(6).
87. Seo J-W, Lee S, Yim MH. Machine Learning Approach for Predicting Hypertension Based on Body Composition in South Korean Adults. Bioengineering. 2024;11(9):921.
88. Tanaka M, Akiyama Y, Mori K, Hosaka I, Endo K, Ogawa T, et al. Machine learning-based analyses of contributing factors for the development of hypertension: a comparative study. Clinical and Experimental Hypertension. 2025;47(1):2449613.
89. Angelaki E, Barmparis GD, Fragkiadakis K, Maragkoudakis S, Zacharis E, Plevritaki A, et al. Diagnostic performance of single-lead electrocardiograms for arterial hypertension diagnosis: a machine learning approach. Journal of human hypertension. 2025;39(1):58-65.
90. Leha A, Hellenkamp K, Unsöld B, Mushemi-Blake S, Shah AM, Hasenfuß G, Seidler T. A machine learning approach for the prediction of pulmonary hypertension. PloS one. 2019;14(10):e0224453.
91. Donmez TB, Kutlu M. Explainable quantum-enhanced machine learning for hypertension prediction. The European Physical Journal Special Topics. 2025:1-13.
92. Saito T, Rehmsmeier MJPo. The precision-recall plot is more informative than the ROC plot when evaluating binary classifiers on imbalanced datasets. 2015;10(3):e0118432.
93. Akosa JS, editor Predictive Accuracy : A Misleading Performance Measure for Highly Imbalanced Data2017.
94. Alam TM, Shaukat K, Hameed IA, Luo S, Sarwar MU, Shabbir S, et al. An investigation of credit card default prediction in the imbalanced datasets. 2020;8:201173-98.
95. Delgado R, Tibau X-AJPo. Why Cohen's Kappa should be avoided as performance measure in classification. 2019;14(9):e0222916.
96. Jeni LA, Cohn JF, De La Torre F, editors. Facing imbalanced data--recommendations for the use of performance metrics. 2013 Humaine association conference on affective computing and intelligent interaction; 2013: IEEE.
97. Wang X, Ren J, Ren H, Song W, Qiao Y, Zhao Y, et al. Diabetes mellitus early warning and factor analysis using ensemble Bayesian networks with SMOTE-ENN and Boruta. Scientific Reports. 2023;13(1):12718.
98. Muntasir Nishat M, Faisal F, Jahan Ratul I, Al-Monsur A, Ar-Rafi AM, Nasrullah SM, et al. A Comprehensive Investigation of the Performances of Different Machine Learning Classifiers with SMOTE-ENN Oversampling Technique and Hyperparameter Optimization for Imbalanced Heart Failure Dataset. Scientific Programming. 2022;2022:3649406.
99. Fitriyani NL, Syafrudin M, Alfian G, Yang Ck, Rhee J, Ulyah SM, editors. Chronic Disease Prediction Model Using Integration of DBSCAN, SMOTE-ENN, and Random Forest. 2022 ASU International Conference in Emerging Technologies for Sustainability and Intelligent Systems (ICETSIS); 2022 22-23 June 2022.

100. Parthasarathy S, Jayaraman V, R JPP, editors. Predicting Heart Failure using SMOTE-ENN-XGBoost. 2023 International Conference on Intelligent Data Communication Technologies and Internet of Things (IDCIoT); 2023 5-7 Jan. 2023.

101. Ullah Z, Saleem F, Jamjoom M, Fakieh B, Kateb F, Ali AM, Shah B. Detecting High-Risk Factors and Early Diagnosis of Diabetes Using Machine Learning Methods. Computational Intelligence and Neuroscience. 2022;2022:2557795.

102. Tang J, Wang X, Wan H, Lin C, Shao Z, Chang Y, et al. Joint modeling strategy for using electronic medical records data to build machine learning models: an example of intracerebral hemorrhage. BMC Medical Informatics and Decision Making. 2022;22(1):278.

103. Ahammed B, Maniruzzaman M, Talukder A, Ferdausi F. Prevalence and risk factors of hypertension among young adults in Albania. High Blood Pressure & Cardiovascular Prevention. 2021;28:35-48.

104. Weare AR, Feng Z, McGrath N. The prevalence of hypertension and hypertension control among married Namibian couples. Plos one. 2023;18(8):e0289788.

105. Khan MN, Islam MM, Islam RM. Pattern of contraceptive use among reproductive-aged women with diabetes and/or hypertension: findings from Bangladesh Demographic and Health Survey. BMC Women's Health. 2022;22(1):230.

106. Bista B, Dhungana RR, Chalise B, Pandey AR. Prevalence and determinants of non-communicable diseases risk factors among reproductive aged women of Nepal: Results from Nepal Demographic Health Survey 2016. PloS one. 2020;15(3):e0218840.

1. Hay SI, Abajobir AA, Abate KH, Abbafati C, Abbas KM, Abd-Allah F, et al. Global, regional, and national disability-adjusted life-years (DALYs) for 333 diseases and injuries and healthy life expectancy (HALE) for 195 countries and territories, 1990–2016: a systematic analysis for the Global Burden of Disease Study 2016. 2017;390(10100):1260-344.

2. Rahman MA, Parvez M, Halder HR, Yadav UN, Mistry SKJCE, Health G. Prevalence of and factors associated with prehypertension and hypertension among Bangladeshi young adults: An analysis of the Bangladesh Demographic and Health Survey 2017–18. 2021;12:100912.

3. Forouzanfar MH, Liu P, Roth GA, Ng M, Biryukov S, Marczak L, et al. Global burden of hypertension and systolic blood pressure of at least 110 to 115 mm Hg, 1990-2015. 2017;317(2):165-82.

4. Ghosh PK, Harun MGD, Shanta IS, Islam A, Jannat KKE, Mannan H. Prevalence and determinants of hypertension among older adults: A comparative analysis of the 6th and 8th national health surveys of Bangladesh. Plos one. 2023;18(10):e0292989.

5. Kibria GMA, Swasey K, Choudhury A, Burrowes V, Stafford KA, Uddin SMI, et al. The new 2017 ACC/AHA guideline for classification of hypertension: changes in prevalence of hypertension among adults in Bangladesh. Journal of human hypertension. 2018;32(8-9):608-16.

6. Farrukh F, Abbasi A, Jawed M, Almas A, Jafar T, Virani SS, Samad Z. Hypertension in Women: A South-Asian Perspective. Frontiers in Cardiovascular Medicine. 2022;9.

7. Kearney PM, Whelton M, Reynolds K, Muntner P, Whelton PK, He J. Global burden of hypertension: analysis of worldwide data. The lancet. 2005;365(9455):217-23.

8. Rahimi K, Emdin CA, MacMahon S. The epidemiology of blood pressure and its worldwide management. Circulation research. 2015;116(6):925-36.

9. National Institute of Population Research and Training Dhaka BMaAD, Bangladesh MEASURE DHS ICF International Calverton, Maryland, U.S.A. . BANGLADESH DEMOGRAPHIC AND HEALTH SURVEY 2011 [Available from: https://dhsprogram.com/pubs/pdf/fr265/fr265.pdf.

10. National Institute of Population Research and Training Medical Education and Family Welfare Division Ministry of Health and Family Welfare Dhaka BTDPIR, Maryland, USA. Bangladesh Demographic and Health Survey 2017-18 October 2020 [Available from: https://dhsprogram.com/pubs/pdf/FR344/FR344.pdf.

11. Razzaque A, Nahar L, Mustafa AHMG, Ahsan KZ, Islam MS, Yunus MJAPJoPH. Sociodemographic differentials of selected noncommunicable diseases risk factors among adults in Matlab, Bangladesh: findings from a WHO STEPS survey. 2011;23(2):183-91.

12. Tareque MI, Koshio A, Tiedt AD, Hasegawa TJPo. Are the rates of hypertension and diabetes higher in people from lower socioeconomic status in Bangladesh? Results from a nationally representative survey. 2015;10(5):e0127954.

13. Khan MN, Oldroyd JC, Chowdhury EK, Hossain MB, Rana J, Renzetti S, Islam RM. Prevalence, awareness, treatment, and control of hypertension in Bangladesh: Findings from National Demographic and Health Survey, 2017-2018. Journal of clinical hypertension (Greenwich, Conn). 2021;23(10):1830-42.

14. Azeez O, Kulkarni A, Kuklina EV, Kim SY, Cox S. Hypertension and Diabetes in Non-Pregnant Women of Reproductive Age in the United States. Preventing chronic disease. 2019;16:E146.

15. Tuoyire DA, Ayetey H. Gender differences in the association between marital status and hypertension in Ghana. Journal of biosocial science. 2019;51(3):313-34.

16. Segawa HK, Uematsu H, Dorji N, Wangdi U, Dorjee C, Yangchen P, et al. Gender with marital status, cultural differences, and vulnerability to hypertension: Findings from the national survey for noncommunicable disease risk factors and mental health using WHO STEPS in Bhutan. Plos one. 2021;16(8):e0256811.

17. Son M, Heo YJ, Hyun H-J, Kwak HJ. Effects of Marital Status and Income on Hypertension: The Korean Genome and Epidemiology Study (KoGES). Journal of Preventive Medicine and Public Health. 2022;55(6):506.

18. Holt-Lunstad J, Birmingham W, Jones BQ. Is there something unique about marriage? The relative impact of marital status, relationship quality, and network social support on ambulatory blood pressure and mental health. Annals of behavioral medicine. 2008;35(2):239-44.

19. Hong T, Xie S, Liu X, Wu J, Chen G. Do Machine Learning Approaches Perform Better Than Regression Models in Mapping Studies? A Systematic Review. Value in Health. 2025;28(5):800-11.

20. Saxena A, Sharma S, Kumar Johari P, Pandey A, Kumar S. A fair and interpretable deep learning approach for healthcare access prediction in underserved communities. Discover Artificial Intelligence. 2025;5(1):185.

21. Shen S, Qi W, Li S, Zeng J, Liu X, Zhu X, et al. Mapping the landscape of machine learning in chronic disease management: A comprehensive bibliometric study. Digit Health. 2025;11:20552076251361614.

22. Dhanda SS, Panwar D, Lin C-C, Sharma TK, Rastogi D, Bindewari S, et al. Advancement in public health through machine learning: a narrative review of opportunities and ethical considerations. Journal of Big Data. 2025;12(1):154.

23. El-Geneedy M, El-Din Moustafa H, Khater H, Abd-Elsamee S, Gamel SA. A comprehensive explainable AI approach for enhancing transparency and interpretability in stroke prediction. Scientific Reports. 2025;15(1):26048.

24. Kumar D, Balraj K, Seth S, Vashista S, Ramteke M, Rathore AS. An improved machine learning-based prediction framework for early detection of events in heart failure patients using mHealth. Health and Technology. 2024;14(3):495-512.

25. Lepakshi VA. Chapter 18 - Machine Learning and Deep Learning based AI Tools for Development of Diagnostic Tools. In: Parihar A, Khan R, Kumar A, Kaushik AK, Gohel H, editors. Computational Approaches for Novel Therapeutic and Diagnostic Designing to Mitigate SARS-CoV-2 Infection: Academic Press; 2022. p. 399-420.
26. Mancia G, Cappuccio FP, Burnier M, Coca A, Persu A, Borghi C, et al. Perspectives on improving blood pressure control to reduce the clinical and economic burden of hypertension. J Intern Med. 2023;294(3):251-68.
27. Tsoi K, Yiu K, Lee H, Cheng HM, Wang TD, Tay JC, et al. Applications of artificial intelligence for hypertension management. Journal of clinical hypertension (Greenwich, Conn). 2021;23(3):568-74.
28. Chaikijurajai T, Laffin LJ, Tang WHW. Artificial Intelligence and Hypertension: Recent Advances and Future Outlook. American Journal of Hypertension. 2020;33(11):967-74.
29. Silva GFS, Fagundes TP, Teixeira BC, Chiavegatto Filho ADP. Machine Learning for Hypertension Prediction: a Systematic Review. Current Hypertension Reports. 2022;24(11):523-33.
30. Padmanabhan S, Tran TQB, Dominiczak AF. Artificial Intelligence in Hypertension. Circulation Research. 2021;128(7):1100-18.
31. Hasselström J, Zarrinkoub R, Holmquist C, Hjerpe P, Ljungman C, Qvarnström M, et al. The Swedish Primary Care Cardiovascular Database (SPCCD): 74 751 hypertensive primary care patients. Blood Pressure. 2014;23(2):116-25.
32. Ji W, Zhang Y, Cheng Y, Wang Y, Zhou Y. Development and validation of prediction models for hypertension risks: A cross-sectional study based on 4,287,407 participants. Front Cardiovasc Med. 2022;9:928948.
33. Tayefi M, Esmaeili H, Saberi Karimian M, Amirabadi Zadeh A, Ebrahimi M, Safarian M, et al. The application of a decision tree to establish the parameters associated with hypertension. Comput Methods Programs Biomed. 2017;139:83-91.
34. Islam MM, Alam MJ, Maniruzzaman M, Ahmed N, Ali MS, Rahman MJ, Roy DC. Predicting the risk of hypertension using machine learning algorithms: A cross sectional study in Ethiopia. PLoS One. 2023;18(8):e0289613.
35. Chowdhury MZI, Leung AA, Walker RL, Sikdar KC, O'Beirne M, Quan H, Turin TC. A comparison of machine learning algorithms and traditional regression-based statistical modeling for predicting hypertension incidence in a Canadian population. Scientific Reports. 2023;13(1):13.
36. Aish M, Ghafoor A, Nasim F, Ali K, Akhter S, Azeem S. Improving Stroke Prediction Accuracy through Machine Learning and Synthetic Minority Over-sampling. Journal of Computing & Biomedical Informatics. 2024;07.
37. Schjerven FE, Ingeström EML, Steinsland I, Lindseth F. Development of risk models of incident hypertension using machine learning on the HUNT study data. Scientific Reports. 2024;14(1):5609.
38. Chang W, Liu Y, Xiao Y, Yuan X, Xu X, Zhang S, Zhou S. A machine-learning-based prediction method for hypertension outcomes based on medical data. Diagnostics. 2019;9(4):178.
39. Du Z, Yang Y, Zheng J, Li Q, Lin D, Li Y, et al. Accurate Prediction of Coronary Heart Disease for Patients With Hypertension From Electronic Health Records With Big Data and Machine-Learning Methods: Model Development and Performance Evaluation. JMIR Med Inform. 2020;8(7):e17257.
40. Kourou K, Exarchos TP, Exarchos KP, Karamouzis MV, Fotiadis DI. Machine learning applications in cancer prognosis and prediction. Comput Struct Biotechnol J. 2015;13:8-17.
41. Selya AS, Anshutz D. Machine learning for the classification of obesity from dietary and physical activity patterns. Advanced Data Analytics in Health. 2018:77-97.
42. Panaretos D, Koloverou E, Dimopoulos AC, Kouli G-M, Vamvakari M, Tzavelas G, et al. A comparison of statistical and machine-learning techniques in evaluating the association between dietary patterns and 10-year cardiometabolic risk (2002–2012): the ATTICA study. British Journal of Nutrition. 2018;120(3):326-34.

43. Premsagar P, Aldous C, Esterhuizen TM, Gomes BJ, Gaskell JW, Tabb DL. Comparing conventional statistical models and machine learning in a small cohort of South African cardiac patients. Informatics in Medicine Unlocked. 2022;34:101103.
44. Hu P, Li Y, Liu Y, Guo G, Gao X, Su Z, et al. Comparison of Conventional Logistic Regression and Machine Learning Methods for Predicting Delayed Cerebral Ischemia After Aneurysmal Subarachnoid Hemorrhage: A Multicentric Observational Cohort Study. Front Aging Neurosci. 2022;14:857521.
45. Jerez JM, Molina I, García-Laencina PJ, Alba E, Ribelles N, Martín M, Franco L. Missing data imputation using statistical and machine learning methods in a real breast cancer problem. Artificial Intelligence in Medicine. 2010;50(2):105-15.
46. Xing Y, Wang J, Zhao Z, Gao a. Combination Data Mining Methods with New Medical Data to Predicting Outcome of Coronary Heart Disease. Convergence Information Technology, International Conference on. 2007;0:868-72.
47. Ture M, Omurlu I, Kurum A, Ozdamar K. Comparing classification techniques for predicting essential hypertension. Expert Systems with Applications. 2005;29:583-8.
48. Wu X, Yuan X, Wang W, Liu K, Qin Y, Sun X, et al. Value of a machine learning approach for predicting clinical outcomes in young patients with hypertension. Hypertension. 2020;75(5):1271-8.
49. Assi S, Jayabalan M, Parakh V, Assi J, Al Hamid A, Obe DA-J. Predicting Incidence of Stroke via Supervised Machine Learning Methods on Class Imbalanced Data. Non-Invasive Health Systems based on Advanced Biomedical Signal and Image Processing: CRC Press; 2024. p. 128-44.
50. Ijaz MF, Alfian G, Syafrudin M, Rhee J. Hybrid Prediction Model for Type 2 Diabetes and Hypertension Using DBSCAN-Based Outlier Detection, Synthetic Minority Over Sampling Technique (SMOTE), and Random Forest. Applied Sciences. 2018;8(8):1325.
51. Alsmariy R, Healy G, Abdelhafez H. Predicting cervical cancer using machine learning methods. International Journal of Advanced Computer Science and Applications. 2020;11(7).
52. Sihag G, Yadav P, Vijay V, Delcroix V, Siebert X, Yadav SK, Puisieux F, editors. Advantages of oversampling techniques: a case study in risk factors for fall prediction. International Conference on Information and Communication Technologies for Ageing Well and e-Health; 2021: Springer.
53. Muraru MM, Simó Z, Iantovics LB. Cervical Cancer Prediction based on Imbalanced Data using Machine Learning Algorithms. Preprints: Preprints; 2024.
54. Chai SS, Goh KL, Cheah WL, Chang YHR, Ng GW. Hypertension Prediction in Adolescents Using Anthropometric Measurements: Do Machine Learning Models Perform Equally Well? Applied Sciences. 2022;12(3):1600.
55. Shuja M, Mittal S, Zaman M. Effective Prediction of Type II Diabetes Mellitus Using Data Mining Classifiers and SMOTE. 2020. p. 195-211.
56. Wang K, Tian J, Zheng C, Yang H, Ren J, Li C, et al. Improving risk identification of adverse outcomes in chronic heart failure using SMOTE+ ENN and machine learning. Risk management and healthcare policy. 2021:2453-63.
57. Zheng H, Sherazi SWA, Lee JY. A cost-sensitive deep neural network-based prediction model for the mortality in acute myocardial infarction patients with hypertension on imbalanced data. Frontiers in Cardiovascular Medicine. 2024;11.
58. Matondang N, Surantha N. Effects of oversampling SMOTE in the classification of hypertensive dataset. Adv Sci Technol Eng Syst. 2020;5(4).
59. Huang X, Cao T, Chen L, Wu H, Li J, Tan Z, et al. Predicting Stroke Risk in a Chinese Hypertensive Population Using Machine Learning. 2021.
60. Alabdallah A, Pashami S, Rögnvaldsson T, Ohlsson M, editors. SurvSHAP: a proxy-based algorithm for explaining survival models with SHAP. 2022 IEEE 9th international conference on data science and advanced analytics (DSAA); 2022: IEEE.

61. Cakiroglu C, Demir S, Hakan Ozdemir M, Latif Aylak B, Sariisik G, Abualigah L. Data-driven interpretable ensemble learning methods for the prediction of wind turbine power incorporating SHAP analysis. Expert Systems with Applications. 2024;237:121464.
62. Luo H, Xiang C, Zeng L, Li S, Mei X, Xiong L, et al. SHAP based predictive modeling for 1 year all-cause readmission risk in elderly heart failure patients: feature selection and model interpretation. Scientific Reports. 2024;14(1):17728.
63. Xiaomao X, Xudong Z, Yuanfang W. A Comparison of Feature Selection Methodology for Solving Classification Problems in Finance. Journal of Physics: Conference Series. 2019;1284(1):012026.
64. Raufi B, Longo L. Comparing ANOVA and PowerShap Feature Selection Methods via Shapley Additive Explanations of Models of Mental Workload Built with the Theta and Alpha EEG Band Ratios. BioMedInformatics. 2024;4(1):853-76.
65. Ejiyi CJ, Qin Z, Ukwuoma CC, Nneji GU, Monday HN, Ejiyi MB, et al. Comparative performance analysis of Boruta, SHAP, and Borutashap for disease diagnosis: A study with multiple machine learning algorithms. Network: Computation in Neural Systems.1-38.
66. Asadi R, Khattak A, Vashani H, Almujibah HR, Rabie H, Asadi S, Dimitrijevic B. Self-Paced Ensemble-SHAP Approach for the Classification and Interpretation of Crash Severity in Work Zone Areas. Sustainability. 2023;15(11):9076.
67. Tran VQ, Byeon H. Predicting dementia in Parkinson's disease on a small tabular dataset using hybrid LightGBM–TabPFN and SHAP. DIGITAL HEALTH. 2024;10:20552076241272585.
68. Amin P. Feature Importance in Predicting Clinical Outcome: Statistics vs. Explainable Artificial Intelligence. bioRxiv. 2024:2024.07. 21.604467.
69. Lee Y, Kim K, Seo J. CLE-SH: Comprehensive Literal Explanation package for SHapley values by statistical validity. arXiv preprint arXiv:240912578. 2024.
70. Asadullah M, Hossain MM, Rahaman S, Amin MS, Sumy MSA, Parh MYA, Hossain MA. Evaluation of machine learning techniques for hypertension risk prediction based on medical data in Bangladesh. Indonesian Journal of Electrical Engineering and Computer Science. 2023;31(3):1794-802.
71. Islam M, Alam J, Kumar S, Islam A, Khan MR, Rabby S, et al. Development and validation of a nomogram model for predicting the risk of hypertension in Bangladesh. Heliyon. 2024;10(22).
72. Islam MM, Rahman MJ, Chandra Roy D, Tawabunnahar M, Jahan R, Ahmed N, Maniruzzaman M. Machine learning algorithm for characterizing risks of hypertension, at an early stage in Bangladesh. Diabetes Metab Syndr. 2021;15(3):877-84.
73. Islam SMS, Talukder A, Awal MA, Siddiqui MMU, Ahamad MM, Ahammed B, et al. Machine Learning Approaches for Predicting Hypertension and Its Associated Factors Using Population-Level Data From Three South Asian Countries. Front Cardiovasc Med. 2022;9:839379.
74. Parvin S, Akter S, Hossain MI, Ali MS, Soni MSM. Residential variations in hypertension prevalence and trends among adults in Bangladesh. Research in Health Services & Regions. 2024;3(1):3.
75. Siddiquee T. Machine Learning Approaches for Estimating Prevalence of Undiagnosed Hypertension among Bangladeshi Adults: Evidence from a Nationwide Survey.
76. Ghosh PK, Islam MA, Haque MA, Tariqujjaman M, Das NC, Ali M, et al. Identifying predictors and assessing causal effect on hypertension risk among adults using Double Machine Learning models: Insights from Bangladesh Demographic and Health Survey. PLoS Comput Biol. 2025;21(7):e1013211.
77. Silvey S, Liu J. Sample Size Requirements for Popular Classification Algorithms in Tabular Clinical Data: Empirical Study. J Med Internet Res. 2024;26:e60231.
78. Baeza-Delgado C, Cerdá Alberich L, Carot-Sierra JM, Veiga-Canuto D, Martínez de las Heras B, Raza B, Martí-Bonmatí L. A practical solution to estimate the sample size required for clinical prediction models generated from observational research on data. European Radiology Experimental. 2022;6(1):22.

79. Infante G, Miceli R, Ambrogi F. Sample size and predictive performance of machine learning methods with survival data: A simulation study. Statistics in Medicine. 2023;42(30):5657-75.
80. Al Kibria GM, Burrowes V, Choudhury A, Sharmeen A, Swasey KJIJoCH. Sex differences in prevalence and associated factors of prehypertension and hypertension among Bangladeshi adults. 2019;1:100006.
81. Chowdhury MAB, Islam M, Rahman J, Uddin MT, Haque MR, Uddin MJJPo. Changes in prevalence and risk factors of hypertension among adults in Bangladesh: An analysis of two waves of nationally representative surveys. 2021;16(12):e0259507.
82. Hossain M, Khan M, Ababneh F, Shaw JJBph. Identifying factors influencing contraceptive use in Bangladesh: evidence from BDHS 2014 data. 2018;18(1):1-14.
83. Khan MN, Islam MM, Islam RM. Association Between Contraception Use, Diabetes and Hypertension: Findings from Bangladesh Demographic and Health Survey. 2022.
84. Hossain MB, Khan MN, Oldroyd JC, Rana J, Magliago DJ, Chowdhury EK, et al. Prevalence of, and risk factors for, diabetes and prediabetes in Bangladesh: Evidence from the national survey using a multilevel Poisson regression model with a robust variance. 2022;2(6):e0000461.
85. Islam R, Khan M, Oldroyd J, Rana J, Chowdhury E, Karim M, Hossain MJM. Prevalence of diabetes and prediabetes among Bangladeshi adults and associated factors: Evidence from the Demographic and Health Survey, 2017-18. 2021.
86. Organization WH. Definition and diagnosis of diabetes mellitus and intermediate hyperglycaemia: report of a WHO/IDF consultation. 2006.
87. Zong X, Wang H, Yang L, Guo Y, Zhao M, Magnussen CG, Xi B. Maternal Pre-pregnancy Body Mass Index Categories and Infant Birth Outcomes: A Population-Based Study of 9 Million Mother-Infant Pairs. Front Nutr. 2022;9:789833.
88. Lemoine A, Tounian P. Childhood anemia and iron deficiency in sub-Saharan Africa - risk factors and prevention: A review. Arch Pediatr. 2020;27(8):490-6.
89. Aksoy S, Haralick RM. Feature normalization and likelihood-based similarity measures for image retrieval. Pattern recognition letters. 2001;22(5):563-82.
90. Byeon HJIJoACS, Applications. Development of a physical impairment prediction model for Korean elderly people using synthetic minority over-sampling technique and XGBoost. 2021;12(1).
91. Byeon HJIJoACS, Applications. Predicting the depression of the South Korean elderly using SMOTE and an imbalanced binary dataset. 2021;12(1).
92. Hanafy M, Ming RJIJoACS, Applications. Improving imbalanced data classification in auto insurance by the data level approaches. 2021;12(6).
93. Geurts P, Ernst D, Wehenkel L. Extremely randomized trees. Machine Learning. 2006;63(1):3-42.
94. Biau G, Scornet E. A random forest guided tour. TEST. 2016;25(2):197-227.
95. Touw WG, Bayjanov JR, Overmars L, Backus L, Boekhorst J, Wels M, van Hijum SAFT. Data mining in the Life Sciences with Random Forest: a walk in the park or lost in the jungle? Briefings in Bioinformatics. 2012;14(3):315-26.
96. Bergstra J, Bengio YJJomlr. Random search for hyper-parameter optimization. 2012;13(2).
97. Sifat IK, Kibria MK. Optimizing hypertension prediction using ensemble learning approaches. PLoS One. 2024;19(12):e0315865.
98. Sanaudi R, Zakaria ZA, Khairulisam AA, Ibrahim N, Ul Saufie AZ. Unveil the Features Influencing Hypertension Adults in Malaysia using Machine Learning Models. Malaysian Journal of Medicine & Health Sciences. 2024;20(6).
99. Seo J-W, Lee S, Yim MH. Machine Learning Approach for Predicting Hypertension Based on Body Composition in South Korean Adults. Bioengineering. 2024;11(9):921.

100. Tanaka M, Akiyama Y, Mori K, Hosaka I, Endo K, Ogawa T, et al. Machine learning-based analyses of contributing factors for the development of hypertension: a comparative study. Clinical and Experimental Hypertension. 2025;47(1):2449613.

101. Angelaki E, Barmparis GD, Fragkiadakis K, Maragkoudakis S, Zacharis E, Plevritaki A, et al. Diagnostic performance of single-lead electrocardiograms for arterial hypertension diagnosis: a machine learning approach. Journal of human hypertension. 2025;39(1):58-65.

102. Leha A, Hellenkamp K, Unsöld B, Mushemi-Blake S, Shah AM, Hasenfuß G, Seidler T. A machine learning approach for the prediction of pulmonary hypertension. PloS one. 2019;14(10):e0224453.

103. Donmez TB, Kutlu M. Explainable quantum-enhanced machine learning for hypertension prediction. The European Physical Journal Special Topics. 2025:1-13.

104. Saito T, Rehmsmeier MJPo. The precision-recall plot is more informative than the ROC plot when evaluating binary classifiers on imbalanced datasets. 2015;10(3):e0118432.

105. Akosa JS, editor Predictive Accuracy : A Misleading Performance Measure for Highly Imbalanced Data2017.

106. Alam TM, Shaukat K, Hameed IA, Luo S, Sarwar MU, Shabbir S, et al. An investigation of credit card default prediction in the imbalanced datasets. 2020;8:201173-98.

107. Delgado R, Tibau X-AJPo. Why Cohen's Kappa should be avoided as performance measure in classification. 2019;14(9):e0222916.

108. Jeni LA, Cohn JF, De La Torre F, editors. Facing imbalanced data--recommendations for the use of performance metrics. 2013 Humaine association conference on affective computing and intelligent interaction; 2013: IEEE.

109. Fitriyani NL, Syafrudin M, Alfian G, Yang Ck, Rhee J, Ulyah SM, editors. Chronic Disease Prediction Model Using Integration of DBSCAN, SMOTE-ENN, and Random Forest. 2022 ASU International Conference in Emerging Technologies for Sustainability and Intelligent Systems (ICETSIS); 2022 22-23 June 2022.

110. Muntasir Nishat M, Faisal F, Jahan Ratul I, Al-Monsur A, Ar-Rafi AM, Nasrullah SM, et al. A Comprehensive Investigation of the Performances of Different Machine Learning Classifiers with SMOTE-ENN Oversampling Technique and Hyperparameter Optimization for Imbalanced Heart Failure Dataset. Scientific Programming. 2022;2022:3649406.

111. Parthasarathy S, Jayaraman V, R JPP, editors. Predicting Heart Failure using SMOTE-ENN-XGBoost. 2023 International Conference on Intelligent Data Communication Technologies and Internet of Things (IDCIoT); 2023 5-7 Jan. 2023.

112. Tang J, Wang X, Wan H, Lin C, Shao Z, Chang Y, et al. Joint modeling strategy for using electronic medical records data to build machine learning models: an example of intracerebral hemorrhage. BMC Medical Informatics and Decision Making. 2022;22(1):278.

113. Ullah Z, Saleem F, Jamjoom M, Fakieh B, Kateb F, Ali AM, Shah B. Detecting High-Risk Factors and Early Diagnosis of Diabetes Using Machine Learning Methods. Computational Intelligence and Neuroscience. 2022;2022:2557795.

114. Ahammed B, Maniruzzaman M, Talukder A, Ferdausi F. Prevalence and risk factors of hypertension among young adults in Albania. High Blood Pressure & Cardiovascular Prevention. 2021;28:35-48.

115. Weare AR, Feng Z, McGrath N. The prevalence of hypertension and hypertension control among married Namibian couples. Plos one. 2023;18(8):e0289788.

116. Khan MN, Islam MM, Islam RM. Pattern of contraceptive use among reproductive-aged women with diabetes and/or hypertension: findings from Bangladesh Demographic and Health Survey. BMC Women's Health. 2022;22(1):230.

117. Bista B, Dhungana RR, Chalise B, Pandey AR. Prevalence and determinants of non-communicable diseases risk factors among reproductive aged women of Nepal: Results from Nepal Demographic Health Survey 2016. PloS one. 2020;15(3):e0218840.

118. Morvaridi M, Rayyani E, Jaafari M, Khiabani A, Rahimlou M. The effect of green coffee extract supplementation on cardio metabolic risk factors: a systematic review and meta-analysis of randomized controlled trials. J Diabetes Metab Disord. 2020;19(1):645-60.

119. Rahimlou M, Grau N, Banaie-Jahromi N, Taheri M, Khosravi A, Mavrommatis Y, Mohammadifard N. Association of adherence to the dietary approach to stop hypertension and Mediterranean diets with blood pressure in a non-hypertensive population: Results from Isfahan Salt Study (ISS). Nutr Metab Cardiovasc Dis. 2022;32(1):109-16.

Supporting Information

Formatted: Font: 12 pt

S1 Appendix: List of class ~~balanced~~balancing techniques

S2 Appendix: Evaluation of machine learning algorithms

S3 Appendix: Evaluation of machine learning algorithms

S4 Appendix: Supplementary Tables

S1 Fig.: Learning curve of the ExtraTrees model for predicting hypertension among married women in Bangladesh.

S2 Fig.: Reliability plot of the ExtraTrees model for predicting hypertension among married women in Bangladesh.

Formatted: Line spacing: single

The Editor,

We are grateful for the thoughtful and constructive comments provided on our manuscript. We have carefully addressed each point raised and made the necessary revisions to improve the clarity, rigor, and reproducibility of our work. Below, we provide a detailed, point-by-point response to each of the comments.

Academic Editor comments
Comments: Please include the following items when submitting your revised manuscript:

- A rebuttal letter that responds to each point raised by the academic editor and reviewer(s). You should upload this letter as a separate file labeled 'Response to Reviewers'.
- A marked-up copy of your manuscript that highlights changes made to the original version. You should upload this as a separate file labeled 'Revised Manuscript with Track Changes'.
- An unmarked version of your revised paper without tracked changes. You should upload this as a separate file labeled 'Manuscript'.

"Response: Thank you for the opportunity to revise our manuscript. In accordance with your instructions, we have submitted the following materials:

- **Response to Reviewers – A detailed rebuttal letter that addresses each point raised by the academic editor and reviewers.**
- **Revised Manuscript with Track Changes – A marked-up version of the manuscript highlighting all revisions made in response to the reviewers' comments.**
- **Manuscript – A clean, unmarked version of the revised manuscript without tracked changes."**

Reviewers' comments:

Reviewer #4: Detailed Comments
Comment: Title and Abstract
The title is clear and descriptive, effectively capturing the dual focus on evaluating ML algorithms and identifying hypertension factors. It could perhaps be tightened for conciseness, something like "Machine Learning Approaches to Predict and Rank Hypertension Risk Factors Among Married Women in Bangladesh" – but that's minor.
The abstract is generally well-structured, summarizing the introduction, methods, results, and conclusion. I like how it highlights key metrics (e.g., F1 score jumping from 8% to 94% with SMOTE+ENN) and the top positive/negative factors from SHAP. However, there are a few awkward phrasings that make it feel a bit rough – for instance, "we aimed to develop a machine learning model to identify and rank predictive factors" could be more precise as "we developed..." since it's a completed study. Also, the prevalence stats (76.9% normal BP vs. 23.1% hypertensive) are useful, but tying them to broader implications right away might help.

Grammatically, "infectundity" in the methods section should be "infecundity" (noted in responses to reviewers, but check if it's fixed). Overall, it's informative, but revise for smoother flow and ensure it matches the submission form version exactly, as flagged by the journal.
“Response: Thank you for the helpful suggestions. We have polished the Title and Abstract for concision, consistency and clarity. The title now reads “Predicting Hypertension and Identifying most important Factors among Married Women in Bangladesh using Machine Learning Approach.” (page. 1, lines 1–2). The Abstract is uniformly in past tense and opens with our completed work: “We developed and interpreted machine-learning (ML) models …” (page. 2, lines 24–26). To contextualize performance gains, we contrast baseline vs. balanced results: F1 = 0.08, recall = 0.04 (imbalanced baseline) → F1 = 0.94, recall = 0.95, AUC-PR = 0.95, ROC-AUC = 0.95 (p. 2, lines 34–37). We state the associational scope (“Models quantify associations rather than causation,” page. 2, line 33) and note next steps (external validation on BDHS-2022, page. 3, lines 47–48). We also articulate programmatic implications (e.g., eRegistries, FP/postpartum screening, spousal education/SMS, district-tailored outreach; page. 3, lines 45–47). The typo “infectundity” → “infecundity” is corrected (page. 8, line 174). Finally, we verified that the Abstract matches the submission-form version exactly.

Comment: Introduction
The introduction sets up the problem nicely, starting with global hypertension stats and narrowing to Bangladesh, with a focus on women and married subgroups. I appreciate the literature review on ML applications in health outcomes – it's comprehensive, covering everything from SVMs in CHD prediction to SHAP's advantages over LASSO/ANOVA. References seem up-to-date and relevant, like the emphasis on tree-based models outperforming logistic regression.
That said, it feels a tad lengthy in places, with some repetition (e.g., multiple paragraphs on ML superiority). The knowledge gap – why married women specifically? – is stated, but could be backed with more evidence on cultural/economic factors in Bangladesh. Also, since the current date is 2025, mentioning why 2017-18 data was used over the 2022 BDHS (as addressed in reviewer responses) should be integrated here for transparency. In my opinion, this section is strong but could be streamlined to about 80% of its length to keep the reader engaged.
“Response: Thank you for the constructive suggestions. We have streamlined the “Introduction” to remove redundancy particularly around statements of ML superiority and tightened the narrative (page. 3–7, lines 52–135). First, we document the national burden and sex disparity in Bangladesh (adults ≥35 years; higher prevalence in women) with nationwide references (page. 3, lines 54–61). Second, we frame marriage as a risk context by citing studies showing higher hypertension among married versus unmarried/never-married women (page. 3, lines 62–64). Third, we add Bangladesh-specific cultural and economic context reproductive roles, domestic workloads, and socioeconomic constraints grounded in relevant studies to explain married women’s elevated risk (p. 6, lines 118–124). Finally, for transparency about data vintage, we clarify that BDHS-2022 was released after our analysis; accordingly, we trained on BDHS-2017–18 and pre-specified external validation on BDHS-2022 (page. 6, lines 124–126).”

Comment: Methods
This is one of the stronger sections, with detailed descriptions of data source (BDHS 2017-18,

n=4,253), preprocessing, and analysis. The choice of 12 algorithms (e.g., Extra Trees, XGBoost, SVM) and 6 balancing techniques (SMOTE, ADASYN, etc.) is justified well for handling imbalance. Validation methods (hold-out + repeated stratified k-fold) and hyperparameter tuning via random search are appropriate, and the evaluation metrics (F1, AUC-PR, etc.) suit imbalanced data. SHAP for feature importance is a great addition for interpretability.
However, a few clarifications are needed. How were features selected initially? The manuscript mentions demographic/clinical vars, but no explicit feature engineering or selection (e.g., Boruta or LASSO, as hinted in intro). Sample size justification is weak – 4,253 is decent, but discuss power for ML, especially with imbalance. Ethical considerations are covered, but confirm if any sensitive vars (e.g., religion) were handled with care. Hardware/software details are fine, but the GitHub repo for code is a plus – ensure it's fully reproducible. I'd suggest adding a flowchart for the workflow to make it easier to follow.
Response: Thank you for the detailed suggestions. We have clarified variable handling, sample-size rationale for imbalanced ML, workflow presentation and ethics/reproducibility. Specifically, we now state that all sociodemographic, behavioral, biometric and anthropometric variables with theoretical/empirical relevance were included without automated feature selection (e.g., Boruta/LASSO/RFE), guided by prior BDHS hypertension literature (page. 10, lines 199–203). Furthermore, previously we mentioned that the variables are considered from the existing literature (page 8-9,lines 163-197). We added a sample-size justification tailored to imbalanced classification and our observed prevalence (page. 8, lines 157–160). Derived variables are specified (diabetes thresholds, page. 9, lines 188–194; BMI categories, page. 9, lines 195–197). A study workflow statement and flowchart was added ("An overview of the analytical workflow from data extraction and preprocessing through class balancing, model training, hyperparameter tuning, validation and evaluation, followed by statistical analysis to compare class-balancing techniques and model performance and SHAP-based interpretation is presented in Fig 2. This schematic provides a concise visual summary of the methodological pipeline described in the preceding subsections.
Fig 2. Workflow of machine learning pipeline for hypertension prediction and risk factor ranking of married women in Bangladesh" page. 15, lines 301–308).
Regarding sensitive attributes: religion was included as recorded in the BDHS public-use dataset, which is de-identified and governed by DHS Program ethical protocols and confidentiality safeguards. We handled this variable with care used only as a covariate for population-level modeling, reported in aggregate, and not for individual targeting or decision rules consistent with the IRB/ethical approvals and BDHS data-use conditions."

Comment: Results
The results are presented logically, starting with descriptive stats (Table 1 is comprehensive, showing hypertension stratification by demographics). Prevalence of 23.1% aligns with prior studies, and breakdowns (e.g., higher in Rangpur, urban areas) add context. Model performances are detailed, with Extra Trees + SMOTE+ENN shining (F1=94%, big improvement from baseline). SHAP plots (Figs 11a-c) effectively rank factors – age <35 negative, overweight positive, etc. – and the directionality (blue/red bars) is intuitive.
But some issues: Tables 4-8 on stats tests (Anderson-Darling, Friedman) are dense; consider consolidating or moving non-essential to supp. Figures need better labels – e.g., ensure font sizes are legible and color contrasts accessible (as per reviewer comments). No mention of overfitting

checks beyond CV scores. Also, while top 20 factors are screened, discuss if multicollinearity (e.g., age and parity) was addressed. Overall, solid but polish the presentation for clarity.
“Response: Thank you for the constructive feedback. We streamlined the Results and strengthened clarity, accessibility and robustness. We consolidated omnibus significance outputs into one main table and moved detailed Anderson–Darling/Friedman statistics to the Supplement (former Tables 4–8 → S4 Appendix → Supplementary Table S2 (S2A–S2E)). Figures were relabeled with larger type, clearer panel markers and self-contained captions; we adopted a color-blind–safe palette, improved contrast to meet accessibility guidelines and verified print-scale legibility via the journal’s PACE checks. To address overfitting, beyond cross-validation we report training–validation deltas, calibration (reliability curves, Brier scores) and learning-curve behavior; preprocessing was nested within folds to prevent leakage. Repeated stratified five-fold CV yielded an average F1 of 0.934 ± 0.012 and nested CV showed an outer-fold mean F1 of 0.965 ± 0.010 (page. 30, 32, lines 425–430 and 454-459), indicating stable generalization. We also added explicit Methods and Results notes on multicollinearity consistent with our analysis. In Methods, we report assessing variance inflation factors (VIF) and pairwise correlations; as expected with one-hot–encoded categorical variables, some VIFs were infinite due to perfect linear dependence among dummy indicators. Because our primary models are gradient-boosted decision trees, which are robust to correlated inputs, this did not compromise predictive validity. We also inspected Spearman correlations among key socio-demographic and fertility-related predictors (page. 14, lines 280-286). In Results, we document structurally inflated VIFs under one-hot encoding and the anticipated dependencies e.g., respondent age <35 vs. husband age ≥40 ($\rho = -0.82$) and mutually exclusive recent-birth categories ($\rho = -0.89$) as well as correlations across BMI categories, parity, and living-children counts. These reflect structural collinearities inherent to categorical coding; tree-based models (e.g., ExtraTrees) are robust to such correlations and predictive performance was unaffected (Supplementary Table S5; Results, page. 31, lines 439–445).”

Comment: Discussion
The discussion ties results back to literature effectively, comparing to studies like Asadullah et al. (hybrid model at 78% accuracy vs. yours at 91%) and others using XGBoost. It highlights strengths: better performance in imbalanced data, context-specific factors for married women. Limitations are acknowledged (no external validation, old data), with plans for BDHS 2022 – good, but expand on how this affects generalizability (e.g., post-COVID changes in health behaviors?).
I think it could delve deeper into implications – e.g., how factors like spousal education inform policy (education campaigns for men?). The SHAP insights are under-discussed; why might secondary education for husbands increase risk? (Perhaps socioeconomic stress?) Avoid overclaiming – e.g., "outperformed other models" is fine, but quantify vs. benchmarks. End with future directions, like integrating real-time data or multi-omics.
Add these recent studies as references to your bibliography: DOI: 10.1007/s40200-020-00536-x, DOI: 10.1016/j.numecd.2021.09.029
“Response: Thank you for these suggestions. We have strengthened the Discussion to (i) quantify gains versus benchmarks, (ii) deepen SHAP interpretation, (iii) spell out policy uses and (iv) clarify generalizability and future work. We now quantify performance against prior work (“AUC-ROC of 0.95 exceeds large clinical models by +0.08–0.17;

accuracy is +13 points vs. Asadullah et al., 91% vs. 78%"; see page. 33, lines 482–485). We justify emphasizing PR over ROC in imbalanced settings ("F1 and AUC-PR provide a truer picture when positives are rare"; see page. 34, lines 486–488). We interpret SHAP signals, noting the unexpected positive association with husbands' secondary education and plausible mechanisms (socioeconomic stressors; wealth/occupation; fertility) and flag this for follow-up (see page. 34, lines 499–501). We translate findings into actions, integrating BP checks into FP/postpartum workflows, engaging husbands via brief education/SMS where partner age/education indicate risk and prioritizing home BP monitoring in high-parity households; district gradients motivate tailored outreach (see page. 34–35, lines 507–510). For generalizability, we note pre-specified external validation on BDHS-2022 to test transportability in a later, post-COVID cohort (discrimination, calibration, threshold transfer; see page. 35, lines 523–526). For robustness, we report training–validation deltas, calibration (reliability and Brier) and nested cross-validation with leakage controls (see page. 35, lines 521–523). We also added the requested citations on diet strategies—green coffee (DOI: 10.1007/s40200-020-00536-x) and DASH/Mediterranean patterns (DOI: 10.1016/j.numecd.2021.09.029; see page. 35, lines 510–513)." Furthermore we added consecutive future pat (see page 36, lines 534-541)

Comment: Conclusion
The conclusion recaps key findings concisely: Extra Trees + SMOTE+ENN best, lists top factors. It emphasizes ensemble methods' potential for imbalanced data, which is spot-on. However, it's a bit short and repetitive of the abstract. Expand to discuss broader impacts (e.g., public health applications in LMICs) and reiterate limitations briefly. As per reviewer #2, make it more explanatory – e.g., how this model could aid screening programs.

"Response: We now close with concrete operational pathways and a clear validation prerequisite: "The best-performing (Extra Trees + SMOTE+ENN) model and SHAP insights can be operationalized through eRegistries, family-planning/postpartum screening, and targeted spousal outreach, pending external validation". To address broader LMIC relevance while keeping the Conclusion explanatory (not duplicative of the Abstract), we add that implementation will require threshold calibration to local prevalence, workload/resource planning, and fairness monitoring. We also reiterate key limitations associational design, focus on married women, and the need for external validation on BDHS-2022 so readers understand scope and generalizability (Conclusion, page. 36–37, lines 528–558)."

General Comments
Comment: Figures and Tables: Mostly clear, but ensure PACE compliance for figures (as noted). Table 1 is excellent; others could use consistent formatting (e.g., avoid repeating stats in Table 5).
Response: We harmonized figure/table references and verified font sizes and contrast per PACE and the Results text now explicitly directs readers to the consolidated performance and diagnostic blocks ("Figures were adjusted for font size and contrast as per PACE; table/figure references are harmonized across Results"; page. 25, lines 367–378; page. 20, lines 352–363; page. 27–28, lines 388–403; page.30,32, lines 426-430,455-459). Cross-references now point directly from the narrative to the consolidated performance table and

the confusion-matrix block and accessibility edits are documented in-text so reviewers can connect the readability updates to the exact evidence blocks. To ensure consistent formatting (including avoiding repeated statistics in Table 5), we standardized table styles across the section and moved non-essential tables to the Supplement (page. 19, 28, 30–32; lines 342–350, 388–396, 426–430, 440–445, 446–463). We also relabeled and re-uploaded all figures after making PACE-compliant adjustments.

Comment: Writing and Language: Mostly good, but some typos/awkward sentences (e.g., "we aimed" in abstract should be past tense). Proofread thoroughly – e.g., "infectundity" fixed? Use consistent terms like "class-balancing" throughout.
Response: The Abstract is now fully in the past tense ("We developed and interpreted …"; page. 2, lines 24–26) and we use "class-balancing" consistently across Methods/Results (e.g., page. 2, lines 28–29; page. 3, line 50; page. 6, line 130; page. 10, line 212; page. 13, line 261; page. 15, lines 302–304; page. 19, lines 341, 343, 349; page. 25, line 379; page. 26, lines 384, 387; page. 28, lines 400–403; page. 29, line 450; page. 32, line 465; page. 33, lines 528, 543, 546, 552; page. 45, line 901). Performance reporting in the Results is cited at page. 30, lines 425–430 and page. 32, lines 454–459. The typo "infectundity" has been corrected to "infecundity" (p. 8, line 174).

Comment: References: Comprehensive (over 100), but check for recency – some pre-2020; add if newer ML-hypertension studies exist.
Response: We audited and refreshed recency across the manuscript and references, adding 2021–2025 ML/SHAP and hypertension studies and integrating them into the narrative where they inform model choice, imbalance-aware metrics, and interpretability. Specifically, new ML-hypertension and benchmarking citations (e.g., Value in Health 2025 (ref. 18), Scientific Reports 2024 HUNT model (ref. 36), Clin Exp Hypert 2025 (ref. 99), J Hum Hypertens 2025 wearable ECG (ref. 100), EPJ Special Topics 2025 QML (ref. 102), plus Bangladesh-focused updates (refs. 69–72, 75, 96)) support comparative performance statements in the Discussion (p. 33, lines 482–485; p. 34, lines 487–488, 499–501) and strengthen context. We also inserted recent SHAP/feature-selection and explainability work (refs. 59–68) alongside our interpretation passages, and added contemporary sample-size/power guidance for tabular clinical ML (refs. 76–78) to the Methods justification (p. 8, lines 157–160). The two diet-related DOIs requested by the reviewer are now cited in the policy/implications sentence green coffee meta-analysis (ref. 117; DOI: 10.1007/s40200-020-00536-x) and DASH/Mediterranean (ref. 118; DOI: 10.1016/j.numecd.2021.09.029)—at Discussion, p. 35, lines 510–513. Together, these updates raise the overall currency of the bibliography while preserving numbering consistency and directly linking new evidence to the affected text blocks

Comment: Reproducibility: GitHub code is a strength, but confirm data access instructions are precise.
Response: We revised Data and code availability to provide precise, step-by-step DHS access and reproducibility details consistent with third-party restrictions. The subsection now clarifies that BDHS 2017–18 data are owned by the DHS Program and cannot be redistributed by the authors; access is free upon registration and approval (typically 1–2

business days), with explicit instructions to visit the DHS data portal and request the Bangladesh 2017–18 Standard DHS dataset (page. 37-38, lines 566–572).

Comment: Originality and Significance: Novel in focusing on married women with SHAP; contributes to ML in global health. But update with 2022 data for timeliness.
Response: We foreground originality and timeliness by stating, "To our knowledge, this is the first Bangladesh study focused on married women combining class-balancing with SHAP explanations," and we specify external validation on BDHS-2022 as the next step (page. 6 and 13 lines 129–131 and 265-272). This placement in the Introduction (novelty) is paired with a concise future-work note in the Discussion to address recency and generalizability, explicitly acknowledging post-COVID shifts and outlining the BDHS-2022 transportability test (Discussion, page. 35, lines 523–526)

Comment: While revising your submission, please upload your figure files to the Preflight Analysis and Conversion Engine (PACE) digital diagnostic tool, https://pacev2.apexcovantage.com/. PACE helps ensure that figures meet PLOS requirements. To use PACE, you must first register as a user. Registration is free. Then, login and navigate to the UPLOAD tab, where you will find detailed instructions on how to use the tool. If you encounter any issues or have any questions when using PACE, please email PLOS at figures@plos.org. Please note that Supporting Information files do not need this step.
Response: All figures will be uploaded to PACE before final submission. Internally, we harmonized contrast and font sizing and made table/figure cross-references explicit in the Results narrative .

Response to Editorial Attachment – PLOS ONE ("Plos One") Word Comments

Evaluating machine learning algorithms and identifying factors associated with hypertension among married women in Bangladesh

Abstract
Comment: The abstract's language is not fully polished (e.g., "we aimed" is not capitalised, and some sections are missing necessary punctuation), affecting professionalism.
Response: We rewrote the Abstract in the past tense and tightened punctuation and flow. The opening now reads: "We developed and interpreted machine-learning (ML) models to predict hypertension and rank associated factors among married women …" (page.2, lines 24-25), replacing the earlier "we aimed" phrasing and aligning with a completed study. This edit standardizes capitalization/punctuation, removes prospective language and aligns tone with completed work, addressing professionalism and consistency concerns flagged by the journal (page 2-3,lines 23-48).

Comment: The results use absolute F1 values (from 8 to 94), but do not mention what the baseline scores for other models were, and the abstract's comparative value is limited without context.
Response: We added explicit baseline-to-balanced context in the Abstract—"The Extra Trees classifier with SMOTE+ENN achieved the best discrimination (F1 = 0.94; AUC-PR = 0.95; ROC-AUC = 0.95). Compared with the original imbalanced training, minority-class detection improved substantially …" (page. 2, lines 34–37). Given PLOS ONE's word-limit for abstracts, we report the full cross-model baselines and balanced scores in the Results narrative (page. 25, lines 367–378) and consolidate per-algorithm metrics (baseline vs. post-resampling across 12 ML algorithms and six class-balancing methods) in Table 5 (page. 26, line 384).

Comment: The abstract does not distinguish between statistical associations and causal inferences; by design, machine learning models show correlation, not causation.
Response: We now make this explicit: "Models quantify associations rather than causation." (page.2, line 33).

Comment: The abstract mentions "top 20 factors," but explores only a few, leaving their full list and ranking method unclear.
Response: We clarified the method and pointed to the full list while keeping the Abstract concise. The Abstract now states: "To interpret results, SHAP was used to rank the top 20 predictors and visualize feature effects" (page. 2, lines 32–41) but, due to word limits, does not enumerate all twenty. The complete ranked list and directions of association are provided in the Results with explicit figure call-outs: Fig. 12a (importance order), Fig. 12b (top-20 with positive/negative direction), and Fig. 12c (summary plot of effects), as referenced in text (page. 29–30, lines 406–421)

Comment: The conclusion focuses on the machine learning aspect without summarizing wider public health implications or practical impact, missing an opportunity to discuss policy or clinical relevance.
Response: We added programmatic implications directly in the Abstract: "Findings support programmatic integration of risk scores into eRegistries, routine blood pressure checks in family planning and postpartum visits, husband-focused education/SMS interventions and prioritization of high-parity households in high-risk regions." (page.3, line 44-47). This deployment language was not present previously.

Comment: The abstract omits main limitations (e.g., no external dataset validation, as discussed in the manuscript). Potential data access and generalizability barriers, as highlighted by reviewers, are not addressed up front.
Response: We now flag external validation as a priority in the Abstract: "External validation on BDHS-2022 is planned to assess generalizability." (page.3, line47-48). This places the limitation up front and points to the immediate next step.

Introduction

Comment: The introduction briefly mentions machine learning but could elaborate more on the advantages, limitations, and previous gaps in traditional statistical models for hypertension risk prediction.

Response: We inserted a concise pros/cons paragraph that recognizes both ML's strengths and limits for public health adoption: "… ML methods can process high-dimensional data … capture nonlinear relationships … At the same time, ML faces limitations … Addressing interpretability and ensuring fairness remain essential …" (page.4, lines 67-73). This balanced framing replaces earlier diffuse statements and anchors the ensuing methods.

Comment: There is an opportunity to better synthesise existing regional studies and clearly indicate how this research advances previous efforts, addressing what is new in methodology or population.

Response: We strengthened the Introduction to synthesize regional ML–hypertension evidence and to make our advance explicit in both population and method. We summarize prior South Asian studies and their recurrent limitations general adult samples, clinical-marker emphasis and scarce subgroup validation then motivate our Bangladesh-specific focus on married women using sociocultural and economic determinants (page. 6, lines 118–123). We state what is new: to our knowledge, this is the first Bangladesh study to evaluate an extensive suite of algorithms with class-balancing and SHAP interpretability specifically for married women (page. 7, lines 129–131), trained on BDHS-2017–18 with pre-specified external validation on BDHS-2022 to ensure transparent assessment of generalizability (page. 6, lines 124–126). We also tie this novelty to programmatic use-cases targeted screening within FP/postpartum services and eRegistries—so the contribution over prior regional work is clear and actionable (page. 7, lines 131–135).

Comment: At times, the introduction contains long sentences that could be better segmented for clarity and flow.

Response: We tightened and segmented the whole Introduction section to improve clarity and flow. Specifically, we broke up long, multi-clause sentences, trimmed repeated "ML superiority" statements and shifted to active voice. The lead-in now appears as two compact, signposted paragraphs: one that concisely summarizes ML capabilities and limitations (page. 4, lines 65-73) and a second that foregrounds the Bangladesh context, the married-women rationale and the study aim (page. 6-7, lines 118-135). Subsequent paragraphs were similarly shortened with clearer transitions (e.g., separating cardiovascular ML use cases, imbalance/metrics, SHAP interpretability and the subgroup gap/BDHS-2022 plan) so key ideas are introduced in smaller, readable units while surfacing the programmatic target screening within existing service touchpoints earlier in the narrative.

Comment: Explicitly state whether this study is the first in Bangladesh to use such an extensive set of algorithms and class balancing approaches for hypertension prediction in married women, highlighting its unique contributions.

Response: **To our knowledge, this is the first Bangladesh study to evaluate an extensive suite of algorithms with class-balancing and SHAP interpretability specifically for married women (page. 7, lines 129–131**

Comment: Address limitations or challenges posed by the dataset (e.g., self-reported data, cross-sectional nature) up front to set proper expectations.
Response: We moved dataset-related caveats to the front of the paper to set expectations. The Introduction now notes that BDHS-2022 was unavailable at analysis and that models were trained on BDHS-2017–18 with pre-specified external validation on BDHS-2022 (page. 6, lines 124-126). We added that the BDHS's cross-sectional design precludes causal inference, results are associational, not causal (page. 35,37 lines 514, 550). We also flag reliance on self-reported variables (e.g., contraceptive use, recent births) with potential recall/measurement error and the limited biomarker scope (primarily BMI and capillary glucose) (page. 10, lines 199-200). Generalizability is explicitly bounded to married women sampled by BDHS (page. 7-8, lines 143–156). The Intro–Methods bridge reiterates the external-validation plan and cites DHS de-identification/ethical safeguards (page. 6,12 lines 124–126,265-272).

Comment: Consider including a brief mention of ethical considerations when using big data or advanced analytics in public health research.
Response: We added an explicit fairness/ethics sentence: "We additionally highlight that fairness and bias are critical in ML health research. Sensitive variables were treated with caution and SHAP interpretability was used to ensure transparency in feature attribution. These safeguards support responsible use of advanced ML in public health policy." (page.15, lines 297-300).

Comment: The transition into the objectives section could be improved for more seamless narrative connection between context and study aims.
Response: We added a bridging objective sentence that ties context to aims: "Therefore, the objective of this study is to develop and validate interpretable machine-learning models for predicting hypertension among married women in Bangladesh, integrating sociodemographic, behavioral, and biometric factors (such as BMI and diabetes status), to identify the most influential predictors and provide evidence to guide targeted screening and public health interventions in low-resource settings
" (page.6-7, lines 131-135)

Comment: Some terminology (e.g., differentiating predictive versus explanatory modeling) can be clarified to aid a broad audience.
Response: We clarify this distinction by repeatedly emphasizing association≠causation and SHAP's role as an explanatory (interpretability) tool rather than a causal estimator (e.g., "Models quantify associations rather than causation," page.2, line 33; and "SHAP values explain model associations, not causal effects," page.35, line 514;).

Methodology
Comment: The lack of validation using independent/external data is a key limitation. While acknowledged, this impacts model generalizability beyond the sampled population. Future work with the now-available BDHS 2022 should be prioritised.

Response: We brought external validation forward and repeated it in Methods and Discussion: "At the time of model development, BDHS-2022 data was not publicly released; consequently, model training and internal validation used BDHS-2017-18. BDHS-2022 is now available and differs modestly in feature scope; to avoid design leakage, we will treat BDHS-2022 strictly as an external test set. Given the scarcity of comparable datasets of the same type, we also reserve BDHS-2022 for validating the deployed system. No retraining or feature re-specification will be performed for the external test; instead, we will apply identical preprocessing and the pre-specified decision threshold" (page.13, lines 265–271) and "as BDHS 2022 was not released then. Since the dataset is now available, we plan to validate our deployed system using BDHS 2022, though its limited feature scope prevents full integration into model training…" (page.37, line 556-558). This sequence was not explicitly laid out previously.

Comment: While a wide set of features is included, the methodology could elaborate whether feature selection (beyond literature-based inclusion) was performed before modeling, possibly using wrapper/filter/embedded methods.
Response: We now explicitly state no automated pre-model selector was used: "All sociodemographic, behavioral, biometric and anthropometric variables with theoretical or empirical relevance to hypertension were extracted from the BDHS 2017–18 dataset. No additional feature engineering or automated feature selection algorithms (e.g., Boruta, LASSO or recursive feature elimination) were applied. Instead, variable inclusion was guided by existing epidemiological literature and prior BDHS-based hypertension studies…" (page.10, lines 199-203). SHAP is identified as the post-hoc interpretability framework.

Comment: The methodology briefly mentions deletion but does not elaborate on the extent or potential bias induced by this approach versus imputation.

Response: We now make missing-data handling explicit and quantified in Methods → Data preparation → "Missing data" (page. 9, lines 206–207): we conducted a complete-case analysis (final n=4,253 married women; 4,546 initially eligible), excluding pregnant respondents and records with missing values; no imputation was performed to avoid model-dependent artifacts in a mixed categorical feature set (page. 7–8, lines 143–156). To show downstream effects, Methods and Results report the hypertension class distribution used for modeling (overall prevalence ≈23.1%), the training/test splits (page. 12, lines 242–243) and the class distributions before and after each resampling method, directing readers to the "Class distribution before and after class balancing" block and sampler-specific counts (page. 19, lines 342–350 and Supplement). These additions clarify what was retained versus excluded, why imputation was not used and how balancing altered minority-class representation, enabling assessment of any bias introduced by deletion versus imputation.

Comment: Training was performed on basic hardware (i5 CPU, 8GB RAM)—potentially limiting more computationally intensive models or hyperparameter searches.
Response: We state compute constraints plainly: "Training was carried out on a workstation with Intel Core i5-8365U … 8 GB RAM … resource limitations constrained

deeper architectures; the chosen algorithms were successfully optimized …" (page. 13-14, lines 275-278).

Comment: No mention of use of survey/sample weights, which are critical for BDHS data to ensure national representativeness. Analytical weighting should be addressed or justified if omitted.

Response: We created a Survey weights subsection and justified the modeling choice: heading "Survey weights" and "The BDHS 2017–18 employs a complex survey design with stratification, clustering and sampling weights to ensure national representativeness. In the present study, we did not apply survey/sample weights because the primary aim was methodological focused on evaluating and comparing the predictive performance of class balancing approaches integrate with machine learning algorithms and ranked risk and protected features rather than estimating population-level prevalence or nationally representative parameters. This approach is consistent with prior ML studies using DHS data in similar contexts (page. 10, line 209-215).

Comment: While IRB considerations are reported, the rationale and implications of using advanced ML (including fairness or bias issues) could be further discussed under ethical considerations.

Response: We added ethics/bias language to complement IRB statements: "As this study involves secondary data analysis, we obtained permission to access the de-identified data from the DHS Program. Since the data was de-identified and publicly available, no additional ethical approval was required for this analysis. The study adhered to the relevant guidelines and regulations for secondary data use.

We additionally highlight that fairness and bias are critical in ML health research. Sensitive variables were treated with caution and SHAP interpretability was used to ensure transparency in feature attribution. These safeguards support responsible use of advanced ML in public health policy. (page.14-15, line 293-300).

Comment: Rationale for preferring Extra Trees, as opposed to XGBoost/Random Forest (other top performers in similar studies), could be more clearly articulated within the methods section, not just in the discussion.

Response: We now state the methodological rationale inside Methods → Machine learning algorithms (page. 12, lines 237–239): Extra Trees was included because it is computationally efficient under extensive resampling, less sensitive to depth/learning-rate tuning than boosted trees, reduces variance via fully randomized split thresholds, and yields stable feature-importance profiles complementing RF's bootstrap splits and XGBoost's gradient-based boosting (92–94). We also justify the choice empirically in Results → "Classification efficacy and confusion matrix" (page. 25, lines 377–378), where paired confusion matrices and bootstrap 95% CIs in the consolidated performance table show Extra Trees + SMOTE+ENN delivering the top F1/AUC-PR while maintaining comparable ROC-AUC versus RF/XGBoost.

Comment: Clearly define composite or derived variables (e.g., explain calculation for BMI and diabetes status) in a dedicated subsection for reproducibility.

Response: We created a dedicated "Derived variables" subsection in Methods and defined each composite variable precisely for reproducibility: Diabetes status is based on fasting plasma glucose measured with the HemoCue Glucose 201 DM (plasma conversion), with values auto-converted to FPG equivalents; diabetes is coded if FPG ≥ 7.0 mmol/L or if antihyperglycemic medication use is self-reported (page. 9, lines 188–194). Body mass index (BMI) is calculated as weight/height² (kg/m²) and categorized using WHO cut-offs: underweight < 18.5, normal 18.5–24.9, overweight 25.0–29.9, and obese ≥ 30 (page. 9, lines 195–197).

Results

Comment: The results could benefit from consolidation, such as combining tables and figures for easier cross-comparison between different algorithms and balancing techniques.

Response: We reorganized the Results to enable side-by-side comparison across algorithms and balancing techniques. A single consolidated performance table (Table 5) now reports all model×sampler metrics, and we place the paired 2×2 confusion matrices for ExtraTrees—original vs. SMOTE+ENN adjacently (Tables 3–4) under the new "Classification efficacy and confusion matrix" subsection see page 25 lines 367-387. We added a compact "Global comparison of class-balancing techniques" block that centralizes the Anderson–Darling normality check (Supplementary S2A), the AUC-PR ANOVA with Tukey HSD (S2B–S2C) and Friedman ranks for non-normal metrics (S2D–S2E) see page 28-29 lines 388-399. To reduce fragmentation, trend visualizations are grouped as boxplots (Figs. 4–11) by metric and the "Class distribution before and after class balancing" subsection reports pre/post sampler counts to contextualize performance shifts see page 19 lines 342-350. Test-set uncertainty for the selected model is summarized once in a bootstrap CI table (Table 6) see page 30 lines 432-435. Furthermore, we reported "Internal overfitting diagnostics" and "Calibration and thresholding" subsection with tables and figure together (see page 30. Lines 426-430 and page. 32, lines 455-459)

Comment: Presenting confidence intervals or statistical uncertainty for key metrics (not only point estimates) would strengthen result reporting and allow readers to assess model reliability.

Response: We agree and now report 95% nonparametric bootstrap CIs for all test-set metrics of the selected ExtraTrees + SMOTE+ENN model (Results, page.31, lines 432-437; Table 6). Specifically, with 1,000 bootstrap replicates on the held-out test set (n=672) we obtain: Precision 0.92 (0.90–0.94), Recall 0.95 (0.93–0.97), F1 0.94 (0.92–0.96), Accuracy 0.91 (0.89–0.94), Specificity 0.83 (0.80–0.87), ROC-AUC 0.95 (0.93–0.97), PR-AUC 0.95 (0.93–0.97), Cohen's κ 0.79 (0.75–0.83), MCC 0.79 (0.75–0.83), and G-mean 0.89 (0.86–0.92). We also clarify the evaluation protocol: class balancing at the individual level, metrics computed at the pre-specified decision threshold (0.55), with percentile CIs). In addition, pairwise AUC-PR comparisons include 95% CIs via Tukey's HSD (Supplementary Table S2C).

Comment: The narrative sections of the results could be more concise and focused, with clearer guidance to the reader on the most clinically or policy-relevant findings.
Response: To improve concision and orient readers to clinically/policy-relevant findings, we colocated interpretability outputs with the consolidated model×sampler table and the paired confusion matrices so the path from discrimination to intervention levers is explicit (page.25, lines 367–387; page.20, lines 352–363). We grouped cross-technique trends and uncertainty into compact summaries a single "Global comparison of class-balancing techniques" block (ANOVA/Tukey for AUC-PR; Friedman ranks for non-normal metrics) and a one-page bootstrap-CI table for the selected model (page.28–29, lines 388–399; page.30, lines 432–435). We also present SHAP rankings alongside factor-direction plots (Figs. 12a–c) to highlight actionable determinants BMI (overweight/obesity, positive), reproductive profile (≥2 living children; limiting/traditional contraception, positive), and spousal education (secondary, positive) and protective strata (age <35, under/normal weight, normoglycemia, negative), with brief, reader-facing text linking these patterns to feasible levers (page.29–30, lines 406–421).

Comment: Visualisation of SHAP values (e.g., summary plots) and confusion matrices should be clearer and referenced in the main results narrative, rather than relegated to supplementary materials.
Response: We made SHAP and confusion matrices prominent in the main text:
"Integrating SHAP with a trained model enabled analysis of global and local predictors. Figure 12a shows the most important features; Fig 12b classifies the top 20 factors by direction of association; Fig 12c presents a SHAP summary plot combining feature importance and effects.
In Fig 12a, respondent age <35 years was the most influential factor, followed by overweight, having two to three ever-born children, and husband/partner with secondary education. Additional contributors included underweight, normal weight, husband/partner <35 years, >2 living children, no births in the last five years and obesity.
Fig 12b indicates negative associations (blue) for age <35, underweight/normal weight, husband/partner <35 years, respondent age 35–40, one child in last five years, normoglycemia, residence in Rajshahi and no formal education for husband/partner. Positive associations (red) included overweight, two to three ever-born children, husband/partner with secondary education, >2 living children, no births in five years, obesity, husband/partner >40, traditional contraceptive use, unmet need for limiting, richest quintile and age difference ≥10 years.
Fig 12c shows these effects at the individual level: blue points (low SHAP values) correspond to features decreasing predicted risk, while red/purple points (high SHAP values) correspond to features increasing predicted risk" (page.29-30, lines 406-421) and the results section headings and table captions for Table 3 and Table 4 (page.25-26, lines 367-382). This moves key visual evidence into the primary narrative.

Comment: Greater emphasis on findings that differ from previous studies, especially regarding novel risk/protective factors found important by the chosen machine learning models.

Response: We agree and now foreground where our results extend or differ from prior reports. In the Abstract and again in Results we added explicit contrasts: "SHAP identified age, parity and recent births, contraceptive pattern, spousal education, and BMI as key predictors; beyond established risks (age ≥40, overweight/obesity), the models highlight program-relevant levers traditional method use and unmet need for limiting, spousal secondary education, larger age-gap (≥10 years) and region (Rajshahi) as independent contributors. Younger age (<35) and normal/underweight status were protective." (Abstract, page.2, line 38-41; Results, page29-30, lines 409–418; Discussion, page.34–35, lines 497–513).

Comment: The discussion of limitations in the results section should be expanded: for example, how the internal validation-only approach may exaggerate performance (potential overfitting).

Response: We expanded the limitations and flagged potential optimism from internal validation. Concretely, we added an Internal overfitting diagnostics block with repeated stratified 5-fold CV, nested CV, and learning-curve checks (Results) and we now report 95% nonparametric bootstrap CIs (1,000 reps) for all test-set metrics of the selected ExtraTrees+SMOTE+ENN model (Results, page.31,lines 432-437; Table 6).. We also document safeguards against leakage/overfitting: class balancing applied inside training folds only; the test set remained untouched; the final model was refit once on the full training set (Sample-flow/class balance, page.32, lines 447–453); metrics were computed at a pre-specified threshold (0.55) with calibration reported (Brier = 0.0626) (Calibration/thresholding; Supplementary Table S3B p.32, lines 455–459);). Subgroup diagnostics reveal heterogeneity (e.g., slightly lower F1 in Rajshahi despite ROC-AUC = 1.0), reinforcing our caution about generalizability (Supplementary Table S6 p.32, lines 461–463). We state explicitly that external, out-of-sample validation and local recalibration are prerequisites for deployment, and that current results while uncertainty-quantified may still be optimistic relative to prospective performance.

Comment: More explicit tracking and reporting of sample sizes for each analysis (post-imputation, balancing) would enhance transparency.

Response: We made sample sizes explicit at every stage and added two audit-style elements. First, a new "Class distribution before and after class balancing" block enumerates training-set counts and proportions by method: original train n=3,581 with 827 hypertensive (23.1%) and 2,754 normotensive (76.9%); SMOTE and ADASYN 50/50; Tomek Links 24.7% minority; ENN 35.0% minority; SMOTE+Tomek 50/50; SMOTE+ENN 63.3% hypertensive (Results, page.19, lines 342–350; Supplementary Table S1). Second, a CONSORT-style sample-flow records N=4,253 , train n=3,581 / test n=672, documents that stratified 5-fold CV preserved the 23.1% prevalence within each fold and notes that balancing was applied inside training folds only (Results, page.32, lines 447–453; Supplementary Tables S4A–S4C). We also print raw counts in the paired confusion matrices (Tables 3–4; Results, page.25–26, lines 367–382) and state the analysis population in the CI table (n=672 in Table 6; Results, page.31, lines 432–437), so denominators are clear wherever metrics are reported.

Discussion

Comment: The discussion could more robustly address how findings might translate to policy or clinical practice, with specific recommendations or scenarios.

Response: To translate findings into policy and practice, we now present concrete, scenario-based pathways alongside deployment guardrails. We describe embedding risk scoring in e-registries, integrating routine blood-pressure checks into family-planning and postpartum visits, engaging husbands via brief education/SMS, prioritizing high-parity households and tailoring outreach by district (e.g., Rajshahi) (Discussion, page.34–35, lines 507–513). Policy use is explicitly linked to the Youden-optimal/pre-specified decision threshold (0.55), with guidance to recalibrate thresholds to local prevalence and the relative costs of false negatives versus false positives (Results, page.32, lines 455–459). We add equity and governance safeguards—ongoing subgroup-level fairness monitoring, transparent model versioning and threshold documentation, and mitigation of potential harms such as stigma or resource diversion and note complementary diet strategies (green coffee; DASH/Mediterranean) to support implementation (Discussion, page.34–35, lines 507–513). The Conclusion reiterates how these pathways can be operationalized in LMIC health systems and underscores external validation as a prerequisite to deployment (Conclusion, page.36–37, lines 528–558).

Comment: Limitations could be expanded beyond technical aspects: for example, discuss the impact of cultural/social determinants, potential biases in BDHS sampling, or gender-specific barriers to care in Bangladesh.

Response: We broadened the limitations to encompass social and structural factors, potential sampling biases, and gendered access barriers. The manuscript now notes that BDHS may under-represent some high-risk or marginalized groups, bounding generalizability; that gender-specific barriers in Bangladesh (e.g., mobility constraints, caregiving burden, norms around clinic attendance) can limit care-seeking and program uptake; and that fairness governance (subgroup performance monitoring, transparent versioning/thresholds, mitigation of unintended harms) is required for responsible use. These additions appear in the Discussion (p.34–35, lines 499–520) and the Strengths and limitations section (page.36–37, lines 545–558), and are grounded by earlier context on the BDHS sampling frame and our weighting rationale Methods—Survey weights, page.10, lines 209–215). We also reiterate cross-sectional and self-report constraints (associations ≠ causation).

Comment: The generalizability section could better address the representativeness not only for married women, but also the implications for unmarried women or other populations left out of the dataset.

Response: We explicitly bound generalizability to the analytic population and spell out who is not covered. The Discussion clarifies that findings reflect married women sampled by BDHS and may not extend to unmarried women or under-represented subgroups; we note potential BDHS under-sampling of marginalized populations and advise fairness monitoring and local threshold recalibration to prevalence and error costs (Discussion, page.34–35, lines 507–513). We pre-specify external validation on BDHS-2022 to test discrimination, calibration, and threshold transfer in a later, post-COVID cohort and

under any instrument changes (Discussion, page.35, lines 523–526). The Strengths and limitations section reiterates these population bounds and sampling caveats (p.36–37, lines 545–558). Earlier context on the BDHS sampling frame and our weighting rationale further anchors representativeness (Methods, page.7–8, lines 143–156; Methods,Survey weights, page .10, lines 209–215).

Comment: Interpretation of the predictive models would benefit from a more detailed discussion of their limitations in distinguishing correlation from causation, especially in feature importance ranking.
Response: We now distinguish correlation from causation explicitly across the manuscript. The Abstract states, "Models quantify associations rather than causation" (page.2, lines 33–34). In Methods, SHAP is framed as a post-hoc interpretability tool not a causal estimator and we note that its attributions reflect the fitted model and observed covariate structure (Methods—Interpretability, page.10–11, lines 199–206). The Discussion reiterates that "SHAP values explain model associations, not causal effects," cautions that correlated social and fertility indicators may function as proxies, and flags counter-intuitive signals (e.g., husbands' secondary education) as hypotheses for follow-up rather than causal claims (Discussion, page.35, lines 514–521). The Strengths and limitations section restates the cross-sectional design and non-causal nature of feature rankings (page.36–37, lines 545–558). We also report sensitivity checks on correlated predictors and emphasize that policy use should follow external validation and, where appropriate, causal or quasi-experimental evaluation.

Comment: Ethical considerations of applying ML methods—like fairness, interpretability, and unintended consequences—should be mentioned.
Response: Ethical considerations are now woven throughout the manuscript. The Introduction frames ML's promise alongside risks, explicitly noting the need for interpretability and fairness in public-health use (page.4, lines 65–73). Methods include an Ethics/Fairness subsection that explains our use of de-identified BDHS data, careful handling of sensitive attributes, reliance on SHAP for transparent explanations, and plans for subgroup performance checks (page.14–15, lines 293–300). The Discussion adds deployment guardrails routine fairness monitoring, transparency via model versioning and documented thresholds, calibration to local prevalence and error costs, and mitigation of unintended consequences such as stigma or resource diversion (page.34–35, lines 507–513). Finally, the Strengths and limitations section reiterates that fairness audits revealed subgroup gaps requiring monitoring and mitigation, and that external validation is a prerequisite for any real-world use (page.36–37, lines 545–558).

Comment: The conclusion could offer a more detailed roadmap for future studies, specifying what improvements or validations (e.g., external datasets, prospective studies) are most urgent.
Response: We now provide a prioritized roadmap for future work: (i) strict external validation on BDHS-2022 using identical preprocessing and the pre-specified decision threshold to assess discrimination, calibration, and transportability; (ii) prospective/temporal validation with local recalibration and threshold tuning to reflect

prevalence and error costs; (iii) enrichment with clinical/biometric markers; (iv) routine fairness audits with subgroup-gap mitigation; (v) extension of modeling to unmarried women and other under-represented groups; (vi) transfer learning and cross-country adaptation; (vii) linkage to real-time eRegistry streams for adaptive monitoring and thresholding; and (viii) exploratory multi-omics integration where feasible. These priorities are detailed in the Conclusion (p.36, lines 534–541) and align with the operational pathways outlined in the Discussion (p.34–35, lines 507–513).

Best regards,
Novel Chandra Das
Research officer, icddrb
