# Supplementary material for: Predicting hypertension and identifying most important factors among married women in Bangladesh using machine learning approach
Source: PLoS One. 2025 Oct 30;20(10):e0335442. doi: 10.1371/journal.pone.0335442 (PMC12574887; doi:10.1371/journal.pone.0335442)
Supplement: Attachment — Submitted filename: Response to Reviewers 1.docx [file pone.0335442.s009.docx]

The Editor,

We are grateful for the thoughtful and constructive comments provided on our manuscript. We have carefully addressed each point raised and made the necessary revisions to improve the clarity, rigor, and reproducibility of our work. Below, we provide a detailed, point-by-point response to each of the comments.

**Academic Editor comments**

Comments: Please include the following items when submitting your revised manuscript:

- A rebuttal letter that responds to each point raised by the academic editor and reviewer(s). You should upload this letter as a separate file labeled 'Response to Reviewers'.
- A marked-up copy of your manuscript that highlights changes made to the original version. You should upload this as a separate file labeled 'Revised Manuscript with Track Changes'.
- An unmarked version of your revised paper without tracked changes. You should upload this as a separate file labeled 'Manuscript'.

**“Response: Thank you for the opportunity to revise our manuscript. In accordance with your instructions, we have submitted the following materials:**

- **Response to Reviewers – A detailed rebuttal letter that addresses each point raised by the academic editor and reviewers.**
- **Revised Manuscript with Track Changes – A marked-up version of the manuscript highlighting all revisions made in response to the reviewers’ comments.**
- **Manuscript – A clean, unmarked version of the revised manuscript without tracked changes.”**

Comments: When submitting your revision, we need you to address these additional requirements.

Please ensure that your manuscript meets PLOS ONE's style requirements, including those for file naming. The PLOS ONE style templates can be found at

<https://journals.plos.org/plosone/s/file?id=wjVg/PLOSOne_formatting_sample_main_body.pdf> and

<https://journals.plos.org/plosone/s/file?id=ba62/PLOSOne_formatting_sample_title_authors_affiliations.pdf>”

**“Response: We have carefully revised the manuscript to ensure full compliance with PLOS ONE's style requirements, including formatting and file naming conventions. Specifically:**

**The manuscript now follows the structure and formatting outlined in the PLOS ONE style templates, including title page formatting, author affiliations, and manuscript body layout.**

**File names have been updated according to PLOS ONE guidelines to ensure clarity and consistency across all submitted materials see line 1-5 , 19-20,22,50,180,300,501,590,639,643,655,659 and 661 (page 1, 2,3,9,14,34,38,40,41 and 42).”**

Comment: Please note that PLOS ONE has specific guidelines on code sharing for submissions in which author-generated code underpins the findings in the manuscript. In these cases, we expect all author-generated code to be made available without restrictions upon publication of the work. Please review our guidelines at <https://journals.plos.org/plosone/s/materials-and-software-sharing#loc-sharing-code> and ensure that your code is shared in a way that follows best practice and facilitates reproducibility and reuse.

**“Response: We appreciate the reminder regarding PLOS ONE’s code sharing policy. In compliance with the journal’s guidelines, all author-generated code used in this study is publicly available on GitHub:** [**https://github.com/NobleNovel/Hypertension_Maternal**](https://github.com/NobleNovel/Hypertension_Maternal)**. The repository includes all relevant Python and R scripts for data processing, analysis, and figure generation. The “Data and Code Availability” section of the manuscript has been updated to include this link and to reflect best practices for reproducibility and reuse, please see line 653-654 (page 41).**

Comment: Thank you for uploading your study's underlying data set. Unfortunately, the repository you have noted in your Data Availability statement does not qualify as an acceptable data repository according to PLOS's standards.

At this time, please upload the minimal data set necessary to replicate your study's findings to a stable, public repository (such as figshare or Dryad) and provide us with the relevant URLs, DOIs, or accession numbers that may be used to access these data. For a list of recommended repositories and additional information on PLOS standards for data deposition, please see <https://journals.plos.org/plosone/s/recommended-repositories>.

**“Response: Thank you for the comment. We have updated The “Data and Code Availability” section accordingly Data availability statement please see line 644-652 (page 41)”**

Comment: In the online submission form, you indicated that “The codes are based on python and R and available from the corresponding author on proper request”.

All PLOS journals now require all data underlying the findings described in their manuscript to be freely available to other researchers, either a. In a public repository, b. Within the manuscript itself, or c. Uploaded as supplementary information.

This policy applies to all data except where public deposition would breach compliance with the protocol approved by your research ethics board. If your data cannot be made publicly available for ethical or legal reasons (e.g., public availability would compromise patient privacy), please explain your reasons on resubmission and your exemption request will be escalated for approval.

**“Response: Thank you for your comments regarding data and code availability. We appreciate the reminder about PLOS ONE’s policies and have taken the necessary steps to ensure compliance.**

**In accordance with the journal’s code sharing guidelines, all author-generated code used in this study is now publicly available on GitHub:** [**https://github.com/NobleNovel/Hypertension_Maternal**](https://github.com/NobleNovel/Hypertension_Maternal)**. The repository includes all relevant Python and R scripts used for data processing, analysis, and figure generation. We have updated the “Data and Code Availability” section of the manuscript to reflect this line 653-654 (page 41), following best practices to support reproducibility and reuse.**

**Regarding data sharing, we have updated The “Data and Code Availability” section accordingly Data availability statement please see line 644-652 (page 41)”**

Comment: Please amend either the abstract on the online submission form (via Edit Submission) or the abstract in the manuscript so that they are identical.

**“Response: Thank you for your comment. We have updated the abstract to ensure that the version in the online submission form matches the one in the manuscript. Please let me know if any further revisions are needed.”**

Comment: Is the manuscript technically sound, and do the data support the conclusions?

The manuscript must describe a technically sound piece of scientific research with data that supports the conclusions. Experiments must have been conducted rigorously, with appropriate controls, replication, and sample sizes. The conclusions must be drawn appropriately based on the data presented.

**“Response: Thank you for your thoughtful evaluation. We affirm that the manuscript presents a technically sound piece of scientific research, and we have taken several steps to ensure that the data robustly support the conclusions.**

**Data Integrity and Availability we have updated The “Data and Code Availability” section accordingly Data availability statement please see line 644-652 (page 41).**

**Code Availability All author-generated code used for data preprocessing, model training, evaluation, and visualization is freely accessible on GitHub:** [**https://github.com/NobleNovel/Hypertension_Maternal**](https://github.com/NobleNovel/Hypertension_Maternal)**. This repository adheres to best practices and ensures that all analyses are fully reproducible. The updated “Data and Code Availability” section line 653-654 (page 41), includes this information.**

**Experimental Rigor We rigorously evaluated 12 machine learning algorithms using six different class-balancing techniques. To address reviewer concerns on methodological transparency:**

**A consolidated performance metrics table (F1-score, AUC-PR, recall, precision, accuracy) for each algorithm-class balancing combination has been added (lines 380 , page 25-27).**

**A separate hyperparameter table documents the model-specific configurations used in the study (lines 363, page 20-24), enhancing reproducibility.**

**Model Justification and Generalizability Extra Trees was selected based on superior performance across multiple metrics. We have clarified this decision and compared it with results from recent studies using XGBoost and others for relevant tasks (lines 514–553, page 35-37). To improve the manuscript’s credibility and contextual relevance, we have included a comparative analysis highlighting dataset differences and modeling approaches.**

**External Validation We acknowledge the current lack of external validation as a limitation. The unavailability of publicly accessible comparable datasets constrained us from validating the model externally. We now explicitly note this in the Discussion section and outline plans to validate the model using the recently released BDHS 2022 dataset in future research line 613-617 (page 39).**

Comment: Have the authors made all data underlying the findings in their manuscript fully available?

The [PLOS Data policy](http://track.editorialmanager.com/CL0/http:%2F%2Fwww.plosone.org%2Fstatic%2Fpolicies.action%23sharing/1/010f0196d37d316e-f1a22221-61e9-42f2-9347-7a81ee15749a-000000/M_WO8oN7Zi0WanlNIjglUsSCgz8yjcmuONC8PvLYOL0=211) requires authors to make all data underlying the findings described in their manuscript fully available without restriction, with rare exception (please refer to the Data Availability Statement in the manuscript PDF file). The data should be provided as part of the manuscript or its supporting information, or deposited to a public repository. For example, in addition to summary statistics, the data points behind means, medians and variance measures should be available. If there are restrictions on publicly sharing data—e.g. participant privacy or use of data from a third party—those must be specified.

**“Response: Thank you for highlighting the importance of data availability in accordance with PLOS policy. In response to this concern, we have taken several steps to ensure full compliance. The dataset is publicly accessible and can be found at the following link:**

**Visit the DHS Program data portal:** [**https://dhsprogram.com/data/**](https://dhsprogram.com/data/)

**Navigate to the dataset page:** [**https://dhsprogram.com/data/dataset/Bangladesh_Standard-DHS_2017.cfm**](https://dhsprogram.com/data/dataset/Bangladesh_Standard-DHS_2017.cfm)**.**

**We have also revised the Data Availability Statement in the manuscript to clearly reflect this update and provide access information line 644-652 (page 41). Additionally, to support transparency and reproducibility, we have uploaded the complete analysis code to GitHub, and this is now referenced in the “Data and Code Availability” section of the manuscript. We believe these measures fully address the concern regarding data accessibility and ensure the manuscript is in full alignment with the PLOS Data Availability policy.”**

**Reviewer #1**

**Comment:** Weaknesses of the Study:
Comment: The provided link does not lead to the dataset which makes it impossible to verify the findings because of insufficient reproducibility.

**“Response: We acknowledge that the previously provided dataset link led to the general DHS dataset access portal and not directly to the BDHS 2017–18 dataset. Since BDHS data are governed by the DHS Program, direct public sharing is restricted. However, researchers can request access by creating a free account and submitting a data use request. To improve reproducibility and transparency, we have:**

**Updated the Data Availability Statement to include explicit access instructions for obtaining the BDHS 2017–18 dataset.**

**Shared all analysis code publicly on GitHub at** [**https://github.com/NobleNovel/Hypertension_Maternal**](https://github.com/NobleNovel/Hypertension_Maternal)**, which includes Python and R scripts for data preprocessing, model training, evaluation, and figure generation.**

Comment : The models were not externally validated, limiting generalizability.

**“Response: We agree that external validation is critical to assess model generalizability. Unfortunately, at the time of this study, no publicly accessible dataset with comparable features to BDHS 2017–18 was available, including the then-unreleased BDHS 2022. We have now:**

**Acknowledged this limitation in the Conclusion section line 613-617 (page 39).**

**Discussed plans to validate the model using the newly released BDHS 2022 dataset in future work**.

Comment: Insufficient rationale for selecting the Extra Tree algorithm over other high-performing models (e.g., XGBoost, Random Forest).

**“Response: Thank you for highlighting the need for a clearer rationale regarding model selection. While we evaluated 12 machine learning algorithms in combination with 6 different class-balancing techniques, the selection of the Extra Trees model was based on a systematic performance comparison across multiple metrics (F1-score, AUC-PR, precision, recall, accuracy) under each sampling strategy. As detailed in the section “Classification efficacy” line 365-379, page 24-25 and new performance metrics table (see lines 381, page 25-26)), Extra Trees consistently demonstrated superior performance across most evaluation criteria, particularly in handling class imbalance and generalization within the training dataset.**

Comment: The dataset link provided (<https://dhsprogram.com/data/dataset_admin/index.cfm>) does not grant direct access to the BDHS 2017–18 data. The authors must clarify access procedures (e.g., registration requirements) or provide an alternative repository link

**“Response: Thank you for pointing this out. We acknowledge that the link previously provided directs to the general dataset access portal of the DHS Program rather than directly to the BDHS 2017–18 dataset. As the BDHS 2017–18 data are owned by a third party (The DHS Program), direct public sharing is restricted. However, the dataset is freely available upon request for registered users. We have now updated the Data and Code Availability Statement to clarify that users must register for a free account on the DHS Program website and submit a data request specifying the BDHS 2017–18 dataset. Once approved, users can download the dataset for research purposes. The updated link and access instructions are included in the revised Data Availability Statement. We hope this clarification resolves the concern regarding data accessibility.”**

Comment: Quantitative results are presented in the study for the 12 tested machine learning algorithms yet a clear academic methodological approach remains inadequate. The authors need to create a single performance metrics table in the main text to display F1-score, AUC-PR, recall, precision, accuracy alongside each algorithm-class-balancing combination. A model-specific table must present all the hyperparameter settings selected in order to guarantee experimental repeatability. This enhanced approach will make results more transparent and allow results comparison while making the research methodological practices stronger.

**“Response: Thank you for this valuable suggestion. We agree that presenting a consolidated performance metrics table and clearly documenting hyperparameter settings will significantly improve the methodological transparency and reproducibility of our study. In response, we have created a comprehensive performance metrics table under the section “Classification efficacy”in the main text that displays the F1-score, AUC-PR, recall, precision, and accuracy for each machine learning algorithm across all class-balancing techniques used see line 365-379, page 24-25 and new performance metrics table (see lines 381, page 25-26),. This allows for straightforward comparison of model performance under different conditions. Additionally, we have included a section “Parameter optimization line 346-362, page 19-20 ” separate model-specific table that lists all hyperparameter settings selected for each algorithm, along with brief descriptions of how they were determined see line 364, page 20-24”**

Comment: The model requires external validation through independent data to validate its ability to generalize accurately. You should document unavailability of verification data while recommending researched methods for future validation experiments.

**“Response: We appreciate the comment regarding the need for external validation to assess the generalizability of the model. We fully acknowledge the importance of validating machine learning models using independent datasets to ensure robustness and real-world applicability. At the time this study was conducted, the BDHS 2022 dataset had not yet been released, and no publicly accessible independent datasets with comparable features to BDHS 2017–18 were available for external validation.**

**Looking forward, we plan to evaluate our model using the BDHS 2022 data for next version of model.”**

Comment: The author demonstrates why their Extra Trees model performed best by exploring features regarding parameter optimization and variable relationships. The research credibility can be improved by comparing results from equivalent studies which employed XGBoost in hypertension prediction.

**“Response: Thank you for this insightful comment. We agree that situating our findings within the context of existing research can strengthen the credibility and relevance of our study. In response, we have expanded the discussion section to include a comparison with recent studies that employed XGBoost for hypertension prediction. Specifically, we highlight performance metrics such as accuracy, F1-score, and AUC reported in those studies and compare them to the results obtained in our work. We also discuss differences in dataset characteristics, feature sets, and class balancing approaches that may account for performance variations. This comparative analysis not only reinforces the strengths of our Extra Trees model but also provides a clearer understanding of its relative performance and potential advantages in similar contexts. Please see line 514-553, page 35-37”**

Comment: Standardize terms (e.g., use "class-balancing techniques" instead of variations like "class-balanced techniques").

**“Response: Thank you for pointing this out. We have carefully reviewed the manuscript and standardized the terminology throughout, consistently using the term "class-balancing techniques" to ensure clarity and consistency. This change improves the readability and professionalism of the manuscript please see line 29,176,257,282,395,401,404,412,425,430,503,582,626 (page 2,8,12,13,28,29,31,34,38, and 40)”**

Comment: Ensure figures (e.g., SHAP plots) are labeled clearly, with legible font sizes and color contrasts for accessibility.

**“Response: Due to limitations in re-running the analysis, we have enhanced the clarity of the existing SHAP plot by adjusting the resolution, font size, and labeling using graphic editing tools. The revised figure now conforms to PLOS’s formatting guidelines (TIFF format, ≥600 DPI, RGB color mode). Additionally, we have clarified the color coding for accessibility in both the figure legend and caption.**

**We have also included the following comment, as requested:
“While revising your submission, please upload your figure files to the Preflight Analysis and Conversion Engine (PACE) digital diagnostic tool,** [**https://pacev2.apexcovantage.com/**](https://pacev2.apexcovantage.com/)**. PACE helps ensure that figures meet PLOS requirements. To use PACE, you must first register as a user. Registration is free. Then, login and navigate to the UPLOAD tab, where you will find detailed instructions on how to use the tool. If you encounter any issues or have any questions when using PACE, please email PLOS at figures@plos.org. Please note that Supporting Information files do not need this step.”**

Comment: Minor errors exist (e.g., "infectundity" → "infecundity," ). Perform thorough proofreading.

**“Response: Thank you for pointing out these minor errors. We have carefully proofread the entire manuscript and corrected all typographical and spelling mistakes, including changing “infectundity” to “infecundity.” This thorough review has improved the overall clarity and quality of the manuscript.”**

**Reviewer #2**

Comment: The author has not used the latest Bangladesh Demographic and Health Survey 2022 (URL: <https://www.dhsprogram.com/pubs/pdf/FR386/FR386.pdf>). Is there any special reason? Please respond and justify.

**“Response: Thank you for bringing this to our attention. When we conducted our analysis, the most recent publicly available dataset was the Bangladesh Demographic and Health Survey 2017–18; the 2022 survey data had not yet been released or made accessible for research purposes. We acknowledge that using the 2022 data could improve the relevance and accuracy of our findings. Therefore, we plan to incorporate the 2022 dataset in the next version of our model to update and validate its performance. This will be a key focus in our future work.”**

Comment: The author have used data for 4,253 married women in Bangladesh. As we are aware that to assess the predictive power of machine learning algorithms, a sufficiently large sample is required.

**“Response: Thank you for this important observation. We acknowledge that sample size is a crucial factor in the training and evaluation of machine learning models. The dataset of 4,253 married women from BDHS 2017–18 represents the most complete and relevant data available for our study population, with carefully selected features and sufficient variability to support model development. Additionally, we applied robust validation techniques, including cross-validation and class-balancing methods, to enhance the reliability of our results despite the sample size.**

**Furthermore, Previous studies have demonstrated that good predictive performance can be achieved even with smaller sample sizes than ours.”**

Comment: Please keep the format and use of comma (,) properly throughout the text such as Row Number 154, 214, 218, 395, 482 etc.

**“Response: Thank you for highlighting the issues with comma usage and formatting. We have carefully reviewed the manuscript and corrected all instances to ensure proper and consistent use of commas throughout the text, including in the specified rows. This revision improves the readability and professionalism of the manuscript p-lease see line 151,211,213,215,217,218,219,220,221,222,223,224,225,226, 426 page 7,11,12,31.”**

Comment: Provide heading t table 2 and avoid repeatedly mentioning A and p-values within the table.

**“Response: Thank you for the helpful suggestion. We have added a clear and descriptive heading to Table 2 to improve its clarity. Additionally, we have streamlined the presentation by avoiding repetitive mentions of “A” and p-values within the table, making it easier to read and interpret. Please see line 405, page 28”**

Comment: Avoid repeatedly mentioning chi-squared, df and p-values in table 5.

**“Response: Thank you for the valuable suggestion. We have revised Table 5 to avoid repetitive mentions of chi-squared values, degrees of freedom (df), and p-values by consolidating these statistics where appropriate. This enhances the table’s clarity and readability without compromising the presentation of important statistical information. Please see line 420, page 29”**

Comment: The conclusion part of the study is very short. It should be explanatory and exhaustive in nature.

**“Response: Thank you for this valuable feedback. We have expanded the conclusion section to provide a more detailed and comprehensive summary of our key findings, their implications, and potential applications. The revised conclusion also highlights the strengths and limitations of the study, as well as recommendations for future research, making it more explanatory and exhaustive as suggested. See line 591-623 , page 38-40 ”**

Comment: While revising your submission, please upload your figure files to the Preflight Analysis and Conversion Engine (PACE) digital diagnostic tool, [https://pacev2.apexcovantage.com/](https://track.editorialmanager.com/CL0/https:%2F%2Fpacev2.apexcovantage.com%2F/1/010f0196d37d316e-f1a22221-61e9-42f2-9347-7a81ee15749a-000000/H6ZwkK6WbyDOCCdyVfX9fGl3FezcM9ZXHAyrYRLCY0c=211). PACE helps ensure that figures meet PLOS requirements. To use PACE, you must first register as a user. Registration is free. Then, login and navigate to the UPLOAD tab, where you will find detailed instructions on how to use the tool. If you encounter any issues or have any questions when using PACE, please email PLOS at [figures@plos.org](mailto:figures@plos.org). Please note that Supporting Information files do not need this step.

**“ Response: Thank you for the guidance regarding figure preparation. We have registered with the PACE digital diagnostic tool and uploaded all figure files as instructed to ensure they meet PLOS requirements. We carefully reviewed the outputs and made any necessary adjustments to improve figure quality and compliance. We appreciate this resource for helping us enhance the clarity and presentation of our figures and uploaded the figures accordingly.”**

Best regards,

Novel Chandra Das

Research officer, icddrb
