# Supplementary material for: Predicting hypertension and identifying most important factors among married women in Bangladesh using machine learning approach
Source: PLoS One. 2025 Oct 30;20(10):e0335442. doi: 10.1371/journal.pone.0335442 (PMC12574887; doi:10.1371/journal.pone.0335442)
Supplement: Attachment — Submitted filename: Plos One.docx [file pone.0335442.s010.docx]

**Evaluating machine learning algorithms and identifying factors associated with hypertension among married women in Bangladesh**

**Abstract**

- The abstract’s language is not fully polished (e.g., “we aimed” is not capitalised, and some sections are missing necessary punctuation), affecting professionalism.
- The results use absolute F1 values (from 8 to 94), but do not mention what the baseline scores for other models were, and the abstract’s comparative value is limited without context.
- The abstract does not distinguish between statistical associations and causal inferences; by design, machine learning models show correlation, not causation.
- The abstract mentions "top 20 factors," but explores only a few, leaving their full list and ranking method unclear.
- The conclusion focuses on the machine learning aspect without summarising wider public health implications or practical impact, missing an opportunity to discuss policy or clinical relevance.
- The abstract omits main limitations (e.g., no external dataset validation, as discussed in the manuscript). Potential data access and generalizability barriers, as highlighted by reviewers, are not addressed up front.

**Introduction**

- The introduction briefly mentions machine learning but could elaborate more on the advantages, limitations, and previous gaps in traditional statistical models for hypertension risk prediction.
- There is an opportunity to better synthesise existing regional studies and clearly indicate how this research advances previous efforts, addressing what is new in methodology or population.
- At times, the introduction contains long sentences that could be better segmented for clarity and flow.
- Explicitly state whether this study is the first in Bangladesh to use such an extensive set of algorithms and class balancing approaches for hypertension prediction in married women, highlighting its unique contributions.
- Address limitations or challenges posed by the dataset (e.g., self-reported data, cross-sectional nature) up front to set proper expectations.
- Consider including a brief mention of ethical considerations when using big data or advanced analytics in public health research.
- The transition into the objectives section could be improved for more seamless narrative connection between context and study aims.
- Some terminology (e.g., differentiating predictive versus explanatory modeling) can be clarified to aid a broad audience.

**Methodology**

- The lack of validation using independent/external data is a key limitation. While acknowledged, this impacts model generalizability beyond the sampled population. Future work with the now-available BDHS 2022 should be prioritised.
- While a wide set of features is included, the methodology could elaborate whether feature selection (beyond literature-based inclusion) was performed before modeling, possibly using wrapper/filter/embedded methods.
- The methodology briefly mentions deletion but does not elaborate on the extent or potential bias induced by this approach versus imputation.
- Training was performed on basic hardware (i5 CPU, 8GB RAM)—potentially limiting more computationally intensive models or hyperparameter searches.
- No mention of use of survey/sample weights, which are critical for BDHS data to ensure national representativeness. Analytical weighting should be addressed or justified if omitted.
- While IRB considerations are reported, the rationale and implications of using advanced ML (including fairness or bias issues) could be further discussed under ethical considerations.
- Rationale for preferring Extra Trees, as opposed to XGBoost/Random Forest (other top performers in similar studies), could be more clearly articulated within the methods section, not just in the discussion.
- Clearly define composite or derived variables (e.g., explain calculation for BMI and diabetes status) in a dedicated subsection for reproducibility.

**Results**

- The results could benefit from consolidation, such as combining tables and figures for easier cross-comparison between different algorithms and balancing techniques.
- Presenting confidence intervals or statistical uncertainty for key metrics (not only point estimates) would strengthen result reporting and allow readers to assess model reliability.
- The narrative sections of the results could be more concise and focused, with clearer guidance to the reader on the most clinically or policy-relevant findings.
- Visualisation of SHAP values (e.g., summary plots) and confusion matrices should be clearer and referenced in the main results narrative, rather than relegated to supplementary materials.
- Greater emphasis on findings that differ from previous studies, especially regarding novel risk/protective factors found important by the chosen machine learning models.
- The discussion of limitations in the results section should be expanded: for example, how the internal validation-only approach may exaggerate performance (potential overfitting).
- More explicit tracking and reporting of sample sizes for each analysis (post-imputation, balancing) would enhance transparency.

**Discussion**

- The discussion could more robustly address how findings might translate to policy or clinical practice, with specific recommendations or scenarios.
- Limitations could be expanded beyond technical aspects: for example, discuss the impact of cultural/social determinants, potential biases in BDHS sampling, or gender-specific barriers to care in Bangladesh.
- The generalizability section could better address the representativeness not only for married women, but also the implications for unmarried women or other populations left out of the dataset.
- Interpretation of the predictive models would benefit from a more detailed discussion of their limitations in distinguishing correlation from causation, especially in feature importance ranking.
- Ethical considerations of applying ML methods—like fairness, interpretability, and unintended consequences—should be mentioned.
- The conclusion could offer a more detailed roadmap for future studies, specifying what improvements or validations (e.g., external datasets, prospective studies) are most urgent.
