# Supplementary material for: Predicting hypertension and identifying most important factors among married women in Bangladesh using machine learning approach
Source: PLoS One. 2025 Oct 30;20(10):e0335442. doi: 10.1371/journal.pone.0335442 (PMC12574887; doi:10.1371/journal.pone.0335442)
Supplement: Attachment — Submitted filename: Response to reviewers comments v2.docx [file pone.0335442.s011.docx]

The Editor,

We are grateful for the thoughtful and constructive comments provided on our manuscript. We have carefully addressed each point raised and made the necessary revisions to improve the clarity, rigor, and reproducibility of our work. Below, we provide a detailed, point-by-point response to each of the comments.

**Academic Editor comments**

Comments: Please include the following items when submitting your revised manuscript:

- A rebuttal letter that responds to each point raised by the academic editor and reviewer(s). You should upload this letter as a separate file labeled 'Response to Reviewers'.
- A marked-up copy of your manuscript that highlights changes made to the original version. You should upload this as a separate file labeled 'Revised Manuscript with Track Changes'.
- An unmarked version of your revised paper without tracked changes. You should upload this as a separate file labeled 'Manuscript'.

**“Response: Thank you for the opportunity to revise our manuscript. In accordance with your instructions, we have submitted the following materials:**

- **Response to Reviewers – A detailed rebuttal letter that addresses each point raised by the academic editor and reviewers.**
- **Revised Manuscript with Track Changes – A marked-up version of the manuscript highlighting all revisions made in response to the reviewers’ comments.**
- **Manuscript – A clean, unmarked version of the revised manuscript without tracked changes.”**

**Reviewers' comments:**

**Reviewer #4:** Detailed Comments
Comment: Title and Abstract
The title is clear and descriptive, effectively capturing the dual focus on evaluating ML algorithms and identifying hypertension factors. It could perhaps be tightened for conciseness, something like "Machine Learning Approaches to Predict and Rank Hypertension Risk Factors Among Married Women in Bangladesh" – but that's minor.
The abstract is generally well-structured, summarizing the introduction, methods, results, and conclusion. I like how it highlights key metrics (e.g., F1 score jumping from 8% to 94% with SMOTE+ENN) and the top positive/negative factors from SHAP. However, there are a few awkward phrasings that make it feel a bit rough – for instance, "we aimed to develop a machine learning model to identify and rank predictive factors" could be more precise as "we developed..." since it's a completed study. Also, the prevalence stats (76.9% normal BP vs. 23.1% hypertensive) are useful, but tying them to broader implications right away might help. Grammatically, "infectundity" in the methods section should be "infecundity" (noted in responses to reviewers, but check if it's fixed). Overall, it's informative, but revise for smoother flow and ensure it matches the submission form version exactly, as flagged by the journal.

**“Response: Thank you for the helpful suggestions. We have polished the Title and Abstract for concision, consistency and clarity. The title now reads “Predicting Hypertension and Identifying most important Factors among Married Women in Bangladesh using Machine Learning Approach.” (page. 1, lines 1–2). The Abstract is uniformly in past tense and opens with our completed work: “We developed and interpreted machine-learning (ML) models …” (page. 2, lines 24–26). To contextualize performance gains, we contrast baseline vs. balanced results: F1 = 0.08, recall = 0.04 (imbalanced baseline) → F1 = 0.94, recall = 0.95, AUC-PR = 0.95, ROC-AUC = 0.95 (p. 2, lines 34–37). We state the associational scope (“Models quantify associations rather than causation,” page. 2, line 33) and note next steps (external validation on BDHS-2022, page. 3, lines 47–48). We also articulate programmatic implications (e.g., eRegistries, FP/postpartum screening, spousal education/SMS, district-tailored outreach; page. 3, lines 45–47). The typo “infectundity” → “infecundity” is corrected (page. 8, line 174). Finally, we verified that the Abstract matches the submission-form version exactly.**

Comment: Introduction
The introduction sets up the problem nicely, starting with global hypertension stats and narrowing to Bangladesh, with a focus on women and married subgroups. I appreciate the literature review on ML applications in health outcomes – it's comprehensive, covering everything from SVMs in CHD prediction to SHAP's advantages over LASSO/ANOVA. References seem up-to-date and relevant, like the emphasis on tree-based models outperforming logistic regression.
That said, it feels a tad lengthy in places, with some repetition (e.g., multiple paragraphs on ML superiority). The knowledge gap – why married women specifically? – is stated, but could be backed with more evidence on cultural/economic factors in Bangladesh. Also, since the current date is 2025, mentioning why 2017-18 data was used over the 2022 BDHS (as addressed in reviewer responses) should be integrated here for transparency. In my opinion, this section is strong but could be streamlined to about 80% of its length to keep the reader engaged.

**“Response: Thank you for the constructive suggestions. We have streamlined the “Introduction” to remove redundancy particularly around statements of ML superiority and tightened the narrative (page. 3–7, lines 52–135). First, we document the national burden and sex disparity in Bangladesh (adults ≥35 years; higher prevalence in women) with nationwide references (page. 3, lines 54–61). Second, we frame marriage as a risk context by citing studies showing higher hypertension among married versus unmarried/never-married women (page. 3, lines 62–64). Third, we add Bangladesh-specific cultural and economic context reproductive roles, domestic workloads, and socioeconomic constraints grounded in relevant studies to explain married women’s elevated risk (p. 6, lines 118–124). Finally, for transparency about data vintage, we clarify that BDHS-2022 was released after our analysis; accordingly, we trained on BDHS-2017–18 and pre-specified external validation on BDHS-2022 (page. 6, lines 124–126).”**

Comment: Methods
This is one of the stronger sections, with detailed descriptions of data source (BDHS 2017-18, n=4,253), preprocessing, and analysis. The choice of 12 algorithms (e.g., Extra Trees, XGBoost, SVM) and 6 balancing techniques (SMOTE, ADASYN, etc.) is justified well for handling imbalance. Validation methods (hold-out + repeated stratified k-fold) and hyperparameter tuning via random search are appropriate, and the evaluation metrics (F1, AUC-PR, etc.) suit imbalanced data. SHAP for feature importance is a great addition for interpretability.
However, a few clarifications are needed. How were features selected initially? The manuscript mentions demographic/clinical vars, but no explicit feature engineering or selection (e.g., Boruta or LASSO, as hinted in intro). Sample size justification is weak – 4,253 is decent, but discuss power for ML, especially with imbalance. Ethical considerations are covered, but confirm if any sensitive vars (e.g., religion) were handled with care. Hardware/software details are fine, but the GitHub repo for code is a plus – ensure it's fully reproducible. I'd suggest adding a flowchart for the workflow to make it easier to follow.

**Response: Thank you for the detailed suggestions. We have clarified variable handling, sample-size rationale for imbalanced ML, workflow presentation and ethics/reproducibility. Specifically, we now state that all sociodemographic, behavioral, biometric and anthropometric variables with theoretical/empirical relevance were included without automated feature selection (e.g., Boruta/LASSO/RFE), guided by prior BDHS hypertension literature (page. 10, lines 199–203). Furthermore, previously we mentioned that the variables are considered from the existing literature (page 8-9,lines 163-197). We added a sample-size justification tailored to imbalanced classification and our observed prevalence (page. 8, lines 157–160). Derived variables are specified (diabetes thresholds, page. 9, lines 188–194; BMI categories, page. 9, lines 195–197). A study workflow statement and flowchart was added (“An overview of the analytical workflow from data extraction and preprocessing through class balancing, model training, hyperparameter tuning, validation and evaluation, followed by statistical analysis to compare class-balancing techniques and model performance and SHAP-based interpretation is presented in Fig 2. This schematic provides a concise visual summary of the methodological pipeline described in the preceding subsections.**

**Fig 2. Workflow of machine learning pipeline for hypertension prediction and risk factor ranking of married women in Bangladesh” page. 15, lines 301–308).**

**Regarding sensitive attributes: religion was included as recorded in the BDHS public-use dataset, which is de-identified and governed by DHS Program ethical protocols and confidentiality safeguards. We handled this variable with care used only as a covariate for population-level modeling, reported in aggregate, and not for individual targeting or decision rules consistent with the IRB/ethical approvals and BDHS data-use conditions.”**

Comment: Results
The results are presented logically, starting with descriptive stats (Table 1 is comprehensive, showing hypertension stratification by demographics). Prevalence of 23.1% aligns with prior studies, and breakdowns (e.g., higher in Rangpur, urban areas) add context. Model performances are detailed, with Extra Trees + SMOTE+ENN shining (F1=94%, big improvement from baseline). SHAP plots (Figs 11a-c) effectively rank factors – age <35 negative, overweight positive, etc. – and the directionality (blue/red bars) is intuitive.
But some issues: Tables 4-8 on stats tests (Anderson-Darling, Friedman) are dense; consider consolidating or moving non-essential to supp. Figures need better labels – e.g., ensure font sizes are legible and color contrasts accessible (as per reviewer comments). No mention of overfitting checks beyond CV scores. Also, while top 20 factors are screened, discuss if multicollinearity (e.g., age and parity) was addressed. Overall, solid but polish the presentation for clarity.

**“Response: Thank you for the constructive feedback. We streamlined the Results and strengthened clarity, accessibility and robustness. We consolidated omnibus significance outputs into one main table and moved detailed Anderson–Darling/Friedman statistics to the Supplement (former Tables 4–8 → S4 Appendix → Supplementary Table S2 (S2A–S2E)). Figures were relabeled with larger type, clearer panel markers and self-contained captions; we adopted a color-blind–safe palette, improved contrast to meet accessibility guidelines and verified print-scale legibility via the journal’s PACE checks. To address overfitting, beyond cross-validation we report training–validation deltas, calibration (reliability curves, Brier scores) and learning-curve behavior; preprocessing was nested within folds to prevent leakage. Repeated stratified five-fold CV yielded an average F1 of 0.934 ± 0.012 and nested CV showed an outer-fold mean F1 of 0.965 ± 0.010 (page. 30, 32, lines 425–430 and 454-459), indicating stable generalization. We also added explicit Methods and Results notes on multicollinearity consistent with our analysis. In Methods, we report assessing variance inflation factors (VIF) and pairwise correlations; as expected with one-hot–encoded categorical variables, some VIFs were infinite due to perfect linear dependence among dummy indicators. Because our primary models are gradient-boosted decision trees, which are robust to correlated inputs, this did not compromise predictive validity. We also inspected Spearman correlations among key socio-demographic and fertility-related predictors (page. 14, lines 280-286). In Results, we document structurally inflated VIFs under one-hot encoding and the anticipated dependencies e.g., respondent age <35 vs. husband age ≥40 (ρ = −0.82) and mutually exclusive recent-birth categories (ρ = −0.89) as well as correlations across BMI categories, parity, and living-children counts. These reflect structural collinearities inherent to categorical coding; tree-based models (e.g., ExtraTrees) are robust to such correlations and predictive performance was unaffected (Supplementary Table S5; Results, page. 31, lines 439–445).”**

Comment: Discussion
The discussion ties results back to literature effectively, comparing to studies like Asadullah et al. (hybrid model at 78% accuracy vs. yours at 91%) and others using XGBoost. It highlights strengths: better performance in imbalanced data, context-specific factors for married women. Limitations are acknowledged (no external validation, old data), with plans for BDHS 2022 – good, but expand on how this affects generalizability (e.g., post-COVID changes in health behaviors?).
I think it could delve deeper into implications – e.g., how factors like spousal education inform policy (education campaigns for men?). The SHAP insights are under-discussed; why might secondary education for husbands increase risk? (Perhaps socioeconomic stress?) Avoid overclaiming – e.g., "outperformed other models" is fine, but quantify vs. benchmarks. End with future directions, like integrating real-time data or multi-omics.
Add these recent studies as references to your bibliography: DOI: 10.1007/s40200-020-00536-x, DOI: 10.1016/j.numecd.2021.09.029

**“Response: Thank you for these suggestions. We have strengthened the Discussion to (i) quantify gains versus benchmarks, (ii) deepen SHAP interpretation, (iii) spell out policy uses and (iv) clarify generalizability and future work. We now quantify performance against prior work (“AUC-ROC of 0.95 exceeds large clinical models by +0.08–0.17; accuracy is +13 points vs. Asadullah et al., 91% vs. 78%”; see page. 33, lines 482–485). We justify emphasizing PR over ROC in imbalanced settings (“F1 and AUC-PR provide a truer picture when positives are rare”; see page. 34, lines 486–488). We interpret SHAP signals, noting the unexpected positive association with husbands’ secondary education and plausible mechanisms (socioeconomic stressors; wealth/occupation; fertility) and flag this for follow-up (see page. 34, lines 499–501). We translate findings into actions, integrating BP checks into FP/postpartum workflows, engaging husbands via brief education/SMS where partner age/education indicate risk and prioritizing home BP monitoring in high-parity households; district gradients motivate tailored outreach (see page. 34–35, lines 507–510). For generalizability, we note pre-specified external validation on BDHS-2022 to test transportability in a later, post-COVID cohort (discrimination, calibration, threshold transfer; see page. 35, lines 523–526). For robustness, we report training–validation deltas, calibration (reliability and Brier) and nested cross-validation with leakage controls (see page. 35, lines 521–523). We also added the requested citations on diet strategies—green coffee (DOI: 10.1007/s40200-020-00536-x) and DASH/Mediterranean patterns (DOI: 10.1016/j.numecd.2021.09.029; see page. 35, lines 510–513).” Furthermore we added consecutive future pat (see page 36, lines 534-541)**

Comment: Conclusion
The conclusion recaps key findings concisely: Extra Trees + SMOTE+ENN best, lists top factors. It emphasizes ensemble methods' potential for imbalanced data, which is spot-on.
However, it's a bit short and repetitive of the abstract. Expand to discuss broader impacts (e.g., public health applications in LMICs) and reiterate limitations briefly. As per reviewer #2, make it more explanatory – e.g., how this model could aid screening programs.

**“Response: We now close with concrete operational pathways and a clear validation prerequisite: “The best-performing (Extra Trees + SMOTE+ENN) model and SHAP insights can be operationalized through eRegistries, family-planning/postpartum screening, and targeted spousal outreach, pending external validation”. To address broader LMIC relevance while keeping the Conclusion explanatory (not duplicative of the Abstract), we add that implementation will require threshold calibration to local prevalence, workload/resource planning, and fairness monitoring. We also reiterate key limitations associational design, focus on married women, and the need for external validation on BDHS-2022 so readers understand scope and generalizability (Conclusion, page. 36–37, lines 528–558).”**

General Comments
Comment: Figures and Tables: Mostly clear, but ensure PACE compliance for figures (as noted). Table 1 is excellent; others could use consistent formatting (e.g., avoid repeating stats in Table 5).

**Response: We harmonized figure/table references and verified font sizes and contrast per PACE and the Results text now explicitly directs readers to the consolidated performance and diagnostic blocks (“Figures were adjusted for font size and contrast as per PACE; table/figure references are harmonized across Results”; page. 25, lines 367–378; page. 20, lines 352–363; page. 27–28, lines 388–403; page.30,32, lines 426-430,455-459). Cross-references now point directly from the narrative to the consolidated performance table and the confusion-matrix block and accessibility edits are documented in-text so reviewers can connect the readability updates to the exact evidence blocks. To ensure consistent formatting (including avoiding repeated statistics in Table 5), we standardized table styles across the section and moved non-essential tables to the Supplement (page. 19, 28, 30–32; lines 342–350, 388–396, 426–430, 440–445, 446–463). We also relabeled and re-uploaded all figures after making PACE-compliant adjustments.**

Comment: Writing and Language: Mostly good, but some typos/awkward sentences (e.g., "we aimed" in abstract should be past tense). Proofread thoroughly – e.g., "infectundity" fixed? Use consistent terms like "class-balancing" throughout.

**Response: The Abstract is now fully in the past tense (“We developed and interpreted …”; page. 2, lines 24–26) and we use “class-balancing” consistently across Methods/Results (e.g., page. 2, lines 28–29; page. 3, line 50; page. 6, line 130; page. 10, line 212; page. 13, line 261; page. 15, lines 302–304; page. 19, lines 341, 343, 349; page. 25, line 379; page. 26, lines 384, 387; page. 28, lines 400–403; page. 29, line 450; page. 32, line 465; page. 33, lines 528, 543, 546, 552; page. 45, line 901). Performance reporting in the Results is cited at page. 30, lines 425–430 and page. 32, lines 454–459. The typo “infectundity” has been corrected to “infecundity” (p. 8, line 174).**

Comment: References: Comprehensive (over 100), but check for recency – some pre-2020; add if newer ML-hypertension studies exist.

**Response: We audited and refreshed recency across the manuscript and references, adding 2021–2025 ML/SHAP and hypertension studies and integrating them into the narrative where they inform model choice, imbalance-aware metrics, and interpretability. Specifically, new ML-hypertension and benchmarking citations (e.g., Value in Health 2025 (ref. 18), Scientific Reports 2024 HUNT model (ref. 36), Clin Exp Hypert 2025 (ref. 99), J Hum Hypertens 2025 wearable ECG (ref. 100), EPJ Special Topics 2025 QML (ref. 102), plus Bangladesh-focused updates (refs. 69–72, 75, 96)) support comparative performance statements in the Discussion (p. 33, lines 482–485; p. 34, lines 487–488, 499–501) and strengthen context. We also inserted recent SHAP/feature-selection and explainability work (refs. 59–68) alongside our interpretation passages, and added contemporary sample-size/power guidance for tabular clinical ML (refs. 76–78) to the Methods justification (p. 8, lines 157–160). The two diet-related DOIs requested by the reviewer are now cited in the policy/implications sentence green coffee meta-analysis (ref. 117; DOI: 10.1007/s40200-020-00536-x) and DASH/Mediterranean (ref. 118; DOI: 10.1016/j.numecd.2021.09.029)—at Discussion, p. 35, lines 510–513. Together, these updates raise the overall currency of the bibliography while preserving numbering consistency and directly linking new evidence to the affected text blocks**

Comment: Reproducibility: GitHub code is a strength, but confirm data access instructions are precise.

**Response: We revised Data and code availability to provide precise, step-by-step DHS access and reproducibility details consistent with third-party restrictions. The subsection now clarifies that BDHS 2017–18 data are owned by the DHS Program and cannot be redistributed by the authors; access is free upon registration and approval (typically 1–2 business days), with explicit instructions to visit the DHS data portal and request the Bangladesh 2017–18 Standard DHS dataset (page. 37-38, lines 566–572).**

Comment: Originality and Significance: Novel in focusing on married women with SHAP; contributes to ML in global health. But update with 2022 data for timeliness.

**Response: We foreground originality and timeliness by stating, “To our knowledge, this is the first Bangladesh study focused on married women combining class-balancing with SHAP explanations,” and we specify external validation on BDHS-2022 as the next step (page. 6 and 13 lines 129–131 and 265-272). This placement in the Introduction (novelty) is paired with a concise future-work note in the Discussion to address recency and generalizability, explicitly acknowledging post-COVID shifts and outlining the BDHS-2022 transportability test (Discussion, page. 35, lines 523–526)**

Comment: While revising your submission, please upload your figure files to the Preflight Analysis and Conversion Engine (PACE) digital diagnostic tool, [https://pacev2.apexcovantage.com/](https://track.editorialmanager.com/CL0/https:%2F%2Fpacev2.apexcovantage.com%2F/1/010f01992723bb10-81ecfbe2-6e01-4859-82f5-ecb41ea82f46-000000/Iqc3zKuXWA3SKCegthxiECCJ-qkl7WNyeo8O-tEtcP8=227). PACE helps ensure that figures meet PLOS requirements. To use PACE, you must first register as a user. Registration is free. Then, login and navigate to the UPLOAD tab, where you will find detailed instructions on how to use the tool. If you encounter any issues or have any questions when using PACE, please email PLOS at [figures@plos.org](mailto:figures@plos.org). Please note that Supporting Information files do not need this step.

**Response: All figures will be uploaded to PACE before final submission. Internally, we harmonized contrast and font sizing and made table/figure cross-references explicit in the Results narrative .**

**Response to Editorial Attachment – PLOS ONE (“Plos One”) Word Comments**

**Evaluating machine learning algorithms and identifying factors associated with hypertension among married women in Bangladesh**

**Abstract**

Comment: The abstract’s language is not fully polished (e.g., “we aimed” is not capitalised, and some sections are missing necessary punctuation), affecting professionalism.

**Response: We rewrote the Abstract in the past tense and tightened punctuation and flow. The opening now reads: “We developed and interpreted machine-learning (ML) models to predict hypertension and rank associated factors among married women …” (page.2, lines 24-25), replacing the earlier “we aimed” phrasing and aligning with a completed study. This edit standardizes capitalization/punctuation, removes prospective language and aligns tone with completed work, addressing professionalism and consistency concerns flagged by the journal (page 2-3,lines 23-48).**

Comment: The results use absolute F1 values (from 8 to 94), but do not mention what the baseline scores for other models were, and the abstract’s comparative value is limited without context.

**Response: We added explicit baseline-to-balanced context in the Abstract—“The Extra Trees classifier with SMOTE+ENN achieved the best discrimination (F1 = 0.94; AUC-PR = 0.95; ROC-AUC = 0.95). Compared with the original imbalanced training, minority-class detection improved substantially …” (page. 2, lines 34–37). Given PLOS ONE’s word-limit for abstracts, we report the full cross-model baselines and balanced scores in the Results narrative (page. 25, lines 367–378) and consolidate per-algorithm metrics (baseline vs. post-resampling across 12 ML algorithms and six class-balancing methods) in Table 5 (page. 26, line 384).**

Comment: The abstract does not distinguish between statistical associations and causal inferences; by design, machine learning models show correlation, not causation.

**Response: We now make this explicit: “Models quantify associations rather than causation.” (page.2, line 33).**

Comment: The abstract mentions "top 20 factors," but explores only a few, leaving their full list and ranking method unclear.

**Response: We clarified the method and pointed to the full list while keeping the Abstract concise. The Abstract now states: “To interpret results, SHAP was used to rank the top 20 predictors and visualize feature effects” (page. 2, lines 32–41) but, due to word limits, does not enumerate all twenty. The complete ranked list and directions of association are provided in the Results with explicit figure call-outs: Fig. 12a (importance order), Fig. 12b (top-20 with positive/negative direction), and Fig. 12c (summary plot of effects), as referenced in text (page. 29–30, lines 406–421)**

Comment: The conclusion focuses on the machine learning aspect without summarizing wider public health implications or practical impact, missing an opportunity to discuss policy or clinical relevance.

**Response: We added programmatic implications directly in the Abstract: “Findings support programmatic integration of risk scores into eRegistries, routine blood pressure checks in family planning and postpartum visits, husband-focused education/SMS interventions and prioritization of high-parity households in high-risk regions.” (page.3, line 44-47). This deployment language was not present previously.**

Comment: The abstract omits main limitations (e.g., no external dataset validation, as discussed in the manuscript). Potential data access and generalizability barriers, as highlighted by reviewers, are not addressed up front.

**Response: We now flag external validation as a priority in the Abstract: “External validation on BDHS-2022 is planned to assess generalizability.” (page.3, line47-48). This places the limitation up front and points to the immediate next step.**

**Introduction**

Comment: The introduction briefly mentions machine learning but could elaborate more on the advantages, limitations, and previous gaps in traditional statistical models for hypertension risk prediction.

**Response: We inserted a concise pros/cons paragraph that recognizes both ML’s strengths and limits for public health adoption: “… ML methods can process high-dimensional data … capture nonlinear relationships … At the same time, ML faces limitations … Addressing interpretability and ensuring fairness remain essential …” (page.4, lines 67-73). This balanced framing replaces earlier diffuse statements and anchors the ensuing methods.**

Comment: There is an opportunity to better synthesise existing regional studies and clearly indicate how this research advances previous efforts, addressing what is new in methodology or population.

**Response: We strengthened the Introduction to synthesize regional ML–hypertension evidence and to make our advance explicit in both population and method. We summarize prior South Asian studies and their recurrent limitations general adult samples, clinical-marker emphasis and scarce subgroup validation then motivate our Bangladesh-specific focus on married women using sociocultural and economic determinants (page. 6, lines 118–123). We state what is new: to our knowledge, this is the first Bangladesh study to evaluate an extensive suite of algorithms with class-balancing and SHAP interpretability specifically for married women (page. 7, lines 129–131), trained on BDHS-2017–18 with pre-specified external validation on BDHS-2022 to ensure transparent assessment of generalizability (page. 6, lines 124–126). We also tie this novelty to programmatic use-cases targeted screening within FP/postpartum services and eRegistries—so the contribution over prior regional work is clear and actionable (page. 7, lines 131–135).**

Comment: At times, the introduction contains long sentences that could be better segmented for clarity and flow.

**Response: We tightened and segmented the whole Introduction section to improve clarity and flow. Specifically, we broke up long, multi-clause sentences, trimmed repeated “ML superiority” statements and shifted to active voice. The lead-in now appears as two compact, signposted paragraphs: one that concisely summarizes ML capabilities and limitations (page. 4, lines 65-73) and a second that foregrounds the Bangladesh context, the married-women rationale and the study aim (page. 6-7, lines 118-135). Subsequent paragraphs were similarly shortened with clearer transitions (e.g., separating cardiovascular ML use cases, imbalance/metrics, SHAP interpretability and the subgroup gap/BDHS-2022 plan) so key ideas are introduced in smaller, readable units while surfacing the programmatic target screening within existing service touchpoints earlier in the narrative.**

Comment: Explicitly state whether this study is the first in Bangladesh to use such an extensive set of algorithms and class balancing approaches for hypertension prediction in married women, highlighting its unique contributions.

Response: **To our knowledge, this is the first Bangladesh study to evaluate an extensive suite of algorithms with class-balancing and SHAP interpretability specifically for married women (page. 7, lines 129–131**

Comment: Address limitations or challenges posed by the dataset (e.g., self-reported data, cross-sectional nature) up front to set proper expectations.

**Response: We moved dataset-related caveats to the front of the paper to set expectations. The Introduction now notes that BDHS-2022 was unavailable at analysis and that models were trained on BDHS-2017–18 with pre-specified external validation on BDHS-2022 (page. 6, lines 124-126). We added that the BDHS’s cross-sectional design precludes causal inference, results are associational, not causal (page. 35,37 lines 514, 550). We also flag reliance on self-reported variables (e.g., contraceptive use, recent births) with potential recall/measurement error and the limited biomarker scope (primarily BMI and capillary glucose) (page. 10, lines 199-200). Generalizability is explicitly bounded to married women sampled by BDHS (page. 7-8, lines 143–156). The Intro–Methods bridge reiterates the external-validation plan and cites DHS de-identification/ethical safeguards (page. 6,12 lines 124–126,265-272).**

Comment: Consider including a brief mention of ethical considerations when using big data or advanced analytics in public health research.

**Response: We added an explicit fairness/ethics sentence: “We additionally highlight that fairness and bias are critical in ML health research. Sensitive variables were treated with caution and SHAP interpretability was used to ensure transparency in feature attribution. These safeguards support responsible use of advanced ML in public health policy.” (page.15, lines 297-300).**

Comment: The transition into the objectives section could be improved for more seamless narrative connection between context and study aims.

**Response: We added a bridging objective sentence that ties context to aims: “Therefore, the objective of this study is to develop and validate interpretable machine-learning models for predicting hypertension among married women in Bangladesh, integrating sociodemographic, behavioral, and biometric factors (such as BMI and diabetes status), to identify the most influential predictors and provide evidence to guide targeted screening and public health interventions in low-resource settings**

**” (page.6-7, lines 131-135)**

Comment: Some terminology (e.g., differentiating predictive versus explanatory modeling) can be clarified to aid a broad audience.

**Response: We clarify this distinction by repeatedly emphasizing association≠causation and SHAP’s role as an explanatory (interpretability) tool rather than a causal estimator (e.g., “Models quantify associations rather than causation,” page.2, line 33; and “SHAP values explain model associations, not causal effects,” page.35, line 514;).**

**Methodology**

Comment: The lack of validation using independent/external data is a key limitation. While acknowledged, this impacts model generalizability beyond the sampled population. Future work with the now-available BDHS 2022 should be prioritised.

**Response: We brought external validation forward and repeated it in Methods and Discussion: “At the time of model development, BDHS-2022 data was not publicly released; consequently, model training and internal validation used BDHS-2017-18. BDHS-2022 is now available and differs modestly in feature scope; to avoid design leakage, we will treat BDHS-2022 strictly as an external test set. Given the scarcity of comparable datasets of the same type, we also reserve BDHS-2022 for validating the deployed system. No retraining or feature re-specification will be performed for the external test; instead, we will apply identical preprocessing and the pre-specified decision threshold” (page.13, lines 265–271) and “as BDHS 2022 was not released then. Since the dataset is now available, we plan to validate our deployed system using BDHS 2022, though its limited feature scope prevents full integration into model training…” (page.37, line 556-558). This sequence was not explicitly laid out previously.**

Comment: While a wide set of features is included, the methodology could elaborate whether feature selection (beyond literature-based inclusion) was performed before modeling, possibly using wrapper/filter/embedded methods.

**Response: We now explicitly state no automated pre-model selector was used: “All sociodemographic, behavioral, biometric and anthropometric variables with theoretical or empirical relevance to hypertension were extracted from the BDHS 2017–18 dataset. No additional feature engineering or automated feature selection algorithms (e.g., Boruta, LASSO or recursive feature elimination) were applied. Instead, variable inclusion was guided by existing epidemiological literature and prior BDHS-based hypertension studies…” (page.10, lines 199-203). SHAP is identified as the post-hoc interpretability framework.**

Comment: The methodology briefly mentions deletion but does not elaborate on the extent or potential bias induced by this approach versus imputation.

**Response: We now make missing-data handling explicit and quantified in Methods → Data preparation → “Missing data” (page. 9, lines 206–207): we conducted a complete-case analysis (final n=4,253 married women; 4,546 initially eligible), excluding pregnant respondents and records with missing values; no imputation was performed to avoid model-dependent artifacts in a mixed categorical feature set (page. 7–8, lines 143–156). To show downstream effects, Methods and Results report the hypertension class distribution used for modeling (overall prevalence ≈23.1%), the training/test splits (page. 12, lines 242–243) and the class distributions before and after each resampling method, directing readers to the “Class distribution before and after class balancing” block and sampler-specific counts (page. 19, lines 342–350 and Supplement). These additions clarify what was retained versus excluded, why imputation was not used and how balancing altered minority-class representation, enabling assessment of any bias introduced by deletion versus imputation.**

Comment: Training was performed on basic hardware (i5 CPU, 8GB RAM)—potentially limiting more computationally intensive models or hyperparameter searches.

**Response: We state compute constraints plainly: “Training was carried out on a workstation with Intel Core i5-8365U … 8 GB RAM … resource limitations constrained deeper architectures; the chosen algorithms were successfully optimized …” (page. 13-14, lines 275-278).**

Comment: No mention of use of survey/sample weights, which are critical for BDHS data to ensure national representativeness. Analytical weighting should be addressed or justified if omitted.

**Response: We created a Survey weights subsection and justified the modeling choice: heading “Survey weights” and “The BDHS 2017–18 employs a complex survey design with stratification, clustering and sampling weights to ensure national representativeness. In the present study, we did not apply survey/sample weights because the primary aim was methodological focused on evaluating and comparing the predictive performance of class balancing approaches integrate with machine learning algorithms and ranked risk and protected features rather than estimating population-level prevalence or nationally representative parameters. This approach is consistent with prior ML studies using DHS data in similar contexts (page. 10, line 209-215).**

Comment: While IRB considerations are reported, the rationale and implications of using advanced ML (including fairness or bias issues) could be further discussed under ethical considerations.

**Response: We added ethics/bias language to complement IRB statements: “As this study involves secondary data analysis, we obtained permission to access the de-identified data from the DHS Program. Since the data was de-identified and publicly available, no additional ethical approval was required for this analysis. The study adhered to the relevant guidelines and regulations for secondary data use.**

**We additionally highlight that fairness and bias are critical in ML health research. Sensitive variables were treated with caution and SHAP interpretability was used to ensure transparency in feature attribution. These safeguards support responsible use of advanced ML in public health policy. (page.14-15, line 293-300).**

Comment: Rationale for preferring Extra Trees, as opposed to XGBoost/Random Forest (other top performers in similar studies), could be more clearly articulated within the methods section, not just in the discussion.

**Response: We now state the methodological rationale inside Methods → Machine learning algorithms (page. 12, lines 237–239): Extra Trees was included because it is computationally efficient under extensive resampling, less sensitive to depth/learning-rate tuning than boosted trees, reduces variance via fully randomized split thresholds, and yields stable feature-importance profiles complementing RF’s bootstrap splits and XGBoost’s gradient-based boosting (92–94). We also justify the choice empirically in Results → “Classification efficacy and confusion matrix” (page. 25, lines 377–378), where paired confusion matrices and bootstrap 95% CIs in the consolidated performance table show Extra Trees + SMOTE+ENN delivering the top F1/AUC-PR while maintaining comparable ROC-AUC versus RF/XGBoost.**

Comment: Clearly define composite or derived variables (e.g., explain calculation for BMI and diabetes status) in a dedicated subsection for reproducibility.

**Response: We created a dedicated “Derived variables” subsection in Methods and defined each composite variable precisely for reproducibility: Diabetes status is based on fasting plasma glucose measured with the HemoCue Glucose 201 DM (plasma conversion), with values auto-converted to FPG equivalents; diabetes is coded if FPG ≥ 7.0 mmol/L or if antihyperglycemic medication use is self-reported (page. 9, lines 188–194). Body mass index (BMI) is calculated as weight/height² (kg/m²) and categorized using WHO cut-offs: underweight < 18.5, normal 18.5–24.9, overweight 25.0–29.9, and obese ≥ 30 (page. 9, lines 195–197).**

**Results**

Comment: The results could benefit from consolidation, such as combining tables and figures for easier cross-comparison between different algorithms and balancing techniques.

**Response: We reorganized the Results to enable side-by-side comparison across algorithms and balancing techniques. A single consolidated performance table (Table 5) now reports all model×sampler metrics, and we place the paired 2×2 confusion matrices for ExtraTrees—original vs. SMOTE+ENN adjacently (Tables 3–4) under the new “Classification efficacy and confusion matrix” subsection see page 25 lines 367-387. We added a compact “Global comparison of class-balancing techniques” block that centralizes the Anderson–Darling normality check (Supplementary S2A), the AUC-PR ANOVA with Tukey HSD (S2B–S2C) and Friedman ranks for non-normal metrics (S2D–S2E) see page 28-29 lines 388-399. To reduce fragmentation, trend visualizations are grouped as boxplots (Figs. 4–11) by metric and the “Class distribution before and after class balancing” subsection reports pre/post sampler counts to contextualize performance shifts see page 19 lines 342-350. Test-set uncertainty for the selected model is summarized once in a bootstrap CI table (Table 6) see page 30 lines 432-435. Furthermore, we reported “Internal overfitting diagnostics” and “Calibration and thresholding” subsection with tables and figure together (see page 30. Lines 426-430 and page. 32, lines 455-459)**

Comment: Presenting confidence intervals or statistical uncertainty for key metrics (not only point estimates) would strengthen result reporting and allow readers to assess model reliability.

**Response: We agree and now report 95% nonparametric bootstrap CIs for all test-set metrics of the selected ExtraTrees + SMOTE+ENN model (Results, page.31, lines 432-437; Table 6). Specifically, with 1,000 bootstrap replicates on the held-out test set (n=672) we obtain: Precision 0.92 (0.90–0.94), Recall 0.95 (0.93–0.97), F1 0.94 (0.92–0.96), Accuracy 0.91 (0.89–0.94), Specificity 0.83 (0.80–0.87), ROC-AUC 0.95 (0.93–0.97), PR-AUC 0.95 (0.93–0.97), Cohen’s κ 0.79 (0.75–0.83), MCC 0.79 (0.75–0.83), and G-mean 0.89 (0.86–0.92). We also clarify the evaluation protocol: class balancing at the individual level, metrics computed at the pre-specified decision threshold (0.55), with percentile CIs). In addition, pairwise AUC-PR comparisons include 95% CIs via Tukey’s HSD (Supplementary Table S2C).**

Comment: The narrative sections of the results could be more concise and focused, with clearer guidance to the reader on the most clinically or policy-relevant findings.

**Response: To improve concision and orient readers to clinically/policy-relevant findings, we colocated interpretability outputs with the consolidated model×sampler table and the paired confusion matrices so the path from discrimination to intervention levers is explicit (page.25, lines 367–387; page.20, lines 352–363). We grouped cross-technique trends and uncertainty into compact summaries a single “Global comparison of class-balancing techniques” block (ANOVA/Tukey for AUC-PR; Friedman ranks for non-normal metrics) and a one-page bootstrap-CI table for the selected model (page.28–29, lines 388–399; page.30, lines 432–435). We also present SHAP rankings alongside factor-direction plots (Figs. 12a–c) to highlight actionable determinants BMI (overweight/obesity, positive), reproductive profile (≥2 living children; limiting/traditional contraception, positive), and spousal education (secondary, positive) and protective strata (age <35, under/normal weight, normoglycemia, negative), with brief, reader-facing text linking these patterns to feasible levers (page.29–30, lines 406–421).**

Comment: Visualisation of SHAP values (e.g., summary plots) and confusion matrices should be clearer and referenced in the main results narrative, rather than relegated to supplementary materials.

**Response: We made SHAP and confusion matrices prominent in the main text: “Integrating SHAP with a trained model enabled analysis of global and local predictors. Figure 12a shows the most important features; Fig 12b classifies the top 20 factors by direction of association; Fig 12c presents a SHAP summary plot combining feature importance and effects.**

**In Fig 12a, respondent age <35 years was the most influential factor, followed by overweight, having two to three ever-born children, and husband/partner with secondary education. Additional contributors included underweight, normal weight, husband/partner <35 years, >2 living children, no births in the last five years and obesity.**

**Fig 12b indicates negative associations (blue) for age <35, underweight/normal weight, husband/partner <35 years, respondent age 35–40, one child in last five years, normoglycemia, residence in Rajshahi and no formal education for husband/partner. Positive associations (red) included overweight, two to three ever-born children, husband/partner with secondary education, >2 living children, no births in five years, obesity, husband/partner >40, traditional contraceptive use, unmet need for limiting, richest quintile and age difference ≥10 years.**

**Fig 12c shows these effects at the individual level: blue points (low SHAP values) correspond to features decreasing predicted risk, while red/purple points (high SHAP values) correspond to features increasing predicted risk” (page.29-30, lines 406-421) and the results section headings and table captions for Table 3 and Table 4 (page.25-26, lines 367-382). This moves key visual evidence into the primary narrative.**

Comment: Greater emphasis on findings that differ from previous studies, especially regarding novel risk/protective factors found important by the chosen machine learning models.

**Response: We agree and now foreground where our results extend or differ from prior reports. In the Abstract and again in Results we added explicit contrasts: “SHAP identified age, parity and recent births, contraceptive pattern, spousal education, and BMI as key predictors; beyond established risks (age ≥40, overweight/obesity), the models highlight program-relevant levers traditional method use and unmet need for limiting, spousal secondary education, larger age-gap (≥10 years) and region (Rajshahi) as independent contributors. Younger age (<35) and normal/underweight status were protective.” (Abstract, page.2, line 38-41; Results, page29-30, lines 409–418; Discussion, page.34–35, lines 497–513).**

Comment: The discussion of limitations in the results section should be expanded: for example, how the internal validation-only approach may exaggerate performance (potential overfitting).

**Response: We expanded the limitations and flagged potential optimism from internal validation. Concretely, we added an Internal overfitting diagnostics block with repeated stratified 5-fold CV, nested CV, and learning-curve checks (Results) and we now report 95% nonparametric bootstrap CIs (1,000 reps) for all test-set metrics of the selected ExtraTrees+SMOTE+ENN model (Results, page.31,lines 432-437; Table 6).. We also document safeguards against leakage/overfitting: class balancing applied inside training folds only; the test set remained untouched; the final model was refit once on the full training set (Sample-flow/class balance, page.32, lines 447–453); metrics were computed at a pre-specified threshold (0.55) with calibration reported (Brier = 0.0626) (Calibration/thresholding; Supplementary Table S3B p.32, lines 455–459);). Subgroup diagnostics reveal heterogeneity (e.g., slightly lower F1 in Rajshahi despite ROC-AUC = 1.0), reinforcing our caution about generalizability (Supplementary Table S6 p.32, lines 461–463). We state explicitly that external, out-of-sample validation and local recalibration are prerequisites for deployment, and that current results while uncertainty-quantified may still be optimistic relative to prospective performance.**

Comment: More explicit tracking and reporting of sample sizes for each analysis (post-imputation, balancing) would enhance transparency.

**Response: We made sample sizes explicit at every stage and added two audit-style elements. First, a new “Class distribution before and after class balancing” block enumerates training-set counts and proportions by method: original train n=3,581 with 827 hypertensive (23.1%) and 2,754 normotensive (76.9%); SMOTE and ADASYN 50/50; Tomek Links 24.7% minority; ENN 35.0% minority; SMOTE+Tomek 50/50; SMOTE+ENN 63.3% hypertensive (Results, page.19, lines 342–350; Supplementary Table S1). Second, a CONSORT-style sample-flow records N=4,253 , train n=3,581 / test n=672, documents that stratified 5-fold CV preserved the 23.1% prevalence within each fold and notes that balancing was applied inside training folds only (Results, page.32, lines 447–453; Supplementary Tables S4A–S4C). We also print raw counts in the paired confusion matrices (Tables 3–4; Results, page.25–26, lines 367–382) and state the analysis population in the CI table (n=672 in Table 6; Results, page.31, lines 432–437), so denominators are clear wherever metrics are reported.**

**Discussion**

Comment: The discussion could more robustly address how findings might translate to policy or clinical practice, with specific recommendations or scenarios.

**Response: To translate findings into policy and practice, we now present concrete, scenario-based pathways alongside deployment guardrails. We describe embedding risk scoring in e-registries, integrating routine blood-pressure checks into family-planning and postpartum visits, engaging husbands via brief education/SMS, prioritizing high-parity households and tailoring outreach by district (e.g., Rajshahi) (Discussion, page.34–35, lines 507–513). Policy use is explicitly linked to the Youden-optimal/pre-specified decision threshold (0.55), with guidance to recalibrate thresholds to local prevalence and the relative costs of false negatives versus false positives (Results, page.32, lines 455–459). We add equity and governance safeguards—ongoing subgroup-level fairness monitoring, transparent model versioning and threshold documentation, and mitigation of potential harms such as stigma or resource diversion and note complementary diet strategies (green coffee; DASH/Mediterranean) to support implementation (Discussion, page.34–35, lines 507–513). The Conclusion reiterates how these pathways can be operationalized in LMIC health systems and underscores external validation as a prerequisite to deployment (Conclusion, page.36–37, lines 528–558).**

Comment: Limitations could be expanded beyond technical aspects: for example, discuss the impact of cultural/social determinants, potential biases in BDHS sampling, or gender-specific barriers to care in Bangladesh.

**Response: We broadened the limitations to encompass social and structural factors, potential sampling biases, and gendered access barriers. The manuscript now notes that BDHS may under-represent some high-risk or marginalized groups, bounding generalizability; that gender-specific barriers in Bangladesh (e.g., mobility constraints, caregiving burden, norms around clinic attendance) can limit care-seeking and program uptake; and that fairness governance (subgroup performance monitoring, transparent versioning/thresholds, mitigation of unintended harms) is required for responsible use. These additions appear in the Discussion (p.34–35, lines 499–520) and the Strengths and limitations section (page.36–37, lines 545–558), and are grounded by earlier context on the BDHS sampling frame and our weighting rationale Methods—Survey weights, page.10, lines 209–215). We also reiterate cross-sectional and self-report constraints (associations ≠ causation).**

Comment: The generalizability section could better address the representativeness not only for married women, but also the implications for unmarried women or other populations left out of the dataset.

**Response: We explicitly bound generalizability to the analytic population and spell out who is not covered. The Discussion clarifies that findings reflect married women sampled by BDHS and may not extend to unmarried women or under-represented subgroups; we note potential BDHS under-sampling of marginalized populations and advise fairness monitoring and local threshold recalibration to prevalence and error costs (Discussion, page.34–35, lines 507–513). We pre-specify external validation on BDHS-2022 to test discrimination, calibration, and threshold transfer in a later, post-COVID cohort and under any instrument changes (Discussion, page.35, lines 523–526). The Strengths and limitations section reiterates these population bounds and sampling caveats (p.36–37, lines 545–558). Earlier context on the BDHS sampling frame and our weighting rationale further anchors representativeness (Methods, page.7–8, lines 143–156; Methods,Survey weights, page .10, lines 209–215).**

Comment: Interpretation of the predictive models would benefit from a more detailed discussion of their limitations in distinguishing correlation from causation, especially in feature importance ranking.

**Response: We now distinguish correlation from causation explicitly across the manuscript. The Abstract states, “Models quantify associations rather than causation” (page.2, lines 33–34). In Methods, SHAP is framed as a post-hoc interpretability tool not a causal estimator and we note that its attributions reflect the fitted model and observed covariate structure (Methods—Interpretability, page.10–11, lines 199–206). The Discussion reiterates that “SHAP values explain model associations, not causal effects,” cautions that correlated social and fertility indicators may function as proxies, and flags counter-intuitive signals (e.g., husbands’ secondary education) as hypotheses for follow-up rather than causal claims (Discussion, page.35, lines 514–521). The Strengths and limitations section restates the cross-sectional design and non-causal nature of feature rankings (page.36–37, lines 545–558). We also report sensitivity checks on correlated predictors and emphasize that policy use should follow external validation and, where appropriate, causal or quasi-experimental evaluation.**

Comment: Ethical considerations of applying ML methods—like fairness, interpretability, and unintended consequences—should be mentioned.

**Response: Ethical considerations are now woven throughout the manuscript. The Introduction frames ML’s promise alongside risks, explicitly noting the need for interpretability and fairness in public-health use (page.4, lines 65–73). Methods include an Ethics/Fairness subsection that explains our use of de-identified BDHS data, careful handling of sensitive attributes, reliance on SHAP for transparent explanations, and plans for subgroup performance checks (page.14–15, lines 293–300). The Discussion adds deployment guardrails routine fairness monitoring, transparency via model versioning and documented thresholds, calibration to local prevalence and error costs, and mitigation of unintended consequences such as stigma or resource diversion (page.34–35, lines 507–513). Finally, the Strengths and limitations section reiterates that fairness audits revealed subgroup gaps requiring monitoring and mitigation, and that external validation is a prerequisite for any real-world use (page.36–37, lines 545–558).**

Comment: The conclusion could offer a more detailed roadmap for future studies, specifying what improvements or validations (e.g., external datasets, prospective studies) are most urgent.

**Response: We now provide a prioritized roadmap for future work: (i) strict external validation on BDHS-2022 using identical preprocessing and the pre-specified decision threshold to assess discrimination, calibration, and transportability; (ii) prospective/temporal validation with local recalibration and threshold tuning to reflect prevalence and error costs; (iii) enrichment with clinical/biometric markers; (iv) routine fairness audits with subgroup-gap mitigation; (v) extension of modeling to unmarried women and other under-represented groups; (vi) transfer learning and cross-country adaptation; (vii) linkage to real-time eRegistry streams for adaptive monitoring and thresholding; and (viii) exploratory multi-omics integration where feasible. These priorities are detailed in the Conclusion (p.36, lines 534–541) and align with the operational pathways outlined in the Discussion (p.34–35, lines 507–513).**

Best regards,

Novel Chandra Das

Research officer, icddrb
